# A multi-substrate screening approach for the identification of a broadly applicable Diels–Alder catalyst

**DOI:** 10.1038/s41467-019-08374-z

**Published:** 2019-02-15

**Authors:** Hyejin Kim, Gabriela Gerosa, Jonas Aronow, Pinar Kasaplar, Jie Ouyang, Julia B. Lingnau, Paul Guerry, Christophe Farès, Benjamin List

**Affiliations:** 1 Max-Planck-Institut für Kohlenforschung, Kaiser-Wilhelm-Platz 1, D-45470 Mülheim an der Ruhr, Germany; 2 Therapeutics & Biotechnology Division, Korea Research Institute of Chemical Technology, 34114 Daejeon, Republic of Korea

## Abstract

When developing a synthetic methodology, chemists generally optimize a single substrate and then explore the substrate scope of their method. This approach has led to innumerable and widely-used chemical reactions. However, it frequently provides methods that only work on model substrate-like compounds. Perhaps worse, reaction conditions that would enable the conversion of other substrates may be missed. We now show that a different approach, originally proposed by Kagan, in which a collection of structurally distinct substrates are evaluated in a single reaction vessel, can not only provide information on the substrate scope at a much earlier stage in methodology development, but even lead to a broadly applicable synthetic methodology. Using this multi-substrate screening approach, we have identified an efficient and stereoselective imidodiphosphorimidate organocatalyst for scalable Diels–Alder reactions of cyclopentadiene with different classes of *α*,*β*-unsaturated aldehydes.

## Abstract (web summary)

Investigation of a reaction scope usually starts with the optimization for a model substrate. Here, the authors apply a time-efficient multi-substrate screening approach to identify a general organocatalyst for the Diels–Alder reaction of cyclopentadiene with* α*,*β*-unsaturated aldehydes.

## Introduction

The Diels–Alder reaction is one of the most powerful transformations in chemical synthesis and generates six-membered cyclic products with up to four stereogenic centers. Among the most notable advances of this reaction has been the development of catalytic asymmetric versions. In addition to a variety of chiral Lewis acid catalysts^1–3^, organic molecules also catalyze Diels–Alder reactions stereoselectively^4–6^. Moreover, the Diels–Alder reaction between *α*,*β*-unsaturated aldehydes and dienes has attracted particular attention due to not only the synthetic utility of aldehydes, but also its wide applicability in the synthesis of drugs, natural products, and fragrances^7^. For example, chiral secondary amines catalyze highly enantioselective Diels–Alder reactions of *β*-monosubstituted enals^8–13^. However, these catalysts typically give moderate diastereoselectivities and cannot readily be used with *α*-substituted enals^14–16^. In addition, most reported catalysts that convert *α*-substituted enals display a limited scope in terms of tolerated substituents^1–3,14–20^. Despite intense research in this area, a general catalyst of the Diels–Alder reaction that can accommodate a broad range of structurally distinct enals with cyclopentadiene has yet to be developed.

**Fig. 1:**
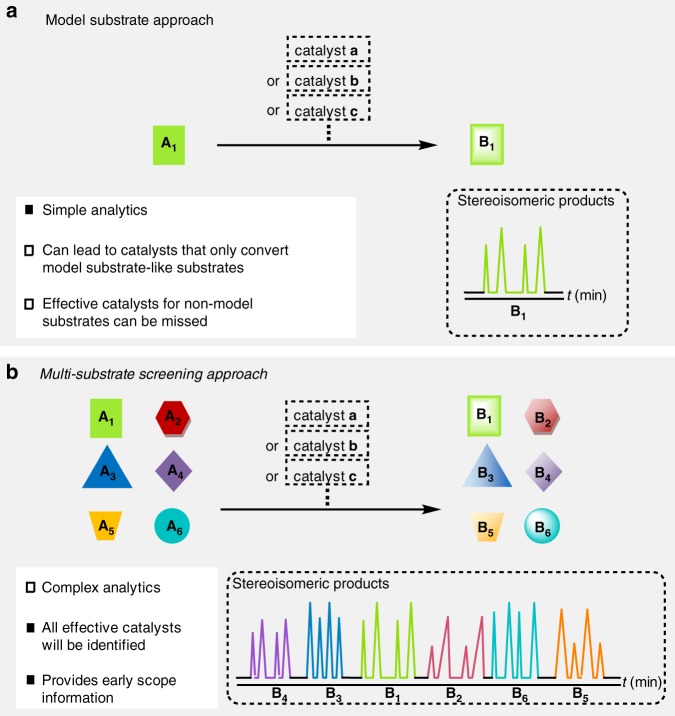
Two approaches to identify a selective catalyst. **a** Model substrate approach vs. **b** multi-substrate screening approach

Realizing limitations of the model substrate approach (Fig. 1a), Kagan et al. suggested a catalyst screening that involves the utilization of a pooled collection of different substrates as an alternative method (Fig. 1b)^21^. The concept has originally been exemplified in the asymmetric reduction of ketones and has subsequently been applied to identify chiral ligands for metal-catalyzed asymmetric transformations such as additions of diethylzinc, hydroformylations, and hydrogenations^22–25^. The two main advantages of multi-substrate screenings are that information about the scope of a catalyst is revealed early on, and that catalysts that perform suboptimally with the model substrate but excellently with others, can still be identified. Challenges of multi-substrate screenings include the difficulty of developing an accurate analytical assay that can simultaneously differentiate all substrates and all stereoisomeric products. Furthermore, unproductive interactions between substrates and products as well as kinetic competition may complicate the analytics and exacerbate the readout of the assay. Nonetheless, the multi-substrate screening approach has not previously led to a general and broadly applicable catalyst and we became interested in applying it to a challenging synthetic problem. We report here the use of a multi-substrate screening approach for the development of a general catalyst of the asymmetric [4+2] cycloaddition of diverse *α*,*β*-unsaturated aldehydes with cyclopentadiene.

## Results

### Assay development

**Fig. 2:**
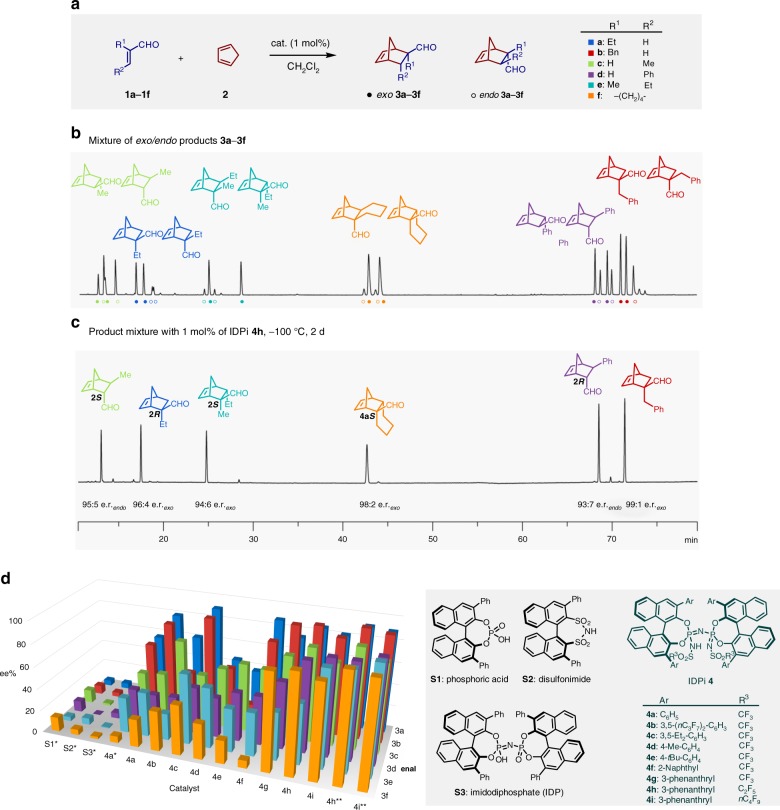
Multi-substrate screening of Diels–Alder reactions of *α*,*β*-unsaturated aldehydes and cyclopentadiene. **a** Investigated reactions. **b** GC chromatogram of the stereoisomeric product mixture. **c** Chromatogram of the product mixture using catalyst **4h**. **d** Graphical representation of a subset of the multi-substrate screening. *Reactions at room temperature. **Reactions at −100 °C. For detailed reaction conditions and results, see Supplementary Note 2

We initiated our studies by establishing an assay to screen several structurally distinct enal dienophiles simultaneously (Fig. 2a). Six representative *α*,*β*-unsaturated aldehydes were selected according to the position, nature, and size of the substituents (**1a**–**1f**), and to enable product separation on a single chiral stationary gas chromatography (GC) phase. In addition to *α-* and *β*-monosubstituted enals **1a**–**1d**, substrates **1e** and **1f** were chosen as acyclic and cyclic *α*,*β*-disubstituted enals. When a representative of a particular substrate class led to product peak overlaps, a minor modification quickly provided baseline separation. For example, the ethyl-substituted aldehyde **1a** was employed instead of its methyl analog to circumvent product peak overlap with cycloadduct **3c** during GC separation. Gas chromatography with 2,3-dimethyl-6-tert-butyldimethylsilyl-*β*-cyclodextrin as chiral stationary phase was applied to measure the stereoisomeric ratios of all possible products in the crude mixture. A chromatogram with 23 distinct peaks (*endo* **3b** could not be separated under the described condition) was established and used as the platform for the multi-substrate screening (Fig. 2b).

### Catalyst screening with multiple substrates

With this robust analytical protocol established, we carried out a simultaneous catalyst screening using pooled substrates. Different Brønsted acid catalysts including phosphoric acid **S1**^26,27^, disulfonimide **S2**^28,29^, and imidodiphosphate **S3**^30^ all provided the desired cycloaddition products, albeit sometimes with moderate reactivity and generally with poor stereoselectivities (Fig. 2d and Supplementary Table 1). A remarkable improvement of reactivity and stereoselectivity was observed using highly acidic and confined imidodiphosphorimidate (IDPi) catalyst **4a**^31–34^, and further enhancement of enantiomeric ratios in all reactions was observed at −78 °C. Thus, we focused on screening IDPi catalysts for the stereoselective cycloaddition of *α*,*β*-unsaturated aldehydes (**1a**–**1f**) and cyclopentadiene **2** (Fig. 2d, for detailed results on the optimization of reaction conditions, see the Supplementary Table 2).

From screening a wide range of IDPi catalysts representatively depicted in Fig. 2d, we found that catalyst **4c** showed excellent stereoselectivity exclusively with *α*-substituted enals (**3a**: 92:8 *exo*/*endo*, 95.5:4.5 e.r._*exo*_; **3b**: 91:9 *exo*/*endo*, 94.5:5.5 e.r._*exo*_), while aldehydes having *β*-substituents (**1c**–**1f**) were not converted efficiently and stereoselectively (**3c**: 10:90 *exo*/*endo*, 73:27 e.r._*endo*_; **3d**–**3f**: < 5% conv.). In contrast, catalyst **4e** imparted higher enantioselectivities for *β*-monosubstituted dienophiles **1c** and **1d** (**3c**: 6:94 *exo*/*endo*, 81:19 e.r._*endo*_; **3d**: 2:98 *exo*/*endo*, 72:28 e.r. _*endo*_). Notably, the most favorable results were obtained using IDPi catalysts involving polycyclic aromatic substituents at the 3,3′-positions of the 1,1′-bi-2-naphthol (BINOL) backbone. For example, catalyst **4g** gave relatively high stereoselectivities when converting enals **1a**–**1f**. Further investigations to tune the chiral environment of the catalyst by modifying the inner substituent R^3^ improved the enantioselectivities of the major diastereomers (**4g**–**4i**)^33,34^. Finally, we were delighted to find that catalysts **4h** and **4i** showed excellent reactivity and stereoselectivity for all pooled *α*,*β*-unsaturated aldehydes (**1a**–**1f**), achieving full consumption of all dienophiles with only 1 mol% of the catalyst (Figs. 2c, d, for detailed reaction conditions and results, see the Supplementary Tables 2–5).

## Discussion

Importantly, individual experiments using aldehydes **1a**–**1f** separately corresponded well to the multi-substrate experiments, providing consistently high yields and stereoselectivities. Moreover, the optimized catalytic conditions were efficient for reacting a broad range of *α*,*β*-unsaturated aldehydes (Table 1). Variations of the size of the *α*-substituent were well tolerated, giving *exo*-enriched cycloadducts with excellent enantioselectivities (**3a**, **3b**, **3g**–**3j**; 96:4–99:1 *exo*/*endo*, 93:7–99:1 e.r._*exo*_). In contrast, dienophiles bearing an aromatic substituent at the *β*-position yielded highly enantioenriched *endo* products (**3d**, **3p**–**3t**; 3:97–1:99 *exo*/*endo*, 94:6–97:3 e.r._*endo*_) while *β*-aliphatic substituents gave moderate to good diastereoselectivities (**3c, 3k**–**3n**; 25:75–4:96 *exo*/*endo*, 91:9–96:4 e.r._*endo*_). Consistent with the observation during the multi-substrate screening, *α*,*β*-disubstituted substrates furnished *exo*-products in good yields with high diastereo- and enantioselectivities (**3e**, **3f**, **3u** and **3v**). Acrolein afforded product **3w** in relatively good yield and stereoselectivity. The reaction was also tolerant of functional groups such as an alkene, a silyl ether, halides, and heteroarenes at the dienophiles, maintaining high yields and stereoselectivities (**3h**–**3j**, and **3o–3t**). Notably, all dienophiles (**1a**–**1w**) underwent the asymmetric Diels–Alder reaction with cyclopentadiene **2** with generally good to excellent diastereoselectivity and enantioselectivity in the presence of either catalysts **4h** or **4i**.

**Table 1:** Substrate scope of the Diels–Alder reaction

Reactions were performed with *α*,*β*-unsaturated aldehydes **1** (0.3 mmol), cyclopentadiene **2** (5.0 equiv.) and IDPi catalyst **4h** (1 mol%) in CH_2_Cl_2_ (0.3 mL) at −100 °C or −110 °C for 2–5 days. All yields are those of isolated products. Diastereomeric ratios (*exo*/*endo*) were determined by ^1^H nuclear magnetic resonance (NMR) analysis and enantiomeric ratios (e.r.) were determined by GC or high pressure liquid chromatography (HPLC) analysis. The relative and absolute configurations of cycloadducts were determined by comparison of the data with those reported. ^a^Catalyst **4i** was used. See the Supplementary Methods.

**Fig. 3:**
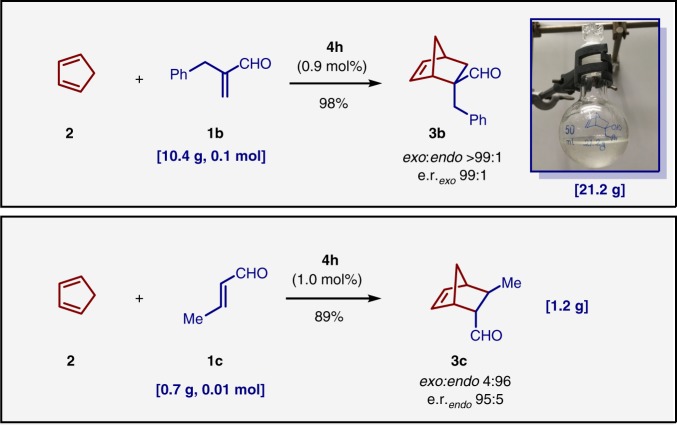
Large scale experiments. Two gram scale experiments were conducted with each cyclopentadiene and aldehyde **1b** and aldehyde **1c**, respectively. 21.2 g of pure product **3b** was obtained

Furthermore, efficiency and preparative utility of our Brønsted acid catalyzed reaction was demonstrated by large scale experiments with aldehydes **1b** and **1c** (Fig. 3). A decagram scale reaction using 0.1 mol of aldehyde **1b** and only 0.9 mol% of catalyst **4h** was performed to afford 21 g of product **3b** (98% yield, *exo*/*endo*, > 99:1, 99:1 e.r._*exo*_). A 0.01 mol scale reaction of aldehyde **1c** and cyclopentadiene **2** with 1 mol% of catalyst **4h** furnished 1.2 g of product **3c** in high yield and stereoselectivities (89% yield, *exo*/*endo*, 4:96, 95:5 e.r._*endo*_). In both cases, catalyst **4i** was recovered by flash column chromatography and subsequent re-acidification (97% and 92%).

**Fig. 4:**
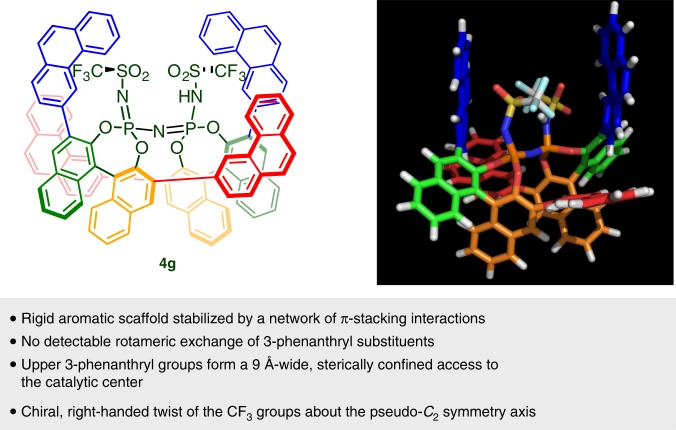
The solution structure of catalyst **4g**. Structural characteristics of our catalysts were revealed by using NMR-based models

To gain structural insight regarding the activity and stereoselectivity of the 3-phenanthryl-substituted IDPis, a 10-ns molecular dynamics simulation of **4g** was performed with distance restraints based on carefully evaluated nuclear Overhauser effect (NOE) contacts. The assembly of the BINOL backbone and its substituents bears strong similarity with other IDPi crystal structures^28,30,31^, and defines a rigid, narrow and chiral access to the catalytic center. The important structural features are summarized in Fig. 4 (see Supplementary Note 5, Supplementary Discussions 1, 2 and Supplementary Data 1 for details).

In conclusion, we report the discovery of a general, and scalable Brønsted acid catalyst of the asymmetric Diels–Alder reaction between structurally diverse *α*,*β*-unsaturated aldehydes and cyclopentadiene (2,3-dimethylbuta-1,3-diene could also be used, as described in the Supplementary Methods). While the optimal catalysts **4h** and **4i**, in this particularly fortuitous case, may have also been identified using single substrate approaches, other more specialized catalysts such as acid **4c** could have easily been missed. Our findings deliver a powerful Diels–Alder catalyst and suggest that multi-substrate screenings can aid in identifying broadly useful and highly stereoselective catalysts of challenging carbon–carbon bond forming reactions.

## Methods

### General procedure for the asymmetric Diels–Alder reaction

To a solution of the catalyst (1–3 mol%) in anhydrous CH_2_Cl_2_ (0.3 mL), immersed in a liquid nitrogen-ethanol slush at −116 °C, were added aldehyde **1** (0.3 mmol, 1.0 equiv) and diene **2** (5.0 equiv). The resulting mixture was stirred at the described temperature. Upon completion of the reaction, NEt_3_ (50 μL) was added and the reaction mixture was warmed to ambient temperature. After removal of the solvent, the crude mixture was purified by column chromatography to afford product **3**.

## Supplementary information

Supplementary Information

Description of Additional Supplementary Files

Supplementary Data 1

## Data Availability

The authors declare that the data supporting the findings of this study are available within the paper and its supplementary information files.
