# Supplementary material for: A multi-substrate screening approach for the identification of a broadly applicable Diels–Alder catalyst
Source: Nat Commun. 2019 Feb 15;10:770. doi: 10.1038/s41467-019-08374-z (PMC6377681; doi:10.1038/s41467-019-08374-z)
Supplement: Supplementary file 1 — Supplementary Information [file 41467_2019_8374_MOESM1_ESM.pdf]

**Supplementary Information**

A multi-substrate screening approach for the identification of a broadly applicable Diels−Alder catalyst

H. Kim *et al.*

**Supplementary Note 1.** General information

Unless otherwise stated, all reagents were purchased from commercial suppliers and used without further purification. Aldehydes were distilled and stored under argon prior to use. All solvents used in the reactions were distilled from appropriate drying agents prior to use. Reactions were monitored by thin layer chromatography (TLC) on silica gel pre-coated plastic sheets (0.2 mm, Macherey-Nagel) or glass plates (SIL G-25 $UV_{254}$, 0.25 mm, (Macherey-Nagel). Visualization was accomplished by irradiation with UV light at 254 nm and/or phosphomolybdic acid (PMA) stain. PMA stain: PMA (10 g) in EtOH (100 mL). Column chromatography was performed on Merck silica gel (60, particle size 0.040–0.063 mm). NMR spectra were recorded on Bruker AV-500, Bruker AV-400 or Bruker AV-300 spectrometer in deuterated solvents. Proton chemical shifts are reported in ppm (δ) relative to tetramethylsilane (TMS) with the solvent resonance employed as the internal standard ($CDCl_3$ δ 7.26 ppm; $CD_2Cl_2$ δ 5.32 ppm). Data are reported as follows: chemical shift, multiplicity (s = singlet, d = doublet, t = triplet, q = quartet, p = pentet, sext = sextet, h = heptet, m = multiplet, br = broad), coupling constants (Hz) and integration. $^{13}C$ chemical shifts are reported in ppm from tetramethylsilane (TMS) with the solvent resonance as the internal standard ($CDCl_3$ δ 77.16 ppm; $CD_2Cl_2$ δ 53.84 ppm). $^{19}F$, $^{31}P$ NMR spectra were referenced in ppm from $CCl_3F$ and $H_3PO_4$, respectively. High resolution mass spectra were determined on a Bruker APEX III FTMS (7 T magnet). All reported yields, unless otherwise specified, refer to spectroscopically and chromatographically pure compounds. Optical rotations were determined with Autopol IV polarimeter (Rudolph Research Analytical) at 589 nm and 20 or 25 °C. Data are reported as follows: $[\alpha]_{\lambda}^{temp}$, concentration (*c*; g/100 mL), and solvents. Enantiomeric ratios (e.r.) were determined by GC or HPLC analysis employing a chiral stationary phase column specified in the individual experiment, by comparing the samples with the appropriate racemic mixtures. Diastereomeric ratios (d.r.) were determined by $^{1}H$ NMR spectra of the crude reaction mixtures, GC or HPLC analysis employing a chiral stationary phase. The crystals were measured and analyzed in the department of Chemical Crystallography and Electron Microscopy at Max-Planck-Institut für Kohlenforschung. Data were face-indexed absorption corrected and scaled using the program SADABS (Bruker AXS, 2014). The structure was solved and refined using the programs SHELXS and SHELXL, both programs from G. M. Sheldrick (Göttingen, 2014).

## Supplementary Methods

### *Synthesis & Characterization of Catalysts (IDPis)*

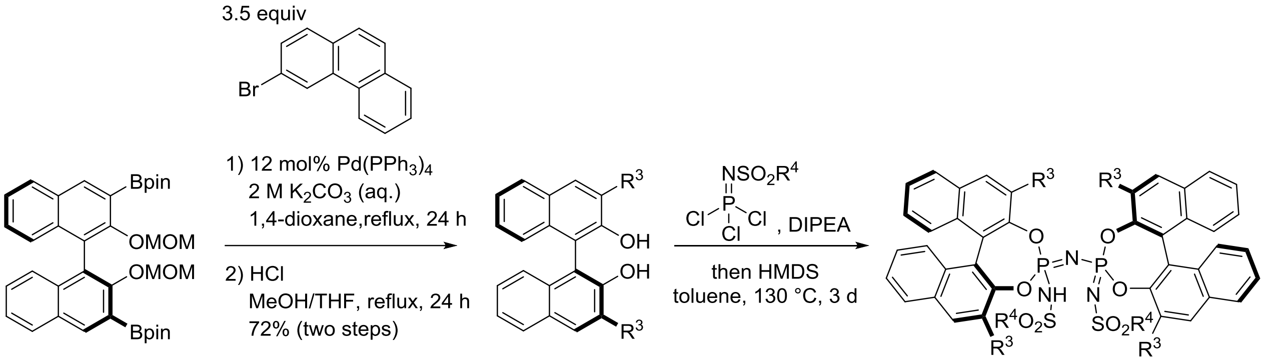


**S4**: $R^3$ = 3-phen

**4h:** $R^3$ = 3-phen, $R^4$ = $C_2F_5$
**4i:** $R^3$ = 3-phen, $R^4$ = $nC_4F_9$

**Supplementary Figure 1.** Synthesis of IDPi catalysts **4h** and **4i**.

**(*S*)-3,3'-di(phenanthren-3-yl)-[1,1'-binaphthalene]-2,2'-diol (S4)**

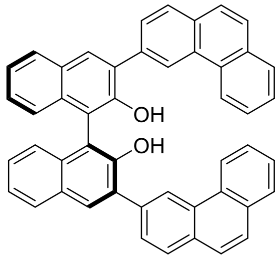


**S4**

To a flame-dried two-neck round-bottom flask with a condenser were added (*S*)-2,2'-(2,2'-bis(methoxymethoxy)-1,1'binaphthyl-3,3'-diyl)bis(4,4,-5,5-tetramethyl-1,3,2-dioxaborolane)[1] (3.0 g, 4.8 mmol, 1.0 equiv), 3-bromophenanthrene (4.3 g, 16.7 mmol, 3.5 equiv) and 1,4-dioxane (140 mL). After degassing the reaction mixture with argon for 20 min, tetrakis(triphenylphosphine)palladium (0.6 g, 0.56 mmol, 0.12 equiv) and a degassed solution of $K_2CO_3$ (2.0 M, aq., 19 mL) were sequentially added. The mixture was refluxed for 24 h. After cooling down to room temperature, HCl (10%, aq.) was added and the reaction mixture was extracted three times with $CH_2Cl_2$. The combined organic layers were dried over $Na_2SO_4$ and concentrated under reduced pressure. The residue was purified by recrystallization (hexanes : EtOAc = 90 : 10) to give (*S*)-3,3'-bis(3-phenanthryl)-2,2'-bis(methoxymethoxy)-1,1'-binaphthalene (2.8 g, 3.9 mmol, 80%) as a light yellow solid.

**$^1$H NMR** (500 MHz, $CDCl_3$): δ 8.98 (s, 2H), 8.72 (d, *J* = 8.2 Hz, 2H), 8.05 (s, 2H), 7.97 (dd, *J* = 8.2, 1.4 Hz, 2H), 7.87 (dd, *J* = 11.6, 8.2 Hz, 4H), 7.81 (d, *J* = 7.3 Hz, 2H), 7.68 (q, *J* = 8.9 Hz, 4H), 7.60–7.55 (m, 2H), 7.50 (dd, *J* = 10.8, 3.9 Hz, 2H), 7.38–7.30 (m, 4H), 7.26–7.22 (m, 2H), 4.40 (dd, *J* = 12.9, 5.9 Hz, 4H), 2.28 (s, 6H).

$^{13}$**C NMR** (126 MHz, $CDCl_3$): δ 151.8, 137.6, 135.9, 133.9, 132.4, 131.3, 131.2, 131.1, 130.6, 130.5, 128.8, 128.7, 128.5, 128.1, 127.3, 126.84, 126.82, 126.79, 126.7, 126.6, 125.5, 123.6, 123.0, 98.8, 56.2.

**HRMS** (ESI+) (*m*/*z*): calculated for $C_{52}H_{38}O_4Na_1$ $[M+Na]^+$: 749.2662; found 749.2665.

To a round-bottom flask charged with (*S*)-3,3'-bis(3-phenanthryl)-2,2'-bis(methoxymethoxy)-1,1'-binaphthalene (2.8 g, 3.9 mmol) in MeOH (100 mL) and THF (20 mL) was added HCl (6.0 M. aq., 20 mL) at room temperature. The reaction mixture was then refluxed for 24 h and concentrated under reduced pressure. The residue was purified by recrystallization (hexanes : EtOAc = 90 : 10) to give (*S*)-3,3'-di(phenanthren-3-yl)-[1,1'-binaphthalene]-2,2'-diol **S4** (2.2 g, 3.5 mmol, 90%) as a colorless solid.

$^{1}$**H NMR** (500 MHz, $CDCl_3$): δ 9.08 (s, 2H), 8.78 (d, *J* = 8.1 Hz, 2H), 8.22 (s, 2H), 8.06–7.96 (m, 6H), 7.92 (dd, *J* = 7.8, 1.2 Hz, 2H), 7.83–7.76 (m, 4H), 7.69–7.59 (m, 4H), 7.47–7.42 (m, 2H), 7.39 (ddd, *J* = 8.0, 6.7, 1.3 Hz, 2H), 7.35 (d, *J* = 8.4 Hz, 2H), 5.54 (s, 2H).

$^{13}$**C NMR** (126 MHz, $CDCl_3$): δ 150.5, 135.8, 133.3, 132.4, 132.0, 131.6, 131.1, 130.5, 129.77, 128.81, 128.7, 128.4, 127.7, 127.5, 126.9, 126.8, 126.73, 124.65, 124.6, 123.9, 123.0, 112.7 (peak overlap is observed).

**HRMS** (ESI–) (*m*/*z*): calculated for $C_{48}H_{29}O_2$ $[M–H]^–$: 637.2173; found 637.2172.

### ***N*,*N*'-((11b*S*,11b'*S*)-azanediylbis(2,6-di(phenanthren-3-yl)-4λ⁵-dinaphtho[2,1-*d*:1',2'-*f*][1,3,2]dioxaphosphepine-4-yl-4-ylidene))bis(1,1,2,2,2-pentafluoroethane-1-sulfonamide) (4h)**

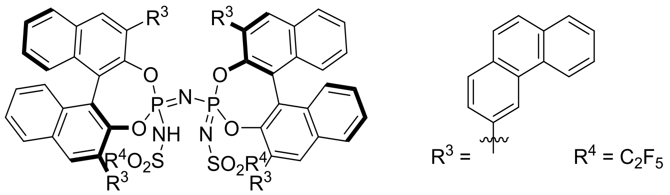

**4h**

In a flame-dried flask under argon, diol **S4** (1.0 g, 1.6 mmol, 2.1 equiv) was dissolved in toluene (10.6 mL, 0.15 M). Subsequently, *N*,*N*-diisopropylethylamine (DIPEA, 1.6 g, 12.4 mmol, 16.0 equiv), followed by ((perfluoroethyl)sulfonyl)phosphorimidoyl trichloride, $P(NSO_2C_2F_5)Cl_3$[2] (0.5 g, 1.6 mmol, 2.1 equiv) were added at 90 °C and the solution was stirred for 15 min. 1,1,1,3,3,3-Hexamethyldisilazane (HMDS, 0.13 g, 0.77 mmol, 1.0 equiv) was added to the reaction mixture, stirred at 90 °C for 10 min, and heated to 130 °C for 3 days.

The reaction mixture was cooled to room temperature, diluted with $CH_2Cl_2$ (15 mL), and stirred with HCl (6.0 M, aq., 3 mL) for 30 min. The organic phase was then separated, dried over $MgSO_4$, filtered, and concentrated under reduced pressure. The residue was purified by column chromatography on silica gel ($R_f$ 0.45, hexanes : EtOAc = 75 : 25) to give a colorless solid, which was then acidified in $CH_2Cl_2$ (15 mL) with HCl (6.0 M, aq., 6 mL) by stirring at room temperature for 30 min. The organic layer was diluted with $CH_2Cl_2$ (30 mL), washed with HCl (6.0 M, aq., 2x20 mL), followed by drying under reduced pressure to provide compound **4h** as a light yellow solid (1.2 g, 0.66 mmol, 85%).

**$^{1}$H NMR** (500 MHz, $CDCl_3$): δ 8.89 (s, 2H, H12b), 8.66 (d, *J* = 8.4 Hz, 2H, H15b), 8.57 (s, 2H, H12a), 8.50 (m, 2H, H15a), 8.26 (d, *J* = 7.9 Hz, 2H, H7b), 8.09 (s, 2H, H4a), 8.07 (d, 8.3 Hz, 2H, H7a), 7.90 (m, 2H, H10b), 7.89 (m, 2H, H8b), 7.88 (m, 2H, H18b), 7.77 (m, 2H, H9b), 7.75 (s, 2H, H4b), 7.68 (m, 2H, H17b), 7.66 (m, 2H, H18a), 7.64 (m, 2H, H20b), 7.63 (m, 2H, H8a), 7.62 (m, 2H, H10a), 7.61 (m, 2H, H16b), 7.47 (3d, *J* = 8.7, 6.9, 1.2 Hz, 2H, H9a), 7.44 (m, 2H, H21b), 7.43 (m, 2H, H20a), 7.42 (m, 2H, H16a), 7.40 (m, 2H, H17a), 7.01 (d, *J* = 8.9 Hz, 2H, H21a), 6.96 (d, *J* = 8.3 Hz, 2H, H23b), 5.98 (dd, *J* = 8.3, 1.7 Hz, 2H, H24b), 5.70 (d, *J* = 8.4 Hz, 2H, H23a), 5.60 (dd, *J* = 8.4, 1.6 Hz, 2H, H24a).

**$^{13}$C NMR** (126 MHz, $CDCl_3$): δ 143.32 (m, ($^{2}J_{CP}$, $^{4}J_{CP}$), AA'X, C2a), 143.31 (m, ($^{2}J_{CP}$, $^{4}J_{CP}$), AA'X, C2b), 134.40 (m, ($^{3}J_{CP}$, $^{5}J_{CP}$), AA'X, C3a), 134.15 (m, ($^{3}J_{CP}$, $^{5}J_{CP}$), AA'X, C3b), 133.58 (C11b), 133.28 (C11a), 132.42 (C19b), 132.30 (C4a), 132.23 (C6b, C5a), 132.16 (C5b), 131.82 (C19a), 131.80 (C4b), 131.77 (C6a), 131.35 (C22b), 131.01 (C22a), 130.38 (C14b), 130.22 (C14a), 130.13 (C13a), 129.93 (C13b), 128.88 (C7b), 128.81 (C7a), 128.61 (C18b), 128.18 (C23b), 128.12 (C18a), 127.95 (C20b), 127.56 (C9b), 127.37 (C23a), 127.32 (C10b), 127.23 (C24b, C24a), 127.18 (C8b), 127.11 (C10a), 126.95 (C9a), 126.84 (C17b), 126.73 (C20a), 126.70 (C16b), 126.58 (C8a), 126.25 (C21b), 126.18 (C16a), 126.12 (C21a, C17a), 124.23 (C12b), 123.85 (m, ($^{3}J_{CP}$, $^{5}J_{CP}$), AA'X, C1b), 123.51 (C12a), 123.42 (C15b), 122.80 (C15a), 122.29 (m, ($^{3}J_{CP}$, $^{5}J_{CP}$), AA'X, C1a), 116.62 (qt 289 Hz ($^{1}J_{CF}$), ~32 Hz ($^{2}J_{CF}$), C26), 110.53 (tq 296 Hz ($^{1}J_{CF}$), 41 Hz ($^{2}J_{CF}$), C25).

**$^{19}$F NMR** (470 MHz, $CDCl_3$): δ –78.90 (s, 6F, F26), –116.36 (d 253 Hz ($^{2}J_{FF}$), AB, 2F, F25a), –116.47 (d 253 Hz ($^{2}J_{FF}$), AB, 2F, F25b).

**$^{31}$P NMR** (202 MHz, $CDCl_3$): δ –15.49.

**HRMS** (ESI–) (*m*/*z*): calculated for $C_{100}H_{56}N_3O_8F_{10}P_2S_2$ $[M–H]^-$: 1742.2830; found 1742.2837.

**$N,N'$-((11b$S$,11b'$S$)-azanediylbis(2,6-di(phenanthren-3-yl)-4$\lambda^5$-dinaphtho[2,1-$d$:1',2'-$f$][1,3,2]dioxaphosphepine-4-yl-4-ylidene))bis(1,1,2,2,3,3,4,4,4-nonafluorobutane-1-sulfonamide) (4i)**

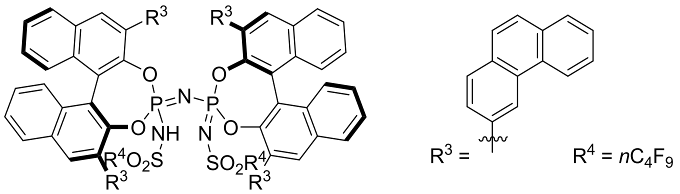


**4i**

In a flame-dried flask under argon, diol **S4** (0.20 g, 0.31 mmol, 2.1 equiv) was dissolved in toluene (2.0 mL, 0.15 M). Subsequently, DIPEA (0.32 g, 2.44 mmol, 16.0 equiv), followed by (($n$-perfluorobutyl)sulfonyl)phosphorimidoyl trichloride, $P(NSO_2(nC_4F_9))Cl_3$ which was prepared by the same protocol with $P(NSO_2C_2F_5)Cl_3$[2] (0.14 g, 0.31 mmol, 2.1 equiv) were added at 90 °C and the solution was stirred for 15 min. HMDS (25 mg, 0.15 mmol, 1.0 equiv) was added to the reaction mixture, stirred at 90 °C for 10 min, and heated to 130 °C for 3 days. The reaction mixture was cooled to room temperature, diluted with $CH_2Cl_2$ (5 mL), and stirred with HCl (6.0 M, aq., 1 mL) for 30 min. The organic phase was then separated, dried with $MgSO_4$, filtered, and concentrated under reduced pressure. The residue was purified by column chromatography on silica gel ($R_f$ 0.47, hexanes : EtOAc = 75 : 25) to give a colorless solid, which was then acidified in $CH_2Cl_2$ (5 mL) with HCl (6.0 M, aq., 2 mL) by stirring at room temperature for 30 min. The organic layer was diluted with $CH_2Cl_2$ (8 mL), washed with HCl (6.0 M, aq., 2x10 mL), followed by drying under reduced pressure to provide compound **4i** as a light yellow solid (135 mg, 0.10 mmol, 68%).

**$^1$H NMR** (500 MHz, $CDCl_3$): δ 8.85 (s, 2H, H12b), 8.63 (d, $J$ = 8.4 Hz, 2H, H15b), 8.55 (d, $J$ = 1.7 Hz, 2H, H12a), 8.49 (d, $J$ = 8.0 Hz, 2H, H15a), 8.22 (d, $J$ = 8.3 Hz, 2H, H7b), 8.12 (s, 2H, H4a), 8.08 (d, $J$ = 8.3 Hz, 2H, H7a), 7.88 (m, 2H, H10b), 7.87 (m, 2H, H8b), 7.81 (dd, $J$ = 8.0, 1.3 Hz, 2H, H18b), 7.75 (ddd, $J$ = 8.5, 6.9, 1.3 Hz, 2H, H9b), 7.72 (s, 2H, H4b), 7.65 (m, 2H, H18a), 7.63 (m, 2H, H8a), 7.61 (m, 2H, H17b), 7.59 (m, 2H, H10a), 7.58 (m, 2H, H20b), 7.54 (m, 2H, H16b), 7.46 (ddd, $J$ = 8.5, 6.9, 1.3 Hz, 2H, H9a), 7.43 (m, 2H, H16a), 7.42 (m, 2H, H21b), 7.41 (m, 2H, H20a), 7.40 (m, 2H, H17a), 7.01 (d, $J$ = 8.8 Hz, 2H, H21a), 6.98 (d, $J$ = 8.3 Hz, 2H, H23b), 6.11 (d, $J$ = 8.3 Hz, 2H, H24b), 5.80 (d, $J$ = 8.4 Hz, 2H, H23a), 5.67 (dd, $J$ = 8.4, 1.7 Hz, 2H, H24a).

**$^{13}$C NMR** (126 MHz, $CDCl_3$): δ 143.42 (m, ($^2J_{CP}$, $^4J_{CP}$), AA'X, C2a), 143.31 (m, ($^2J_{CP}$, $^4J_{CP}$), AA'X, C2b), 134.08 (m, ($^3J_{CP}$, $^5J_{CP}$), AA'X, C3b), 134.07 (m, ($^3J_{CP}$, $^5J_{CP}$), AA'X, C3a), 133.60 (C11b), 133.20 (C11a), 132.33 (C19b), 132.22 (C5a), 132.13 (C5b, C6b), 132.10 (C4a), 131.82 (C19a), 131.77 (C6a), 131.67 (C4b), 131.28 (C22b), 131.03 (C22a), 130.34

(C14b), 130.15 (C14a), 130.02 (C13a), 129.90 (C13b), 128.99 (C7b), 128.74 (C7a), 128.54 (C18b), 128.08 (C23b), 128.06 (C18a), 127.78 (C20b), 127.54 (C9b), 127.38 (C23a), 127.28 (C24b), 127.21 (C24a, C10b), 127.08 (C8b), 127.02 (C10a), 126.91 (C9a), 126.72 (C16b), 126.69 (C17b), 126.55 (C8a, C20a), 126.12 (C21b), 126.08 (C21a), 126.03 (C16a), 126.02 (C17a), 124.04 (C12b), 123.70 (m, ($^3J_{CP}$, $^5J_{CP}$), AA'X, C1b), 123.44 (C12a), 123.21 (C15b), 122.54 (C15a), 122.31 (m, ($^3J_{CP}$, $^5J_{CP}$), AA'X, C1a), 116.73 (s ($^{19}$F-dec), C28), 112.4 (s ($^{19}$F-dec), C25 (or C27)), 109.33 (s ($^{19}$F-dec), C26), 107.6 (s ($^{19}$F-dec), C27 (or C25)).

**$^{19}$F NMR** (470 MHz, $CDCl_3$): δ –81.13 (t, *J* = 10.2 Hz ($^4J_{FF}$), 6F, F28), –112.40 (d, *J* = 259 Hz ($^2J_{FF}$), AB, 2F, F25a), –113.32 (d, *J* = 259 Hz ($^2J_{FF}$), AB, 2F, F25b), –121.45 (q, *J* = 10.2 Hz ($^4J_{FF}$), 4F, F26a, F26b), –126.16 (d, *J* = 193 Hz ($^2J_{FF}$), AB, 2F, F27a), –126.84 (d, *J* = 293 Hz ($^2J_{FF}$), 2F, F27b).

**$^{31}$P NMR** (202 MHz, $CDCl_3$): δ –14.44.

**HRMS** (ESI–) (*m*/*z*): calculated for $C_{104}H_{56}N_3O_8F_{18}P_2S_2$ [M–H]$^-$: 1942.2702; found 1942.2707.

### *Synthesis & Characterization of Substrates*

Aldehydes **1h–1j**[3], **1q**[4] were synthesized according to previously reported procedures.

*Procedure for aldehydes* **1h–1j**

A screw-cap vial was charged with aldehyde (1.0 equiv), dimethylamine hydrochloride (1.2 equiv) and 37 % aq formaldehyde solution (1.2 equiv). Upon heating the heterogeneous mixture under air at 70 °C for 1 h, the formation of a clear, homogenous layer was observed. Prolonged heating at 70°C for 12 to 15 h again resulted in the separation in a biphasic mixture. After allowing the mixture to cool to ambient temperature, water was added and the phases were separated. The aqueous layer was extracted with $CH_2Cl_2$ (× 3), and the combined organic layers were washed with brine (× 1), dried ($Na_2SO_4$) and concentrated under reduced pressure. The crude residue was purified via column chromatography on silica gel (*n*-pentane : $Et_2O$ = 90 : 10) to furnish the desired *α*-methylenated aldehyde.

**2-Methyleneundec-10-enal (1h)**

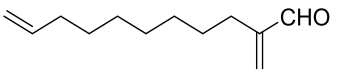


**1h**

**$^1$H NMR** (500 MHz, $CDCl_3$): δ 9.54 (s, 1H), 6.25–6.23 (m, 1H), 5.98 (s, 1H), 5.81 (ddt, *J* = 16.9, 10.1, 6.7 Hz, 1H), 5.05–4.85 (m, 2H), 2.23 (t, *J* = 7.1 Hz, 2H), 2.10–1.98 (m, 2H), 1.48–1.41 (m, 2H), 1.41–1.34 (m, 2H), 1.34–1.26 (q, *J* = 3.5, 2.2 Hz, 6H).

**$^{13}$C NMR** (126 MHz, $CDCl_3$): δ 194.9, 150.6, 139.3, 134.0, 114.3, 33.9, 29.38, 29.37, 29.2, 29.0, 27.91, 27.89.

**HRMS** (ESI+) (*m*/*z*): calculated for $C_{12}H_{20}O_1Na_1$ $[M+Na]^+$: 203.1406 found 203.1407.

**6-((*tert*-butyldimethylsilyl)oxy)-2-methylenehexanal (1i)**

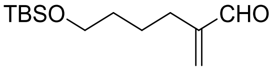


**1i**

**$^{1}$H NMR** (500 MHz, $CDCl_3$): δ 9.54 (s, 1H), 6.30–6.21 (m, 1H), 6.04–5.96 (m, 1H), 3.65–3.58 (m, 2H), 2.32–2.19 (m, 2H), 1.59–1.46 (m, 4H), 0.89 (s, 9H), 0.04 (s, 6H).

**$^{13}$C NMR** (126 MHz, $CDCl_3$): δ 194.7, 150.3, 134.0, 62.8, 32.4, 27.5, 26.0, 24.0, 18.4, –5.3.

**HRMS** (ESI+) (*m*/*z*): calculated for $C_{13}H_{26}O_2Si_1Na_1$ $[M+Na]^+$: 265.1589; found 265.1594.

**2-(2-Fluorobenzyl)acrylaldehyde (1j)**

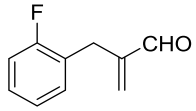


**1j**

**$^{1}$H NMR** (500 MHz, $CDCl_3$): δ 9.61 (s, 1H), 7.25–7.17 (m, 2H), 7.10–7.00 (m, 2H), 6.15–6.03 (m, 2H), 3.60 (s, 2H).

**$^{13}$C NMR** (126 MHz, $CDCl_3$): δ 193.7, 161.1 (d, *J* = 245.8 Hz), 148.1, 135.1, 131.5 (d, *J* = 4.7 Hz), 128.4 (d, *J* = 3.7 Hz), 125.1 (d, *J* = 15.8 Hz), 124.1 (d, *J* = 3.7 Hz), 115.4 (d, *J* = 21.8 Hz), 27.5 (d, *J* = 3.2 Hz).

**$^{19}$F NMR** (470 MHz, $CDCl_3$): δ –117.69.

**HRMS** (EI) (*m*/*z*): calculated for $C_{10}H_9F_1O_1$ [M]: 164.0637; found 164.0633.

**(*E*)-3-(3-vinylphenyl)acrylaldehyde (1q)**

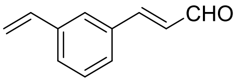


**1q**

**$^{1}$H NMR** (500 MHz, $CDCl_3$): δ 9.72 (d, *J* = 7.7 Hz, 1H), 7.58 (d, *J* = 1.7 Hz, 1H), 7.52–7.45 (m, 3H), 7.40 (t, *J* = 7.6 Hz, 1H), 6.78–6.70 (m, 2H), 5.81 (d, *J* = 17.6 Hz, 1H), 5.34 (d, *J* = 11.1 Hz, 1H).

**$^{13}$C NMR** (126 MHz, $CDCl_3$): δ 193.6, 152.6, 138.5, 135.9, 134.3, 129.3, 128.9, 128.8, 127.7, 126.4, 115.3.

**HRMS** (EI) (*m*/*z*): calculated for $C_{11}H_{10}O_1$ [M]: 158.0732; found 158.0728.

## *Preparation of Stereoisomeric Product Mixtures*

Stereoisomeric cycloadduct mixtures of *α*,*β*-unsaturated aldehydes and cyclopentadiene were prepared by following method A (for **1a**–**1d**, **1g**–**1o**, **1r**, and **1s**) and method B (for **1e**, **1f**, **1p**, and **1t**–**1w**).

**[Method A]**

To a solution of aldehyde **1** (1.0 mmol) in anhydrous toluene (1.0 mL) was added diene (5.0 mmol). The resulting mixture was stirred at 100 °C for 12 h. Purification was performed by column chromatography or preparative thin layer chromatography on silica gel using 5–10% $Et_2O$/*n*-pentane as the eluent.

**[Method B]**

To a solution of aldehyde **1** (1.0 mmol) in anhydrous toluene (1.0 mL) were added diene (5.0 mmol) and $AlCl_3$ (10 mol%). The resulting mixture was stirred at –10 °C for 12 h. Purification was performed by column chromatography or preparative thin layer chromatography on silica gel using 5–10% $Et_2O$/*n*-pentane as the eluent.

## *Establishment of Analytical Assay by Chiral GC*

### *Detailed information of the chiral GC measurement*

· Column name: BGB 176 (2,3-dimethyl-6-tert-butyldimethylsilyl-*β*-cyclodextrin)
· Column length: 25.0 m
· Inner diameter of the column: 0.25mm
· Signal detection: FID
· Gas: $H_2$ (1.00 bar)
· Injector temperature: 220 °C
· Detector temperature: 350 °C
· Column temperature: 85 °C (10 min, iso) to 110 °C (1 °C/min, 20 min iso)
to 144 °C (15 °C/min) to 170 °C (1 °C/min)

**Supplementary Figure 2a.** Collection of GC chromatograms of stereoisomers **3a–3f**.

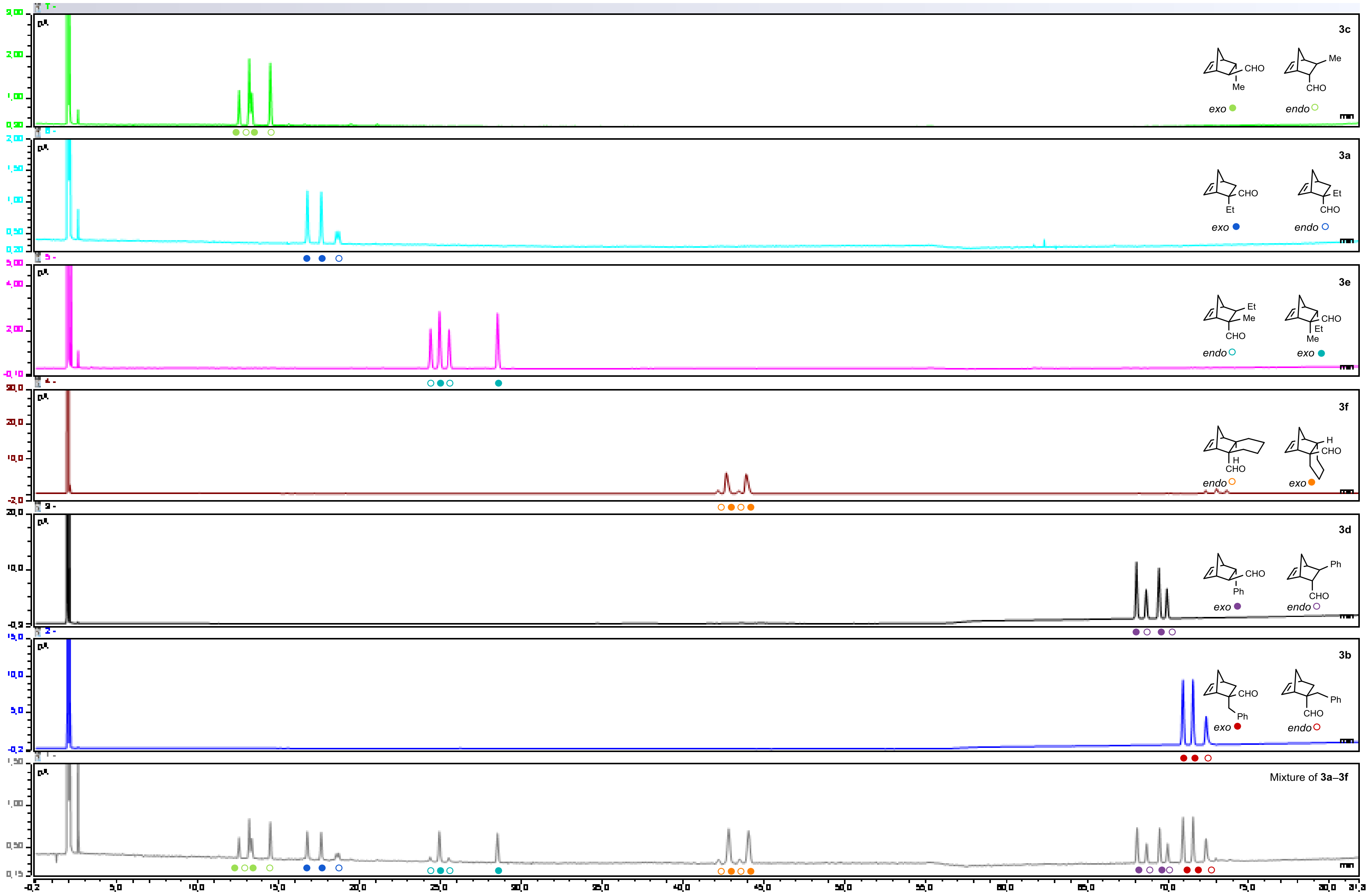

## ***Procedure of the Multi-Substrate Screening***

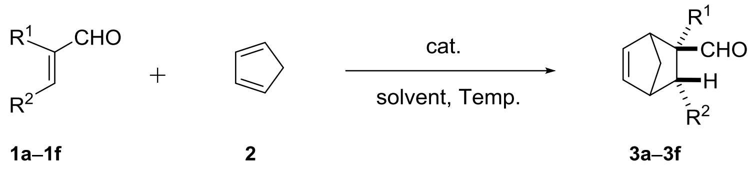


To a vial with a catalyst, immersed in a dry ice-acetone bath at –78 °C, were added a solution of aldehydes (**1a**–**1f** or **1a**–**1d**) and cyclopentadiene **2**. The resulting mixture was stirred at the described temperature for described reaction times. After addition of $NEt_3$ (30 µL), the reaction mixture was warmed to ambient temperature. Conversions were determined by $^1H$ NMR analysis by comparison to *p*-nitrobenzaldehyde as an internal standard. The diastereomeric and enantiomeric ratios were measured by GC analysis on a chiral stationary phase.

## **Supplementary Note 2.** Multi-Substrate Screening for Reaction Optimization

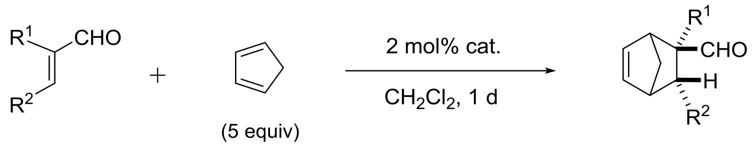


**1a–1f** **2** **3a–3f**

| | cat. | S1 Ph-PA | S2 Ph-DSI | S3 Ph-IDP | 4a Ph-IDPi | 4a Ph-IDPi |
|---|---|---|---|---|---|---|
| | temp. | RT | RT | RT | RT | –78 °C |
| 1a | conv.[b] | 100 | 86 | 100 | 100 | 100 |
| | d.e.[c] | 68 | 62 | 76 | 82 | 90 |
| | e.e.[c] | 5 | 5 | 13 | 57 | 80 |
| 1b | conv.[b] | 100 | 88 | 100 | 100 | 100 |
| | d.e.[c] | 76 | 100 | 84 | 88 | 96 |
| | e.e.[c] | 8 | 4 | 7 | 57 | 79 |
| 1c | conv.[b] | 91 | 62 | 82 | 100 | 98 |
| | d.e.[c] | 64 | 62 | 64 | 40 | 82 |
| | e.e.[c] | 12 | 2 | 1 | 38 | 40 |
| 1d | conv.[b] | 44 | 27 | 48 | 100 | 66 |
| | d.e.[c] | 72 | 0 | 46 | 100 | 100 |
| | e.e.[c] | 10 | 0 | 5 | 31 | 29 |
| 1e | conv.[b] | 56 | 46 | 51 | 91 | 69 |
| | d.e.[c] | 62 | 100 | 80 | 92 | 94 |
| | e.e.[c] | 4 | 6 | 1 | 32 | 40 |
| 1f | conv.[b] | 37 | 35 | 38 | 45 | 51 |
| | d.e.[c] | 100 | 100 | 100 | 100 | 100 |
| | e.e.[c] | 13 | 5 | 2 | 6 | 25 |

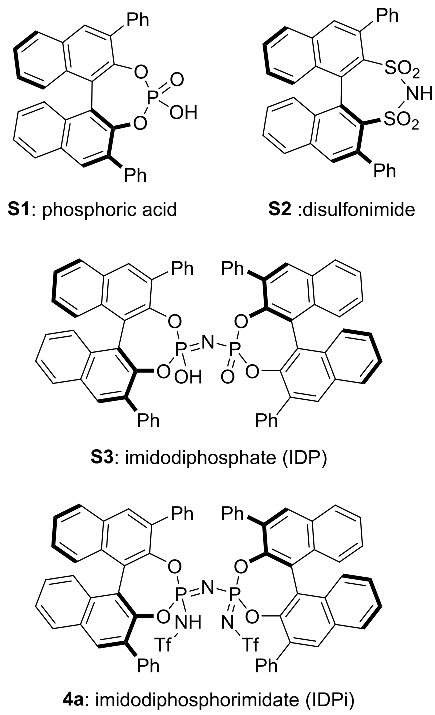


**Supplementary Table 1.** Effects of catalysts: acidity.[a]: a. Unless otherwise indicated, reactions were performed with aldehydes (0.02 mmol each, total 0.12 mmol), diene **2**, and the corresponding catalyst (1.2 μmol) in $CH_2Cl_2$ (120 μL) for 24 h. b. Conversions were determined by $^1H$ NMR analysis by comparison to *p*-nitrobenzaldehyde as an internal standard after addition of TEA. c. The diastereomeric and enantiomeric ratios were measured by GC analysis on a chiral stationary phase.

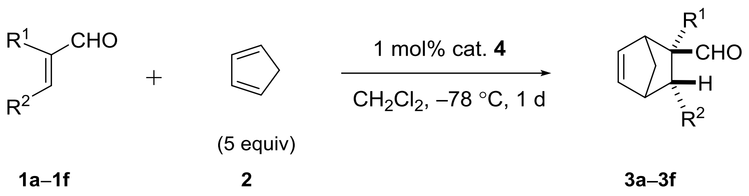


| | | 4a | 4b | 4c | 4d | 4e | 4f | 4g | 4h | 4i |
|---|---|---|---|---|---|---|---|---|---|---|
| | conv.[b] | 100 | 100 | 100 | 93 | 91 | 100 | 100 | 100 | 100 |
| | d.e.[c] | 90 | 72 | 84 | 66 | 66 | 86 | 86 | 94 | 96 |
| **1a** | e.e.[c] | 80 | 62 | 91 | 46 | 38 | 88 | 75 | 86 | 82 |
| | conv.[b] | 100 | 100 | 100 | 82 | 69 | 92 | 100 | 100 | 100 |
| | d.e.[c] | 96 | 56 | 82 | 88 | 90 | 90 | 99 | 100 | 98 |
| **1b** | e.e.[c] | 79 | 36 | 89 | 46 | 15 | 76 | 93 | 95 | 87 |
| | conv.[b] | 98 | 100 | 67 | 93 | 94 | 100 | 100 | 100 | 100 |
| | d.e.[c] | 82 | 82 | 80 | 100 | 88 | 95 | 84 | 86 | 80 |
| **1c** | e.e.[c] | 40 | 73 | 45 | 54 | 61 | 59 | 74 | 84 | 74 |
| | conv.[b] | 66 | 45 | 3 | 39 | 30 | 43 | 30 | 100 | 100 |
| | d.e.[c] | 100 | 96 | 100 | 100 | 96 | 98 | 94 | 96 | 94 |
| **1d** | e.e.[c] | 29 | 30 | 42 | 36 | 44 | 44 | 60 | 81 | 86 |
| | conv.[b] | 69 | 74 | 3 | 35 | 15 | 37 | 43 | 100 | 100 |
| | d.e.[c] | 94 | 90 | 96 | 100 | 100 | 100 | 100 | 100 | 100 |
| **1e** | e.e.[c] | 40 | 24 | 63 | 54 | 39 | 39 | 43 | 54 | 86 |
| | conv.[b] | 51 | 83 | 3 | 21 | 3 | 25 | 39 | 100 | 100 |
| | d.e.[c] | 100 | 100 | 100 | 100 | 100 | 100 | 100 | 100 | 100 |
| **1f** | e.e.[c] | 25 | 36 | 45 | 32 | 21 | 7 | 86 | 89 | 84 |

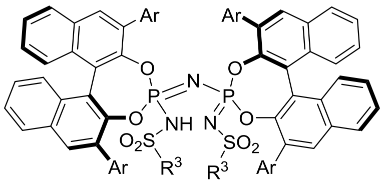


IDPi **4**

| | Ar | $R^3$ |
|---|---|---|
| **4a**: | $C_6H_5$ | $CF_3$ |
| **4b**: | 3,5-$(nC_3F_7)_2$-$C_6H_3$ | $CF_3$ |
| **4c**: | 3,5-$Et_2$-$C_6H_3$ | $CF_3$ |
| **4d**: | 4-Me-$C_6H_4$ | $CF_3$ |
| **4e**: | 4-*t*Bu-$C_6H_4$ | $CF_3$ |
| **4f**: | 2-Naphthyl | $CF_3$ |
| **4g**: | 3-phenanthryl | $CF_3$ |
| **4h**: | 3-phenanthryl | $C_2F_5$ |
| **4i**: | 3-phenanthryl | $nC_4F_9$ |

**Supplementary Table 2.** Effects of catalysts: substituent.[a]: a. Unless otherwise indicated, reactions were performed with aldehydes (**1a–1f**, 0.02 mmol each, total 0.12 mmol), diene **2**, and catalyst **4** (1.2 µmol) in $CH_2Cl_2$ (120 µL) at –78 °C for 24 h. b. Conversions were determined by $^1$H NMR analysis by comparison to *p*-nitrobenzaldehyde as an internal standard after addition of TEA. c. The diastereomeric and enantiomeric ratios were measured by GC analysis on a chiral stationary phase.

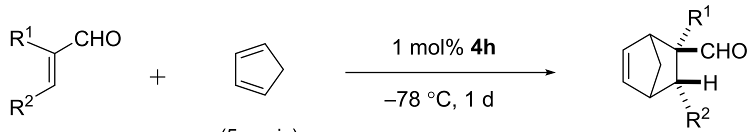


(5 equiv)

**1a–1f** **2** **3a–3f**

| **solvent** | | EtOAc | **DCM** | $CHCl_3$ | $CHCl_3$-$CCl_4$ (1:1) | DCE | $CCl_4$ | Toluene | Ph-Cl | $Et_2O$ | THF | *n*Hex |
|---|---|---|---|---|---|---|---|---|---|---|---|---|
| | conv.[b] | 100 | 100 | 100 | 100 | 100 | 100 | 100 | 100 | 100 | 58 | 100 |
| | d.e.[c] | 90 | 94 | 94 | 96 | 94 | 94 | 96 | 96 | 88 | 88 | 96 |
| **1a** | e.e.[c] | 83 | 86 | 81 | 85 | 81 | 82 | 79 | 82 | 78 | 84 | 84 |
| | conv.[b] | 100 | 100 | 100 | 100 | 100 | 100 | 100 | 100 | 100 | 35 | 100 |
| | d.e.[c] | 98 | 100 | 100 | 100 | 98 | 100 | 100 | 100 | 98 | 96 | 100 |
| **1b** | e.e.[c] | 92 | 95 | 95 | 97 | 91 | 93 | 93 | 93 | 88 | 68 | 92 |
| | conv.[b] | 100 | 100 | 100 | 100 | 100 | 100 | 100 | 100 | 100 | 20 | 100 |
| | d.e.[c] | 88 | 86 | 86 | 84 | 86 | 82 | 86 | 86 | 84 | 84 | 76 |
| **1c** | e.e.[c] | 85 | 84 | 79 | 81 | 82 | 82 | 85 | 82 | 81 | 82 | 76 |
| | conv.[b] | 64 | 100 | 90 | 93 | 90 | 91 | 90 | 85 | 75 | 5 | 73 |
| | d.e.[c] | 96 | 96 | 96 | 94 | 94 | 96 | 96 | 96 | 98 | 100 | 94 |
| **1d** | e.e.[c] | 85 | 81 | 86 | 84 | 83 | 85 | 84 | 85 | 87 | 51 | 81 |
| | conv.[b] | 100 | 100 | 81 | 85 | 97 | 95 | 95 | 86 | 84 | 2 | 83 |
| | d.e.[c] | 100 | 100 | 94 | 100 | 100 | 100 | 96 | 100 | 34 | 100 | 100 |
| **1e** | e.e.[c] | 32 | 54 | 55 | 44 | 55 | 40 | 34 | 49 | 35 | 61 | 67 |
| | conv.[b] | 100 | 100 | 81 | 85 | 96 | 95 | 94 | 89 | 94 | 5 | 88 |
| | d.e.[c] | 100 | 100 | 100 | 100 | 100 | 100 | 100 | 100 | 84 | 100 | 100 |
| **1f** | e.e.[c] | 86 | 89 | 85 | 86 | 85 | 88 | 86 | 88 | 84 | 79 | 90 |

**Supplementary Table 3.** Effects of solvents.[a]: a. Unless otherwise indicated, reactions were performed with aldehydes (0.02 mmol each, total 0.12 mmol), diene **2**, and catalyst **4h** (1.2 µmol) in a solvent (120µL) at –78 °C for 24 h. b. Conversions were determined by $^1$H NMR analysis by comparison to *p*-nitrobenzaldehyde as an internal standard after addition of TEA. c. The diastereomeric and enantiomeric ratios were measured by GC analysis on a chiral stationary phase.

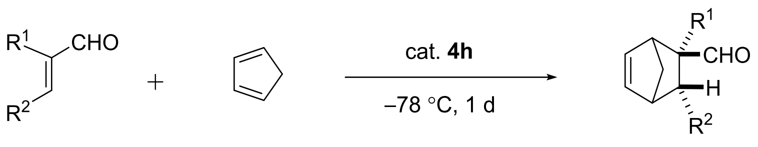


**1a–1f** **2** **3a–3f**

| | 4p mol%<br>2 equiv<br>Conc. | 0.1 mol%<br>5 equiv<br>1.0 M | 0.3 mol%<br>5 equiv<br>1.0 M | 0.5 mol%<br>5 equiv<br>1.0 M | 1 mol%<br>5 equiv<br>1.0 M | 2 mol%<br>5 equiv<br>1.0 M | 3 mol%<br>5 equiv<br>1.0 M | 1 mol%<br>3 equiv<br>1.0 M | 1 mol%<br>10 equiv<br>1.0 M | 1 mol%<br>5 equiv<br>2.0 M | 1 mol%<br>5 equiv<br>No DCM |
|---|---|---|---|---|---|---|---|---|---|---|---|
| | conv.[b] | 29 | 100 | 100 | 100 | 100 | 100 | 100 | 100 | 100 | 100 |
| | d.e.[c] | 92 | 94 | 92 | 94 | 92 | 94 | 94 | 94 | 94 | 92 |
| **1a** | e.e.[c] | 82 | 84 | 86 | 86 | 86 | 86 | 87 | 84 | 84 | 76 |
| | conv.[b] | 16 | 100 | 100 | 100 | 100 | 100 | 100 | 100 | 100 | 100 |
| | d.e.[c] | 98 | 98 | 99 | 99 | 90 | 90 | 90 | 98 | 99 | 98 |
| **1b** | e.e.[c] | 90 | 94 | 93 | 94 | 94 | 94 | 94 | 94 | 92 | 87 |
| | conv.[b] | 44 | 100 | 100 | 100 | 100 | 100 | 100 | 100 | 100 | 100 |
| | d.e.[c] | 88 | 90 | 82 | 82 | 86 | 80 | 84 | 84 | 78 | 86 |
| **1c** | e.e.[c] | 80 | 82 | 82 | 82 | 83 | 81 | 83 | 82 | 81 | 83 |
| | conv.[b] | 10 | 62 | 86 | 100 | 100 | 100 | 100 | 100 | 100 | 100 |
| | d.e.[c] | 100 | 96 | 96 | 96 | 96 | 96 | 96 | 96 | 96 | 98 |
| **1d** | e.e.[c] | 76 | 81 | 80 | 80 | 79 | 80 | 81 | 83 | 81 | 84 |
| | conv.[b] | 11 | 74 | 92 | 100 | 100 | 100 | 100 | 100 | 100 | 100 |
| | d.e.[c] | 100 | 100 | 100 | 100 | 100 | 100 | 100 | 100 | 100 | 100 |
| **1e** | e.e.[c] | 49 | 55 | 54 | 54 | 53 | 55 | 54 | 53 | 55 | 35 |
| | conv.[b] | 5 | 75 | 95 | 100 | 100 | 100 | 100 | 100 | 100 | 100 |
| | d.e.[c] | 100 | 100 | 100 | 100 | 100 | 100 | 100 | 100 | 100 | 100 |
| **1f** | e.e.[c] | 85 | 86 | 88 | 88 | 87 | 88 | 89 | 88 | 89 | 83 |

**Supplementary Table 4.** Effects of catalyst loadings, diene equivalents, concentrations.[a]: a. Unless otherwise indicated, reactions were performed with aldehydes (0.02 mmol each, total 0.12 mmol), diene **2**, and catalyst **4h** in $CH_2Cl_2$at −78 °C for 24 h. b. Conversions were determined by $^1$H NMR analysis by comparison to *p*-nitrobenzaldehyde as an internal standard after addition of TEA. c. The diastereomeric and enantiomeric ratios were measured by GC analysis on a chiral stationary phase.

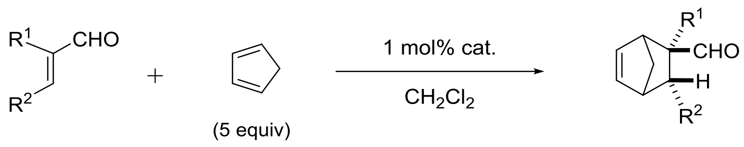


| | | 4h | | 4i | |
|---|---|---|---|---|---|
| | Temp. | –78 °C | –100 °C | –78 °C | –100 °C |
| | Time | 1 d | 2 d | 1 d | 2 d |
| **1a** | conv.[b] | 100 | 100 | 100 | 100 |
| | d.e.[c] | 94 | 94 | 96 | 96 |
| | e.e.[c] | 86 | 92 | 82 | 90 |
| **1b** | conv.[b] | 100 | 100 | 100 | 100 |
| | d.e.[c] | 100 | 99 | 98 | 99 |
| | e.e.[c] | 95 | 98 | 87 | 95 |
| **1c** | conv.[b] | 100 | 100 | 100 | 100 |
| | d.e.[c] | 86 | 92 | 80 | 84 |
| | e.e.[c] | 84 | 90 | 74 | 80 |
| **1d** | conv.[b] | 100 | 100 | 100 | 100 |
| | d.e.[c] | 96 | 98 | 94 | 94 |
| | e.e.[c] | 81 | 86 | 86 | 92 |
| **1e** | conv.[b] | 100 | 100 | 100 | 100 |
| | d.e.[c] | 100 | 100 | 100 | 100 |
| | e.e.[c] | 54 | 88 | 86 | 96 |
| **1f** | conv.[b] | 100 | 100 | 100 | 100 |
| | d.e.[c] | 100 | 100 | 100 | 100 |
| | e.e.[c] | 89 | 96 | 84 | 93 |

**Supplementary Table 5.** Effects of temperatures.[a]: a. Unless otherwise indicated, reactions were performed with aldehydes (0.02 mmol each, total 0.12 mmol), diene **2**, and catalyst **4h** or **4i** (1.2 µmol) in $CH_2Cl_2$ (120µL). b. Conversions were determined by $^1$H NMR analysis by comparison to *p*-nitrobenzaldehyde as an internal standard after addition of TEA. c. The diastereomeric and enantiomeric ratios were measured by GC analysis on a chiral stationary phase.

***General Procedure of the Asymmetric [4+2]-Cycloaddition***

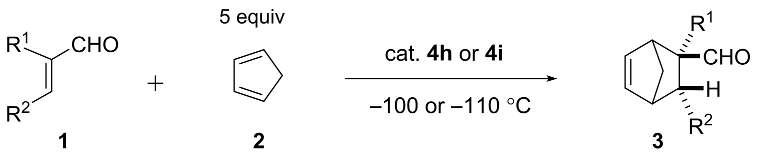


To a solution of the catalyst in anhydrous $CH_2Cl_2$, immersed in a liquid nitrogen-ethanol slush at –116 °C, were added aldehyde **1** (1.0 equiv) and diene **2** (5.0 equiv). The resulting mixture was stirred at the described temperature. Upon completion of the reaction, $NEt_3$ (50 µL) was added and the reaction mixture was warmed to ambient temperature. After removal of the solvent, the crude mixture was dry loaded onto Celite® and purified by column chromatography on silica gel to afford product **3**.

## *Characterization of Products*

### (1*S*,2*R*,4*S*)-2-ethylbicyclo[2.2.1]hept-5-ene-2-carbaldehyde (3a)

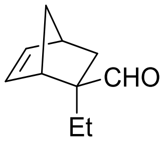


To a solution of catalyst **4h** (1 mol%) in anhydrous $CH_2Cl_2$ (300 µL), immersed in a liquid nitrogen-ethanol slush at –116 °C, were added aldehyde **1a** (25 mg, 0.3 mmol, 1.0 equiv) and diene **2** (99 mg, 1.50 mmol, 5.0 equiv). Then, the resulting reaction mixture was stirred at –100 °C for 2 d. After addition of $NEt_3$ (50 µL) and allowing the mixture to warm to ambient temperature, the mixture was dry loaded onto Celite® and purified by column chromatography on silica gel (*n*-pentane : $Et_2O$ = 95 : 5) to afford **3a** as a colorless oil (42 mg, 0.28 mmol, 92%).

$^1$H NMR and $^{13}$C NMR data for the *exo* isomer were consistent with previously reported values.[5] Diastereoselectivity (*exo*-*endo* ratio) was determined by $^1$H NMR analysis of the crude reaction mixture: 9.70 (s, *exo*), 9.42 (s, *endo*), d.r.(*exo*/*endo*) = 97:3. Absolute configuration was determined by comparison with an authentic sample and from optical rotation.[6]

*exo* isomer

**$^1$H NMR** (500 MHz, $CDCl_3$): δ 9.70 (s, 1H), 6.27 (dd, *J* = 5.7, 3.0 Hz, 1H), 6.08 (dd, *J* = 5.7, 3.1 Hz, 1H), 2.95 (br s, 1H), 2.86 (br s, 1H), 2.14 (dd, *J* = 11.9, 3.9 Hz, 1H), 1.58 (dq, *J* = 14.9, 7.5 Hz, 1H), 1.44 (dd, *J* = 14.2, 7.4 Hz, 1H), 1.39 (ddt, *J* = 8.7, 3.2, 1.9 Hz, 1H), 1.29 (br d, *J* = 8.8 Hz, 1H), 0.82 (dd, *J* = 12.0, 2.8 Hz, 1H), 0.78 (t, *J* = 7.5 Hz, 3H).

**$^{13}$C NMR** (126 MHz, $CDCl_3$): δ 206.4, 139.7, 133.2, 59.5, 47.5, 46.6, 42.6, 32.9, 28.0, 10.1.

**HRMS** (EI) (*m*/*z*): calculated for $C_{10}H_{14}O_1$ [M]: 150.1039; found 150.1041.

$[\boldsymbol{\alpha}]_{\boldsymbol{D}}^{\boldsymbol{25}}$: –58.8 (*c* = 0.35, $CHCl_3$).

**GC**: The enantiomeric ratios were measured by GC analysis on a chiral column (BGB 176 column: 30.0 m; i.D. 0.25mm); FID; Temperature: 220 °C (injector), 220 °C (detector), 85 °C (10 min, iso) to 110 °C (1 °C/min, 20 min iso); Gas: $H_2$ (1.00 bar); $t_R$ = 19.16 min (minor-*exo*), $t_R$ = 19.82 min (major-*exo*), $t_R$ = 21.13 min (major-*endo*), $t_R$ = 21.32 min (minor-*endo*), e.r.(*exo*) = 96:4, e.r.(*endo*) = 75:25.

**(1*S*,2*S*,4*S*)-2-benzylbicyclo[2.2.1]hept-5-ene-2-carbaldehyde (3b)**

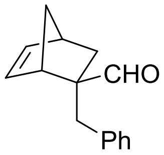


To a solution of catalyst **4h** (1 mol%) in anhydrous $CH_2Cl_2$ (300 µL), immersed in a liquid nitrogen-ethanol slush at –116 °C, were added aldehyde **1b** (29 mg, 0.3 mmol, 1.0 equiv) and diene **2** (99 mg, 1.50 mmol, 5.0 equiv). Then, the resulting reaction mixture was stirred at –100 °C for 2 d. After addition of $NEt_3$ (50 µL) and allowing the mixture to warm to ambient temperature, the mixture was dry loaded onto Celite® and purified by column chromatography on silica gel (*n*-pentane : $Et_2O$ = 95 : 5) to afford **3b** as a colorless oil (59 mg, 0.28 mmol, 92%).

$^1$H NMR and $^{13}$C NMR data for the *exo* isomer were consistent with previously reported values.[7] Diastereoselectivity (*exo-endo* ratio) was determined by $^1$H NMR analysis of the crude reaction mixture: 9.75 (s, *exo*), 9.47 (s, *endo*), d.r.(*exo*/*endo*) = >99:1.

*exo* isomer

**$^1$H NMR** (500 MHz, $CDCl_3$): δ 9.75 (s, 1H), 7.28–7.14 (m, 3H), 7.08–7.03 (m, 2H), 6.39 (dd, *J* = 5.7, 3.0 Hz, 1H), 6.26 (dd, *J* = 5.7, 3.0 Hz, 1H), 3.00–2.94 (m, 2H), 2.89 (br s, 1H), 2.71 (d, *J* = 14.2 Hz, 1H), 2.20 (dd, *J* = 12.1, 3.9 Hz, 1H), 1.42 (ddt, *J* = 8.9, 3.3, 1.8 Hz, 1H), 1.28 (br d, *J* = 9.0 Hz, 1H), 1.01 (dd, *J* = 12.2, 2.8 Hz, 1H).

**$^{13}$C NMR** (126 MHz, $CDCl_3$): δ 206.0, 140.1, 137.9, 133.3, 129.4, 128.4, 126.4, 59.9, 47.5, 47.3, 42.6, 41.6, 33.1.

**HRMS** (ESI+) (*m*/*z*): calculated for $C_{15}H_{16}O_1Na_1$ $[M+Na]^+$: 235.1093; found 235.1094.

$[\alpha]_D^{25}$: –40.2 (*c* = 0.47, $CHCl_3$).

**GC**: The enantiomeric ratio was measured by GC analysis on a chiral column (BGB 176 column: 25.0 m; i.D. 0.25mm); FID; Temperature: 220 °C (injector), 350 °C (detector), 85 °C (10 min, iso) to 110 °C (1 °C/min, 20 min iso) to 144 °C (15 °C/min) to 170 °C (1 °C/min); Gas: $H_2$ (1.00 bar); $t_R$ = 77.56 min (minor-*exo*), $t_R$ = 78.69 min (major-*exo*), e.r.(*exo*) = 99:1.

**(1*S*,2*S*,3*R*,4*R*)-3-methylbicyclo[2.2.1]hept-5-ene-2-carbaldehyde (3c)**

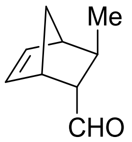


To a solution of catalyst **4h** (1 mol%) in anhydrous $CH_2Cl_2$ (300 µL), immersed in a liquid nitrogen-ethanol slush at –116 °C, were added aldehyde **1c** (21 mg, 0.3 mmol, 1.0 equiv) and diene **2** (99 mg, 1.50 mmol, 5.0 equiv). Then, the resulting reaction mixture was stirred at –100 °C for 3 d. After addition of $NEt_3$ (50 µL) and allowing the mixture to warm to ambient temperature, the mixture was dry loaded onto Celite® and purified by column chromatography on silica gel (*n*-pentane : $Et_2O$ = 95 : 5) to afford **3c** as a colorless oil (38 mg, 0.28 mmol, 93%).

$^1$H NMR and $^{13}$C NMR data for the *endo* isomer were consistent with previously reported values.[8] Diastereoselectivity (*exo*-*endo* ratio) was determined by $^1$H NMR analysis of the crude reaction mixture: 9.79 (d, *exo*), 9.38 (d, *endo*), d.r.(*exo*/*endo*) = 4:96. Absolute configuration was determined by comparison with an authentic sample and from GC analysis.[9,10]

*endo* isomer

**$^1$H NMR** (500 MHz, $CDCl_3$): δ 9.38 (d, *J* = 3.2 Hz, 1H), 6.29 (dd, *J* = 5.7, 3.2 Hz, 1H), 6.05 (dd, *J* = 5.7, 2.8 Hz, 1H), 3.19–3.06 (m, 1H), 2.56 (h, *J* = 1.5 Hz, 1H), 2.33 (dt, *J* = 4.5, 3.3 Hz, 1H), 1.82 (tdd, *J* = 6.9, 5.8, 3.5 Hz, 1H), 1.57 (dt, *J* = 8.9, 1.5 Hz, 1H), 1.48 (dq, *J* = 8.7, 1.7 Hz, 1H), 1.18 (d, *J* = 7.0 Hz, 3H).

**$^{13}$C NMR** (126 MHz, $CDCl_3$): δ 204.9, 138.9, 132.5, 61.3, 49.0, 46.1, 45.4, 36.2, 20.8.

**HRMS** (APCI+) (*m*/*z*): calculated for $C_9H_{13}O_1$ $[M+H]^+$: 137.0961; found 137.0961.

$[\alpha]_D^{25}$: –163 (*c* = 0.27, $CHCl_3$).

**GC**: The enantiomeric ratio was measured by GC analysis on a chiral column (BGB 176 column: 25.0 m; i.D. 0.25mm); FID; Temperature: 220 °C (injector), 350 °C (detector), 85 °C (10 min, iso) to 110 °C (1 °C/min, 20 min iso) ; Gas: $H_2$ (1.00 bar); $t_R$ = 15.00 min (major-*endo*), $t_R$ = 16.91 min (minor-*endo*), e.r.(*endo*) = 95:5.

**(1*S*,2*R*,3*R*,4*R*)-3-phenylbicyclo[2.2.1]hept-5-ene-2-carbaldehyde (3d)**

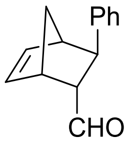


To a solution of catalyst **4i** (1 mol%) in anhydrous $CH_2Cl_2$ (300 µL), immersed in a liquid nitrogen-ethanol slush at –116 °C, were added aldehyde **1d** (25 mg, 0.3 mmol, 1.0 equiv) and diene **2** (99 mg, 1.50 mmol, 5.0 equiv). Then, the resulting reaction mixture was stirred at –100 °C for 4 d. After addition of $NEt_3$ (50 µL) and allowing the mixture to warm to ambient temperature, the mixture was dry loaded onto Celite® and purified by column chromatography on silica gel (*n*-pentane : $Et_2O$ = 90 : 10) to afford **3d** as a colorless oil (56 mg, 0.28 mmol, 95%).

$^1$H NMR and $^{13}$C NMR data for the *endo* isomer were consistent with previously reported values.[8,10] Diastereoselectivity (*exo*-*endo* ratio) was determined by $^1$H NMR analysis of the crude reaction mixture: 9.92 (d, *exo*), 9.60 (d, *endo*), d.r.(*exo*/*endo*) = 3:97. Absolute configuration was determined by comparison with an authentic sample and from GC analysis.[9,10]

*endo* isomer

**$^1$H NMR** (500 MHz, $CDCl_3$): δ 9.60 (d, *J* = 2.2 Hz, 1H), 7.34–7.17 (m, 5H), 6.42 (dd, *J* = 5.7, 3.2 Hz, 1H), 6.17 (dd, *J* = 5.7, 2.8 Hz, 1H), 3.33 (br s, 1H), 3.13 (br s, 1H), 3.09 (dd, *J* = 4.9, 1.7 Hz, 1H), 2.98 (ddd, *J* = 5.3, 3.5, 2.2 Hz, 1H), 1.81 (dt, *J* = 8.7, 1.6 Hz, 1H), 1.62 (dq, *J* = 8.7, 1.9 Hz, 1H).

**$^{13}$C NMR** (126 MHz, $CDCl_3$): δ 203.6, 143.7, 139.4, 133.9, 128.7, 127.5, 126.4, 61.0, 48.5, 47.3, 45.8, 45.3.

**HRMS** (ESI+) (*m*/*z*): calculated for $C_{14}H_{14}O_1Na_1$ $[M+Na]^+$: 221.0937; found 221.0937.

$[\alpha]_D^{25}$: –81.7 (*c* = 0.35, $CHCl_3$).

**GC**: The enantiomeric ratio was measured by GC analysis on a chiral column (BGB 176 column: 25.0 m; i.D. 0.25mm); FID; Temperature: 220 °C (injector), 350 °C (detector), 85 °C (10 min, iso) to 110 °C (1 °C/min, 20 min iso) to 144 °C (15 °C/min) to 170 °C (1 °C/min); Gas: $H_2$ (1.00 bar); $t_R$ = 69.66 min (major-*exo*), $t_R$ = 70.11 min (major-*endo*), 71.24 min (minor-*exo*), $t_R$ = 71.66 min (minor-*endo*), e.r.(*exo*) = 84:16, e.r.(*endo*) = 96:4.

**(1*S*,2*S*,3*S*,4*R*)-3-ethyl-2-methylbicyclo[2.2.1]hept-5-ene-2-carbaldehyde (3e)**

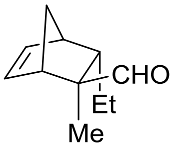


To a solution of catalyst **4i** (1 mol%) in anhydrous $CH_2Cl_2$ (300 µL), immersed in a liquid nitrogen-ethanol slush at –116 °C, were added aldehyde **1e** (29 mg, 0.3 mmol, 1.0 equiv) and diene **2** (99 mg, 1.50 mmol, 5.0 equiv). Then, the resulting reaction mixture was stirred at –100 °C for 4 d. After addition of $NEt_3$ (50 µL) and allowing the mixture to warm to ambient temperature, the mixture was dry loaded onto Celite® and purified by column chromatography on silica gel (*n*-pentane : $Et_2O$ = 95 : 5) to afford **3e** as a colorless oil (46 mg, 0.28 mmol, 93%).

$^1$H NMR and $^{13}$C NMR data for the *exo* isomer were consistent with previously reported values.[11] Diastereoselectivity (*exo*-*endo* ratio) was determined by $^1$H NMR analysis of the crude reaction mixture: 9.64 (s, *exo*), 9.33 (s, *endo*)[11], d.r.(*exo*/*endo*) = 100:0. Absolute configuration was determined by comparison with an authentic sample and from optical rotation.[11]

*exo* isomer

**$^1$H NMR** (500 MHz, $CDCl_3$): δ 9.64 (s, 1H), 6.26 (dd, *J* = 5.7, 3.0 Hz, 1H), 6.18 (dd, *J* = 5.7, 3.0 Hz, 1H), 2.93 (br s, 1H), 2.82 (br s, 1H), 2.27 (ddd, *J* = 10.1, 4.8, 3.3 Hz, 1H), 1.39 (t, *J* = 1.7 Hz, 2H), 1.26–1.12 (m, 1H), 1.00–0.90 (m, 4H), 0.87 (s, 3H).

**$^{13}$C NMR** (126 MHz, $CDCl_3$): δ 206.0, 137.6, 135.3, 56.3, 50.4, 46.12, 46.06, 45.1, 22.8, 15.4, 13.5.

**HRMS** (EI) (*m*/*z*): calculated for $C_{11}H_{16}O_1$ [M]: 164.1196; found 164.1196.

$[\alpha]_D^{25}$: +76.7 (*c* = 0.33, $CHCl_3$).

**GC**: The enantiomeric ratio was measured by GC analysis on a chiral column (BGB 176 column: 25.0 m; i.D. 0.25mm); FID; Temperature: 220 °C (injector), 350 °C (detector), 85 °C (10 min, iso) to 110 °C (1 °C/min, 20 min iso) to 144 °C (15 °C/min); Gas: $H_2$ (1.00 bar); $t_R$ = 27.61 min (major-*exo*), $t_R$ = 31.69 min (minor-*exo*), e.r.(*exo*) = 98:2.

**(1*R*,4*S*,4a*S*,8a*S*)-1,5,6,7,8,8a-hexahydro-1,4-methanonaphthalene-4a(4*H*)-carbaldehyde (3f)**

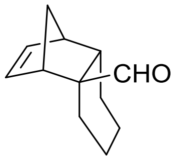


To a solution of catalyst **4h** (1 mol%) in anhydrous $CH_2Cl_2$ (300 µL), immersed in a liquid nitrogen-ethanol slush at –116 °C, were added aldehyde **1f** (25 mg, 0.3 mmol, 1.0 equiv) and diene **2** (99 mg, 1.50 mmol, 5.0 equiv). Then, the resulting reaction mixture was stirred at –100 °C for 4 d. After addition of $NEt_3$ (50 µL) and allowing the mixture to warm to ambient temperature, the mixture was dry loaded onto Celite® and purified by column chromatography on silica gel (*n*-pentane : $Et_2O$ = 90 : 10) to afford **3f** as a colorless oil (49 mg, 0.28 mmol, 93%).

$^1$H NMR and $^{13}$C NMR data for the *endo* isomer were consistent with previously reported values.[11] Diastereoselectivity (*exo*-*endo* ratio) was determined by $^1$H NMR analysis of the crude reaction mixture: 9.64 (s, *exo*), 9.40 (s, *endo*)[11], d.r.(*exo*/*endo*) = 100:0. Absolute configuration was determined by comparison with an authentic sample and from optical rotation.[11]

*exo* isomer

**$^1$H NMR** (500 MHz, $CDCl_3$): δ 9.64 (s, 1H), 6.27 (dd, *J* = 5.7, 3.0 Hz, 1H), 6.17 (dd, *J* = 5.8, 3.2 Hz, 1H), 2.91–2.71 (m, 2H), 2.43 (ddd, *J* = 12.9, 5.4, 3.5 Hz, 1H), 1.78 (ddd, *J* = 13.8, 5.8, 1.5 Hz, 1H), 1.60–1.47 (m, 3H), 1.35 (dt, *J* = 8.6, 1.8 Hz, 1H), 1.29–1.15 (m, 3H), 1.09 (td, *J* = 13.5, 6.3 Hz, 1H), 0.70 (qd, *J* = 13.0, 4.1 Hz, 1H).

**$^{13}$C NMR** (126 MHz, $CDCl_3$): δ 206.3, 138.1, 134.5, 57.5, 48.5, 46.9, 46.0, 39.5, 24.9, 24.3, 19.8, 18.1.

**HRMS** (EI) (*m*/*z*): calculated for $C_{12}H_{16}O_1$ [M]: 176.1196; found 176.1197.

$[\alpha]_D^{25}$: +30.3 (*c* = 0.27, $CHCl_3$).

**GC**: The enantiomeric ratio was measured by GC analysis on a chiral column (BGB 176 column: 25.0 m; i.D. 0.25mm); FID; Temperature: 220 °C (injector), 350 °C (detector), 85 °C (10 min, iso) to 110 °C (1 °C/min, 20 min iso) to 144 °C (15 °C/min); Gas: $H_2$ (1.00 bar); $t_R$ = 47.17 min (major-*exo*), $t_R$ = 49.63 min (minor-*exo*), e.r.(*exo*) = 98:2.

**(1*S*,2*R*,4*S*)-2-methylbicyclo[2.2.1]hept-5-ene-2-carbaldehyde (3g)**

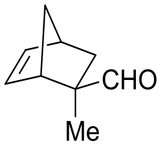


To a solution of catalyst **4h** (1 mol%) in anhydrous $CH_2Cl_2$ (300 µL), immersed in a liquid nitrogen-ethanol slush at –116 °C, were added aldehyde **1g** (21 mg, 0.3 mmol, 1.0 equiv) and diene **2** (99 mg, 1.50 mmol, 5.0 equiv). Then, the resulting reaction mixture was stirred at –100 °C for 3 d. After addition of $NEt_3$ (50 µL) and allowing the mixture to warm to ambient temperature, the mixture was dry loaded onto Celite® and purified by column chromatography on silica gel (*n*-pentane : $Et_2O$ = 95 : 5) to afford **3g** as a colorless oil (35 mg, 0.26 mmol, 86%).

$^1$H NMR and $^{13}$C NMR data for the *exo* isomer were consistent with previously reported values.[5] Diastereoselectivity (*exo*-*endo* ratio) was determined by $^1$H NMR analysis of the crude reaction mixture: 9.69 (s, *exo*), 9.38 (s, *endo*), d.r.(*exo*/*endo*) = 99:1. Absolute configuration was determined by comparison with an authentic sample and from optical rotation.[6]

*exo* isomer

**$^1$H NMR** (500 MHz, $CDCl_3$): δ 9.69 (s, 1H), 6.29 (dd, *J* = 5.6, 3.1 Hz, 1H), 6.11 (dd, *J* = 5.7, 3.1 Hz, 1H), 2.89 (br s, 1H), 2.82 (br s, 1H), 2.25 (dd, *J* = 12.0, 3.8 Hz, 1H), 1.39 (q, *J* = 1.8 Hz, 2H), 1.01 (s, 3H), 0.76 (dt, *J* = 11.9, 1.6 Hz, 1H).

**$^{13}$C NMR** (126 MHz, $CDCl_3$): δ 205.8, 139.6, 133.1, 53.9, 48.5, 47.6, 43.2, 34.6, 20.1.

**HRMS** (EI) (*m*/*z*): calculated for $C_9H_{12}O_1$ [M]: 136.0883; found 136.0885.

$[\alpha]_D^{25}$: –11.7 (*c* = 0.12, $CHCl_3$).

**GC**: The enantiomeric ratios were measured by GC analysis on a chiral column (Hydrodex-gamma-TBDAc column: 25.0 m; i.D. 0.25mm); FID; Temperature: 220 °C (injector), 350 °C (detector), 90 °C (25 min, iso); Gas: $H_2$ (0.80 bar); $t_R$ = 7.46 min (major-*exo*), $t_R$ = 7.97 min (minor-*exo*), $t_R$ = 11.55 min (major-*endo*), $t_R$ = 12.07 min (minor-*endo*), e.r.(*exo*) = 95:5, e.r.(*endo*) = 54:46.

**(((1*S*,2*R*,4*S*)-2-(non-8-en-1-yl)bicyclo[2.2.1]hept-5-en-2-yl)-l3-methyl)-l1-oxidane (3h)**

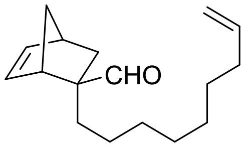


To a solution of catalyst **4h** (1 mol%) in anhydrous $CH_2Cl_2$ (300 µL), immersed in a liquid nitrogen-ethanol slush at –116 °C, were added aldehyde **1h** (37 mg, 0.3 mmol, 1.0 equiv) and diene **2** (99 mg, 1.50 mmol, 5.0 equiv). Then, the resulting reaction mixture was stirred at –100 °C for 3 d. After addition of $NEt_3$ (50 µL) and allowing the mixture to warm to ambient temperature, the mixture was dry loaded onto Celite® and purified by column chromatography on silica gel (*n*-pentane : $Et_2O$ = 95 : 5) to afford **3h** as a colorless oil (66 mg, 0.27 mmol, 90%).

Diastereoselectivity (*exo*-*endo* ratio) was determined by $^1$H NMR analysis of the crude reaction mixture: 9.69 (s, *exo*), 9.43 (s, *endo*), d.r.(*exo*/*endo*) = 98:2. The absolute configuration was assigned by analogy with (1*S*,2*R*,4*S*)-2-ethylbicyclo[2.2.1]hept-5-ene-2-carbaldehyde.

*exo* isomer

**$^1$H NMR** (500 MHz, $CDCl_3$): δ 9.69 (s, 1H), 6.27 (dd, *J* = 5.7, 3.0 Hz, 1H), 6.07 (dd, *J* = 5.7, 3.1 Hz, 1H), 5.80 (ddt, *J* = 17.0, 10.2, 6.7 Hz, 1H), 5.02–4.88 (m, 2H), 2.93 (br s, 1H), 2.85 (br s, 1H), 2.15 (dd, *J* = 11.9, 3.9 Hz, 1H), 2.07–1.95 (m, 2H), 1.54 (ddd, *J* = 13.7, 10.0, 6.7 Hz, 1H), 1.40–1.09 (m, 13H), 0.81 (dd, *J* = 11.9, 2.8 Hz, 1H).

**$^{13}$C NMR** (126 MHz, $CDCl_3$): δ 206.2, 139.6, 139.1, 133.1, 114.2, 58.8, 47.3, 46.9, 42.5, 35.4, 33.7, 33.0, 30.2, 29.2, 29.0, 28.8, 25.7.

**HRMS** (APCI) (*m*/*z*): calculated for $C_{17}H_{27}O_1$ [M+H]$^+$: 247.2056; found 247.2057.

$[\alpha]_D^{25}$: –51.7 (*c* = 0.39, $CHCl_3$).

**GC**: The enantiomeric ratio was measured by GC analysis on a chiral column (BGB 176 column: 25.0 m; i.D. 0.25mm); FID; Temperature: 220 °C (injector), 230 °C (detector), 80 °C (10 min, iso) to 135 °C (20 °C/min, 90 min iso) ; Gas: $H_2$ (1.00 bar); $t_R$ = 88.52 min (minor-*exo*), $t_R$ = 89.86 min (major-*exo*), e.r.(*exo*) = 95:5.

**(4-((1*S*,2*R*,4*S*)-2-((λ¹-oxidanyl)-λ³-methyl)bicyclo[2.2.1]hept-5-en-2-yl)butoxy)(tert-butyl)dimethylsilane (3i)**

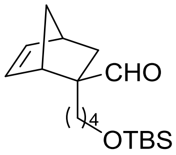


To a solution of catalyst **4h** (1 mol%) and aldehyde **1i** (73 mg, 0.3 mmol, 1.0 equiv) in anhydrous $CH_2Cl_2$ (300 µL), immersed in a liquid nitrogen-ethanol slush at –116 °C, was added diene **2** (99 mg, 1.50 mmol, 5.0 equiv). Then, the resulting reaction mixture was stirred at
–100 °C for 2.5 d. After addition of $NEt_3$ (50 µL) and allowing the mixture to warm to ambient temperature, the mixture was dry loaded onto Celite® and purified by column chromatography on silica gel (0–50% $CH_2Cl_2$ in hexanes) to afford **3i** as a colorless oil (80 mg, 0.26 mmol, 87%).

Diastereoselectivity (*exo*-*endo* ratio) was determined by $^1H$ NMR analysis of the crude reaction mixture: 9.69 (s, *exo*), 9.43 (s, *endo*), d.r.(*exo*/*endo*) = 95:5

*exo* isomer
**$^1$H NMR** (500 MHz, $CDCl_3$): δ 9.69 (s, 1H), 6.27 (dd, *J* = 5.7, 3.0 Hz, 1H), 6.07 (dd, *J* = 5.7, 3.1 Hz, 1H), 3.55 (t, *J* = 6.5 Hz, 2H), 2.93 (h, *J* = 1.6 Hz, 1H), 2.85 (s, 1H), 2.15 (dd, *J* = 11.9, 3.9 Hz, 1H), 1.59–1.52 (m, 1H), 1.47–1.41 (m, 2H), 1.41–1.35 (m, 2H), 1.28 (dt, *J* = 8.7, 1.6 Hz, 1H), 1.23–1.15 (m, 2H), 0.88 (s, 9H), 0.82 (dd, *J* = 12.0, 2.8 Hz, 1H), 0.03 (s, 6H).

**$^{13}$C NMR** (126 MHz, $CDCl_3$): δ 206.1, 139.6, 133.1, 62.7, 58.8, 47.3, 46.9, 42.5, 35.1, 33.3, 33.1, 26.0, 22.0, 18.3, –5.3.

**HRMS** (ESI+) (*m*/*z*): calculated for $C_{18}H_{32}O_2Si_1Na_1$ $[M+Na]^+$: 331.2064; found 331.2064.

$[\alpha]_D^{25}$: –34.4 (*c* = 0.39, $CHCl_3$).

**GC:** The enantiomeric ratios were measured by GC analysis on a chiral column (BGB 176 column: 25.0 m; i.D. 0.25mm); FID; Temperature: 220 °C (injector), 350 °C (detector), 80 °C (10 min, iso) to 145 °C (15 °C/min, 70 min iso); Gas: $H_2$ (1.00 bar); $t_R$ = 67.68 min (minor-*exo*), $t_R$ = 68.86 min (major-*exo*), e.r.(*exo*) = 93:7.

**(1*S*,2*S*,4*S*)-2-(2-fluorobenzyl)bicyclo[2.2.1]hept-5-ene-2-carbaldehyde (3j)**

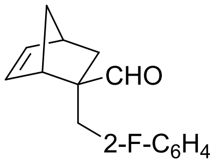


To a solution of catalyst **4h** (1 mol%) in anhydrous $CH_2Cl_2$ (300 µL), immersed in a liquid nitrogen-ethanol slush at –116 °C, were added aldehyde **1j** (49 mg, 0.3 mmol, 1.0 equiv) and diene **2** (99 mg, 1.50 mmol, 5.0 equiv). Then, the resulting reaction mixture was stirred at –100 °C for 2.5 d. After addition of $NEt_3$ (50 µL) and allowing the mixture to warm to ambient temperature, the mixture was dry loaded onto Celite® and purified by column chromatography on silica gel (0–50% $CH_2Cl_2$ in hexanes) to afford **3j** as a colorless oil (64 mg, 0.27 mmol, 93%).

Diastereoselectivity (*exo*-*endo* ratio) was determined by $^1$H NMR analysis of the crude reaction mixture: 9.74 (d, *exo*), 9.58 (d, *endo*), d.r.(*exo*/*endo*) > 99:1.

*exo* isomer

**$^1$H NMR** (500 MHz, $CDCl_3$): 9.74 (d, $J$ = 2.7 Hz, 1H), 7.21–7.14 (m, 1H), 7.09–6.94 (m, 3H), 6.41 (dd, $J$ = 5.7, 3.1 Hz, 1H), 6.26 (dd, $J$ = 5.7, 3.1 Hz, 1H), 3.03–2.96 (m, 2H), 2.89 (br s, 1H), 2.72 (dd, $J$ = 14.3, 1.6 Hz, 1H), 2.23 (dd, $J$ = 12.1, 3.9 Hz, 1H), 1.42 (ddt, $J$ = 8.8, 3.2, 1.8 Hz, 1H), 1.27 (dt, $J$ = 8.9, 1.6 Hz, 1H), 1.00 (dd, $J$ = 12.1, 2.8 Hz, 1H).

**$^{13}$C NMR** (126 MHz, $CDCl_3$): δ 205.0 (d, $J$ = 1.9 Hz), 160.9 (d, $J$ = 244.7 Hz), 140.4, 133.1, 131.6 (d, $J$ = 4.6 Hz), 128.3 (d, $J$ = 8.2 Hz), 124.9 (d, $J$ = 15.9 Hz), 124.0 (d, $J$ = 3.4 Hz), 115.5 (d, $J$ = 22.8 Hz), 59.9, 47.6, 47.4, 42.7, 34.0 (d, $J$ = 1.9 Hz), 32.4.

**$^{19}$F NMR** (470 MHz, $CDCl_3$): δ –115.27.

**HRMS** (EI) (*m*/*z*): calculated for $C_{15}H_{15}F_1O_1$ [M]: 230.1107; found 230.1103.

$[\alpha]_D^{25}$: –58.6 ($c$ = 0.41, $CHCl_3$).

**GC**: The enantiomeric ratios were measured by GC analysis on a chiral column (BGB 176 column: 25.0 m; i.D. 0.25mm) after confirming that there was no minor diastereomer (*endo*) by GC analysis on an achiral column; FID; Temperature: 220 °C (injector), 350 °C (detector), 80 °C (10 min, iso) to 145 °C (15 °C/min, 70 min iso); Gas: $H_2$ (1.00 bar); $t_R$ = 37.42 min (minor-*exo*), $t_R$ = 37.90 min (major-*exo*, no overlapped *endo* isomer), e.r.(*exo*) = 99:1.

**(1*S*,2*S*,3*R*,4*R*)-3-ethylbicyclo[2.2.1]hept-5-ene-2-carbaldehyde (3k)**

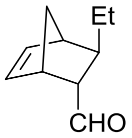


To a solution of catalyst **4h** (1 mol%) in anhydrous $CH_2Cl_2$ (300 µL), immersed in a liquid nitrogen-ethanol slush at –116 °C, were added aldehyde **1k** (25 mg, 0.3 mmol, 1.0 equiv) and diene **2** (99 mg, 1.50 mmol, 5.0 equiv). Then, the resulting reaction mixture was stirred at –100 °C for 3 d. After addition of $NEt_3$ (50 µL) and allowing the mixture to warm to ambient temperature, the mixture was dry loaded onto Celite® and purified by column chromatography on silica gel (*n*-pentane : $Et_2O$ = 95 : 5) to afford **3k** as a colorless oil (40 mg, 0.27 mmol, 90%).

$^{1}H$ NMR and $^{13}C$ NMR data for the *endo* isomer were consistent with previously reported values.[8] Diastereoselectivity (*exo*-*endo* ratio) was determined by $^{1}H$ NMR analysis of the crude reaction mixture: 9.79 (d, *exo*), 9.38 (d, *endo*), d.r.(*exo*/*endo*) = 18:82. The absolute configuration was assigned by analogy with (1*S*,2*S*,3*R*,4*R*)-3-methylbicyclo[2.2.1]hept-5-ene-2-carbaldehyde.

*endo* isomer
**$^{1}H$ NMR** (500 MHz, $CDCl_3$): δ 9.38 (d, *J* = 3.3 Hz, 1H), 6.27 (dd, *J* = 5.7, 3.2 Hz, 1H), 6.07 (dd, *J* = 5.7, 2.8 Hz, 1H), 3.12 (br s, 1H), 2.69 (br s, 1H), 2.38 (dt, *J* = 4.5, 3.4 Hz, 1H), 1.63–1.57 (m, 1H), 1.55–1.43 (m, 4H), 0.96 (t, *J* = 7.3 Hz, 3H).

**$^{13}C$ NMR** (126 MHz, $CDCl_3$): δ 205.1, 138.8, 132.9, 59.9, 46.9, 46.5, 45.0, 44.1, 28.6, 12.9.

**HRMS** (EI) (*m*/*z*): calculated for $C_{10}H_{14}O_1$ [M]: 150.1039; found 150.1039.

$[\alpha]_D^{25}$: –75.6 (*c* = 0.70, $CHCl_3$).

**GC**: The enantiomeric ratios were measured by GC analysis on a chiral column (Hydrodex-gamma-TBDAc column: 25.0 m; i.D. 0.25mm); FID; Temperature: 220 °C (injector), 350 °C (detector), 60 °C (140 min, iso); Gas: $H_2$ (0.50 bar); $t_R$ = 78.84 min (major-*exo*), $t_R$ = 81.11 min (minor-*exo*), $t_R$ = 108.60 min (minor-*endo*), $t_R$ = 127.27 min (major-*endo*), e.r.(*exo*) = 86:14, e.r.(*endo*) = 93:7.

**(1*S*,2*S*,3*R*,4*R*)-3-propylbicyclo[2.2.1]hept-5-ene-2-carbaldehyde (3l)**

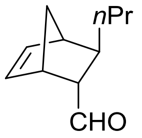


To a solution of catalyst **4h** (1 mol%) in anhydrous $CH_2Cl_2$ (300 µL), immersed in a liquid nitrogen-ethanol slush at –116 °C, were added aldehyde **1l** (29 mg, 0.3 mmol, 1.0 equiv) and diene **2** (99 mg, 1.50 mmol, 5.0 equiv). Then, the resulting reaction mixture was stirred at –100 °C for 3 d. After addition of $NEt_3$ (50 µL) and allowing the mixture to warm to ambient temperature, the mixture was dry loaded onto Celite® and purified by column chromatography on silica gel (*n*-pentane : $Et_2O$ = 95 : 5) to afford **3l** as a colorless oil (45 mg, 0.27 mmol, 91%).

$^1$H NMR and $^{13}$C NMR data for the *endo* isomer were consistent with previously reported values.[8–10] Diastereoselectivity (*exo*-*endo* ratio) was determined by $^1$H NMR analysis of the crude reaction mixture: 9.78 (d, *exo*), 9.37 (d, *endo*), d.r.(*exo*/*endo*) = 19:81. Absolute configuration was determined by comparison with an authentic sample and from optical rotation.[9]

*exo* isomer

**$^1$H NMR** (500 MHz, $CDCl_3$): δ 9.78 (d, *J* = 2.7 Hz, 1H), 6.21 (dd, *J* = 5.7, 3.1 Hz, 1H), 6.13 (dd, *J* = 5.7, 2.9 Hz, 1H), 3.02 (br s, 1H), 2.87 (br s, 1H), 2.29 (tdd, *J* = 7.9, 4.9, 3.4 Hz, 1H), 1.76 (ddd, *J* = 4.7, 2.7, 1.7 Hz, 1H), 1.50–1.07 (m, 6H), 0.87 (t, *J* = 7.3 Hz, 3H).

**$^{13}$C NMR** (126 MHz, $CDCl_3$): δ 204.0, 136.1, 135.9, 58.8, 47.0, 45.7, 44.9, 41.6, 36.4, 21.6, 14.2.

*endo* isomer

**$^1$H NMR** (500 MHz, $CDCl_3$): δ 9.37 (d, *J* = 3.3 Hz, 1H), 6.28 (dd, *J* = 5.7, 3.2 Hz, 1H), 6.06 (dd, *J* = 5.7, 2.8 Hz, 1H), 3.12 (bs, 1H), 2.66 (bs, 1H), 2.38 (dt, *J* = 4.5, 3.4 Hz, 1H), 1.69 (dddd, *J* = 8.2, 6.3, 4.5, 1.6 Hz, 1H), 1.55–1.32 (m, 6H), 0.91 (t, *J* = 7.1 Hz, 3H).

**$^{13}$C NMR** (126 MHz, $CDCl_3$): δ 205.1, 138.8, 132.8, 60.0, 47.2, 46.5, 45.1, 41.9, 38.0, 21.6, 14.2.

**HRMS** (EI) (*m*/*z*): calculated for $C_{11}H_{16}O_1$ [M]: 164.1196; found 164.1196.

$[\alpha]_D^{25}$: –66.3 (*c* = 0.55, $CHCl_3$).

**GC**: The enantiomeric ratios were measured by GC analysis on a chiral column (BGB 176 column: 25.0 m; i.D. 0.25mm); FID; Temperature: 220 °C (injector), 350 °C (detector), 105 °C (40 min, iso); Gas: $H_2$ (0.50 bar); $t_R$ = 26.72 min (major-*exo*), $t_R$ = 27.62 min (major-*endo*), 28.24 min (minor-*exo*), $t_R$ = 30.48 min (minor-*endo*), e.r.(*exo*) = 86:14, e.r.(*endo*) = 94:6.

**(1*S*,2*S*,3*R*,4*R*)-3-heptylbicyclo[2.2.1]hept-5-ene-2-carbaldehyde (3m)**

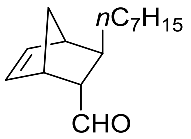


To a solution of catalyst **4h** (1 mol%) in anhydrous $CH_2Cl_2$ (300 µL), immersed in a liquid nitrogen-ethanol slush at –116 °C, were added aldehyde **1m** (46 mg, 0.3 mmol, 1.0 equiv) and diene **2** (99 mg, 1.50 mmol, 5.0 equiv). Then, the resulting reaction mixture was stirred at –100 °C for 3 d. After addition of $NEt_3$ (50 µL) and allowing the mixture to warm to ambient temperature, the mixture was dry loaded onto Celite® and purified by column chromatography on silica gel (*n*-pentane : $Et_2O$ = 95 : 5) to afford **3m** as a colorless oil (64 mg, 0.29 mmol, 96%).

Diastereoselectivity (*exo*-*endo* ratio) was determined by $^1$H NMR analysis of the crude reaction mixture: 9.78 (d, *exo*), 9.37 (d, *endo*), d.r.(*exo*/*endo*) = 19:81.

*exo* isomer

**$^1$H NMR** (500 MHz, $CDCl_3$): δ 9.78 (d, *J* = 2.7 Hz, 1H), 6.21 (dd, *J* = 5.7, 3.1 Hz, 1H), 6.13 (dd, *J* = 5.7, 2.9 Hz, 1H), 3.01 (br s, 1H), 2.87 (br s, 1H), 2.26 (tdd, *J* = 7.9, 4.9, 3.4 Hz, 1H), 1.75 (dt, *J* = 4.6, 2.1 Hz, 1H), 1.51–1.07 (m, 14H), 0.86 (t, *J* = 6.8 Hz, 3H).

**$^{13}$C NMR** (126 MHz, $CDCl_3$): δ 204.0, 136.1, 135.9, 58.8, 47.0, 45.7, 44.9, 41.9, 34.2, 29.7, 29.2, 28.5, 28.4, 22.7, 14.1.

*endo* isomer

**$^1$H NMR** (500 MHz, $CDCl_3$): δ 9.37 (d, *J* = 3.3 Hz, 1H), 6.27 (dd, *J* = 5.7, 3.2 Hz, 1H), 6.06 (dd, *J* = 5.7, 2.8 Hz, 1H), 3.11 (bs, 1H), 2.66 (bs, 1H), 2.37 (dt, *J* = 4.5, 3.4 Hz, 1H), 1.67 (dddd, *J* = 8.3, 6.2, 4.4, 1.5 Hz, 1H), 1.56–1.19 (m, 14H), 0.88 (t, *J* = 6.8 Hz, 3H).

**$^{13}$C NMR** (126 MHz, $CDCl_3$): δ 205.1, 138.8, 132.8, 60.1, 47.2, 46.5, 45.1, 42.2, 35.8, 31.8, 29.7, 29.2, 28.4, 22.7, 14.1.

**HRMS** (ACPI+) (*m*/*z*): calculated for $C_{15}H_{25}O_1$ $[M+H]^+$: 221.1990; found 221.1900.

$[\alpha]_D^{25}$: –53.0 (*c* = 0.45, $CHCl_3$).

**GC**: The enantiomeric ratios were measured by GC analysis on a chiral column (BGB 176 column: 25.0 m; i.D. 0.25mm); FID; Temperature: 220 °C (injector), 350 °C (detector), 85 °C (5 min, iso) to 144 °C (20 °C/min) to 210 °C (2 °C/min); Gas: $H_2$ (0.70 bar); $t_R$ = 22.44 min (major-*exo*), $t_R$ = 22.80 min (minor-*exo*), 22.97 min (major-*endo*), $t_R$ = 23.52 min (minor-*endo*), e.r.(*exo*) = 86:14, e.r.(*endo*) = 95:5.

**(1*S*,2*R*,3*R*,4*R*)-3-isobutylbicyclo[2.2.1]hept-5-ene-2-carbaldehyde (3n)**

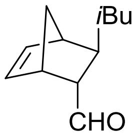


To a solution of catalyst **4h** (1 mol%) in anhydrous $CH_2Cl_2$ (300 µL), immersed in a liquid nitrogen-ethanol slush at –116 °C, were added aldehyde **1n** (25 mg, 0.3 mmol, 1.0 equiv) and diene **2** (99 mg, 1.50 mmol, 5.0 equiv). Then, the resulting reaction mixture was stirred at –100 °C for 4 d. After addition of $NEt_3$ (50 µL) and allowing the mixture to warm to ambient temperature, the mixture was dry loaded onto Celite® and purified by column chromatography on silica gel (*n*-pentane : $Et_2O$ = 95 : 5) to afford **3n** as a colorless oil (50 mg, 0.28 mmol, 93%).

Diastereoselectivity (*exo*-*endo* ratio) was determined by $^1$H-NMR analysis of the crude reaction mixture: 9.79 (d, *exo*), 9.37 (d, *endo*), d.r.(*exo*/*endo*) = 25:75.

*exo* isomer

**$^1$H NMR** (500 MHz, $CDCl_3$): δ 9.79 (d, *J* = 2.8 Hz, 1H), 6.21 (dd, *J* = 5.7, 3.1 Hz, 1H), 6.13 (dd, *J* = 5.6, 3.0 Hz, 1H), 2.43–2.37 (m, 2H), 1.75 (dd, *J* = 4.7, 3.1 Hz, 1H), 1.54–1.51 (m, 1H), 1.44 (q, *J* = 1.5 Hz, 2H), 1.34 (t, *J* = 7.3 Hz, 1H), 1.09 (dt, *J* = 13.6, 7.3 Hz, 1H), 1.00 (ddd, *J* = 13.5, 7.9, 7.1 Hz, 1H), 0.88 (d, *J* = 6.6 Hz, 3H), 0.87 (d, *J* = 6.6 Hz, 3H).

$^{13}$**C NMR** (126 MHz, $CDCl_3$): δ 204.0, 136.04, 136.00, 58.9, 47.0, 45.9, 45.0, 43.5, 39.5, 26.7, 22.83, 22.77.

*endo* isomer

$^{1}$**H NMR** (500 MHz, $CDCl_3$): δ 9.37 (d, *J* = 3.4 Hz, 1H), 6.29 (dd, *J* = 5.8, 3.2 Hz, 1H), 6.06 (dd, *J* = 5.7, 2.8 Hz, 1H), 3.12 (bs, 1H), 2.63 (bs, 1H), 2.38 (dt, *J* = 4.4, 3.4 Hz, 1H), 1.80 (tdd, *J* = 7.6, 4.4, 1.5 Hz, 1H), 1.63 (dp, *J* = 13.4, 6.7 Hz, 1H), 1.57–1.52 (m, 1H), 1.49–1.45 (m, 1H), 1.34 (t, *J* = 7.3 Hz, 2H), 0.90 (d, *J* = 6.6 Hz, 3H), 0.88 (d, *J* = 6.5 Hz, 3H).

$^{13}$**C NMR** (126 MHz, $CDCl_3$): δ 205.1, 138.9, 132.8, 60.1, 47.5, 46.5, 45.3, 45.1, 39.7, 26.6, 23.2, 22.5.

**HRMS** (EI) (*m/z*): calculated for $C_{12}H_{18}O_1$ [M]: 178.1352; found 178.1353.

$[\alpha]_D^{25}$: –59.1 (*c* = 0.34, $CHCl_3$).

**GC**: The enantiomeric ratios were measured by GC analysis on a chiral column (BGB 178 column: 25.0 m; i.D. 0.25mm); FID; Temperature: 220 °C (injector), 350 °C (detector), 105 °C (40 min, iso); Gas: $H_2$ (0.60 bar); $t_R$ = 18.90 min (major-*exo*), $t_R$ = 19.77 min (minor-*exo*), 20.26 min (major-*endo*), $t_R$ = 21.85 min (minor-*endo*), e.r.(*exo*) = 88:12, e.r.(*endo*) = 96:4.

**Ethyl (1*R*,2*R*,3*R*,4*S*)-3-formylbicyclo[2.2.1]hept-5-ene-2-carboxylate (3o)**

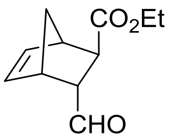


To a solution of catalyst **4h** (1 mol%) in anhydrous $CH_2Cl_2$ (300 µL), immersed in a liquid nitrogen-ethanol slush at –116 °C, were added aldehyde **1o** (38 mg, 0.3 mmol, 1.0 equiv) and diene **2** (99 mg, 1.50 mmol, 5.0 equiv). Then, the resulting reaction mixture was stirred at –100 °C for 3 d. After addition of $NEt_3$ (50 µL) and allowing the mixture to warm to ambient temperature, the mixture was dry loaded onto Celite® and purified by column chromatography on silica gel (*n*-pentane : $Et_2O$ = 80 : 20) to afford **3o** as a colorless oil (52 mg, 0.27 mmol, 90%).

$^{1}H$ NMR and $^{13}C$ NMR data for the *endo* isomer were consistent with previously reported values.[8,10] Diastereoselectivity (*exo-endo* ratio) was determined by $^{1}H$-NMR analysis of the crude reaction mixture: 9.85 (d, *exo*), 9.55 (d, *endo*), d.r.(*exo*/*endo*) = 7:93. Absolute configuration was determined by comparison with an authentic sample and from optical rotation.[8]

*exo* isomer

**$^{1}$H NMR** (500 MHz, $CDCl_3$): δ 9.85 (d, *J* = 0.9 Hz, 1H), 6.30 (dd, *J* = 5.7, 3.2 Hz, 1H), 6.13 (dd, *J* = 5.6, 2.8 Hz, 1H), 4.10 (qd, *J* = 7.1, 4.5 Hz, 2H), 3.41 (t, *J* = 4.1 Hz, 1H), 3.31–3.27 (m, 1H), 3.21 (d, *J* = 2.0 Hz, 1H), 2.84–2.79 (m, 1H), 1.47–1.42 (m, 1H), 1.34 (br d, *J* = 9.0 Hz, 1H), 1.24 (t, *J* = 7.1 Hz, 3H).

**$^{13}$C NMR** (126 MHz, $CDCl_3$): δ 201.0, 173.0, 137.0, 135.8, 60.7, 55.9, 46.7, 45.5, 44.5, 44.4, 14.2.

*endo* isomer

**$^{1}$H NMR** (500 MHz, $CDCl_3$): δ 9.55 (d, *J* = 1.2 Hz, 1H), 6.26 (dd, *J* = 5.7, 3.2 Hz, 1H), 6.09 (dd, *J* = 5.7, 2.7 Hz, 1H), 4.17 (q, *J* = 7.2 Hz, 2H), 3.42–3.30 (m, 2H), 3.24–3.15 (m, 1H), 2.70 (dd, *J* = 4.4, 1.7 Hz, 1H), 1.68 (bd, *J* = 8.9 Hz, 1H), 1.51 (dq, *J* = 8.7, 1.7 Hz, 1H), 1.28 (t, *J* = 7.2 Hz, 3H).

**$^{13}$C NMR** (126 MHz, $CDCl_3$): δ 201.4, 174.0, 137.7, 134.4, 61.0, 56.8, 47.7, 47.3, 44.8, 44.3, 14.2.

**HRMS** (ESI+) (*m*/*z*): calculated for $C_{11}H_{14}O_3Na_1$ $[M+Na]^+$: 217.0835; found 217.0834.

$[\alpha]_D^{25}$: –55.6 (*c* = 0.66, $CHCl_3$).

**GC**: The enantiomeric ratios were measured by GC analysis on a chiral column (Ivadex-1 column: 25.0 m; i.D. 0.25mm); FID; Temperature: 220 °C (injector), 350 °C (detector), 90 °C (100 min, iso); Gas: $H_2$ (0.60 bar); $t_R$ = 73.40 min (major-*endo*), $t_R$ = 84.32 min (major-*exo*), 86.27 min (minor-*endo*), $t_R$ = 89.37 min (minor-*exo*), e.r.(*exo*) = 66:34, e.r.(*endo*) = 91:9.

**(1*S*,2*R*,3*R*,4*R*)-3-(4-methoxyphenyl)bicyclo[2.2.1]hept-5-ene-2-carbaldehyde (3p)**

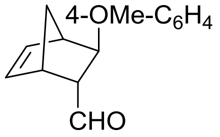


To a solution of catalyst **4i** (1 mol%) and aldehyde **1p** (49 mg, 0.3 mmol, 1.0 equiv) in anhydrous $CH_2Cl_2$ (300 µL), immersed in a liquid nitrogen-ethanol slush at –116 °C, was added diene **2** (99 mg, 1.50 mmol, 5.0 equiv). Then, the resulting reaction mixture was stirred at
–110 °C for 5 d. After addition of $NEt_3$ (50 µL) and allowing the mixture to warm to ambient temperature, the mixture was dry loaded onto Celite® and purified by column chromatography (*n*-pentane : $Et_2O$ = 80 : 20) to afford **3p** as a white solid (60 mg, 0.26 mmol, 87%).

$^1$H NMR and $^{13}$C NMR data for the *endo* isomer were consistent with previously reported values.[12] Diastereoselectivity (*exo*-*endo* ratio) was determined by $^1$H NMR analysis of the crude reaction mixture: 9.91 (d, *exo*), 9.58 (d, *endo*), d.r.(*exo*/*endo*) = 1:99.

*endo* isomer
**$^1$H NMR** (500 MHz, $CDCl_3$): δ 9.58 (d, *J* = 2.4 Hz, 1H), 7.18 (d, *J* = 8.5 Hz, 2H), 6.85 (d, *J* = 8.5 Hz, 2H), 6.41 (dd, *J* = 5.7, 3.2 Hz, 1H), 6.16 (dd, *J* = 5.6, 2.8 Hz, 1H), 3.79 (s, 3H), 3.31 (br s, 1H), 3.07 (br s, 1H), 3.02 (dd, *J* = 5.3, 1.4 Hz, 1H), 2.93 (ddd, *J* = 5.0, 3.5, 2.4 Hz, 1H), 1.79 (dt, *J* = 8.7, 1.5 Hz, 1H), 1.61 (dq, *J* = 8.7, 1.8 Hz, 1H).

**$^{13}$C NMR** (126 MHz, $CDCl_3$): δ 203.9, 158.1, 139.4, 135.7, 133.8, 128.4, 114.1, 61.1, 55.5, 48.8, 47.2, 45.3, 45.2.

**HRMS** (ESI+) (*m*/*z*): calculated for $C_{15}H_{16}O_2Na_1$ $[M+Na]^+$: 251.1042; found 251.1042.

$[\boldsymbol{\alpha}]_{\boldsymbol{D}}^{\mathbf{25}}$: –28.9 (*c* = 0.41, $CHCl_3$).

**HPLC**: The enantiomeric ratio was measured by HPLC analysis on a chiral column (OJ-3 column: i.D. 4.6 mm) after reduction with $NaBH_4$/MeOH. Heptane/ *i*PrOH = 70:30, flow rate = 1.0 mL/min, λ = 230 nm, $t_R$ = 5.47 min (major-*endo*) and $t_R$= 15.77 min (minor-*endo*), e.r.(*endo*) = 96:4.

**(1*S*,2*R*,4*R*)-3-(3-vinylphenyl)bicyclo[2.2.1]hept-5-ene-2-carbaldehyde (3q)**

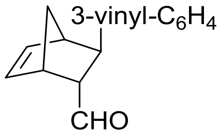


To a solution of catalyst **4i** (1 mol%) and aldehyde **1q** (47 mg, 0.3 mmol, 1.0 equiv) in anhydrous $CH_2Cl_2$ (300 µL), immersed in a liquid nitrogen-ethanol slush at –116 °C, was added diene **2** (99 mg, 1.50 mmol, 5.0 equiv). Then, the resulting reaction mixture was stirred at

–100 °C for 3 d. After addition of $NEt_3$ (50 µL) and allowing the mixture to warm to ambient temperature, the mixture was dry loaded onto Celite® and purified by column chromatography on silica gel (0–60% $CH_2Cl_2$ in hexanes) to afford **3q** as a colorless oil (56 mg, 0.25 mmol, 84%).

Diastereoselectivity (*exo*-*endo* ratio) was determined by $^1$H NMR analysis of the crude reaction mixture: 9.93 (d, *exo*), 9.61 (d, *endo*), d.r.(*exo*/*endo*) = 1:99

*endo* isomer

**$^1$H NMR** (500 MHz, $CDCl_3$): δ 9.61 (d, *J* = 2.1 Hz, 1H), 7.30–7.26 (m, 3H), 7.16 (td, *J* = 4.6, 2.2 Hz, 1H), 6.70 (dd, *J* = 17.6, 10.9 Hz, 1H), 6.42 (dd, *J* = 5.7, 3.2 Hz, 1H), 6.17 (dd, *J* = 5.7, 2.7 Hz, 1H), 5.74 (dd, *J* = 17.6, 0.9 Hz, 1H), 5.25 (dd, *J* = 10.9, 0.9 Hz, 1H), 3.37–3.33 (m, 1H), 3.13 (dq, *J* = 3.4, 1.6 Hz, 1H), 3.09 (dd, *J* = 5.1, 1.6 Hz, 1H), 2.99 (ddd, *J* = 5.2, 3.5, 2.2 Hz, 1H), 1.82 (dt, *J* = 8.6, 1.6 Hz, 1H), 1.63 (dq, *J* = 8.8, 1.8 Hz, 1H).

**$^{13}$C NMR** (126 MHz, $CDCl_3$): δ 203.4, 143.8, 139.2, 137.9, 136.8, 133.8, 128.8, 126.8, 125.5, 124.0, 114.1, 60.8, 48.4, 47.2, 45.6, 45.1.

**HRMS** (EI) (*m*/*z*): calculated for $C_{16}H_{16}O_1$ [M]: 224.1201; found 224.1196.

$[\alpha]_D^{25}$: –55.3 (*c* = 0.35, $CHCl_3$).

**GC:** The enantiomeric ratios were measured by GC analysis on a chiral column (BGB 176 column: 25.0 m; i.D. 0.25mm); FID; Temperature: 220 °C (injector), 350 °C (detector), 80 °C (15 min, iso) to 150 °C (20 °C/min, 40 min iso) to 170 °C (1 °C/min); Gas: $H_2$ (1.00 bar); $t_R$ = 63.67 min (major-*endo*), $t_R$ = 66.94 min (minor-*endo*), e.r.(*endo*) = 94:6.

**(1*S*,2*R*,3*R*,4*R*)-3-(4-bromophenyl)bicyclo[2.2.1]hept-5-ene-2-carbaldehyde (3r)**

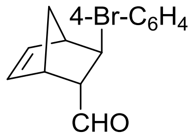


To a solution of catalyst **4i** (1 mol%) and aldehyde **1r** (63 mg, 0.3 mmol, 1.0 equiv) in anhydrous $CH_2Cl_2$ (300 µL), immersed in a liquid nitrogen-ethanol slush at –116 °C, was added diene **2** (99 mg, 1.50 mmol, 5.0 equiv). Then, the resulting reaction mixture was stirred at

–110 °C for 5 d. After addition of $NEt_3$ (50 µL) and allowing the mixture to warm to ambient temperature, the mixture was dry loaded onto Celite® and purified by column chromatography on silica gel (*n*-pentane : $Et_2O$ = 85 : 15) to afford **3r** as a white solid (68 mg, 0.25 mmol, 82%).

$^1$H NMR and $^{13}$C NMR data for the *endo* isomer were consistent with previously reported values. [10,11] Diastereoselectivity (*exo*-*endo* ratio) was determined by $^1$H NMR analysis of the crude reaction mixture: 9.90 (d, *exo*), 9.59 (d, *endo*), d.r.(*exo*/*endo*) = 1:99.

*endo* isomer

**$^1$H NMR** (500 MHz, $CDCl_3$): δ 9.59 (d, *J* = 2.1 Hz, 1H), 7.42 (d, *J* = 8.5 Hz, 2H), 7.14 (d, *J* = 8.5 Hz, 2H), 6.41 (dd, *J* = 5.7, 3.3 Hz, 1H), 6.17 (dd, *J* = 5.7, 2.8 Hz, 1H), 3.35 (br s, *J* = 2.0 Hz, 1H), 3.09 (br s, 1H), 3.04 (br d, *J* = 4.9, 1.6 Hz, 1H), 2.94–2.89 (m, 1H), 1.75 (dt, *J* = 8.7, 1.6 Hz, 1H), 1.63 (dq, *J* = 8.9, 1.9 Hz, 1H).

**$^{13}$C NMR** (126 MHz, $CDCl_3$): δ 203.1, 142.8, 139.3, 133.9, 131.8, 129.2, 120.1, 61.1, 48.4, 47.2, 45.3, 45.2.

**HRMS** (EI) (*m*/*z*): calculated for $C_{14}H_{13}O_1Br_1$ [M]: 276.0150; found 276.0147.

$[\boldsymbol{\alpha}]_{\boldsymbol{D}}^{\mathbf{25}}$: –22.6 (*c* = 0.58, $CHCl_3$).

**HPLC**: The enantiomeric ratio was measured by HPLC analysis on a chiral column (OJ-3 column: i.D. 4.6 mm) after reduction with $NaBH_4$/MeOH. Heptane/ *i*PrOH = 70:30, flow rate = 1.0 mL/min, λ = 230 nm, $t_R$ = 3.03 min (major-*endo*) and $t_R$= 5.50 min (minor-*endo*), e.r.(*endo*) = 96:4.

**(1*S*,2*R*,3*R*,4*R*)-3-(4-chlorophenyl)bicyclo[2.2.1]hept-5-ene-2-carbaldehyde (3s)**

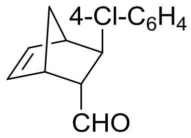


To a solution of catalyst **4i** (1 mol%) and aldehyde **1s** (50 mg, 0.3 mmol, 1.0 equiv) in anhydrous $CH_2Cl_2$ (300 µL), immersed in a liquid nitrogen-ethanol slush at –116 °C, was added diene **2** (99 mg, 1.50 mmol, 5.0 equiv). Then, the resulting reaction mixture was stirred at –110 °C for 5 d. After addition of $NEt_3$ (50 µL) and allowing the mixture to warm to ambient temperature, the mixture was dry loaded onto Celite® and purified by column chromatography on silica gel (*n*-pentane : $Et_2O$ = 85 : 15) to afford **3s** as a white solid (57 mg, 0.24 mmol, 81%).

$^{1}$H NMR and $^{13}$C NMR data for the *endo* isomer were consistent with previously reported values.[13] Diastereoselectivity (*exo*-*endo* ratio) was determined by $^{1}$H NMR analysis of the crude reaction mixture: 9.90 (d, *exo*), 9.59 (d, *endo*), d.r.(*exo*/*endo*) = 2:98.

*endo* isomer

**$^{1}$H NMR** (500 MHz, $CDCl_3$): δ 9.59 (d, *J* = 2.0 Hz, 1H), 7.27 (d, *J* = 8.4 Hz, 2H), 7.19 (d, *J* = 8.4 Hz, 2H), 6.41 (dd, *J* = 5.7, 3.2 Hz, 1H), 6.17 (dd, *J* = 5.7, 2.8 Hz, 1H), 3.35 (br s, 1H), 3.19 (br s, 1H), 3.06 (dd, *J* = 4.9, 1.6 Hz, 1H), 2.91 (ddd, *J* = 5.2, 3.5, 2.1 Hz, 1H), 1.76 (dt, *J* = 8.8, 1.6 Hz, 1H), 1.64 (dq, *J* = 8.7, 1.8 Hz, 1H).

**$^{13}$C NMR** (126 MHz, $CDCl_3$): δ 203.0, 142.1, 139.2, 133.8, 132.0, 128.68, 128.66, 61.0, 48.3, 47.1, 45.13, 45.06.

**HRMS** (ESI–) (*m*/*z*): calculated for $C_{14}H_{12}O_1Cl_1$ $[M]^-$: 231.0582; found 231.0583.

$[\alpha]_D^{25}$: –48.5 (*c* = 0.26, $CHCl_3$).

**HPLC**: The enantiomeric ratio was measured by HPLC analysis on a chiral column (OJ-3 column: i.D. 4.6 mm) after reduction with $NaBH_4$/MeOH. Heptane/ *i*PrOH = 70:30, flow rate = 1.0 mL/min, λ = 230 nm, $t_R$ = 2.95 min (major-*endo*) and $t_R$= 5.89 min (minor-*endo*), e.r.(*endo*) = 95:5.

**(1*S*,2*R*,4*R*)-3-(furan-2-yl)bicyclo[2.2.1]hept-5-ene-2-carbaldehyde (3t)**

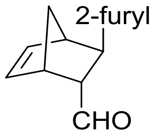


To a solution of catalyst **4i** (3 mol%) and aldehyde **1t** (37 mg, 0.3 mmol, 1.0 equiv) in anhydrous $CH_2Cl_2$ (300 µL), immersed in a liquid nitrogen-ethanol slush at –116 °C, was added diene **2** (99 mg, 1.50 mmol, 5.0 equiv). Then, the resulting reaction mixture was stirred at

–110 °C for 3 d. After addition of $NEt_3$ (50 µL) and allowing the mixture to warm to ambient temperature, the mixture was dry loaded onto Celite® and purified by column chromatography on silica gel (0–70% $CH_2Cl_2$ in hexanes) to afford **3t** as a faint straw-colored solid (34 mg, 0.18 mmol, 60%).

$^{1}$H NMR and $^{13}$C NMR data for the *endo* isomer were consistent with previously reported values.[9] Diastereoselectivity (*exo-endo* ratio) was determined by $^{1}$H NMR analysis of the crude reaction mixture: 9.90 (d, *exo*), 9.56 (d, *endo*), d.r.(*exo*/*endo*) = 1:99.

*endo* isomer

**$^{1}$H NMR** (500 MHz, $CDCl_3$): δ 9.56 (d, *J* = 2.0 Hz, 1H), 7.32 (dd, *J* = 1.9, 0.8 Hz, 1H), 6.35 (dd, *J* = 5.7, 3.1 Hz, 1H), 6.30 (dd, *J* = 3.2, 1.8 Hz, 1H), 6.13 (dd, *J* = 5.7, 2.8 Hz, 1H), 6.07 (d, *J* = 3.2 Hz, 1H), 3.33 (br s, 1H), 3.11 (ddd, *J* = 5.4, 3.5, 2.0 Hz, 1H), 3.08–3.04 (m, 2H), 1.79 (dt, *J* = 8.7, 1.5 Hz, 1H), 1.59–1.54 (m, 1H).

**$^{13}$C NMR** (126 MHz, $CDCl_3$): δ 202.7, 157.0, 141.4, 138.2, 133.8, 110.2, 105.1, 58.4, 48.6, 47.5, 44.7, 39.7.

**HRMS** (EI) (*m*/*z*): calculated for $C_{12}H_{12}O_2$ [M]: 188.0837; found 188.0832.

$[\boldsymbol{\alpha}]_{\boldsymbol{D}}^{\boldsymbol{25}}$: –108.3 (*c* = 0.21, $CHCl_3$).

**GC:** The enantiomeric ratios were measured by GC analysis on a chiral column (BGB 176 column: 25.0 m; i.D. 0.25mm); FID; Temperature: 220 °C (injector), 350 °C (detector), 80 °C (3 min, iso) to 130 °C (15 °C/min, 27 min iso) to 220 °C (15 °C/min, 10 min iso); Gas: $H_2$ (0.50 bar); $t_R$ = 35.78 min (major-*endo*), $t_R$ = 36.60 min (minor-*endo*), e.r.(*endo*) = 95:5.

**(1*S*,2*R*,3*S*,4*R*)-2,3-dimethylbicyclo[2.2.1]hept-5-ene-2-carbaldehyde (3u)**

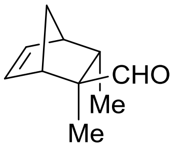


To a solution of catalyst **4h** (1 mol%) in anhydrous $CH_2Cl_2$ (300 µL), immersed in a liquid nitrogen-ethanol slush at –116 °C, were added aldehyde **1u** (25 mg, 0.3 mmol, 1.0 equiv) and diene **2** (99 mg, 1.50 mmol, 5.0 equiv). Then, the resulting reaction mixture was stirred at –100 °C for 3 d. After addition of $NEt_3$ (50 µL) and allowing the mixture to warm to ambient temperature, the mixture was dry loaded onto Celite® and purified by column chromatography on silica gel (*n*-pentane : $Et_2O$ = 95 : 5) to afford **3u** as a colorless oil (41 mg, 0.27 mmol, 90%).

$^1$H NMR and $^{13}$C NMR data for the *endo* isomer were consistent with previously reported values.[11] Diastereoselectivity (*exo*-*endo* ratio) was determined by $^1$H NMR analysis of the crude reaction mixture: 9.64 (s, *exo*), 9.34 (s, *endo*)[9], d.r.(*exo*/*endo*) = 100:0. Absolute configuration was determined by comparison with an authentic sample and from optical rotation.[11]

*exo* isomer

**$^1$H NMR** (500 MHz, $CDCl_3$): δ 9.64 (s, 1H), 6.29 (dd, *J* = 5.7, 3.0 Hz, 1H), 6.20 (dd, *J* = 5.7, 3.1 Hz, 1H), 2.81 (br s, 1H), 2.76 (br s, 1H), 2.54 (qd, *J* = 7.3, 3.4 Hz, 1H), 1.47–1.24 (m, 2H), 0.86 (s, 3H), 0.75 (d, *J* = 7.2 Hz, 3H).

**$^{13}$C NMR** (126 MHz, $CDCl_3$): δ 206.1, 138.0, 135.3, 56.1, 50.4, 49.2, 46.5, 37.4, 15.5, 15.1.

**HRMS** (EI) (*m*/*z*): calculated for $C_{10}H_{14}O_1$ [M]: 150.1039; found 150.1041.

$[\alpha]_D^{25}$: +33.8 (*c* = 0.60, $CHCl_3$).

**GC**: The enantiomeric ratio was measured by GC analysis on a chiral column (BGB 176 column: 25.0 m; i.D. 0.25mm); FID; Temperature: 220 °C (injector), 350 °C (detector), 85 °C (10 min, iso) to 110 °C (1 °C/min, 20 min iso) to 144 °C (15 °C/min) to 144 °C (1 °C/min); Gas: $H_2$ (1.00 bar); $t_R$ = 19.77 min (major-*exo*), $t_R$ = 23.17 min (minor-*exo*), e.r.(*exo*) = 96:4.

**(3a*S*,4*S*,7*R*,7a*S*)-1,2,3,4,7,7a-hexahydro-3a*H*-4,7-methanoindene-3a-carbaldehyde (3v)**

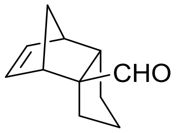

To a solution of catalyst **4i** (1 mol%) in anhydrous $CH_2Cl_2$ (300 µL), immersed in a liquid nitrogen-ethanol slush at –116 °C, were added aldehyde **1v** (29 mg, 0.3 mmol, 1.0 equiv) and diene **2** (99 mg, 1.50 mmol, 5.0 equiv). Then, the resulting reaction mixture was stirred at –100 °C for 3 d. After addition of $NEt_3$ (50 µL) and allowing the mixture to warm to ambient temperature, the mixture was dry loaded onto Celite® and purified by column chromatography on silica gel (0–70% $CH_2Cl_2$ in hexanes) to afford **3v** as a white solid (38 mg, 0.23 mmol, 78%).

$^{1}$H NMR and $^{13}$C NMR data for the *endo* isomer were consistent with previously reported values.[11] Diastereoselectivity (*exo*-*endo* ratio) was determined by $^{1}$H NMR analysis of the crude reaction mixture: 9.74 (s, *exo*), 9.46 (s, *endo*), d.r.(*exo*/*endo*) = 99:1.

*exo* isomer

**$^{1}$H NMR** (500 MHz, $CDCl_3$): δ 9.75 (s, 1H), 6.31 (dd, *J* = 5.8, 2.9 Hz, 1H), 6.21 (dd, *J* = 5.7, 3.2 Hz, 1H), 2.96 (br s, 1H), 2.89–2.83 (m, 2H), 1.88–1.80 (m, 1H), 1.77–1.67 (m, 2H), 1.67–1.58 (m, 2H), 1.58–1.49 (m, 1H), 1.26 (ddd, *J* = 13.3, 9.5, 6.7 Hz, 1H), 1.14 (tt, *J* = 9.6, 7.4 Hz, 1H).

**$^{13}$C NMR** (126 MHz, $CDCl_3$): δ 205.1, 138.9, 136.0, 68.6, 51.7, 50.8, 47.2, 46.4, 29.9, 29.8, 29.5.

**HRMS** (EI) (*m*/*z*): calculated for $C_{11}H_{14}O_1$ [M]: 162.1039; found 162.1039.

$[\alpha]_D^{25}$: +10.2 (*c* = 0.18, $CHCl_3$).

**GC:** The enantiomeric ratios were measured by GC analysis on a chiral column (BGB 176 column: 25.0 m; i.D. 0.25mm); FID; Temperature: 220 °C (injector), 350 °C (detector), 85 °C (10 min, iso) to 110 °C (1 °C/min, 20 min iso) to 144 °C (15 °C/min) to 170 °C (1 °C/min); Gas: $H_2$ (1.00 bar); $t_R$ = 35.42 min (major-*endo*), $t_R$ = 35.89 min (minor-*endo*), $t_R$ = 36.68 min (major-*exo*), $t_R$ = 39.37 min (minor-*endo*), e.r.(*endo*) = 63:37, e.r.(*exo*) = 97:3.

**(1*S*,2*S*,4*S*)-bicyclo[2.2.1]hept-5-ene-2-carbaldehyde (3w)**

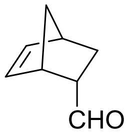


To a solution of catalyst **4h** (1 mol%) in anhydrous $CH_2Cl_2$ (300 µL), immersed in a liquid nitrogen-ethanol slush at –116 °C, were added aldehyde **1w** (17 mg, 0.3 mmol, 1.0 equiv) and diene **2** (99 mg, 1.50 mmol, 5.0 equiv). Then, the resulting reaction mixture was stirred at –110 °C for 2 d. After addition of $NEt_3$ (50 µL) and allowing the mixture to warm to ambient temperature, the mixture was dry loaded onto Celite® and purified by column chromatography on silica gel (0–60% $CH_2Cl_2$ in *n*-pentane) to afford **3w** as a colorless oil (30 mg, 0.25 mmol, 82%).

Diastereoselectivity (*exo*-*endo* ratio) was determined by $^1$H NMR analysis of the crude reaction mixture: 9.76 (d, *exo*), 9.40 (d, *endo*), d.r.(*exo*/*endo*) = 9:91.

*endo* isomer

**$^1$H NMR** (500 MHz, $CD_2Cl_2$): δ 9.40 (d, *J* = 2.7 Hz, 1H), 6.20 (dd, *J* = 5.7, 3.1 Hz, 1H), 5.98 (dd, *J* = 5.7, 2.8 Hz, 1H), 3.23 (br s, 1H), 2.96 (br s, 1H), 2.89 (dtd, *J* = 9.1, 3.8, 2.6 Hz, 1H), 1.88 (ddd, *J* = 11.9, 9.1, 3.7 Hz, 1H), 1.42 (dd, *J* = 4.1, 2.5 Hz, 1H), 1.39 (dd, *J* = 4.2, 2.5 Hz, 1H), 1.32 (dt, *J* = 8.3, 1.6 Hz, 1H).

**$^{13}$C NMR** (126 MHz, $CD_2Cl_2$): δ 204.6, 138.0, 131.8, 52.2, 49.5, 45.0, 42.8, 27.4.

**HRMS** (EI) (*m*/*z*): calculated for $C_8H_{10}O_1$ [M]: 122.0723; found 122.0728.

**GC**: The enantiomeric ratios were measured by GC analysis on a chiral column (Hydrodex-gamma-TBDAc column: 25.0 m; i.D. 0.25mm); FID; Temperature: 220 °C (injector), 350 °C (detector), 80 °C (50 min, iso); Gas: $H_2$ (0.40 bar); $t_R$ = 17.98 min (minor-*exo*), $t_R$ = 19.92 min (major-*exo*), $t_R$ = 28.96 min (minor-*endo*), $t_R$ = 29.94 min (major-*endo*), e.r.(*endo*) = 84:16.

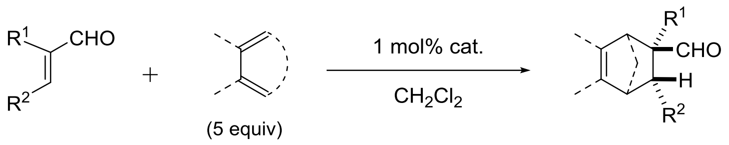


| entry | enal | diene | Temp (°C) | Conv. (%) | Yield (%) | e.r. |
|---|---|---|---|---|---|---|
| 1 | **1b** | 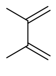 | –40 | 51 | 50 | 83:17 |
| 2 | **1b** | | –20 | 100 | 89 | 91:9 |
| 3 | **1b** | | 0 | 100 | 61 | 89:11 |
| 4 | **1c** | 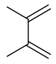 | –40 | 99 | 15 | 82:18 |
| 5 | **1c** | | –20 | 99 | 15 | 84:16 |
| 6 | **1c** | | 0 | 99 | 6 | 87:13 |

**Supplementary Table 6.** Diene Scope.[a]: a. Reactions were performed with aldehydes (0.06 mmol), diene, and catalyst **4h** in $CH_2Cl_2$. b. Conversions were determined by $^1$H NMR analysis by comparison to *p*-nitrobenzaldehyde as an internal standard after addition of TEA. c. The enantiomeric ratios were measured by GC analysis on a chiral stationary phase.

### ***Gram Scale Reactions***

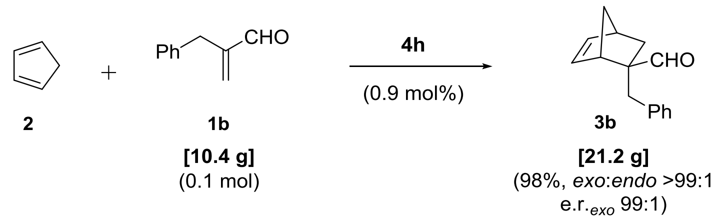


To a flame-dried Schlenk tube, charged with aldehyde **1b** (10.4 g, 102 mmol, 1.0 equiv) and anhydrous $CH_2Cl_2$ (85.0 mL), immersed in a liquid nitrogen-ethanol slush at –116 °C, was added diene **2** (33.7 g, 510 mmol, 5.0 equiv) under an argon atmosphere. To the stirred, clear mixture was added dropwise a solution of catalyst **4h** (1.6 g, 1.0 mmol, 0.9 mol%) in anhydrous $CH_2Cl_2$ (17.0 mL). Then, the resulting reaction mixture was stirred at –100 °C for 2 d. Upon completion of the reaction, $NEt_3$ (280 µL) was added and the reaction mixture was warmed to ambient temperature. After removal of the solvent, the crude mixture was loaded onto Celite® and purified by column chromatography on silica gel (*n*-pentane : $Et_2O$ = 90 : 10) to afford product **3b** as a colorless oil (21.2 g, 100 mmol, 98%, d.r.(*exo*/*endo*) > 99:1, e.r.(*exo*) = 99:1) and to recover catalyst **4h** (1.6 g, 1.0 mmol, 97%).

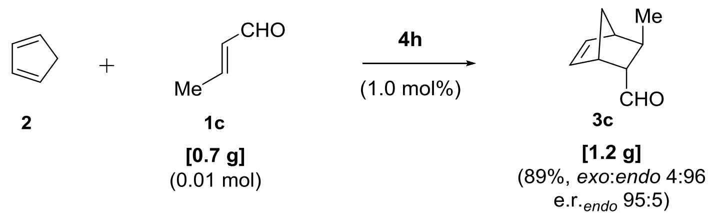


To a flame-dried Schlenk tube, charged with catalyst **4h** (170 mg, 0.1 mmol, 1 mol%) and anhydrous $CH_2Cl_2$ (10.0 mL), immersed in a liquid nitrogen-ethanol slush at –116 °C, was added aldehyde **1c** (0.7 g, 10 mmol, 1.0 equiv) under an argon atmosphere. To the stirred, clear mixture was added dropwise diene **2** (3.3 g, 50 mmol, 5.0 equiv). Then, the resulting reaction mixture was stirred at –100 °C for 2 d. Upon completion of the reaction, $NEt_3$ (50 µL) was added and the reaction mixture was warmed to ambient temperature. After removal of the solvent, the crude mixture was loaded onto Celite® and purified by column chromatography on silica gel (*n*-pentane : $Et_2O$ = 90 : 10) to afford product **3c** as a colorless oil (1.2 g, 8.9 mmol, 89%, d.r.(*exo*/*endo*) 4:96, e.r.(*endo*) = 95:5) and to recover catalyst **4h** (160 mg, 0.09 mmol, 92%).

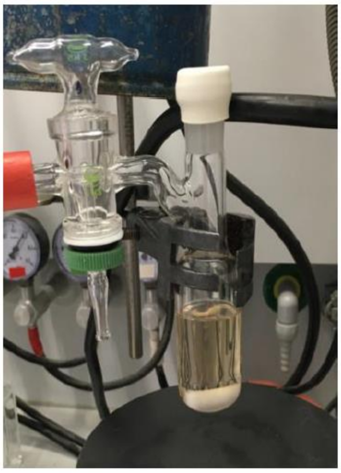

1. catalyst **4p** in $CH_2Cl_2$

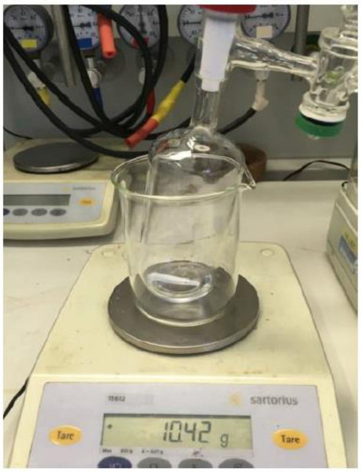


2. aldehyde **1b**

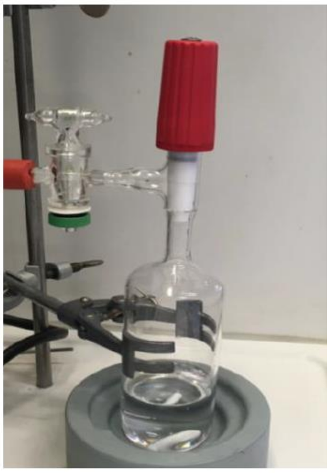

3. aldehyde **1b** in $CH_2Cl_2$

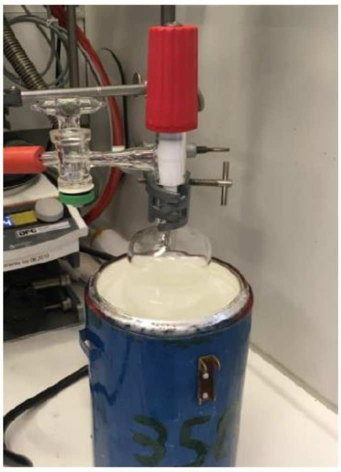

4. solution immersed in a $N_2(l)$-EtOH slush

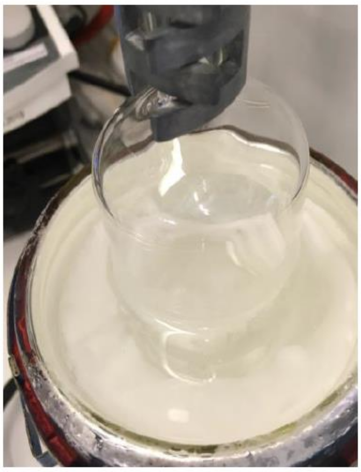

5. diene **2** added

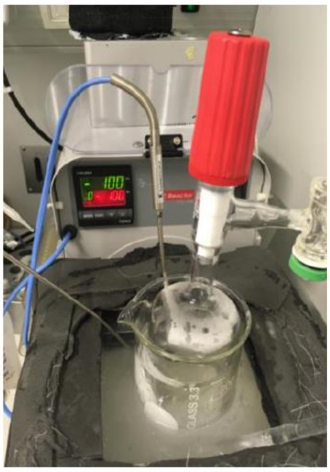

6. running at –100 °C after adding the catalyst **4h** solution

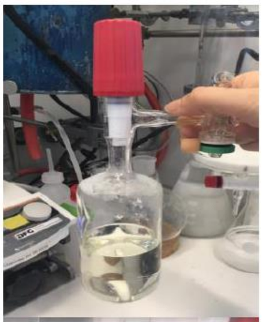

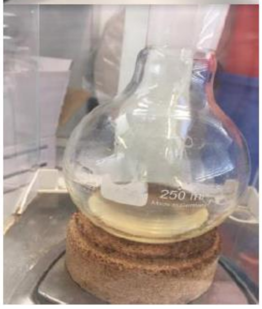

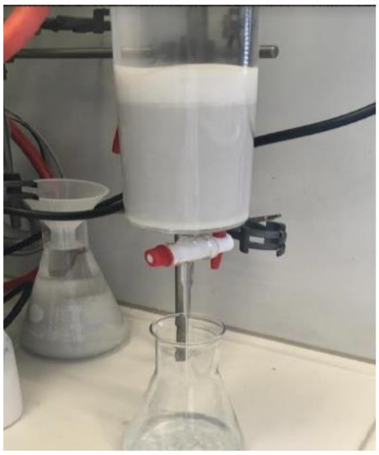

7. completion of the reaction, evaporation of the solvent, then column chromatography

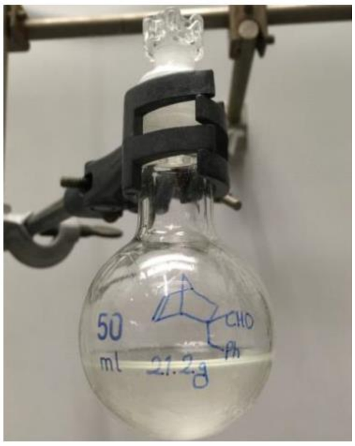


8. product **1b** (21.2 g)

**Supplementary Figure 2b.** Illustrated large-scale experiment.

## **Supplementary Note 3.** Copies of NMR Spectra

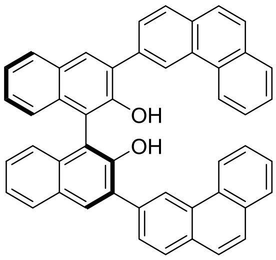

**S4**

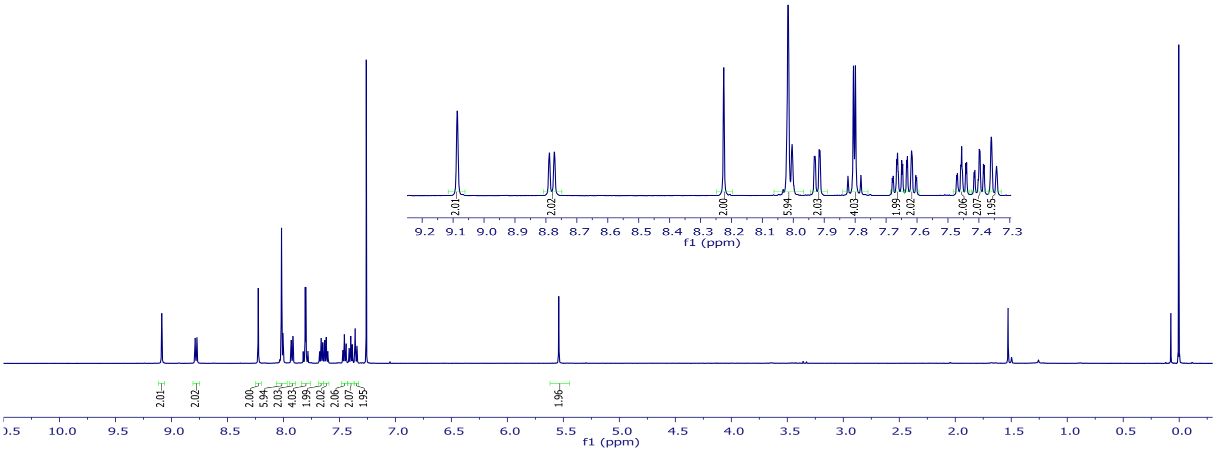

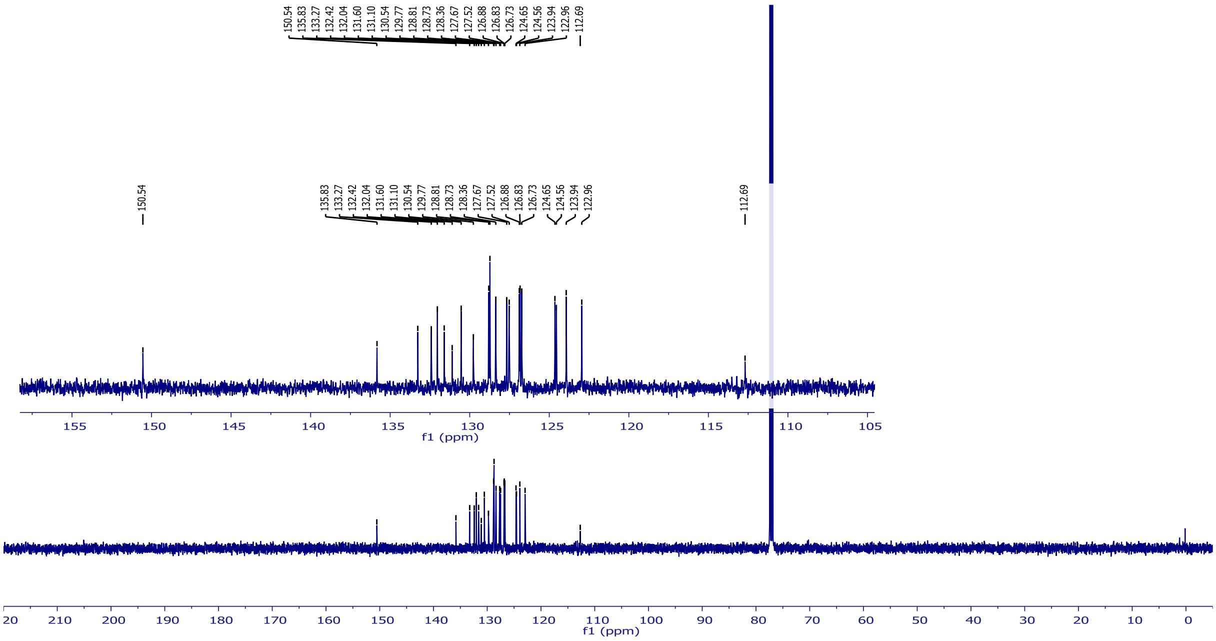

**Supplementary Figure 3.** $^{1}H$ and $^{13}C$ NMR spectra of **S4**

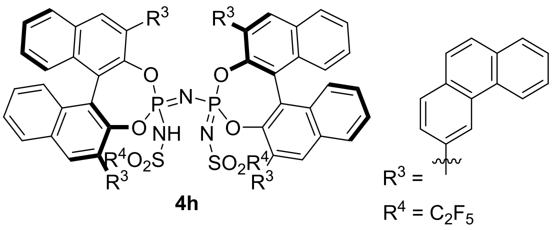

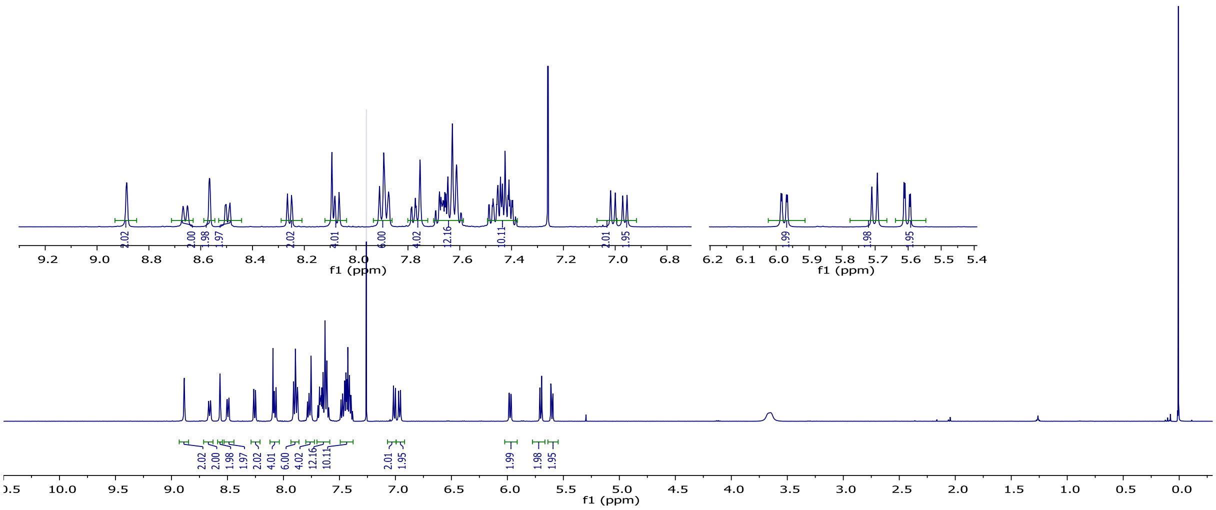

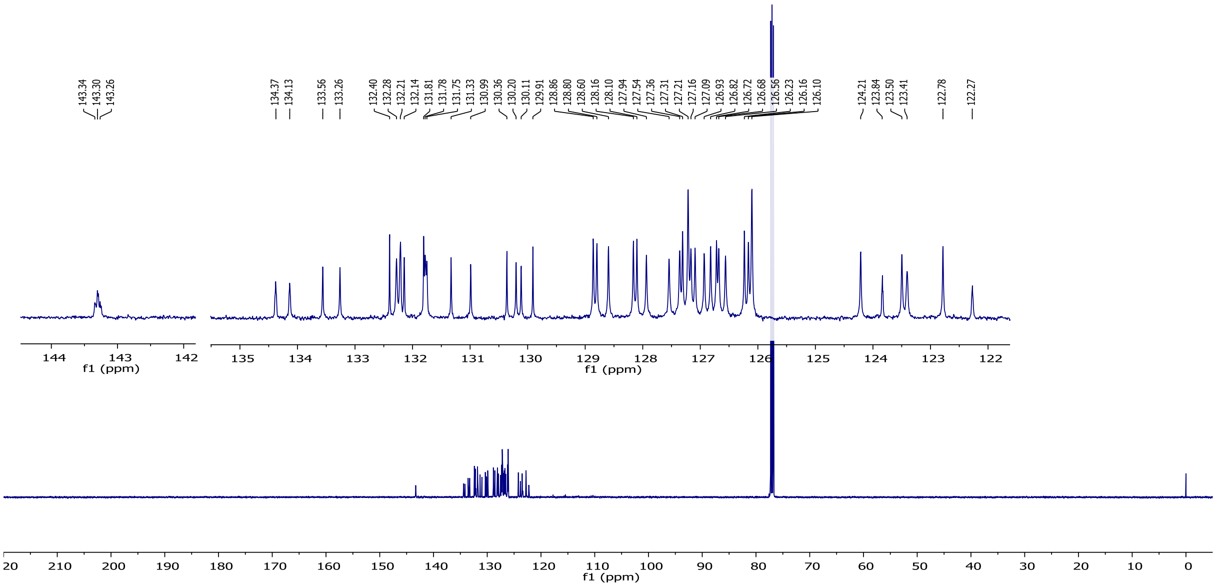

**Supplementary Figure 4.** ¹H and ¹³C NMR spectra of **4h**

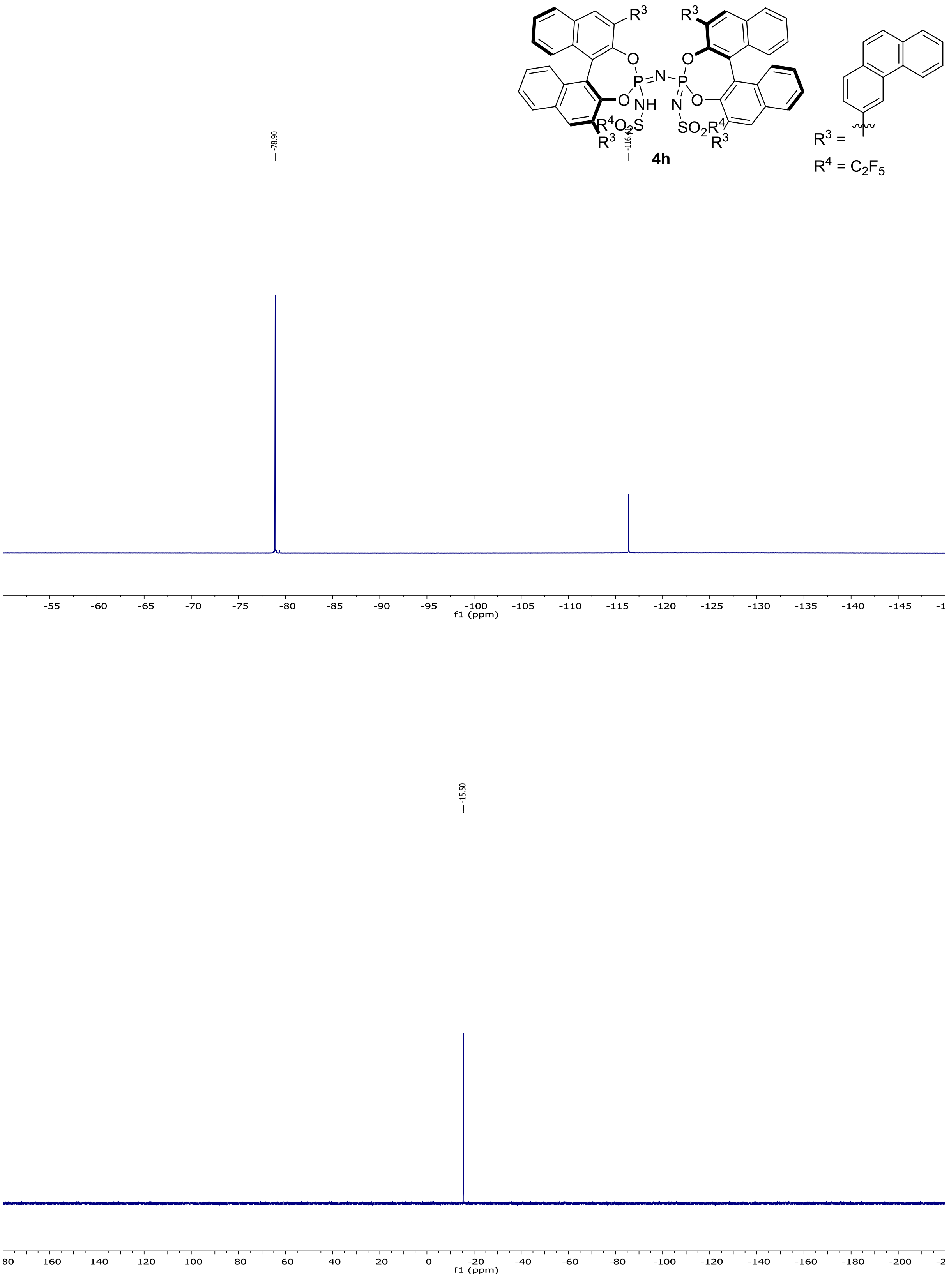


**Supplementary Figure 5.** $^{19}F$ and $^{31}P$ NMR spectra of **4h**

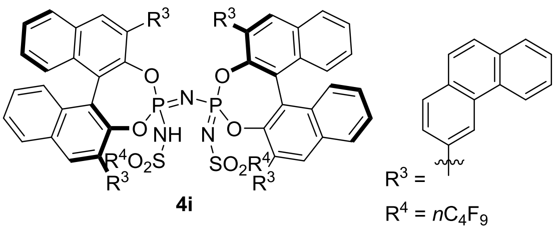

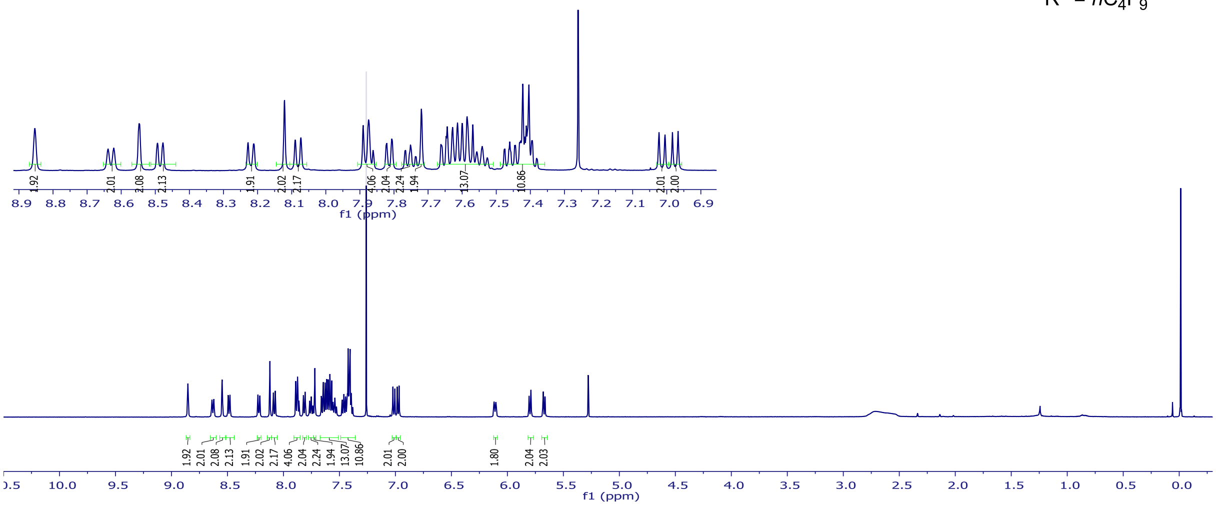

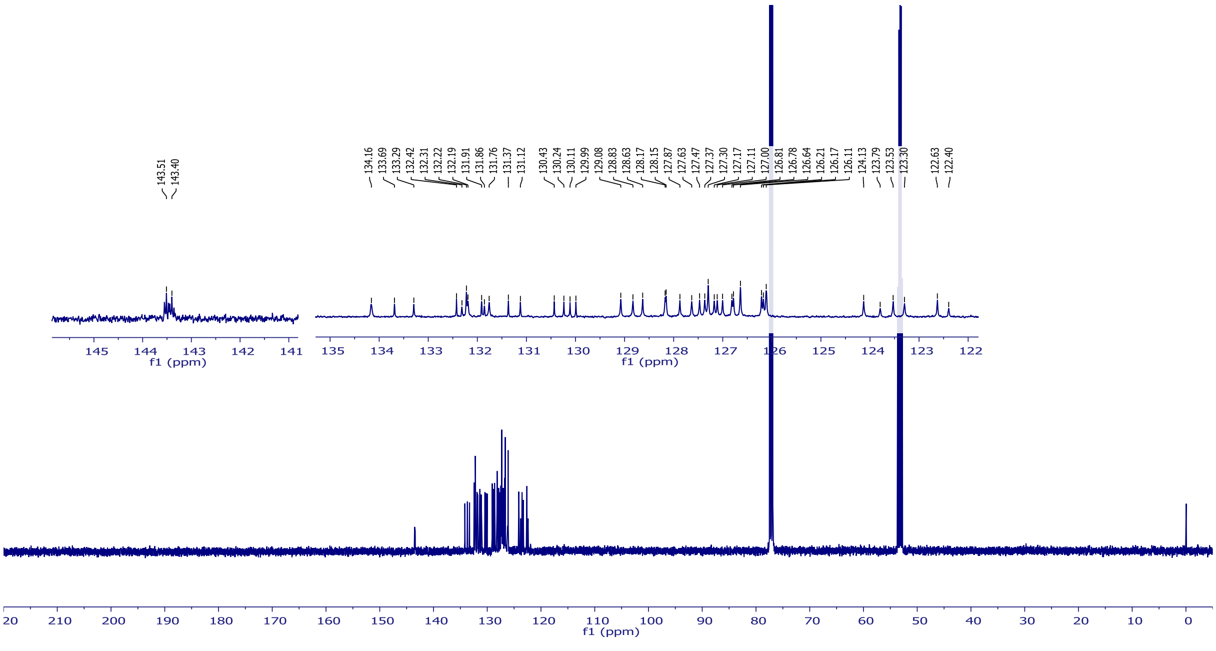

**Supplementary Figure 6.** $^{1}H$ and $^{13}C$ NMR spectra of **4i**

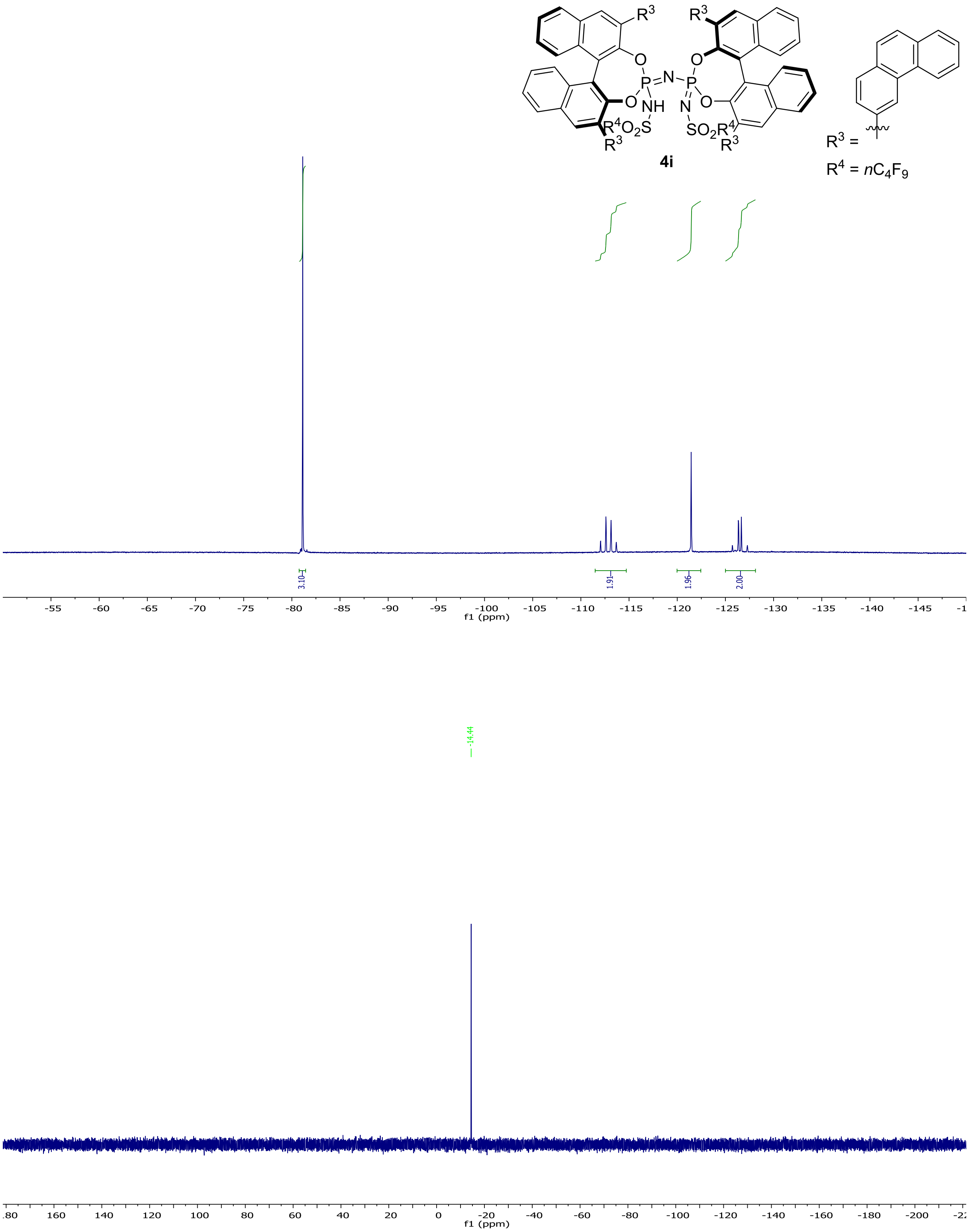


**Supplementary Figure 7.** $^{19}F$ and $^{31}P$ NMR spectra of **4i**

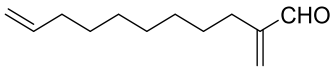


**1h**

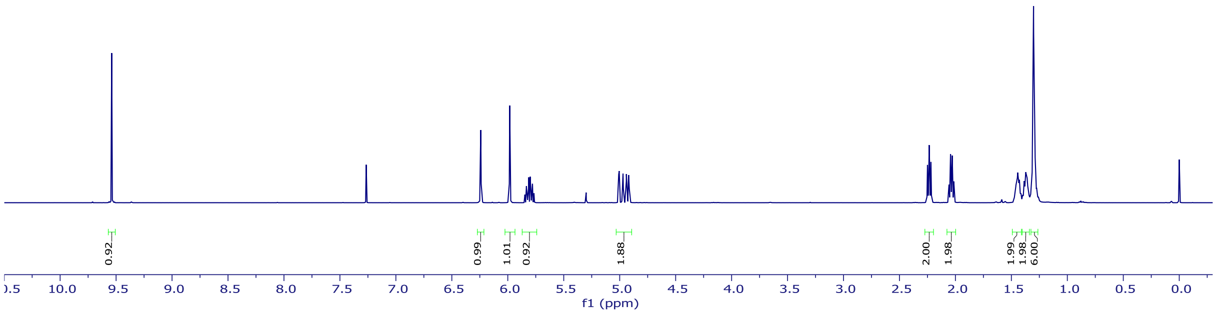


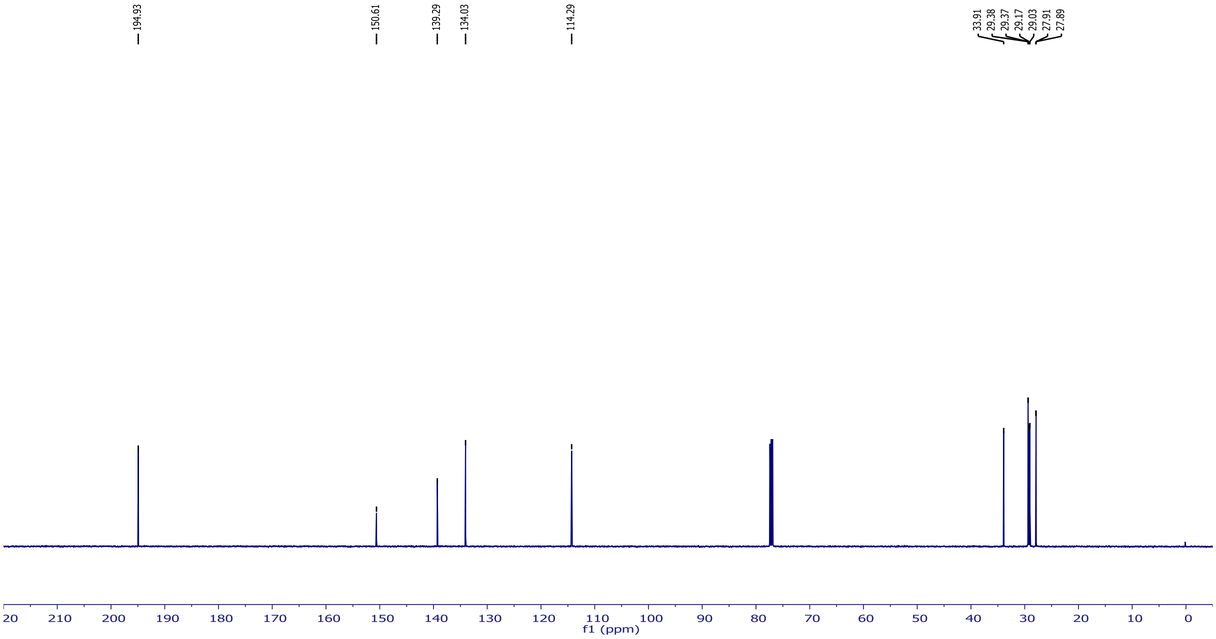


**Supplementary Figure 8.** $^{1}$H and $^{13}$C NMR spectra of **1h**

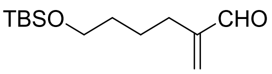


**1i**

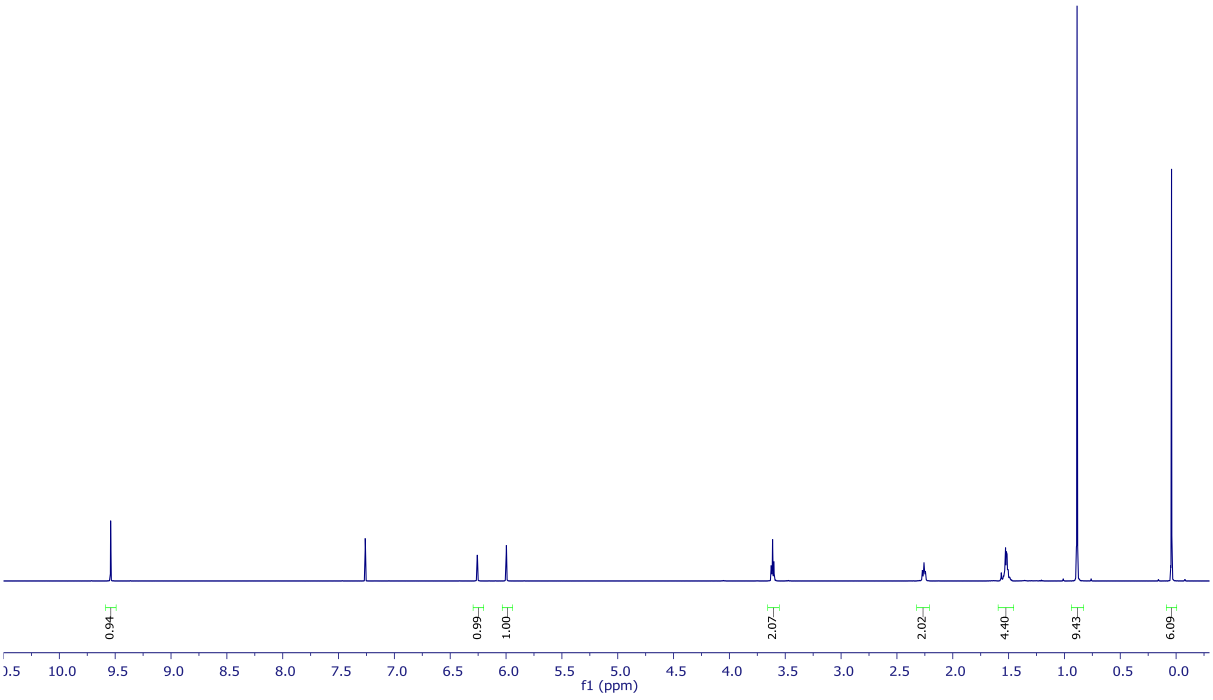


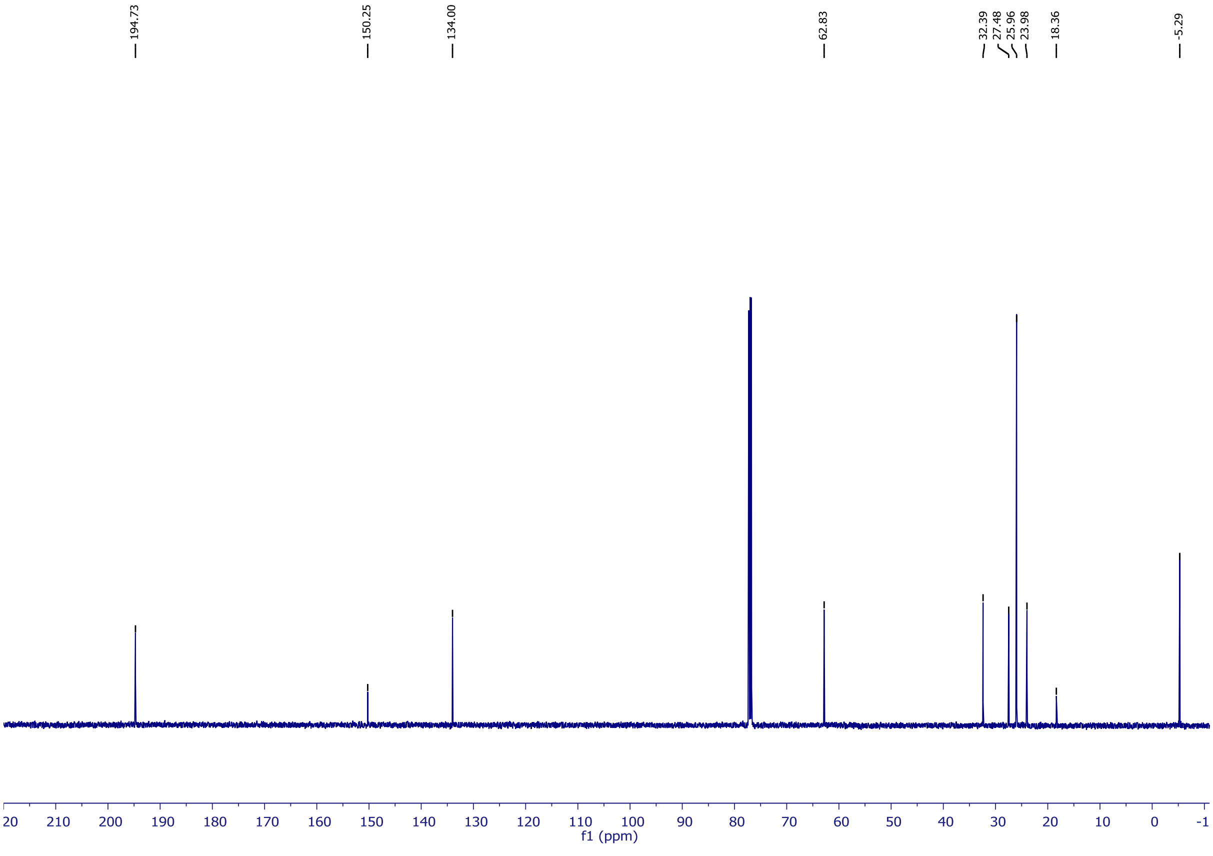


**Supplementary Figure 9.** $^{1}H$ and $^{13}C$ NMR spectra of **1i**

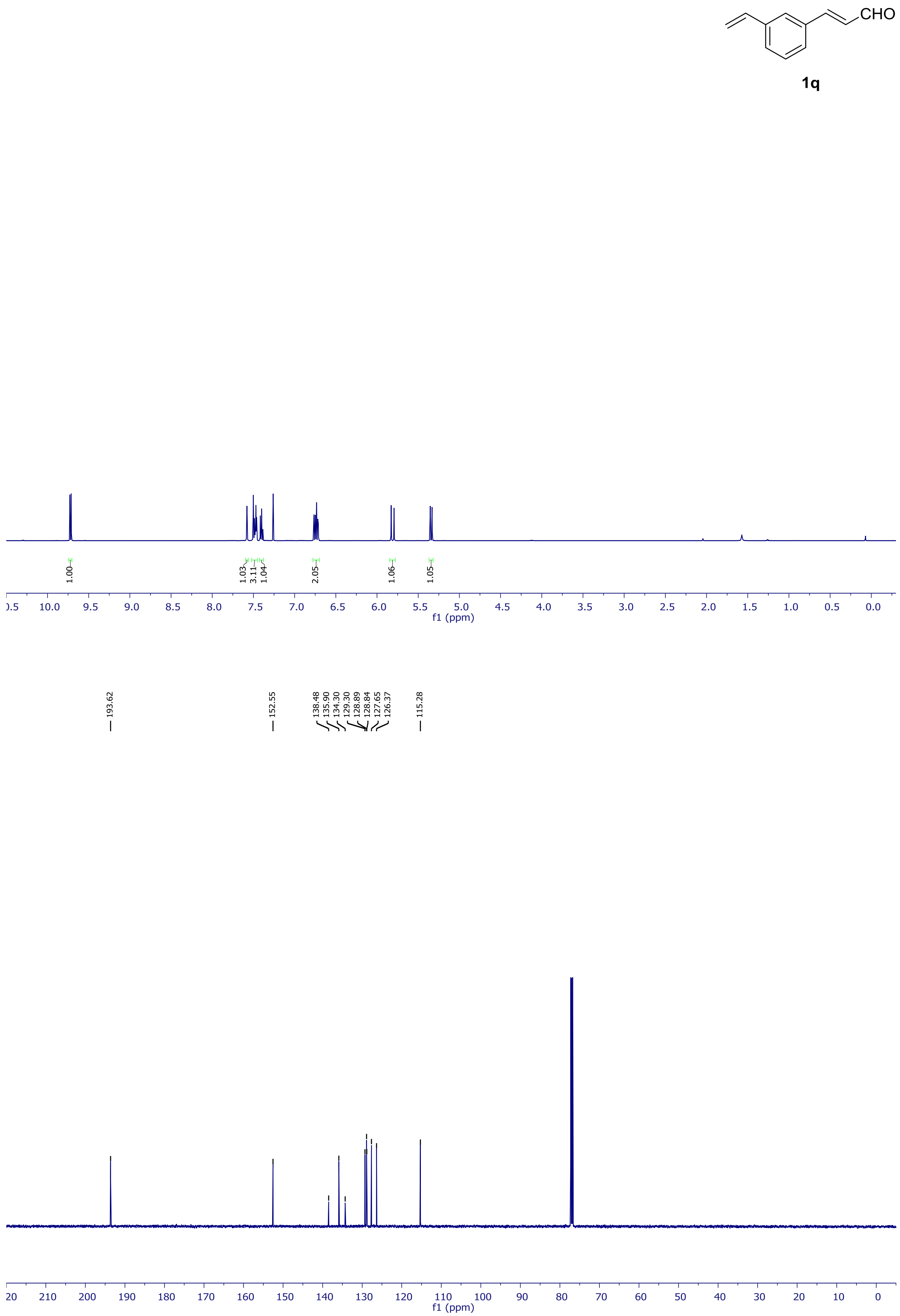


**Supplementary Figure 10.** $^{1}H$ and $^{13}C$ NMR spectra of **1q**

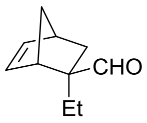


**3a**

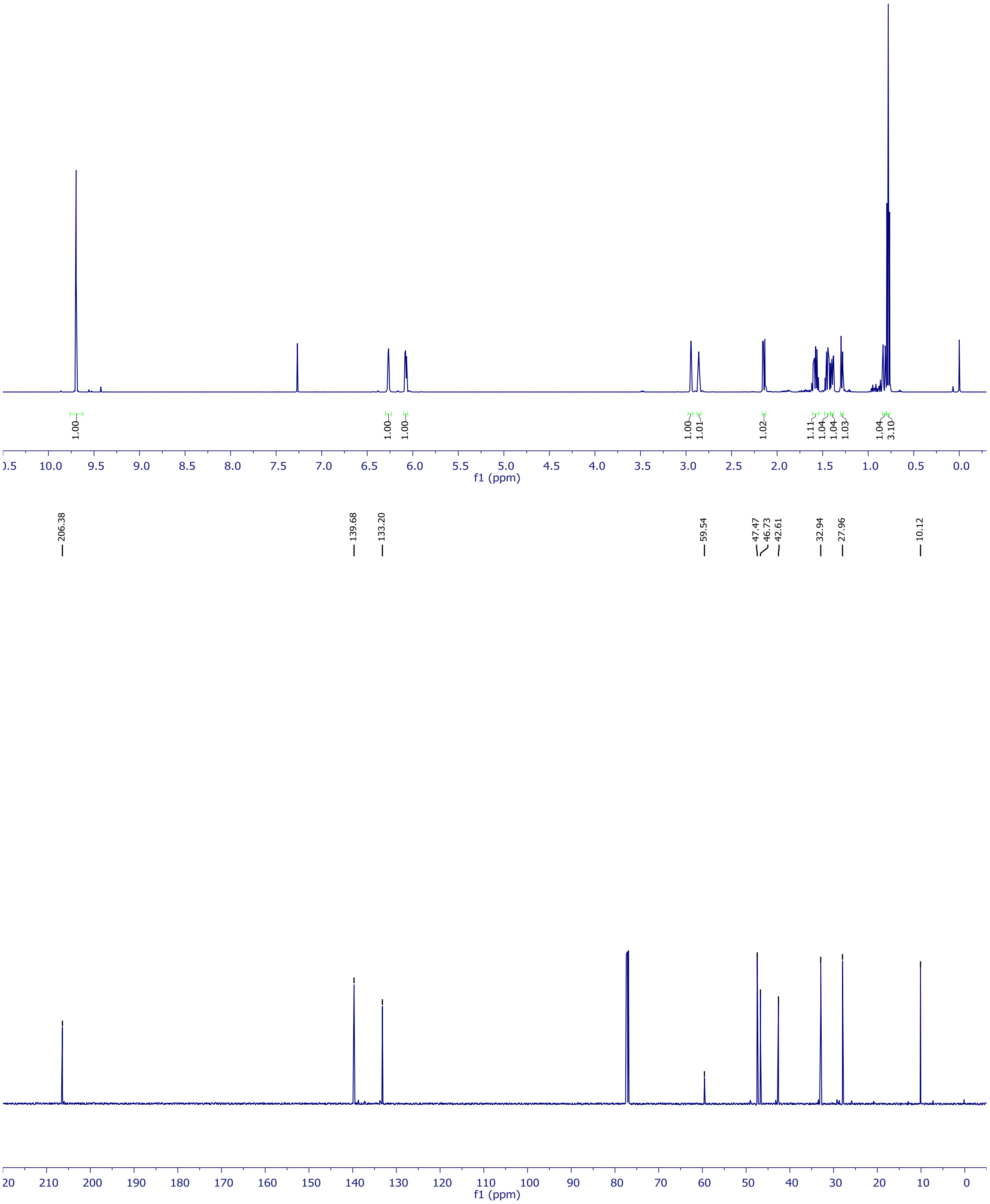


**Supplementary Figure 11.** $^{1}H$ and $^{13}C$ NMR spectra of **3a**

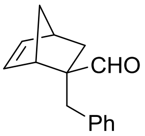


**3b**

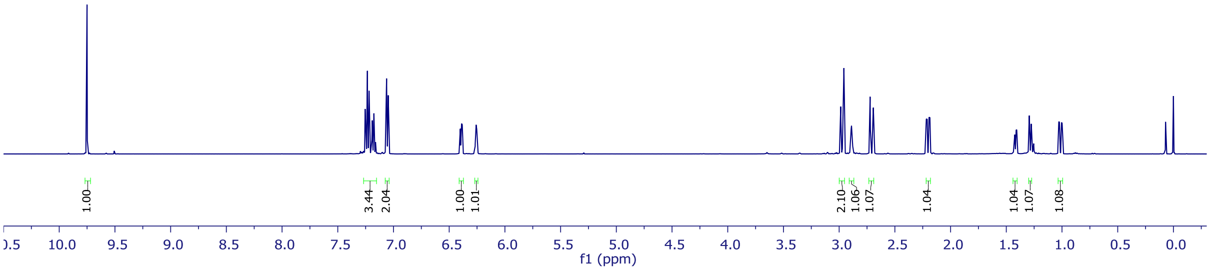


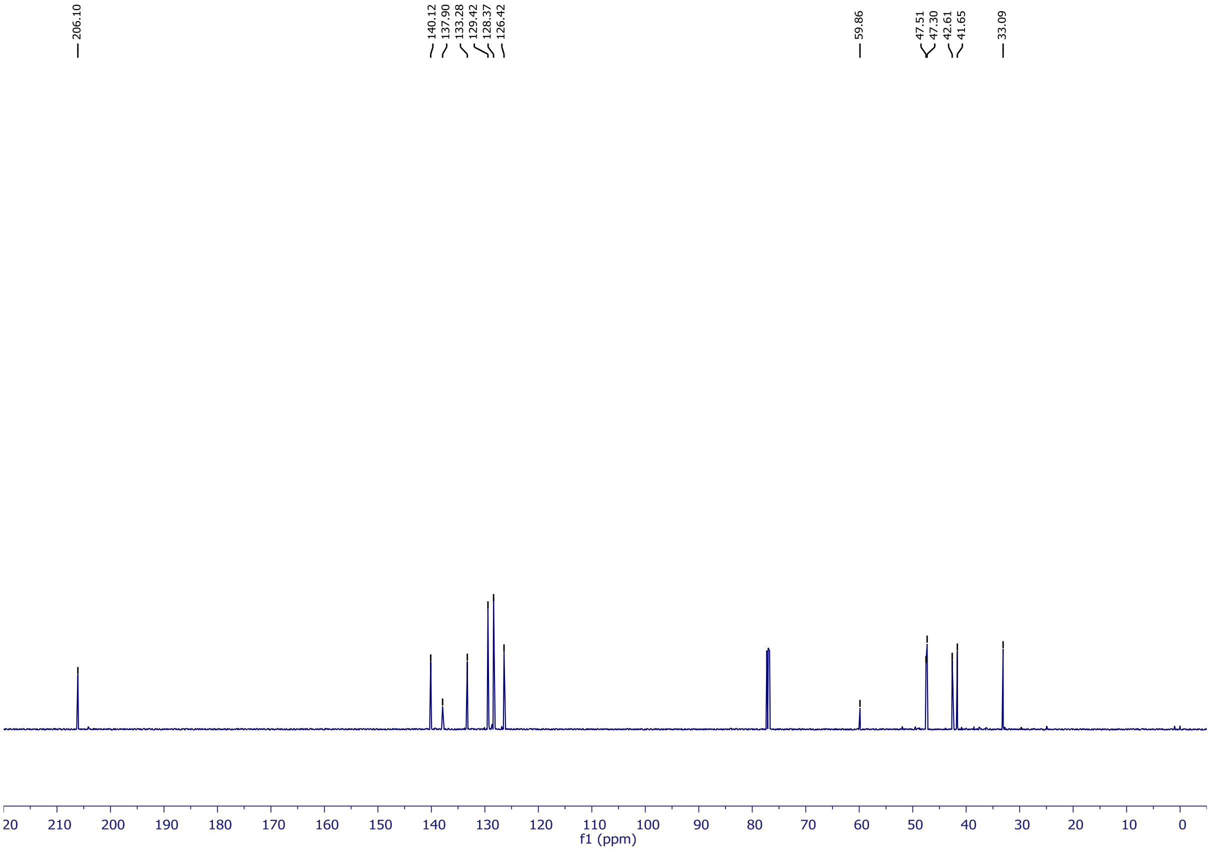


**Supplementary Figure 12.** $^{1}$H and $^{13}$C NMR spectra of **3b**

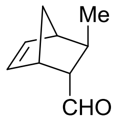


**3c**

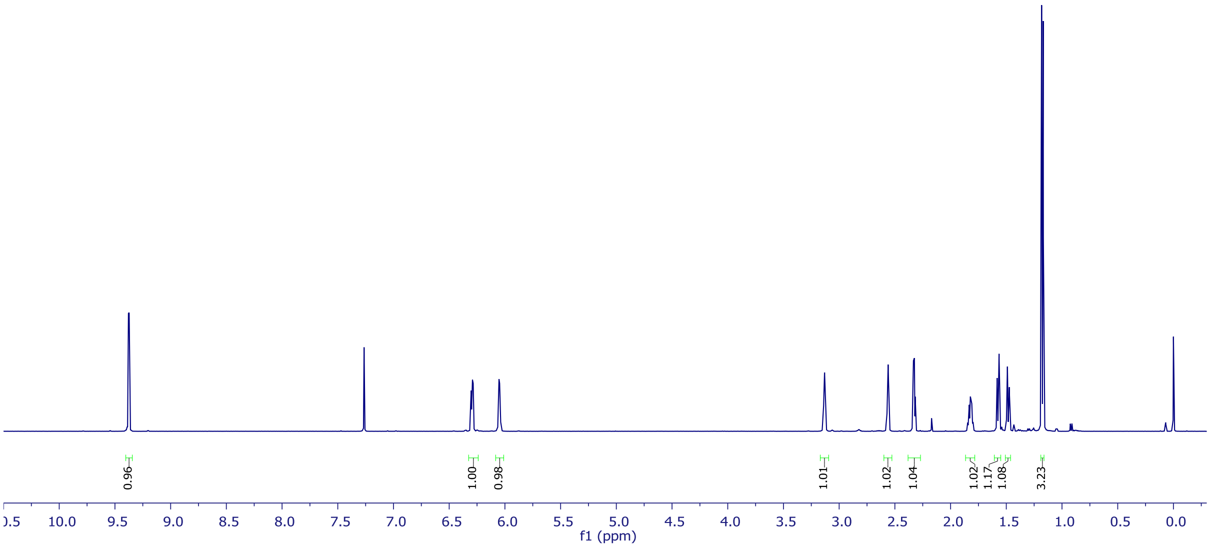


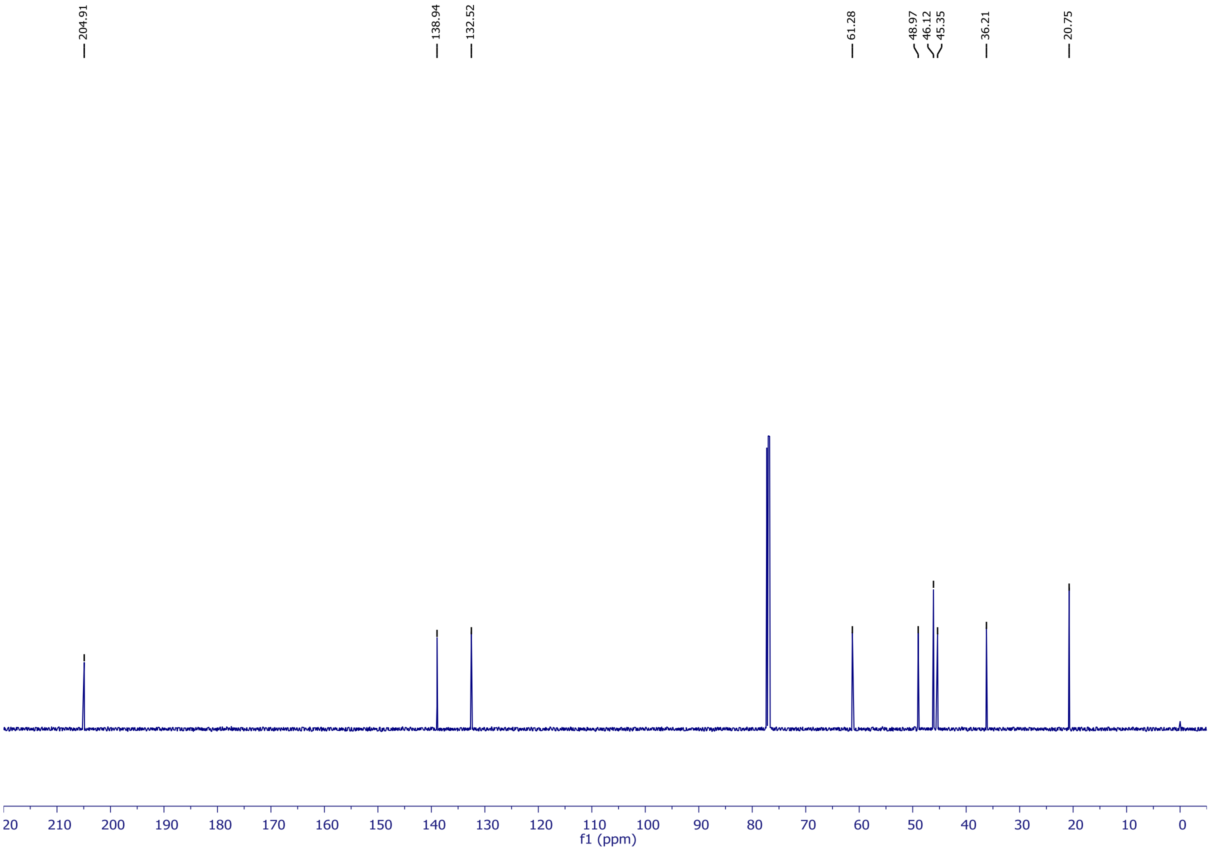


**Supplementary Figure 13.** $^{1}$H and $^{13}$C NMR spectra of **3c**

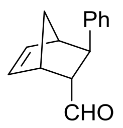


**3d**

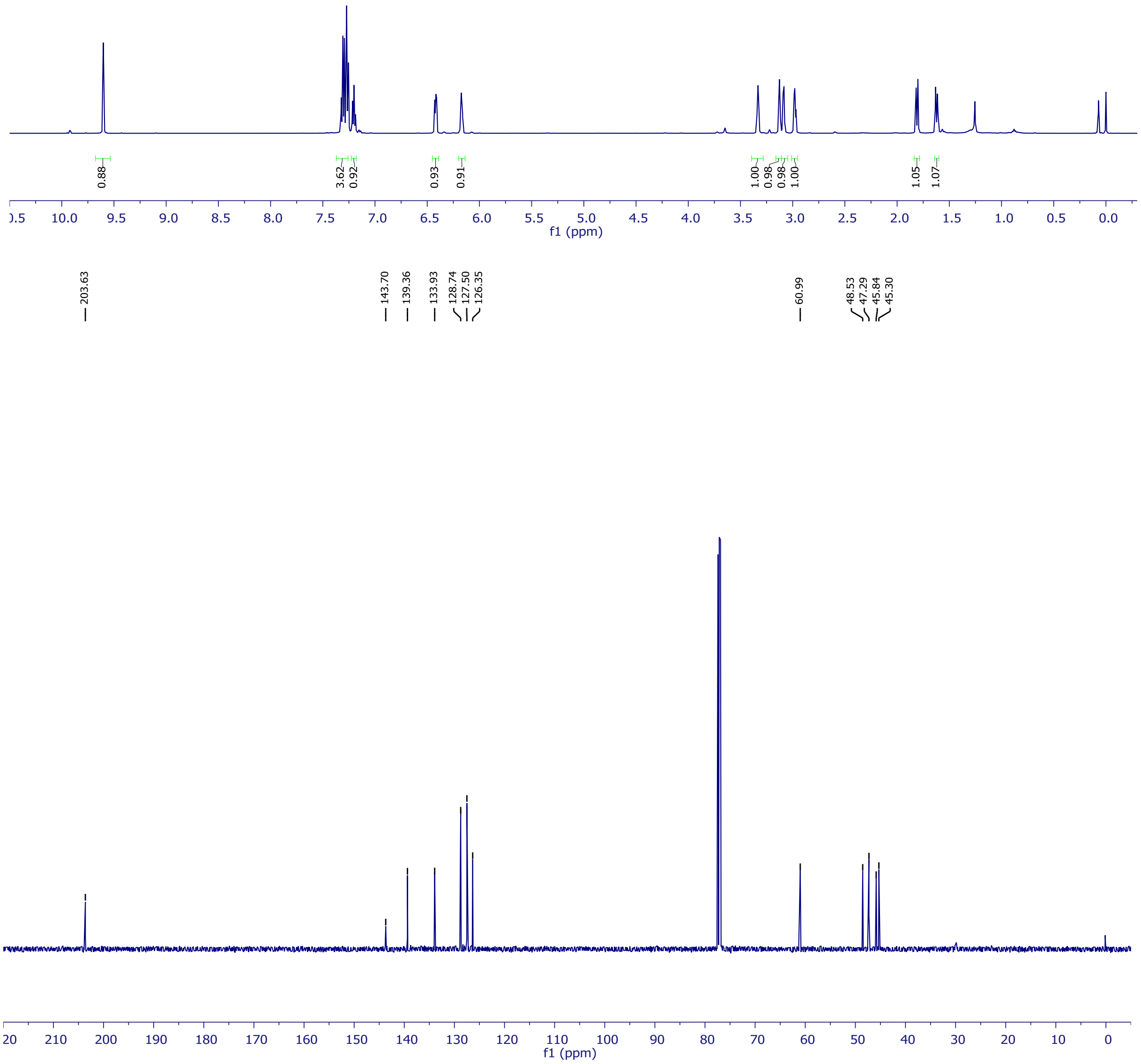

**Supplementary Figure 14.** $^{1}H$ and $^{13}C$ NMR spectra of **3d**

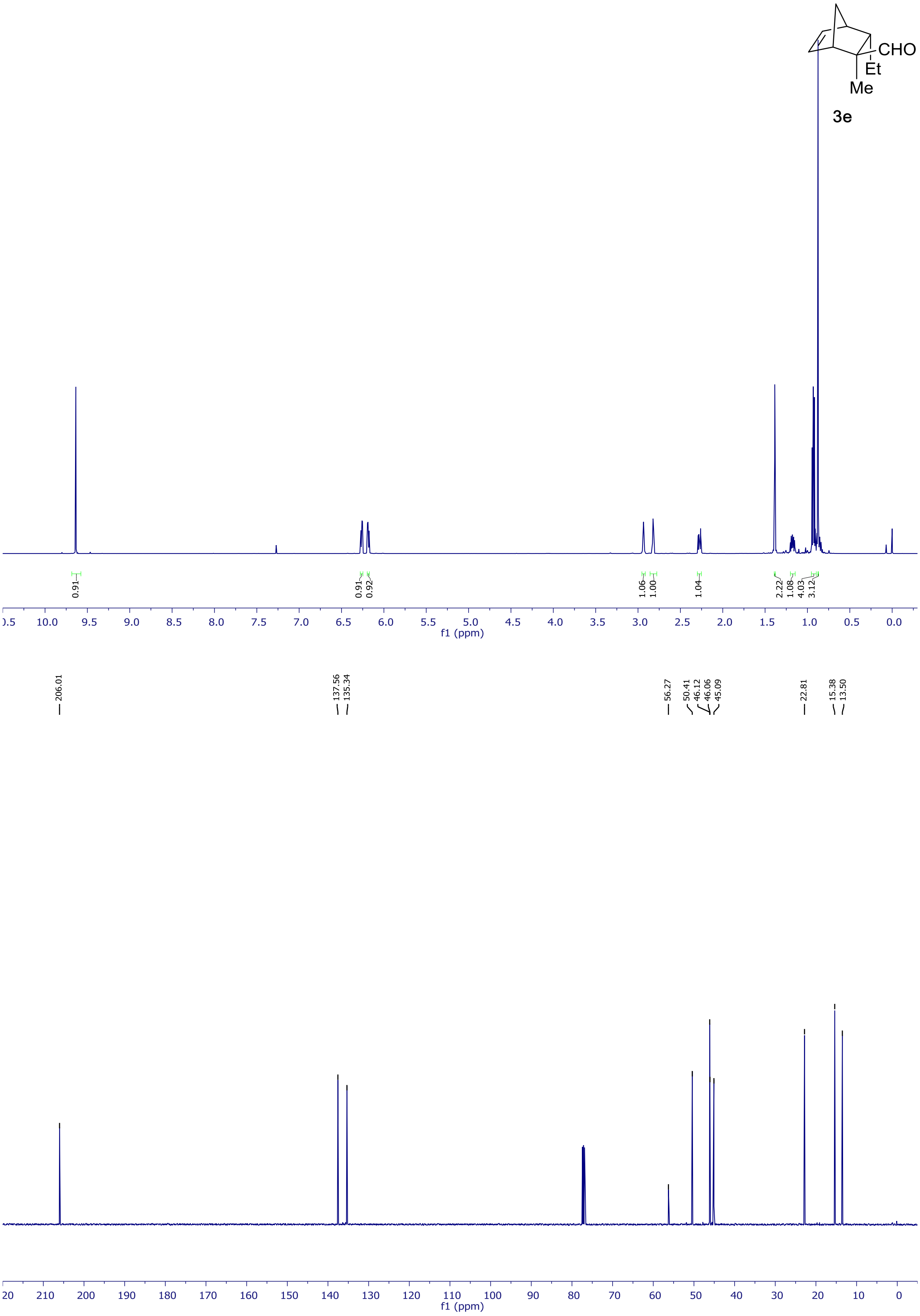


**Supplementary Figure 15.** $^{1}H$ and $^{13}C$ NMR spectra of **3e**

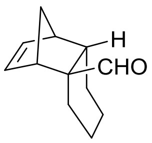


3f

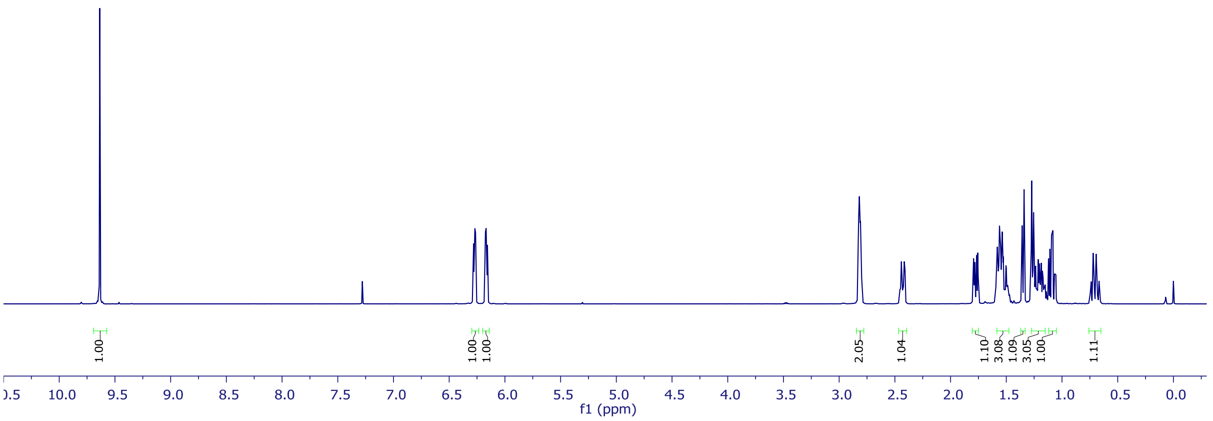


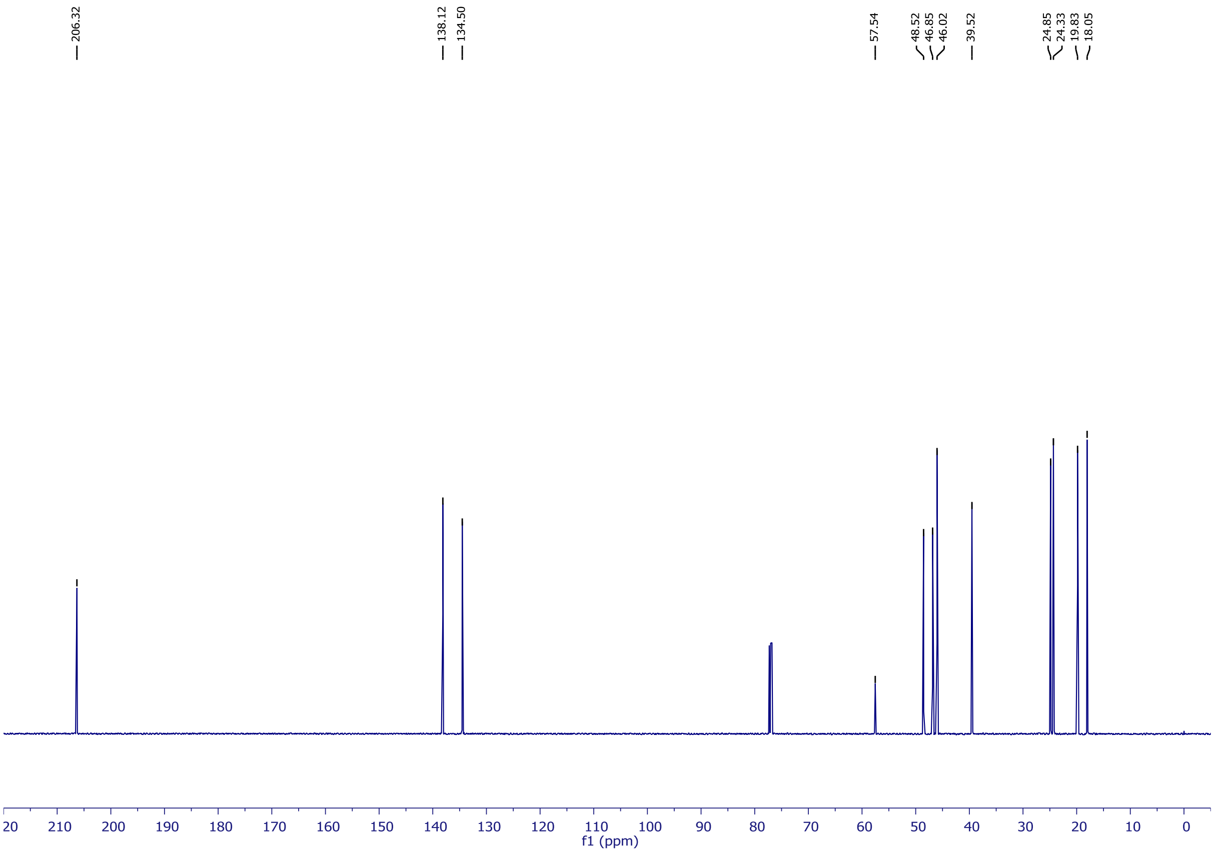


**Supplementary Figure 16.** $^{1}$H and $^{13}$C NMR spectra of **3f**

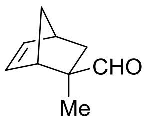


**3g**

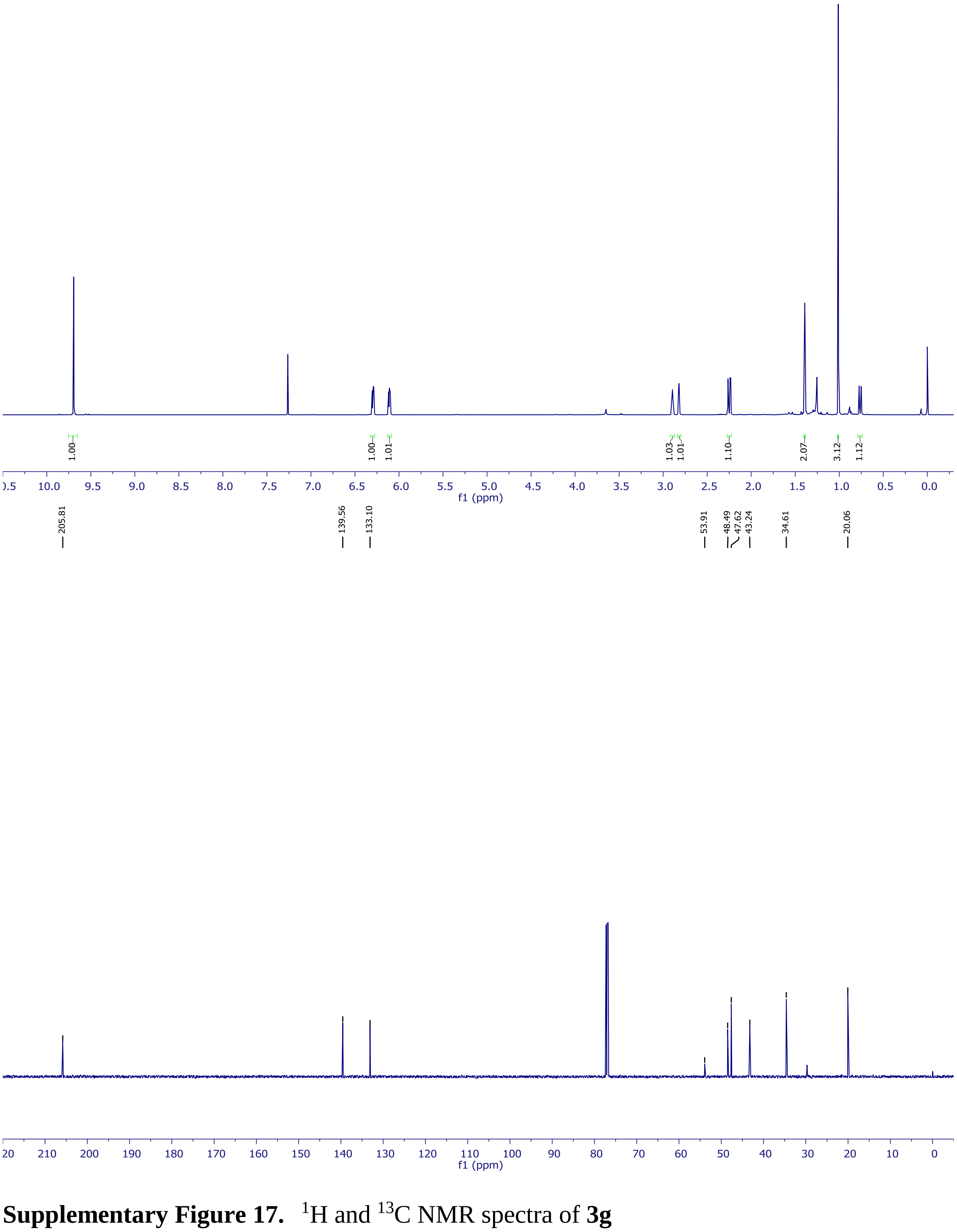


**Supplementary Figure 17.** $^{1}$H and $^{13}$C NMR spectra of **3g**

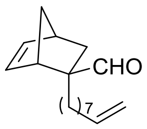


**3h**

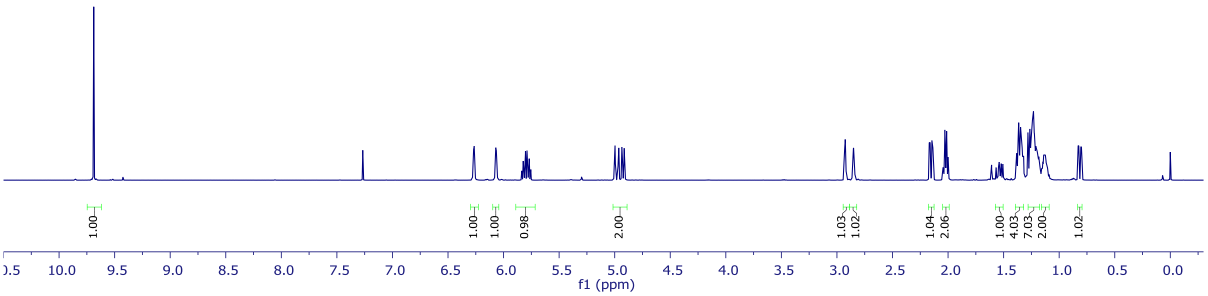


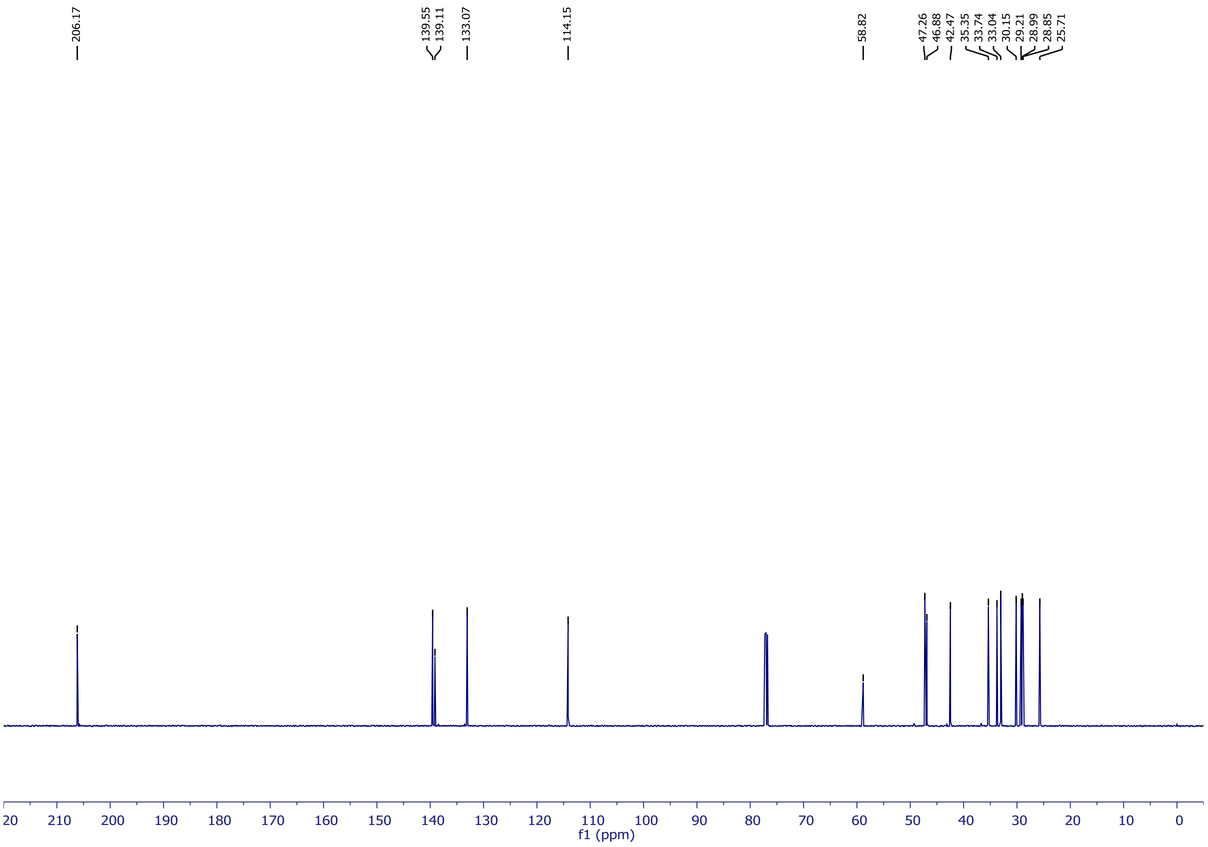


**Supplementary Figure 18.** $^{1}$H and $^{13}$C NMR spectra of **3h**

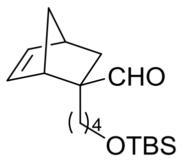


**3i**

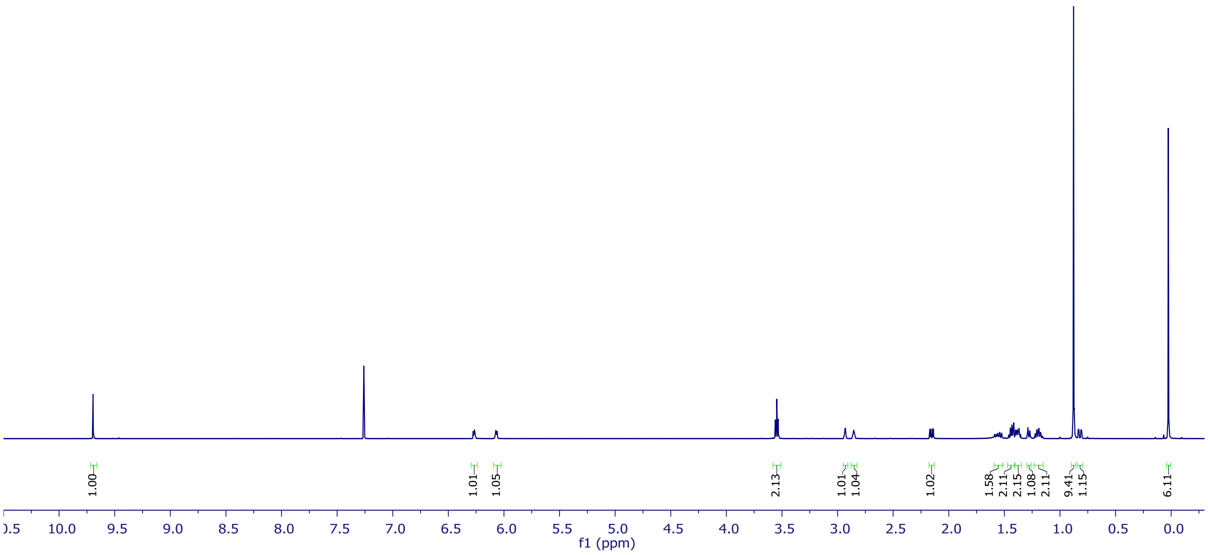

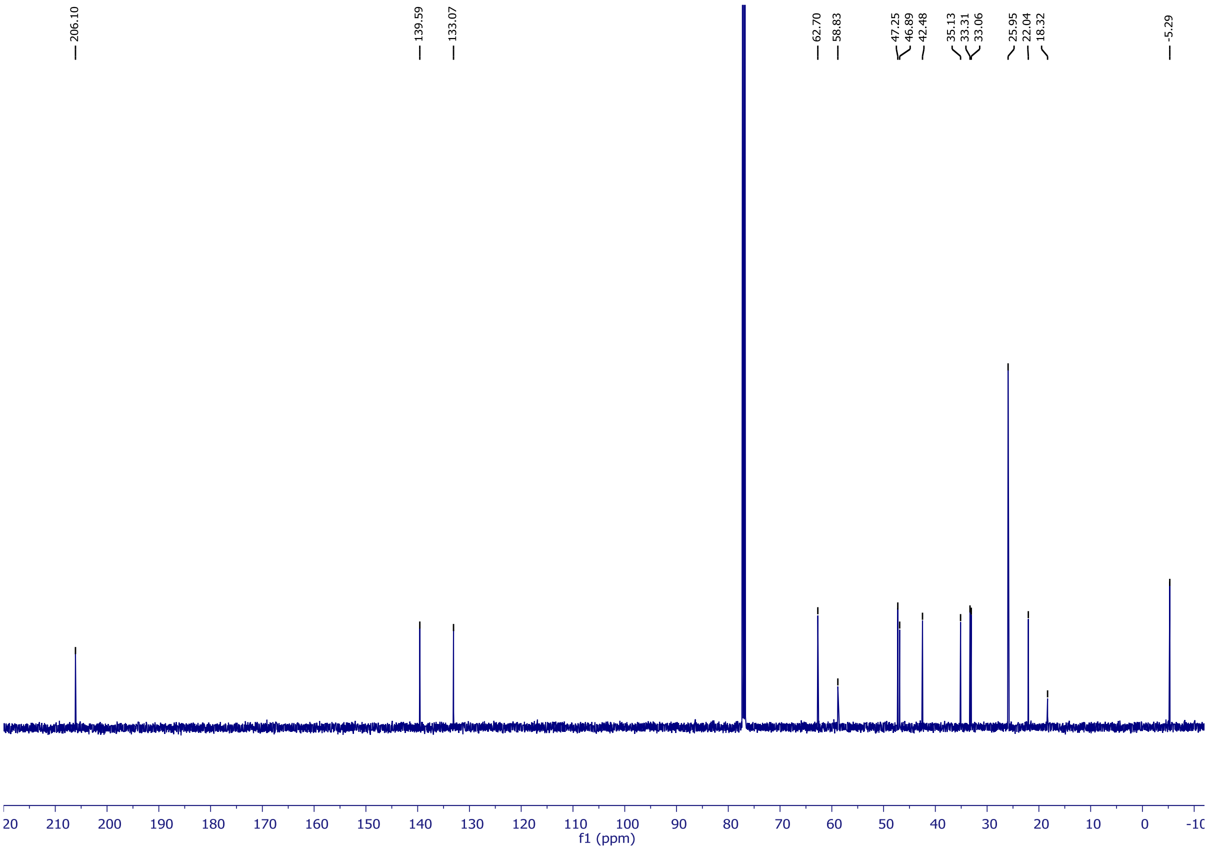

**Supplementary Figure 19.** $^{1}H$ and $^{13}C$ NMR spectra of **3i**

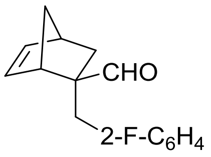


**3j**

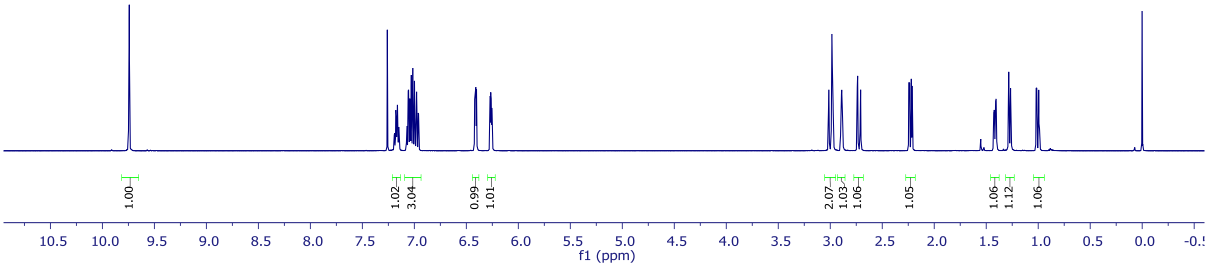


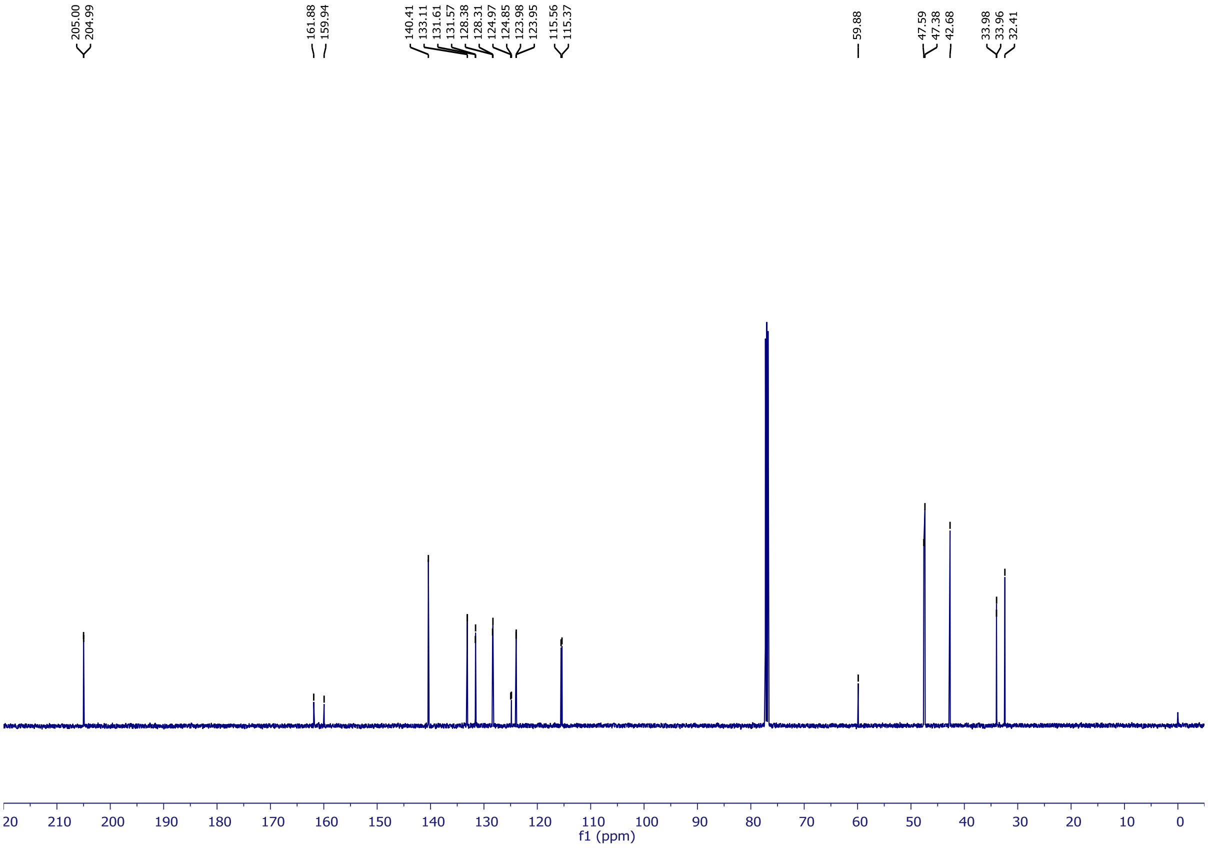


**Supplementary Figure 20.** $^{1}H$ and $^{13}C$ NMR spectra of **3j**

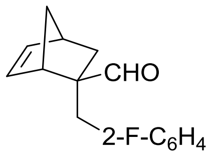


3j

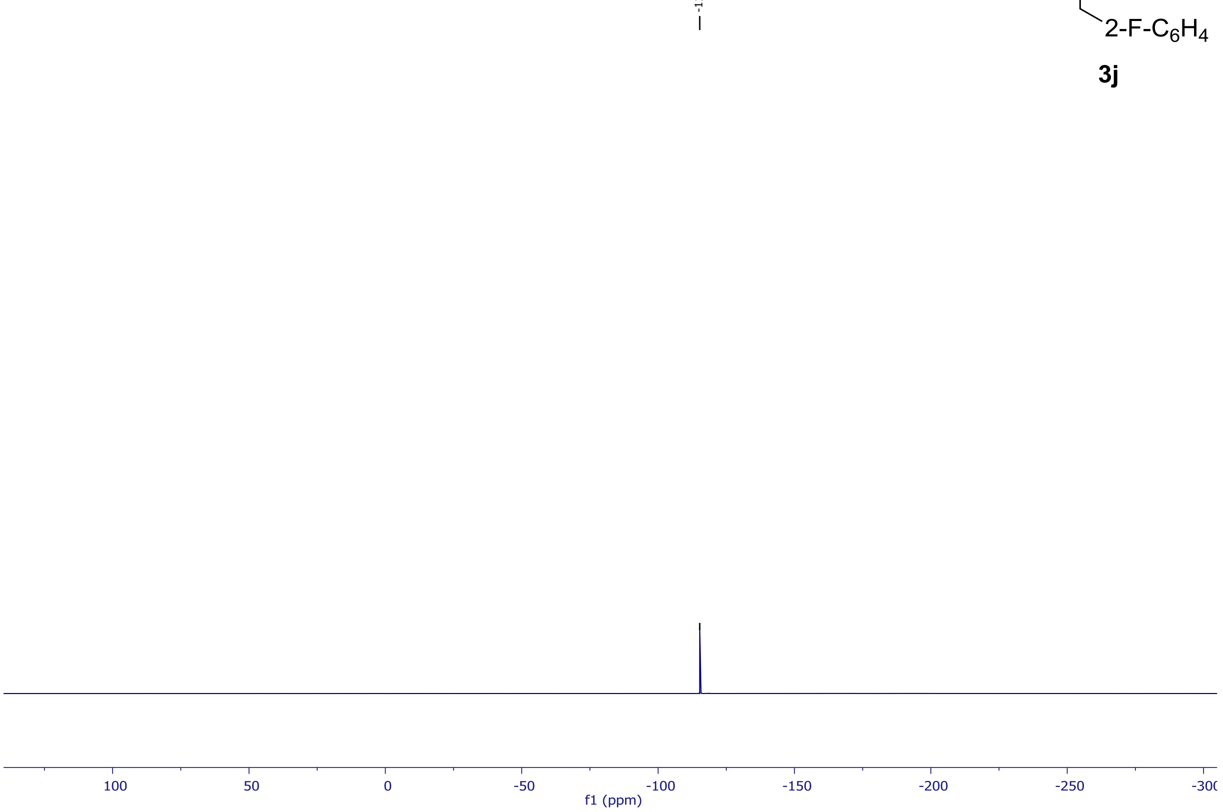


**Supplementary Figure 21.** $^{19}$F NMR spectrum of **3j**

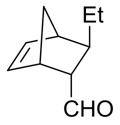


**3k**

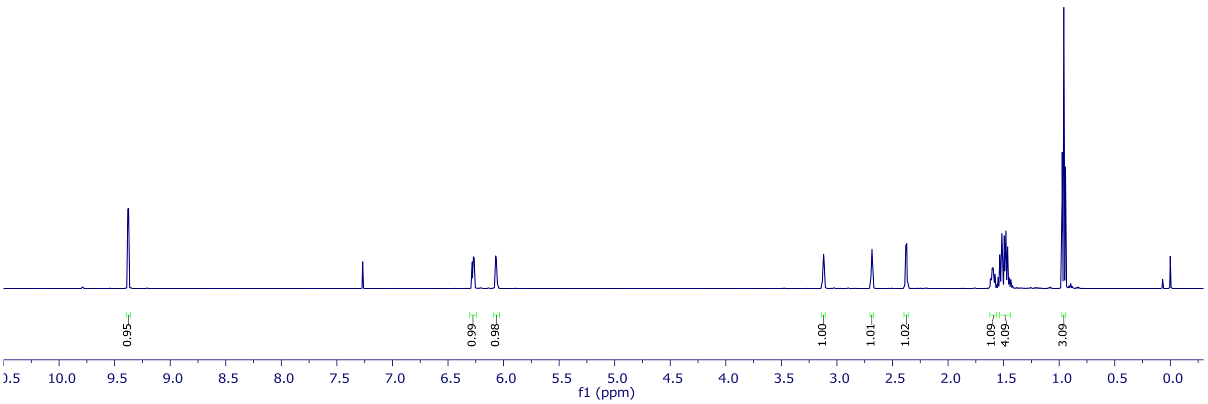


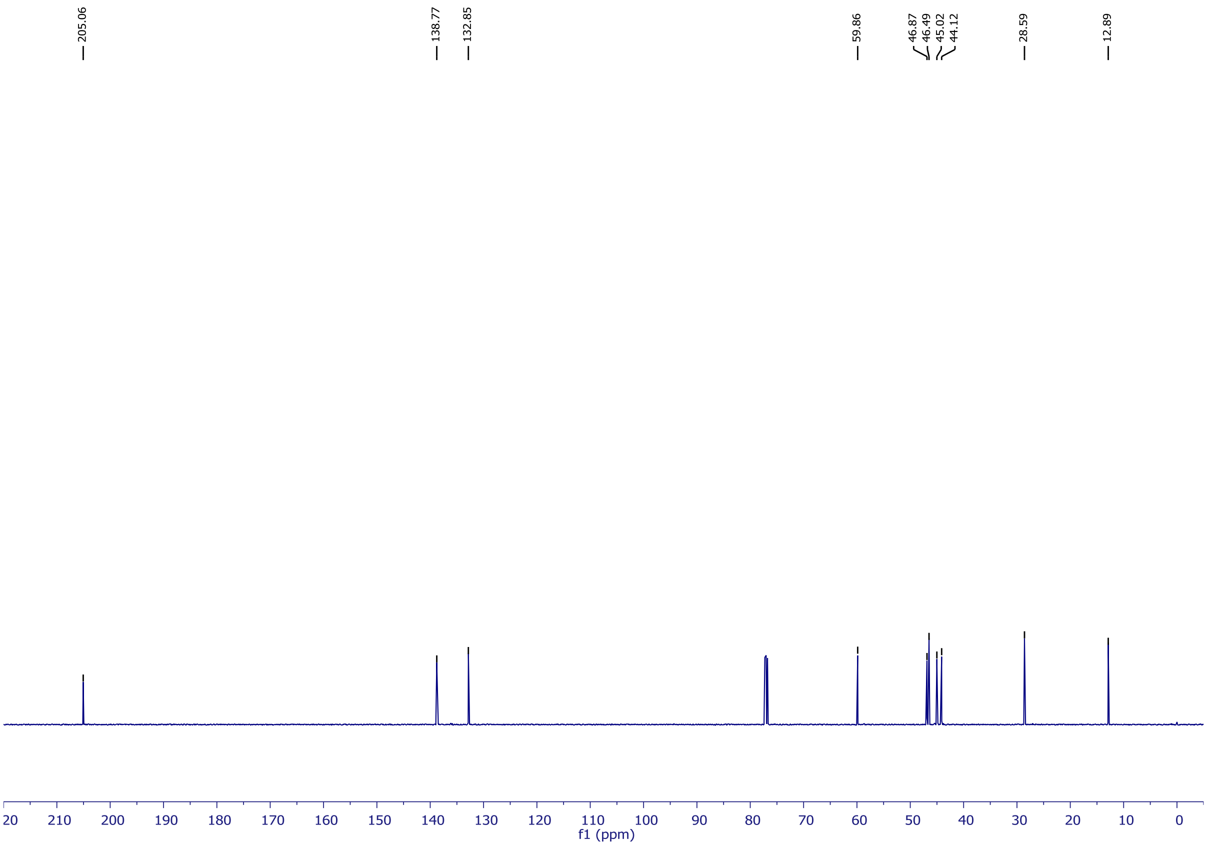


**Supplementary Figure 22.** $^{1}H$ and $^{13}C$ NMR spectra of **3k**

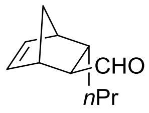


**3l** (*exo*)

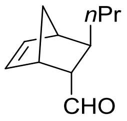


**3l** (*endo*)

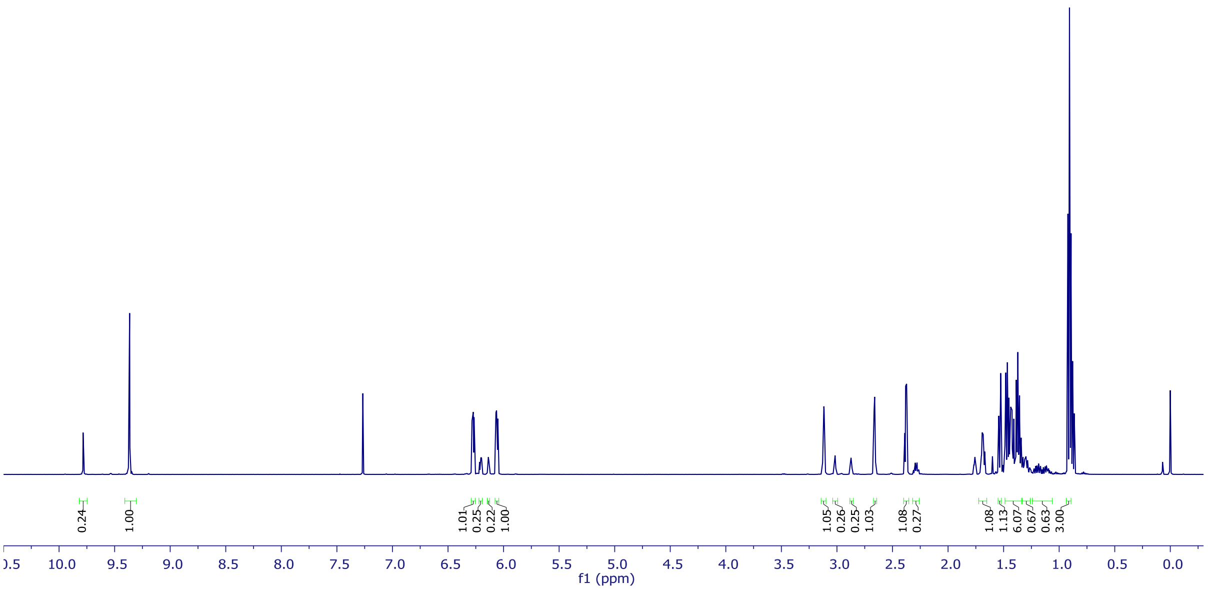


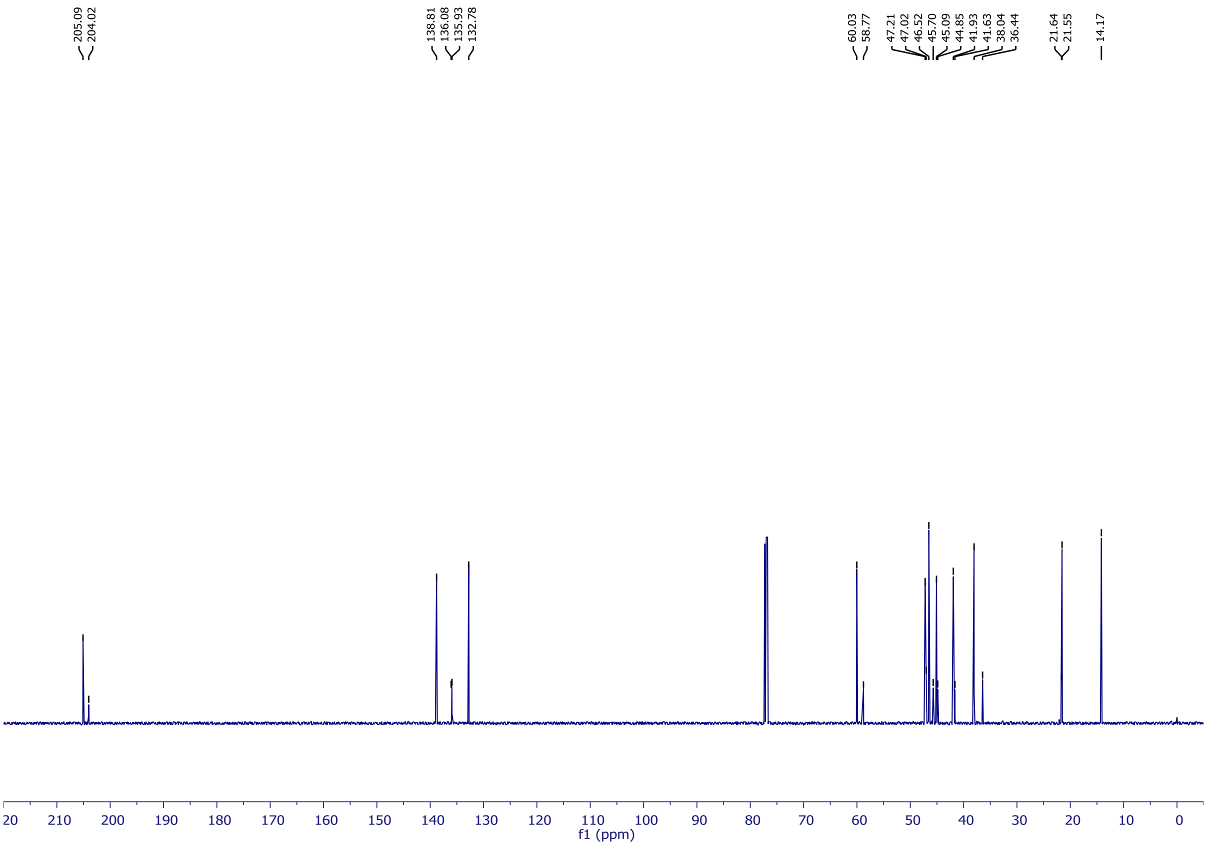


**Supplementary Figure 23.** $^{1}$H and $^{13}$C NMR spectra of **3l**

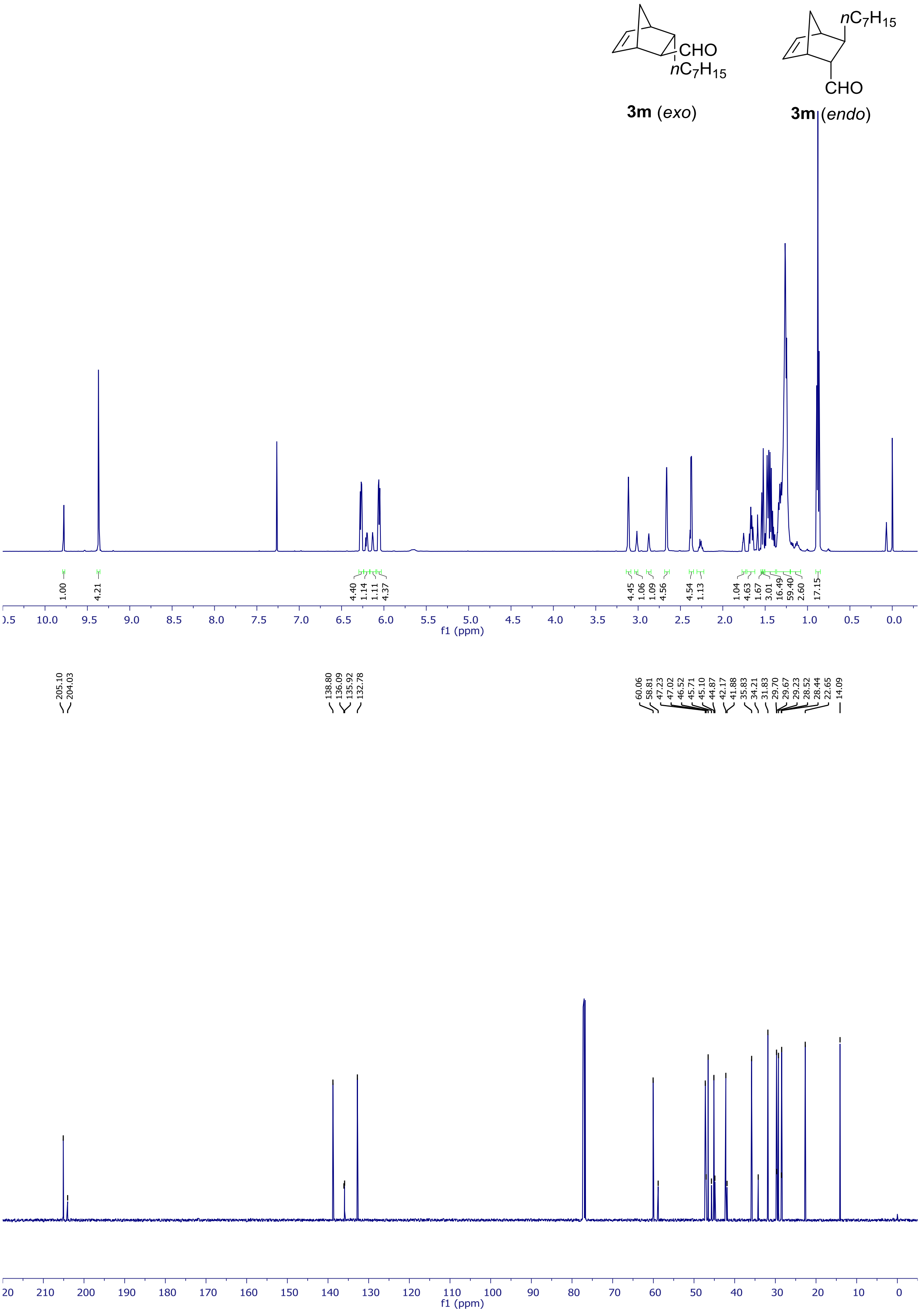


**Supplementary Figure 24.** $^1H$ and $^{13}C$ NMR spectra of **3m**

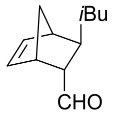


3n

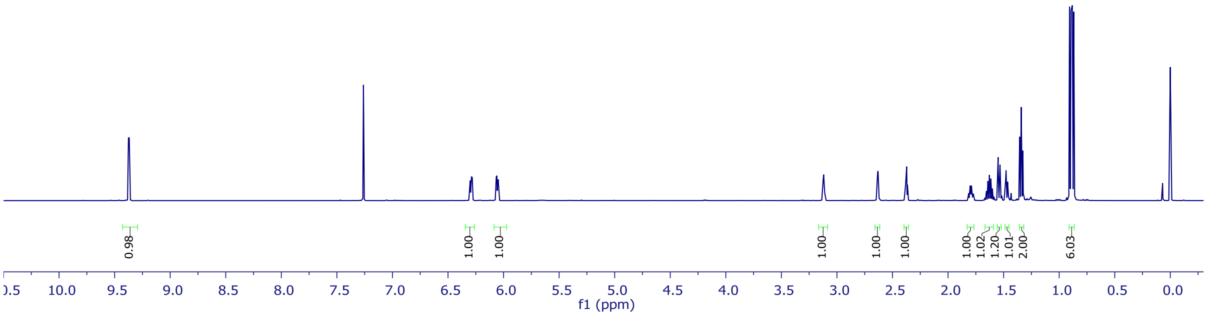

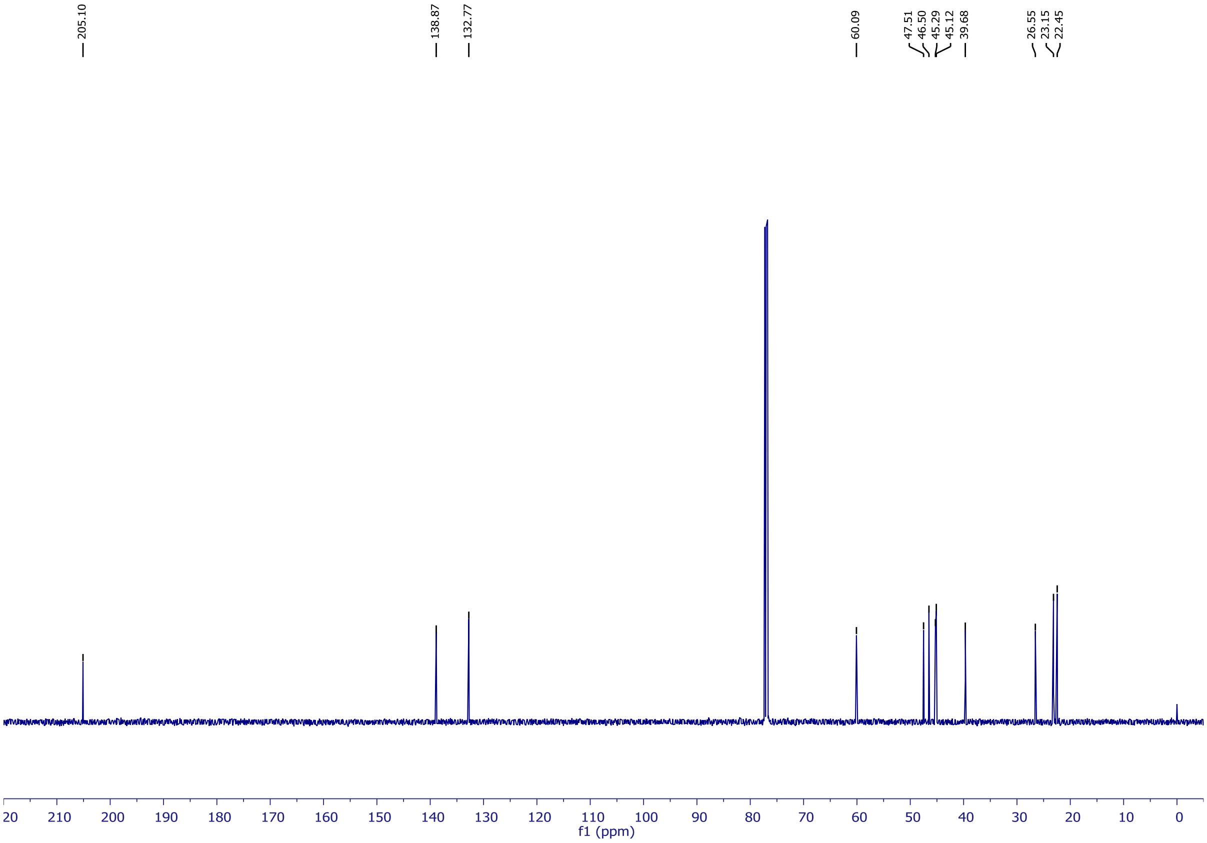

**Supplementary Figure 25.** $^{1}$H and $^{13}$C NMR spectra of **3n**

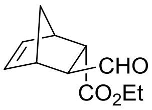


**3o** (*exo*)

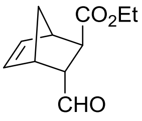


**3o** (*endo*)

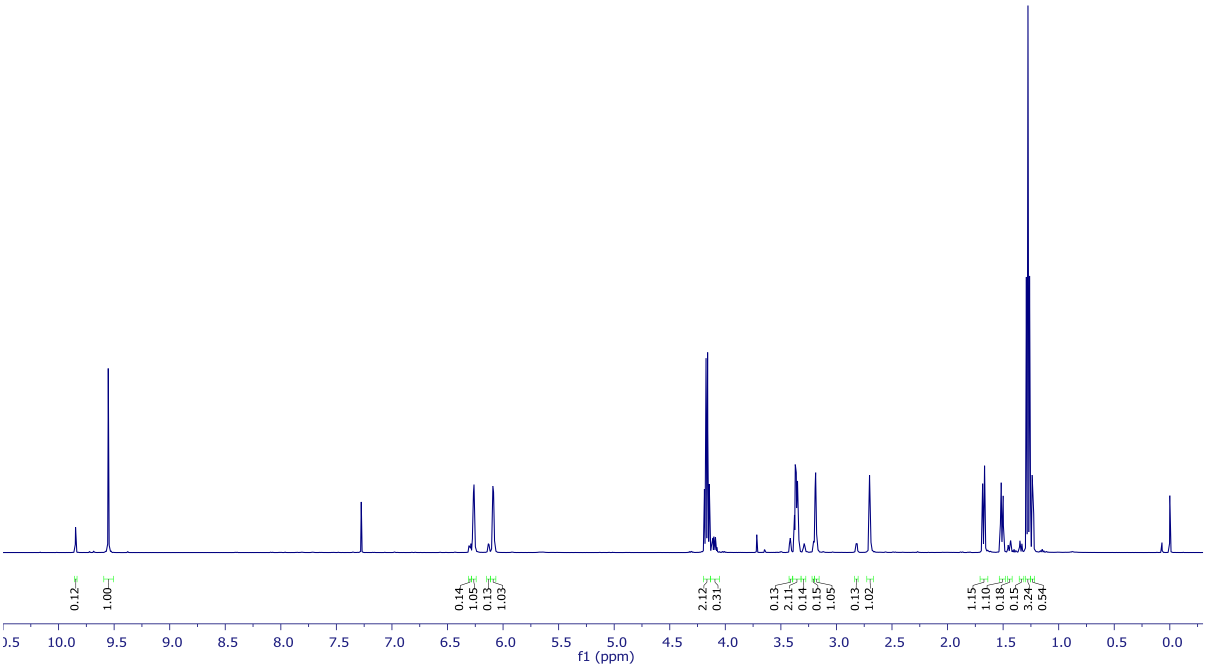


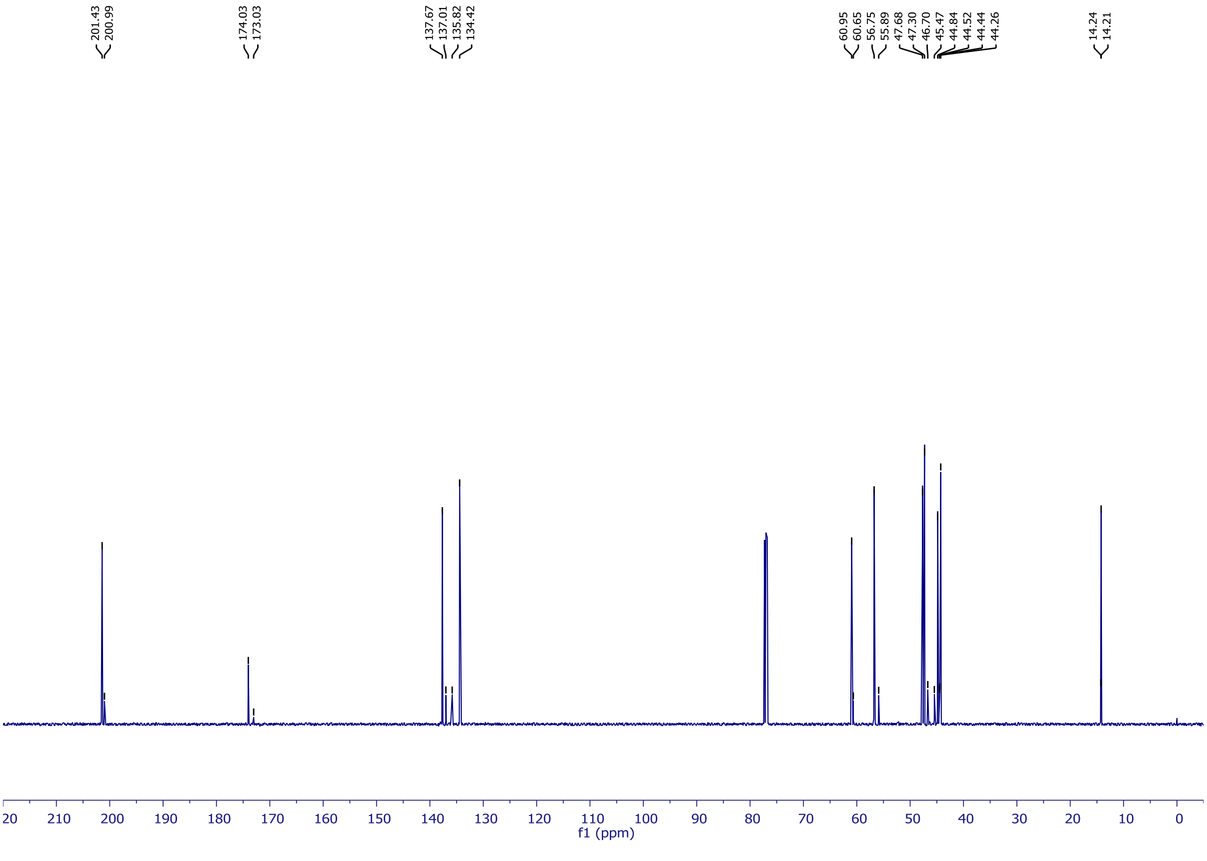


**Supplementary Figure 26.** $^{1}$H and $^{13}$C NMR spectra of **3o**

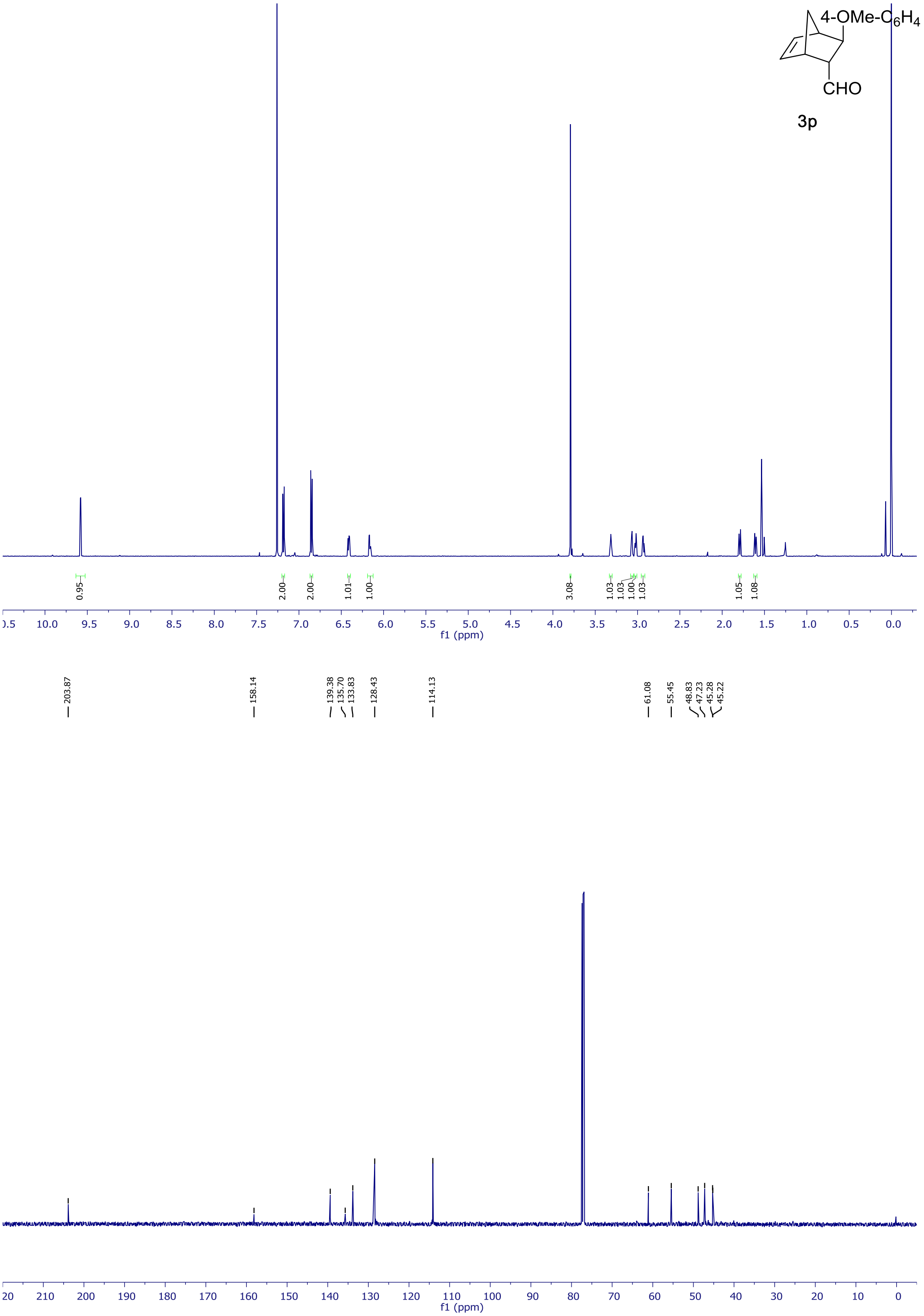


**Supplementary Figure 27.** $^{1}$H and $^{13}$C NMR spectra of **3p**

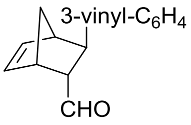


**3q**

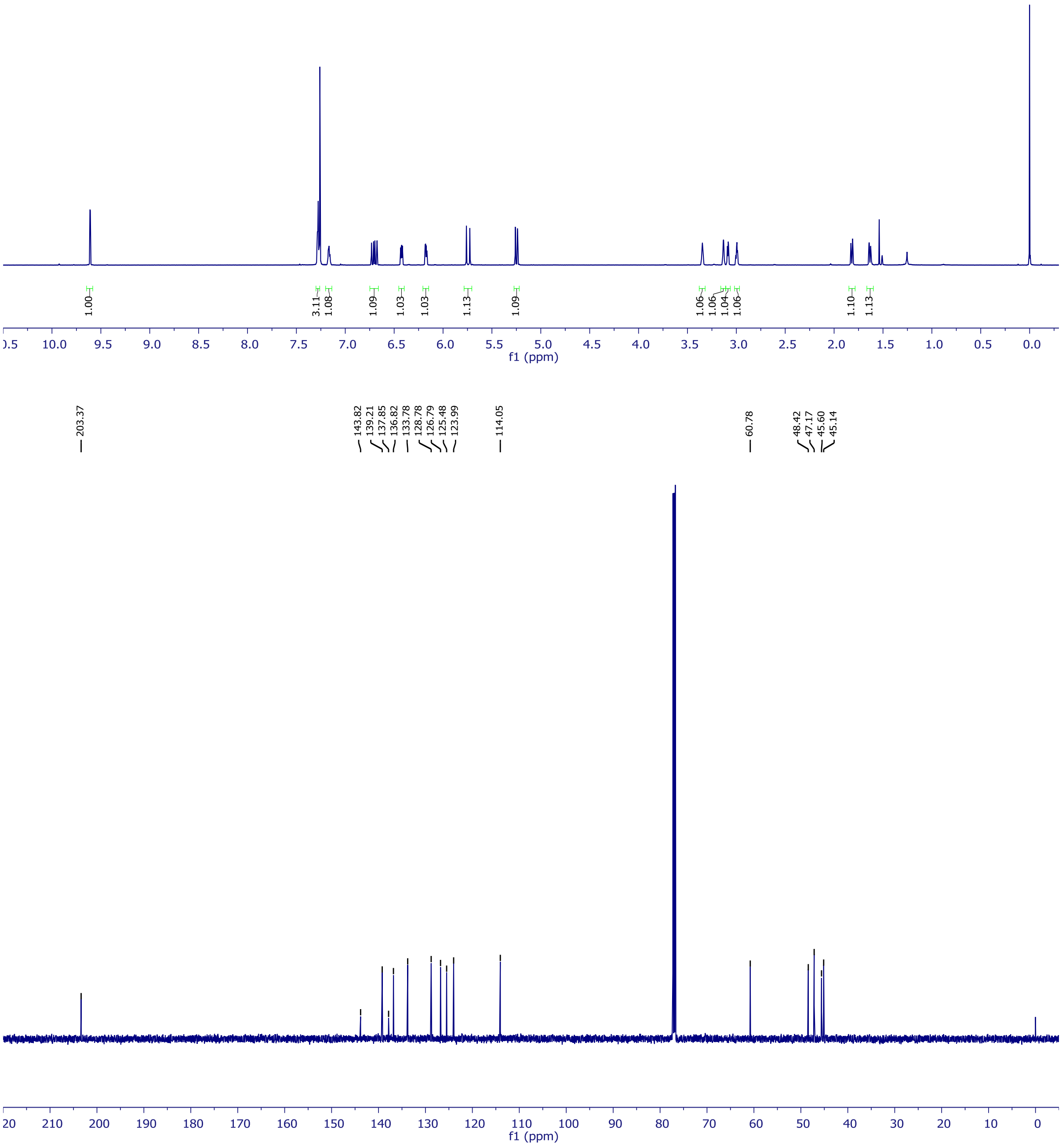


**Supplementary Figure 28.** $^{1}$H and $^{13}$C NMR spectra of **3q**

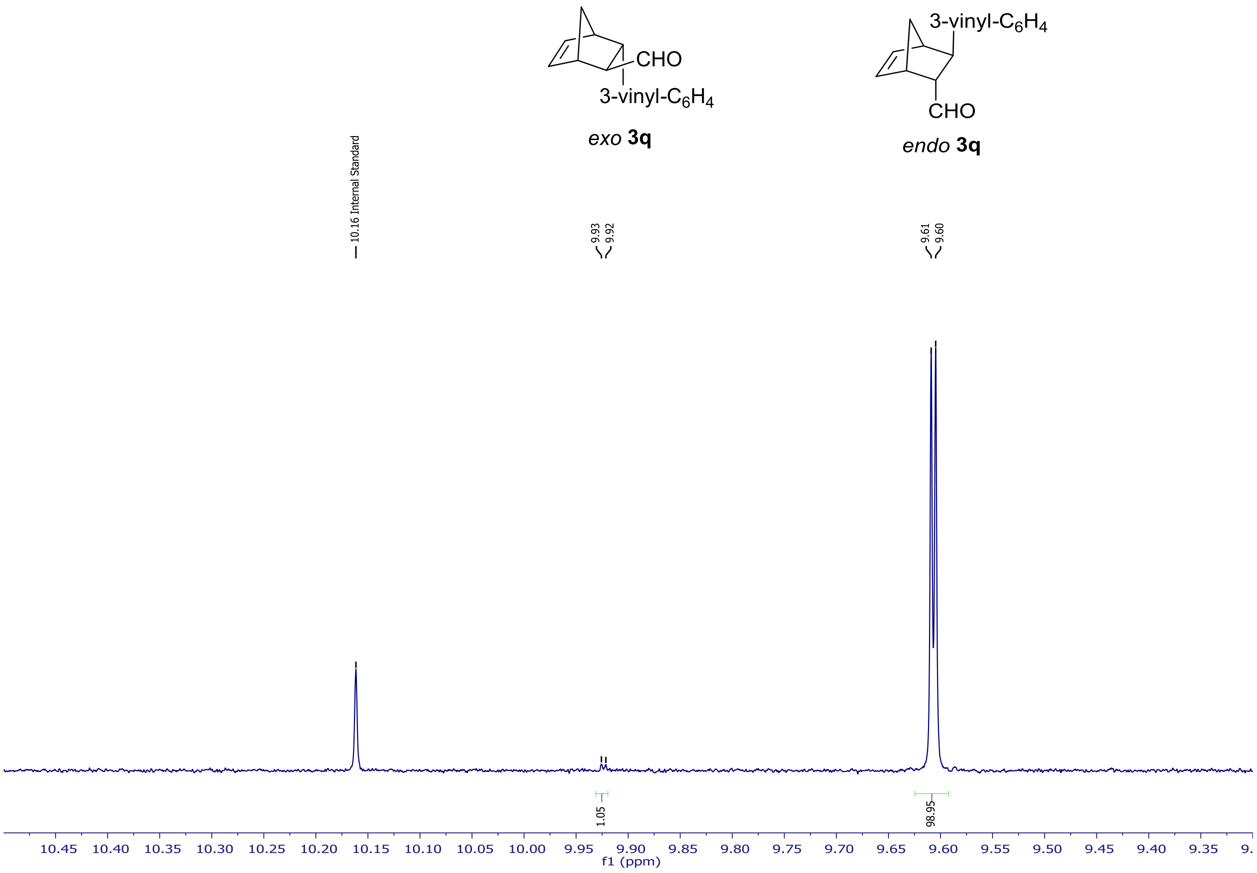


**Supplementary Figure 29.** $^1$H NMR spectrum of crude mixture of **3q**

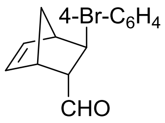


3r

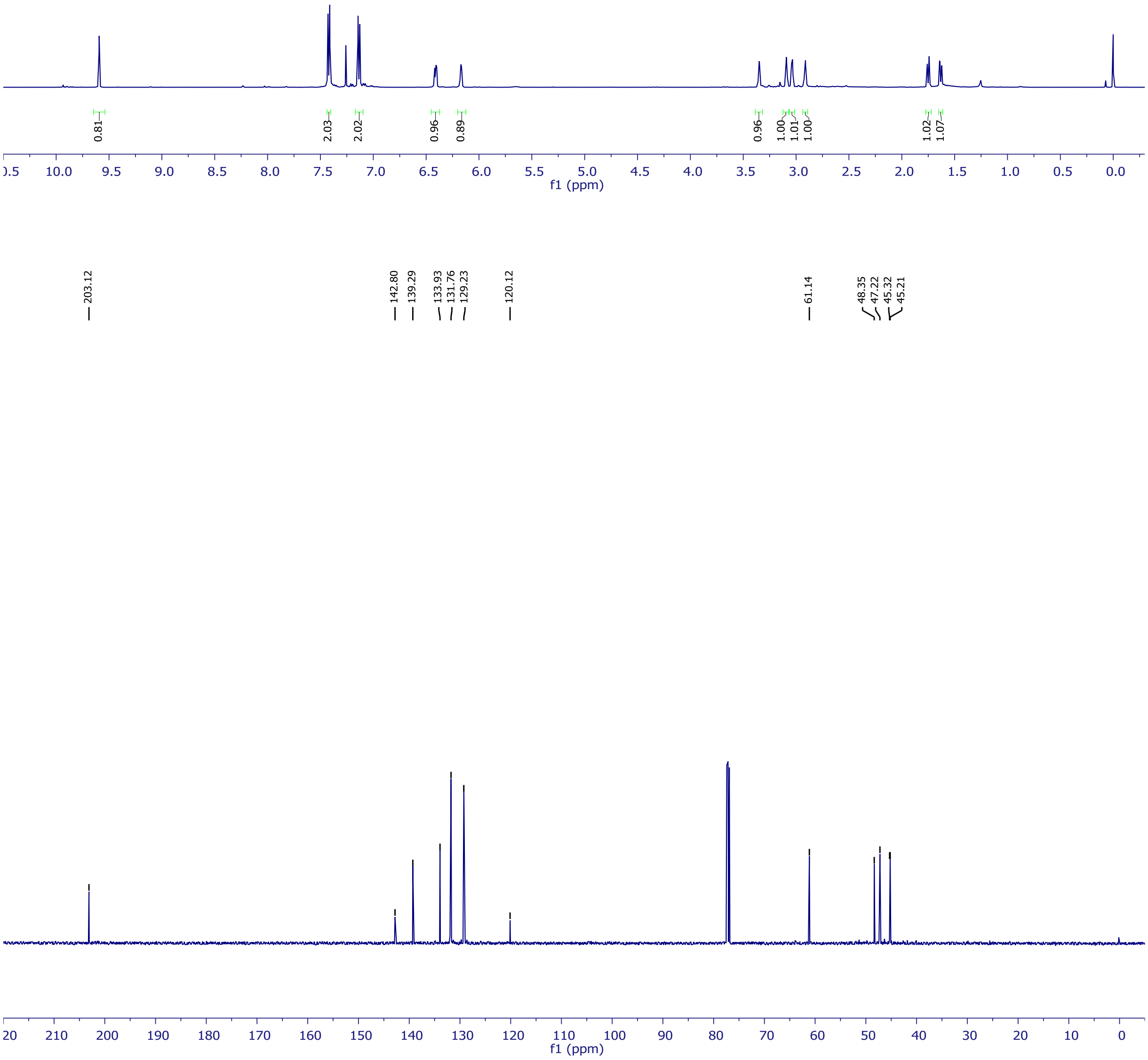


**Supplementary Figure 30.** $^{1}$H and $^{13}$C NMR spectra of **3r**

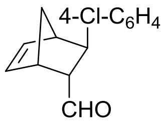


3s

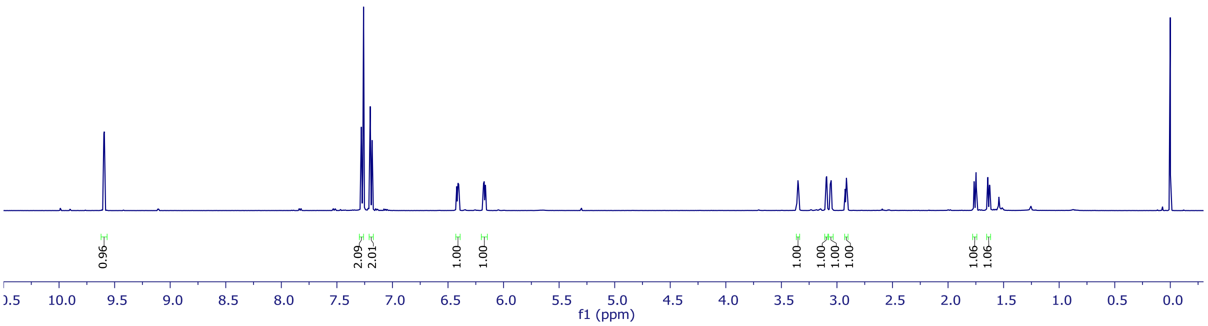


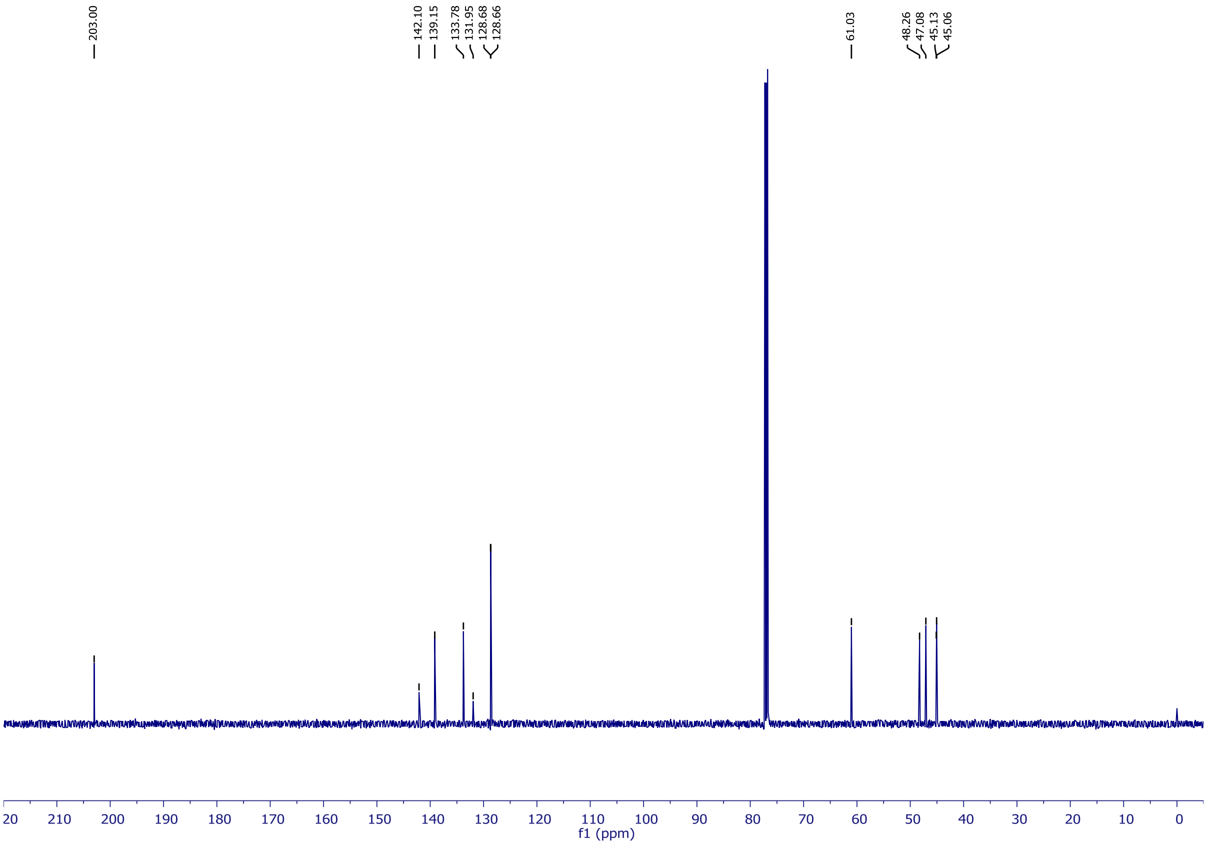


**Supplementary Figure 31.** $^{1}$H and $^{13}$C NMR spectra of **3s**

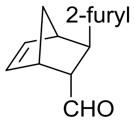


**3t**

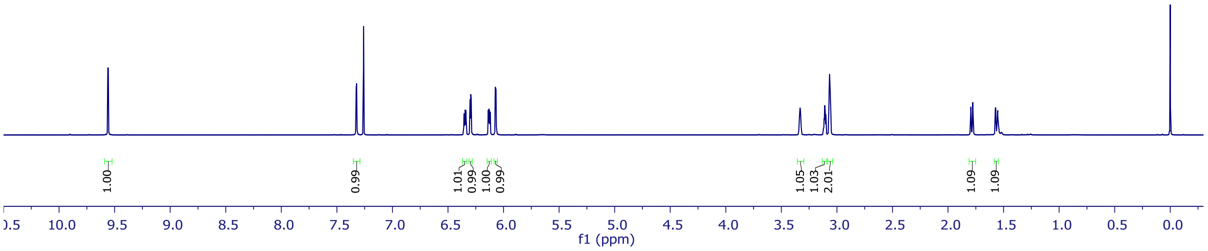


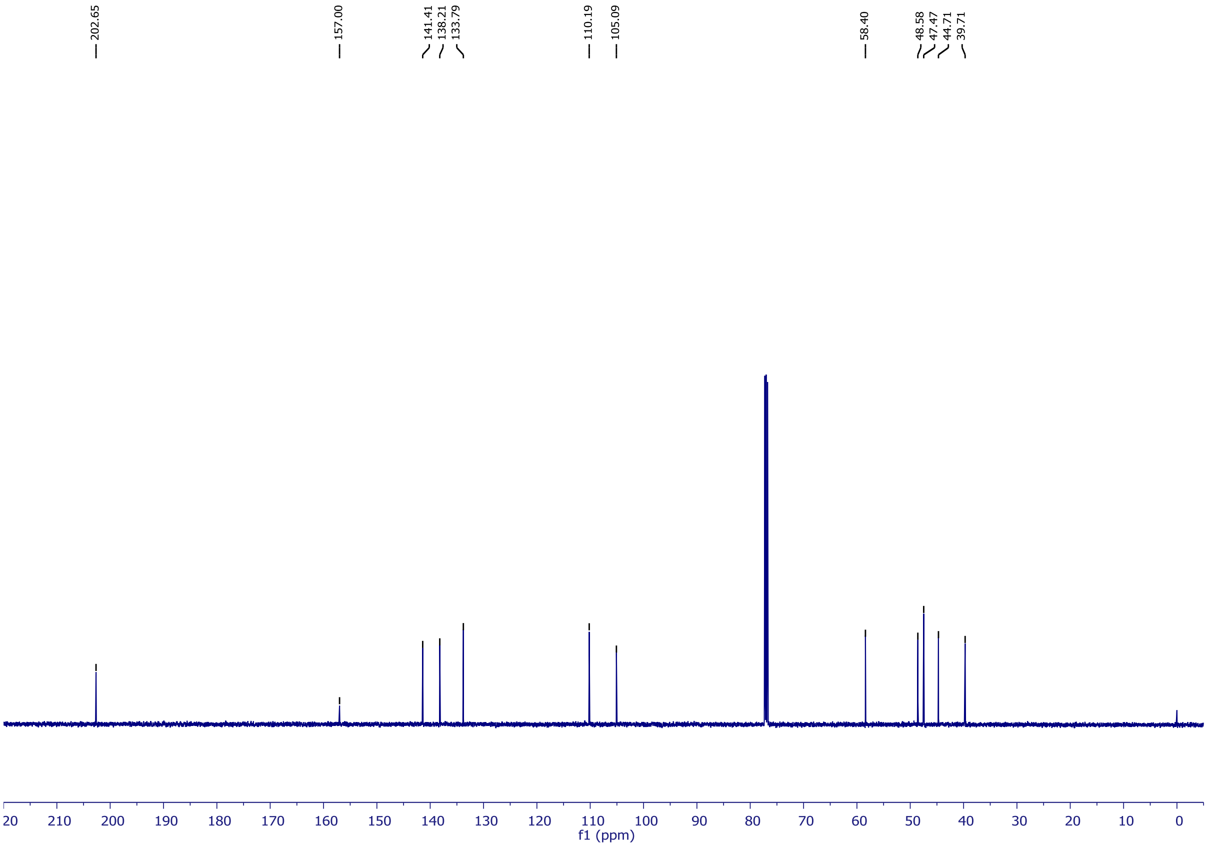


**Supplementary Figure 32.** $^{1}H$ and $^{13}C$ NMR spectra of **3t**

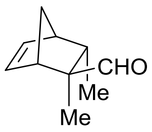


3u

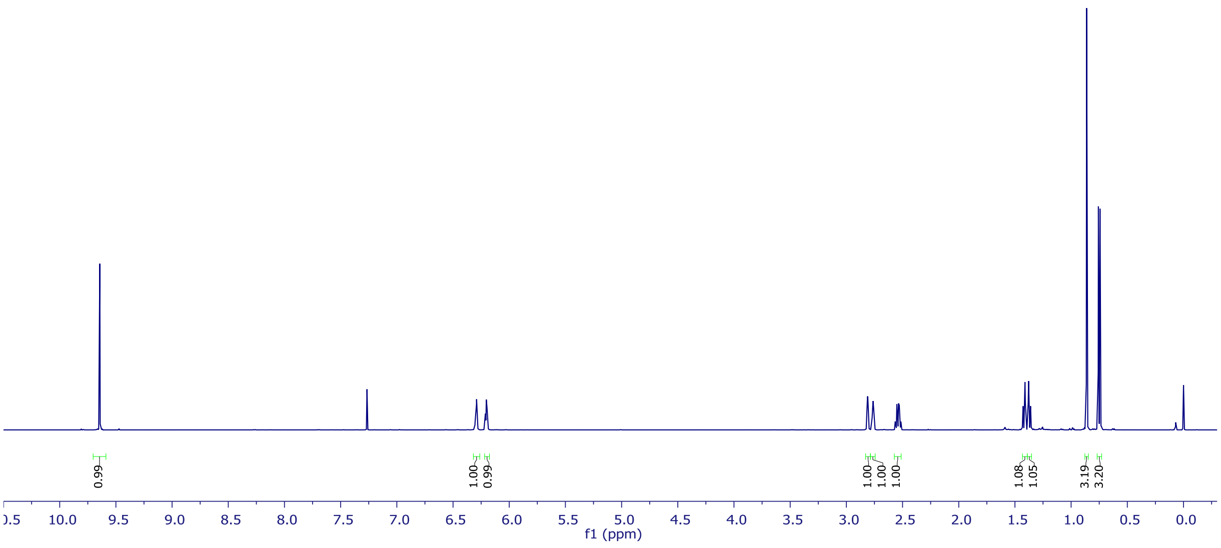


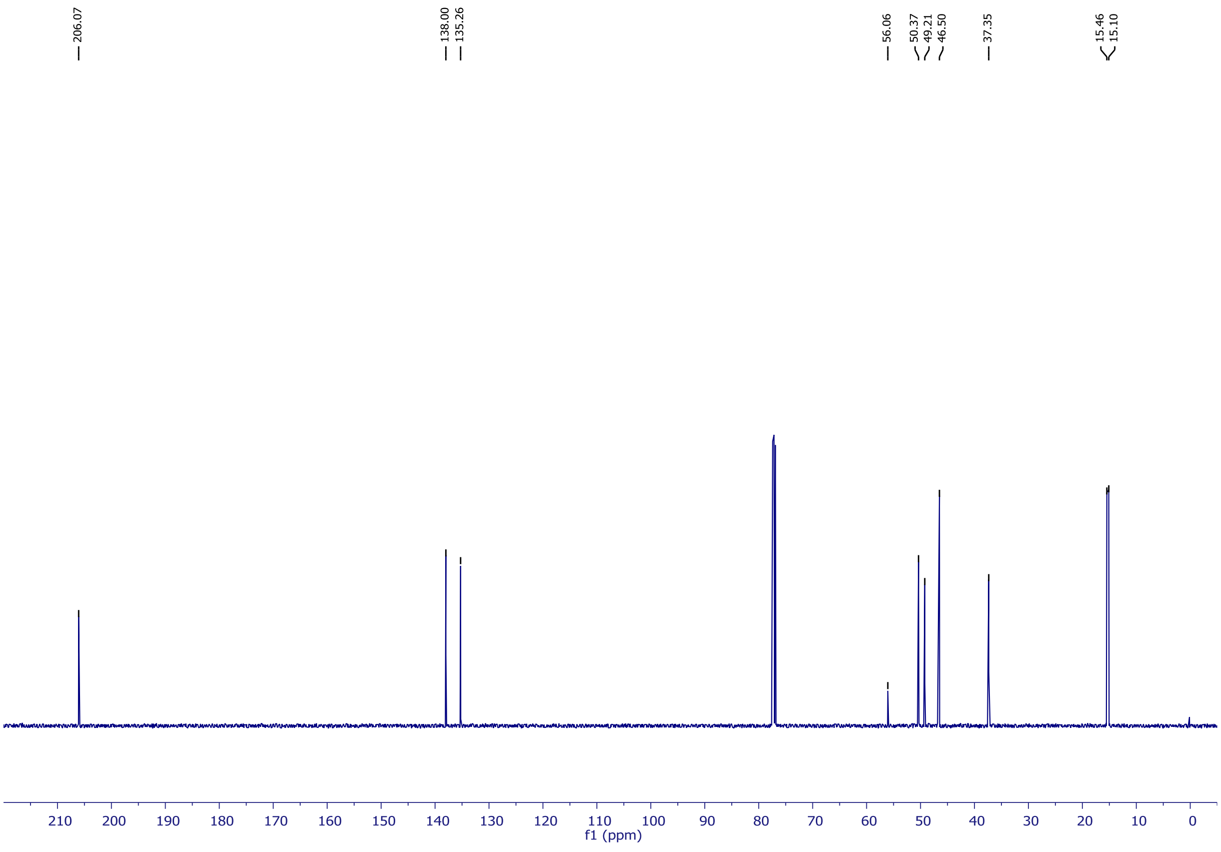


**Supplementary Figure 33.** $^{1}H$ and $^{13}C$ NMR spectra of **3u**

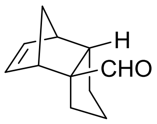


**3v**

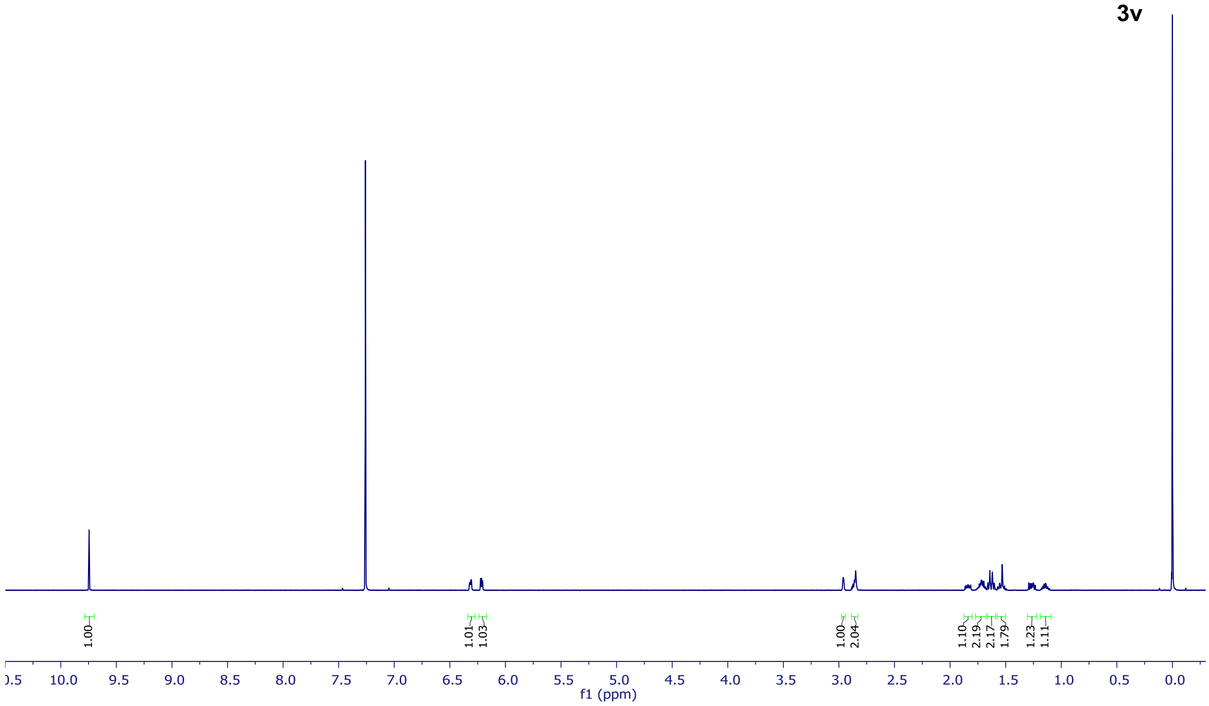


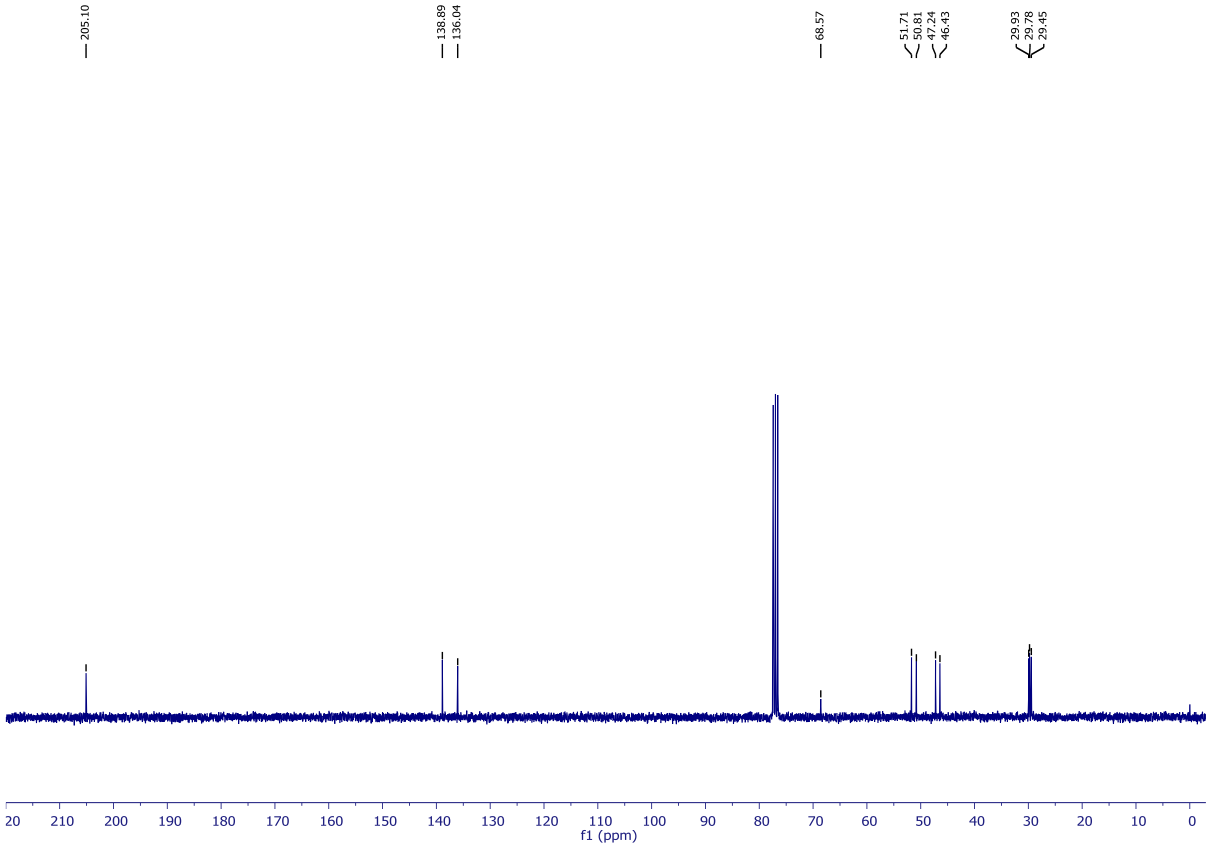


**Supplementary Figure 34.** $^{1}$H and $^{13}$C NMR spectra of **3v**

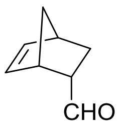


**3w**

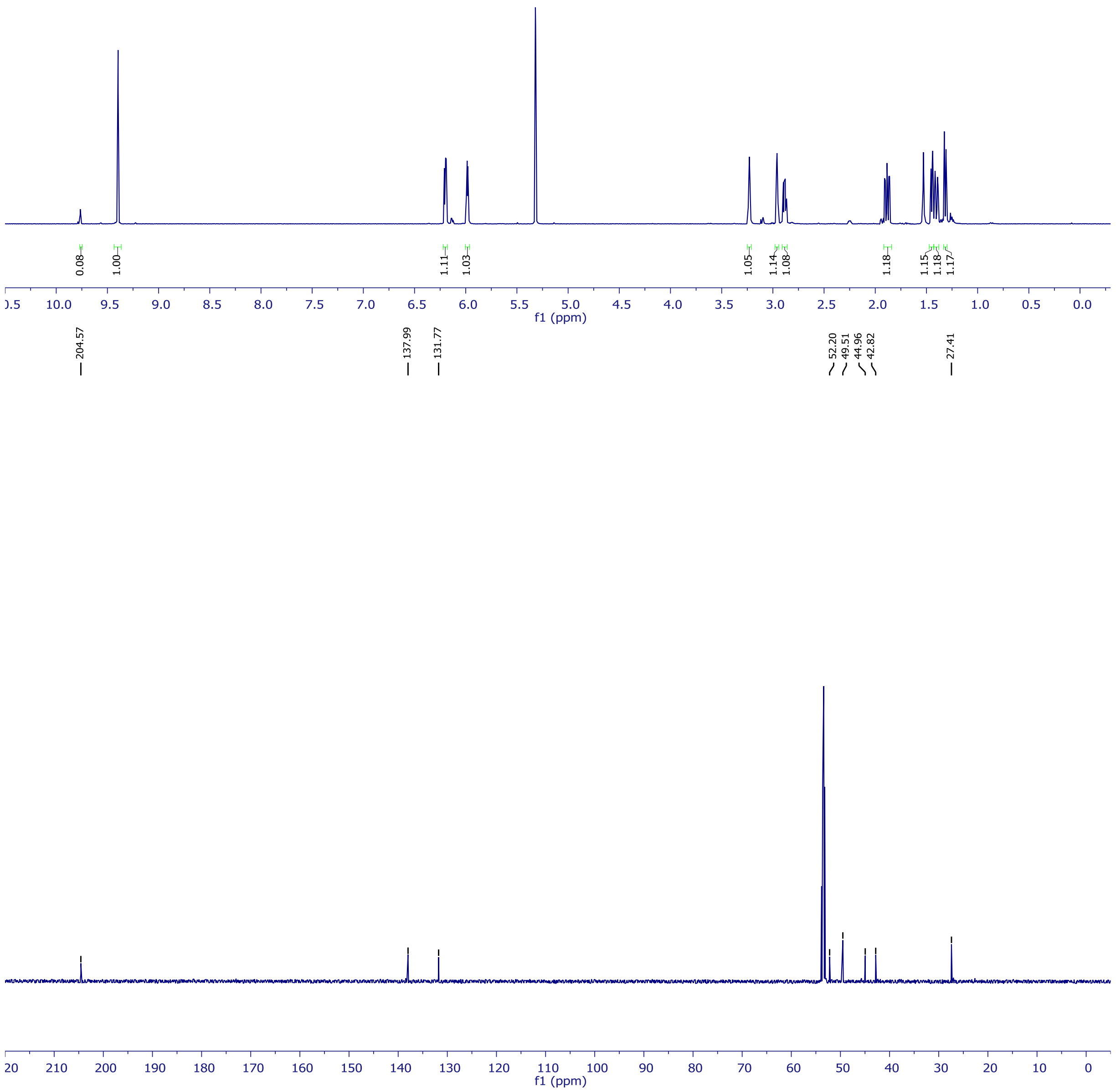


**Supplementary Figure 35.** $^{1}$H and $^{13}$C NMR spectra of **3w**

## **Supplementary Note 4.** GC and HPLC Traces of Products

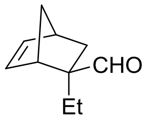


**3a**

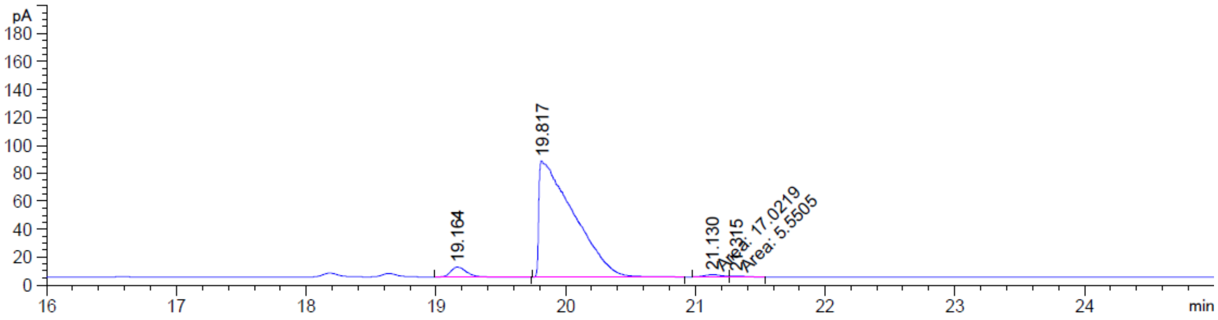


| Peak # | Name | Ret. Time/min | Area/% |
|---|---|---|---|
| 1 | exo 1 | 19.16 | 3.76 |
| 2 | exo 2 | 19.82 | 94.86 |
| 3 | endo 1 | 21.13 | 1.05 |
| 4 | endo 2 | 21.32 | 0.34 |

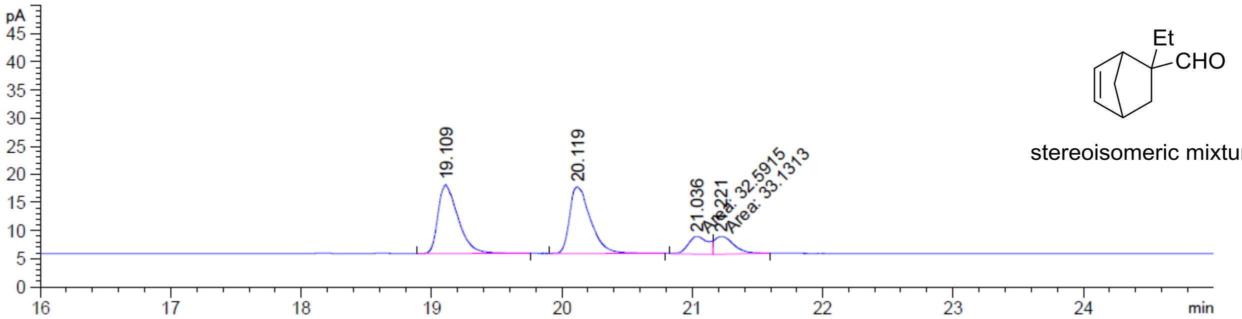


stereoisomeric mixture

| Peak # | Name | Ret. Time/min | Area/% |
|---|---|---|---|
| 1 | exo 1 | 19.11 | 39.99 |
| 2 | exo 2 | 20.12 | 40.12 |
| 3 | endo 1 | 21.04 | 9.86 |
| 4 | endo 2 | 21.22 | 10.03 |

**Supplementary Figure 36.** GC spectra of stereoiso-enriched (up) and stereoisomeric (down) mixture of **3a**

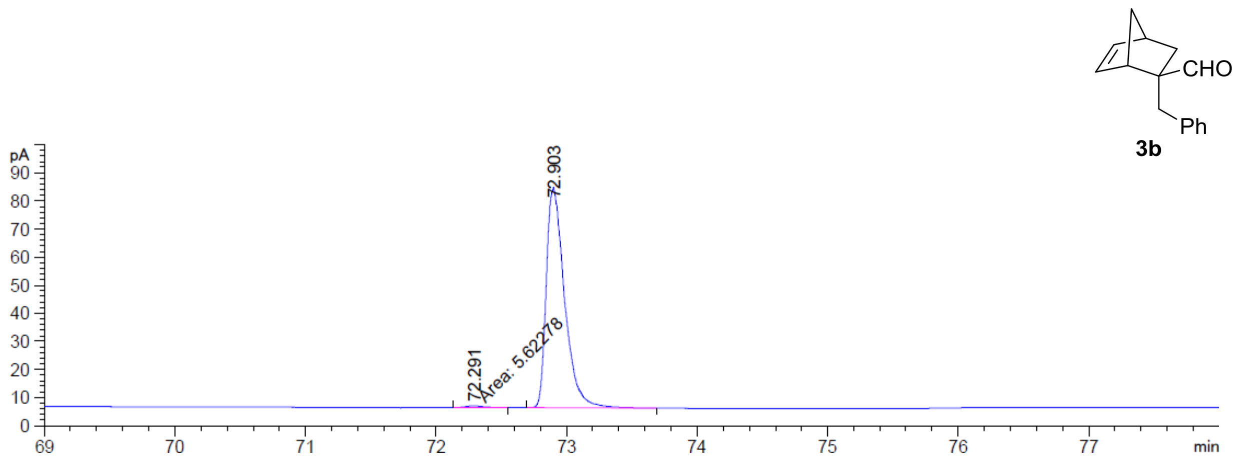


| Peak # | Name | Ret. Time/min | Area/% |
| --- | --- | --- | --- |
| 1 | exo 1 | 72.29 | 0.73 |
| 2 | exo 2 | 72.90 | 99.27 |

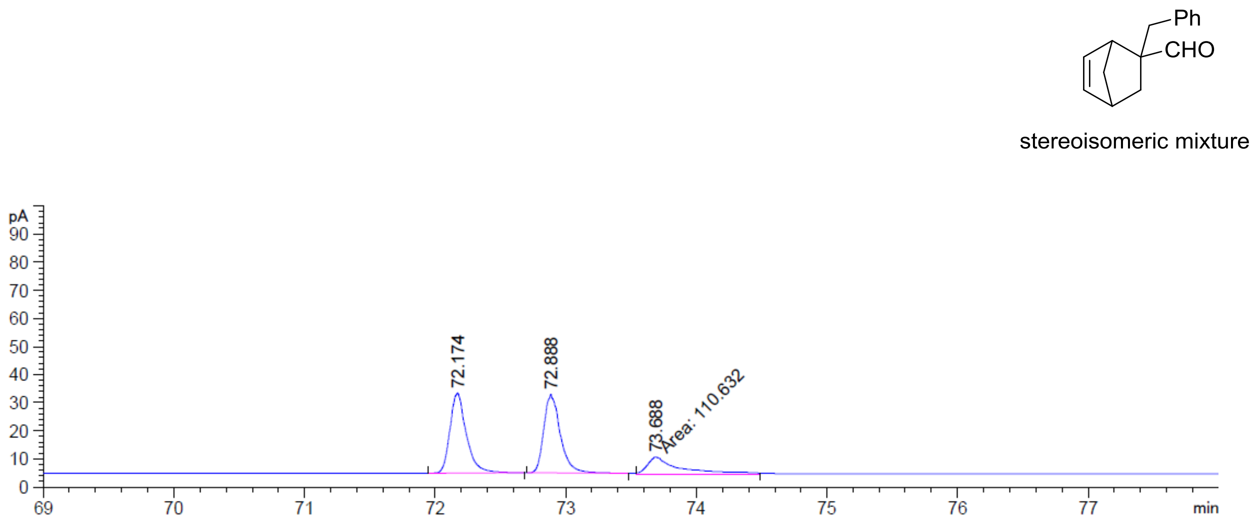


| Peak # | Name | Ret. Time/min | Area/% |
| --- | --- | --- | --- |
| 1 | exo 1 | 72.17 | 40.9 |
| 2 | exo 2 | 72.89 | 40.7 |
| 3 | endo 1 & 2 | 73.69 | 18.3 |

**Supplementary Figure 37.** GC spectra of stereoiso-enriched (up) and stereoisomeric (down) mixture of **3b**

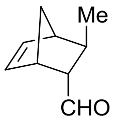


**3c**

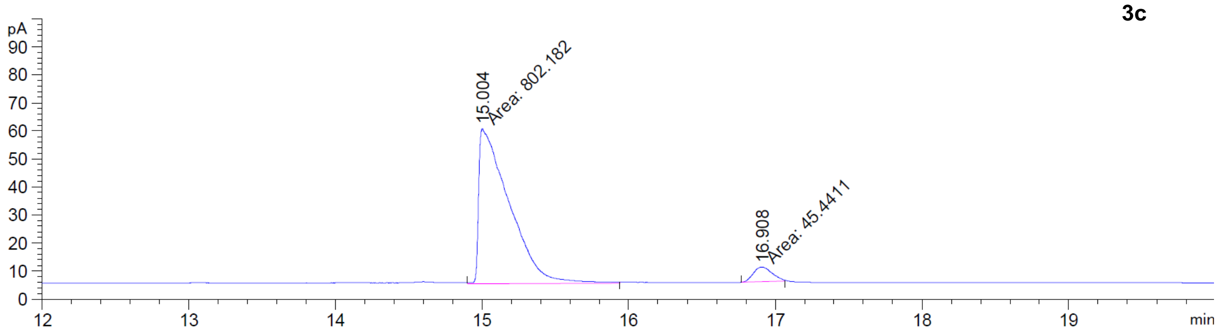


| Peak # | Name | Ret. Time/min | Area/% |
|---|---|---|---|
| 1 | endo 1 | 15.00 | 94.64 |
| 2 | endo 2 | 16.91 | 5.36 |

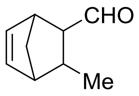


stereoisomeric mixture

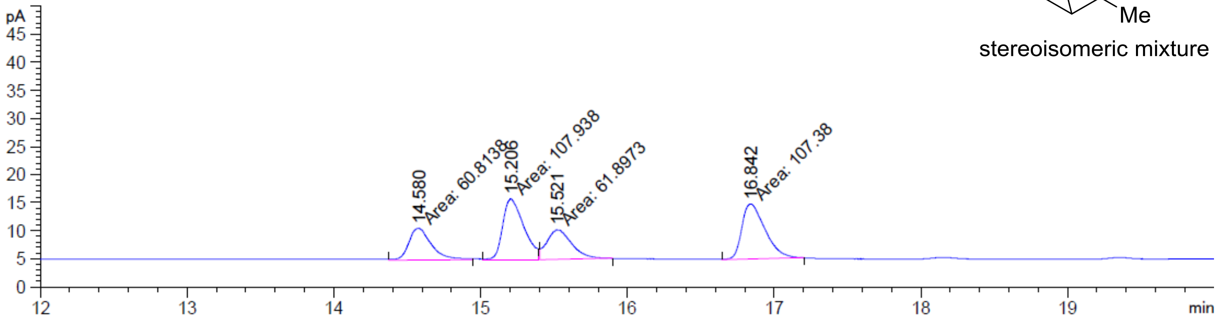


| Peak # | Name | Ret. Time/min | Area/% |
|---|---|---|---|
| 1 | exo 1 | 14.58 | 17.99 |
| 2 | endo 1 | 15.21 | 31.93 |
| 3 | exo 2 | 15.52 | 18.31 |
| 4 | endo 2 | 16.84 | 31.77 |

**Supplementary Figure 38.** GC spectra of stereoiso-enriched (up) and stereoisomeric (down) mixture of **3c**

**Supplementary Figure 39.** Determination of absolute stereochemistry of product **3c**

**3c** (toluene reflux, stereoisomeric mixture)

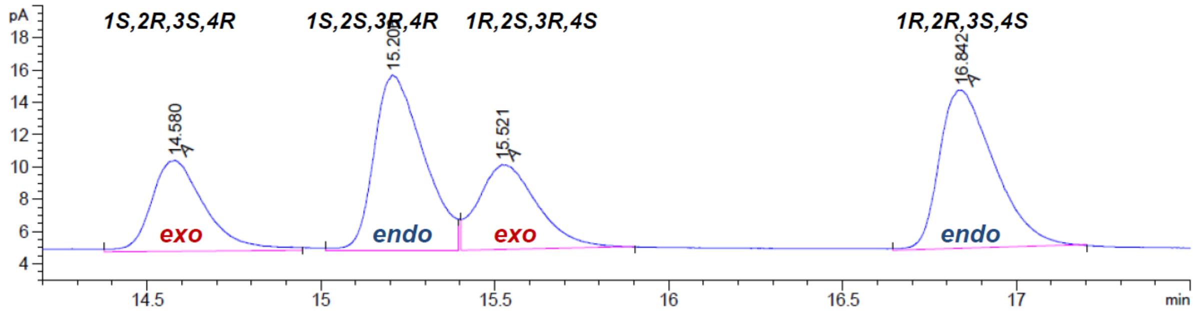


**3c** (10 mol% (*R*)-5-benzyl-2,2,3-trimethylimidazolidin-4-one hydrochloride, RT)

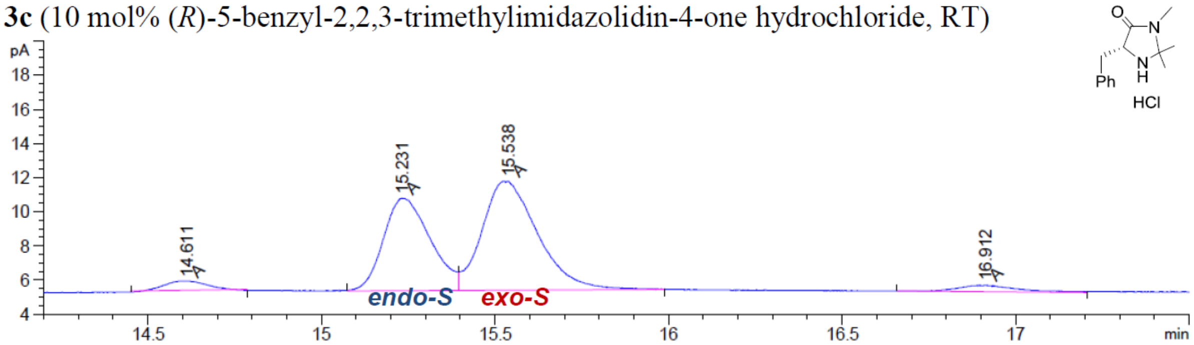


**3c** (10 mol% (*S*)-5-benzyl-2,2,3-trimethylimidazolidin-4-one hydrochloride, RT)

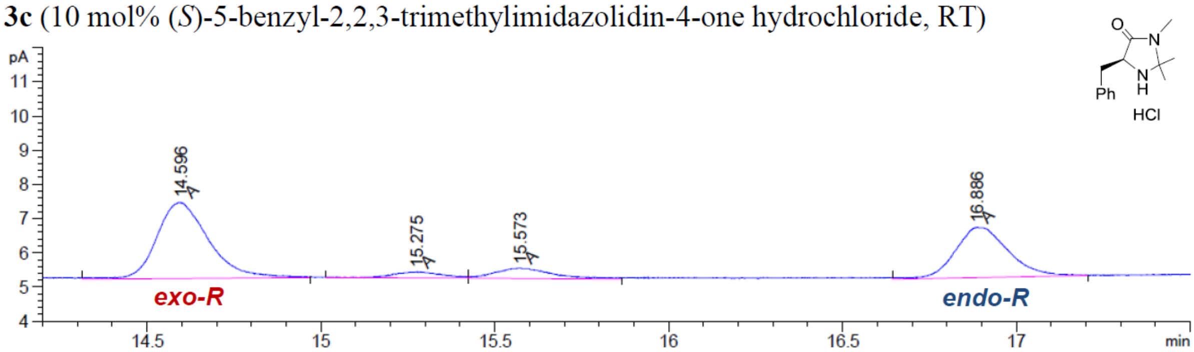


**3c** (1 mol% **4h**, –100 °C)

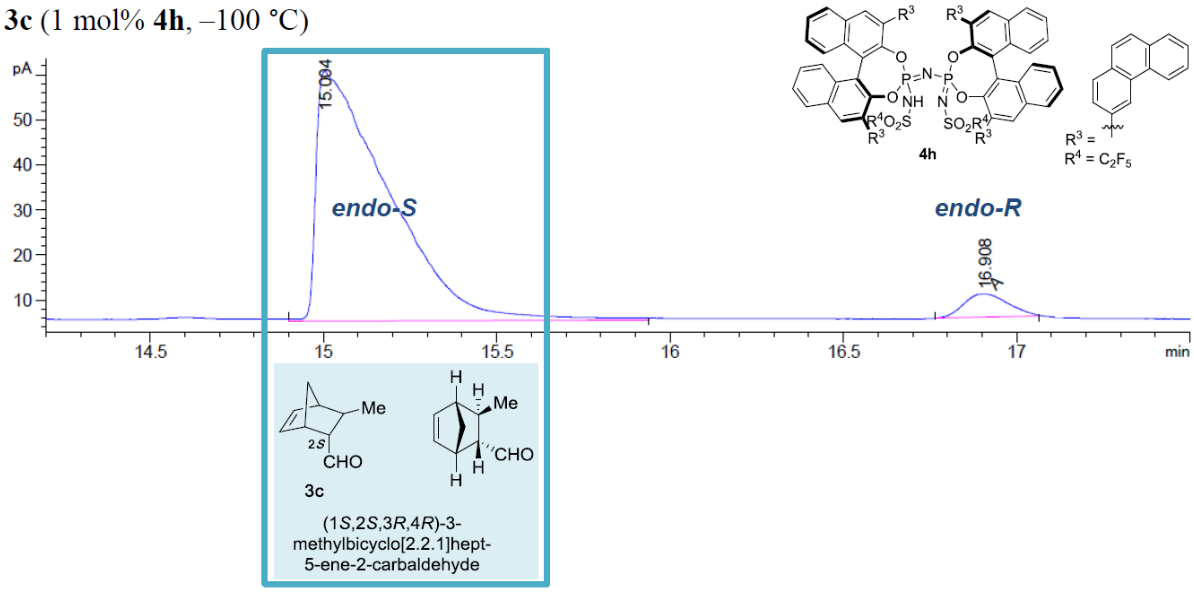

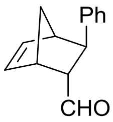


**3d**

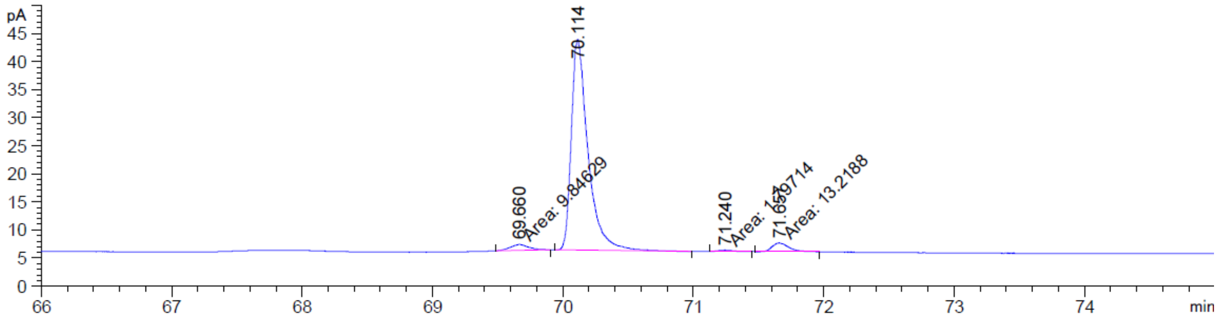


| Peak # | Name | Ret. Time/min | Area/% |
|---|---|---|---|
| 1 | exo 1 | 69.66 | 2.74 |
| 2 | endo 1 | 70.11 | 93.08 |
| 3 | exo 2 | 71.24 | 0.50 |
| 4 | endo 2 | 71.68 | 3.68 |

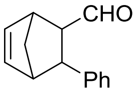


stereoisomeric mixture

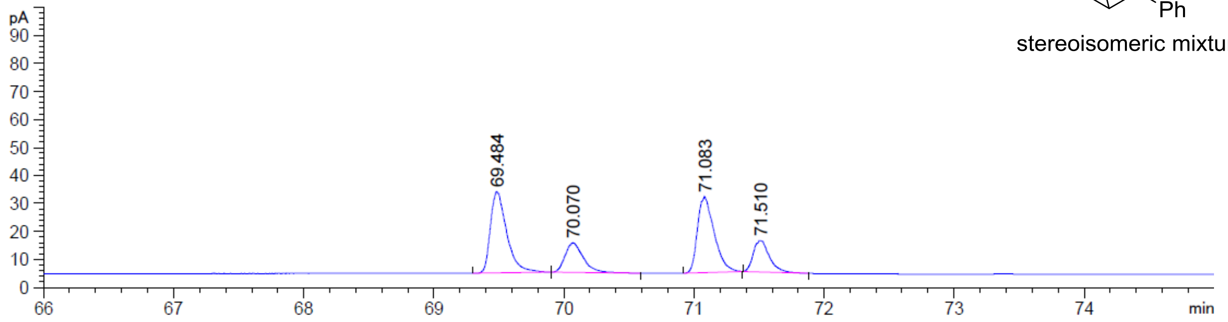


| Peak # | Name | Ret. Time/min | Area/% |
|---|---|---|---|
| 1 | exo 1 | 69.48 | 36.54 |
| 2 | endo 1 | 70.07 | 14.77 |
| 3 | exo 2 | 71.08 | 34.80 |
| 4 | endo 2 | 71.51 | 13.89 |

**Supplementary Figure 40.** GC spectra of stereoiso-enriched (up) and stereoisomeric (down) mixture of **3d**

**Supplementary Figure 41.** Determination of absolute stereochemistry of product **3d**

**3d** (toluene reflux, stereoisomeric mixture)

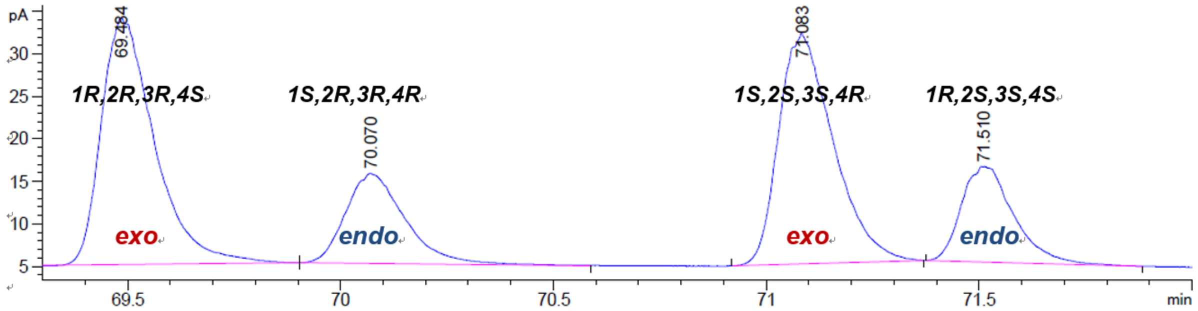


**3d** (10 mol% (*R*)-5-benzyl-2,2,3-trimethylimidazolidin-4-one hydrochloride, RT)

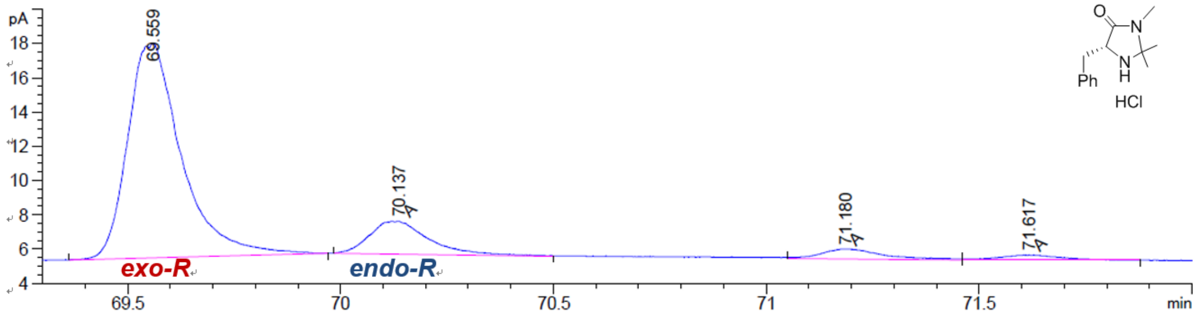


**3d** (10 mol% (*S*)-5-benzyl-2,2,3-trimethylimidazolidin-4-one hydrochloride, RT)

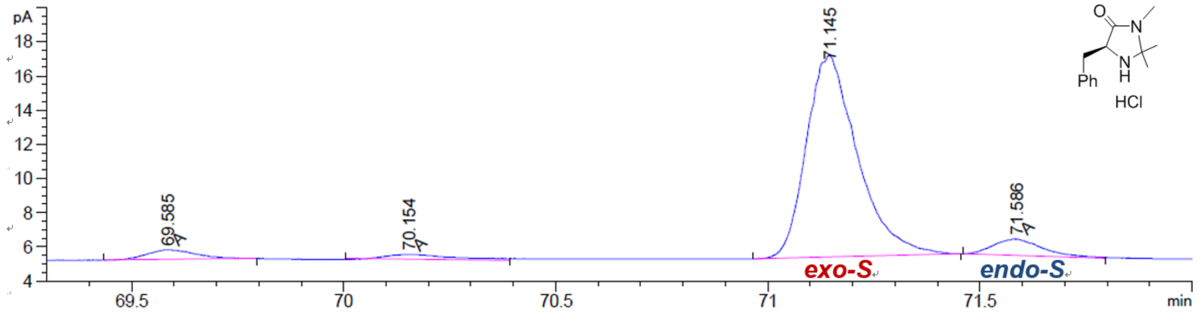


**3d** (1 mol% **4i**, –100 °C)

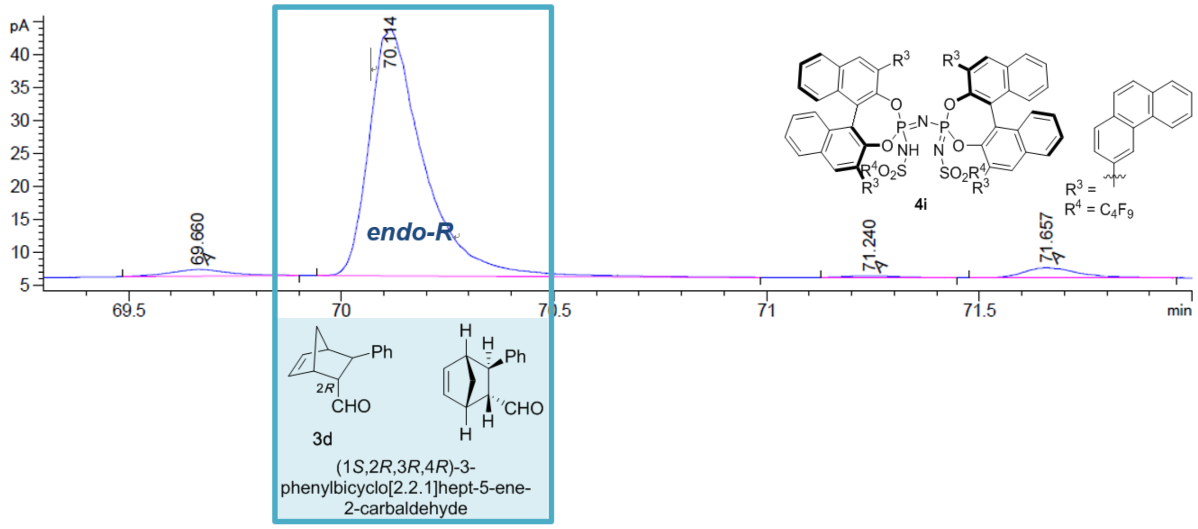

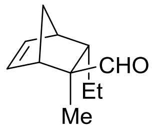


3e

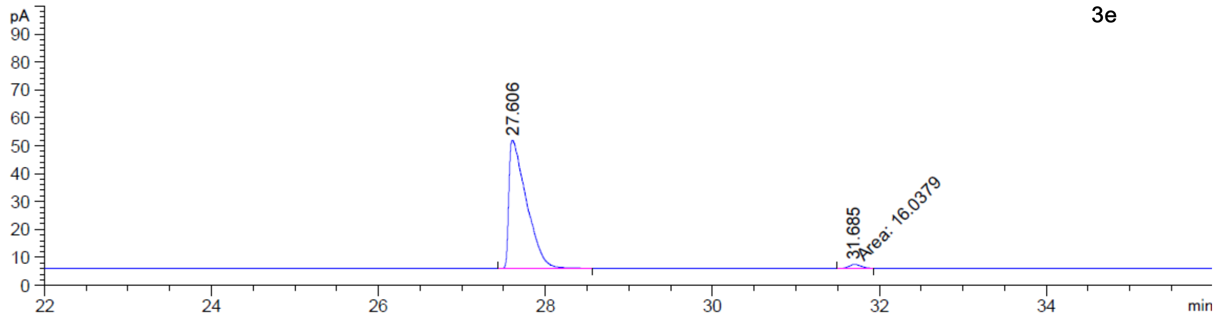


| Peak # | Name | Ret. Time/min | Area/% |
|---|---|---|---|
| 1 | exo 1 | 27.61 | 97.66 |
| 2 | exo 2 | 31.69 | 2.34 |

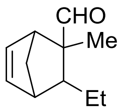


stereoisomeric mixture

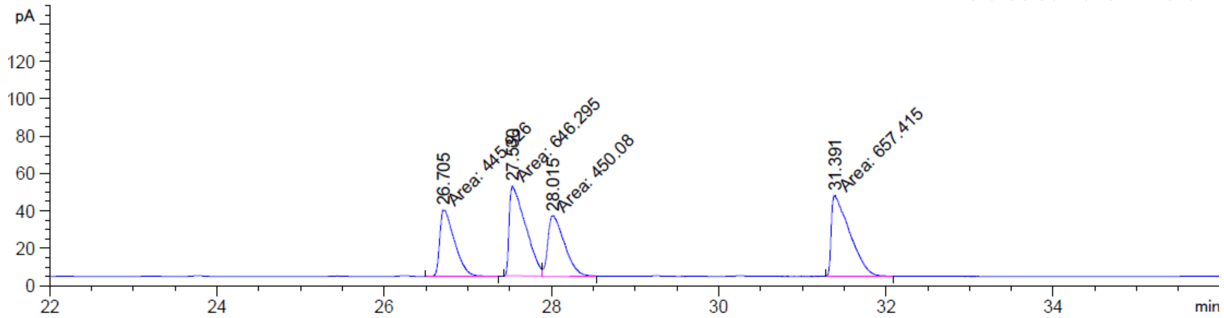


| Peak # | Name | Ret. Time/min | Area/% |
|---|---|---|---|
| 1 | endo 1 | 26.71 | 20.27 |
| 2 | exo 1 | 27.53 | 29.38 |
| 3 | eneo 2 | 28.02 | 20.46 |
| 4 | exo 2 | 31.39 | 29.89 |

**Supplementary Figure 42.** GC spectra of stereoiso-enriched (up) and stereoisomeric (down) mixture of **3e**

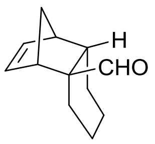


3f

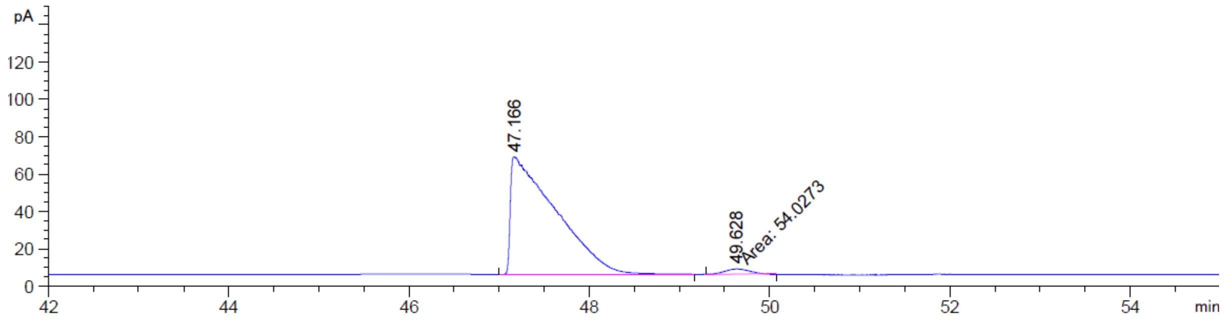


| Peak # | Name | Ret. Time/min | Area/% |
|---|---|---|---|
| 1 | exo 1 | 47.17 | 97.60 |
| 2 | exo 2 | 49.63 | 2.40 |

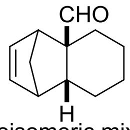


stereoisomeric mixture

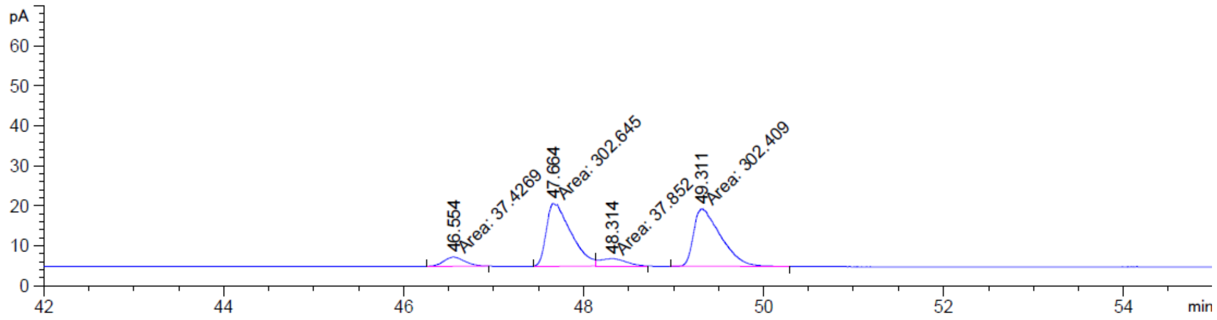


| Peak # | Name | Ret. Time/min | Area/% |
|---|---|---|---|
| 1 | endo 1 | 46.55 | 5.50 |
| 2 | exo 1 | 47.66 | 44.48 |
| 3 | eneo 2 | 48.31 | 5.56 |
| 4 | exo 2 | 49.31 | 44.45 |

**Supplementary Figure 43.** GC spectra of stereoiso-enriched (up) and stereoisomeric (down) mixture of **3f**

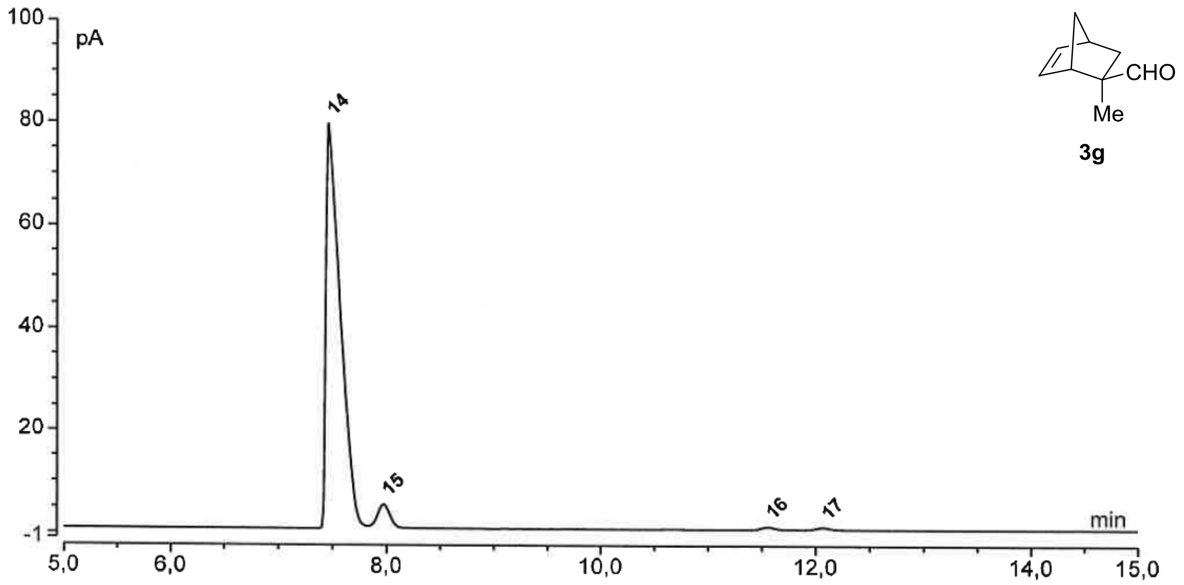


| Peak # | Name | Ret. Time/min | Area/% |
|---|---|---|---|
| 14 | exo 1 | 7.46 | 93.95 |
| 15 | exo 2 | 7.97 | 4.97 |
| 16 | endo 1 | 11.55 | 0.58 |
| 17 | endo 2 | 12.07 | 0.50 |

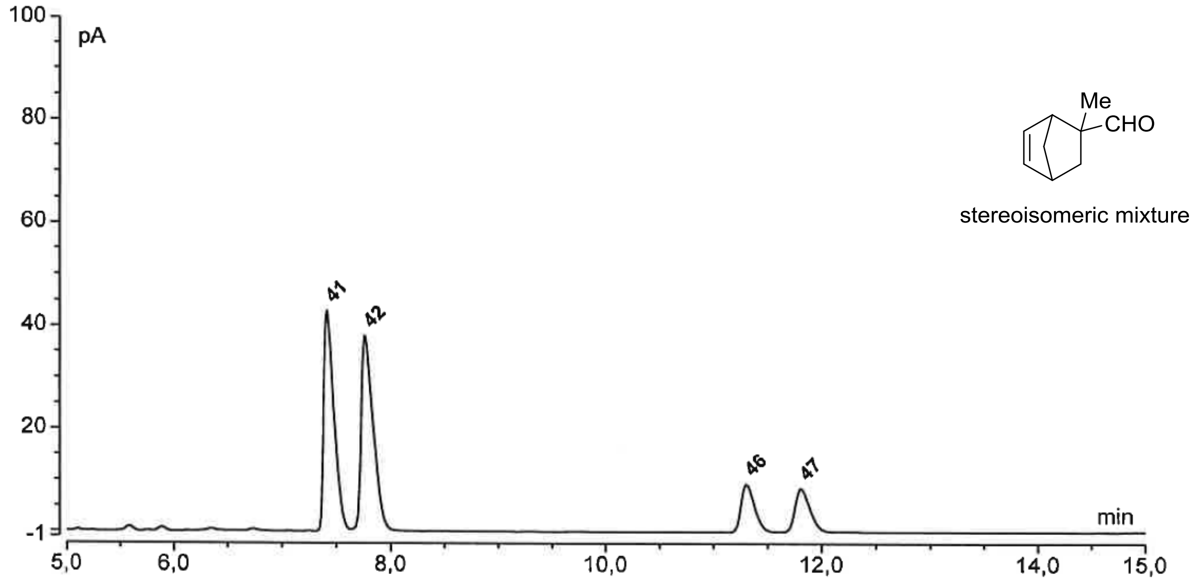


| Peak # | Name | Ret. Time/min | Area/% |
|---|---|---|---|
| 41 | exo 1 | 7.41 | 38.60 |
| 42 | exo 2 | 7.76 | 39.32 |
| 43 | endo 1 | 11.30 | 11.02 |
| 44 | endo 2 | 11.81 | 11.07 |

**Supplementary Figure 44.** GC spectra of stereoiso-enriched (up) and stereoisomeric (down) mixture of **3g**

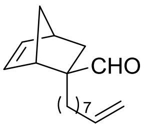


**3h**

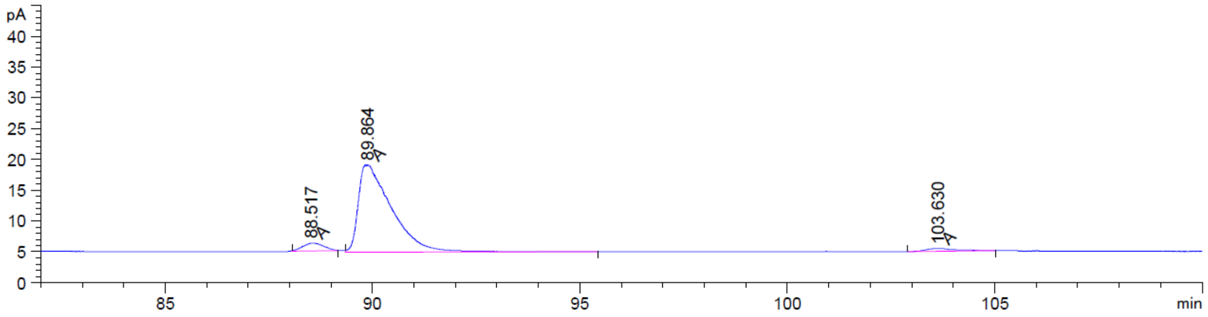


| Peak # | Name | Ret. Time/min | Area/% |
|---|---|---|---|
| 1 | exo 1 | 88.52 | 4.97 |
| 2 | exo 2 | 89.86 | 92.21 |
| 3 | endo 1 & 2 | 103.63 | 2.83 |

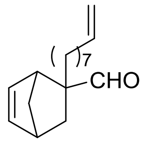


stereoisomeric mixture

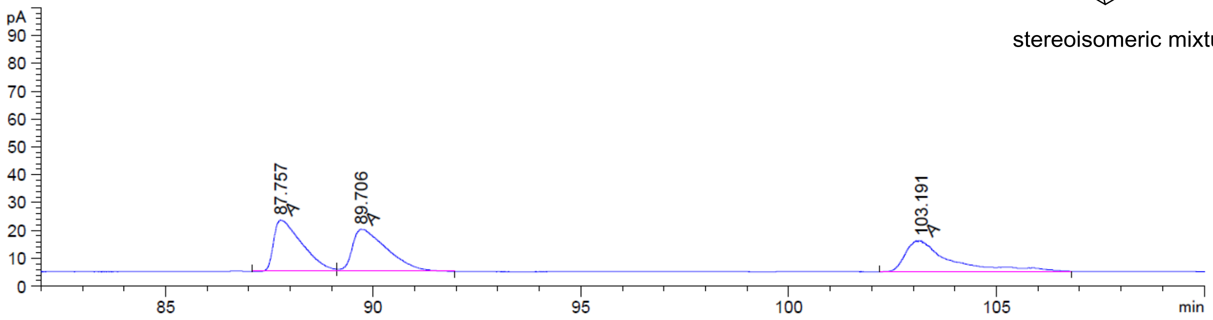


| Peak # | Name | Ret. Time/min | Area/% |
|---|---|---|---|
| 1 | exo 1 | 87.76 | 32.93 |
| 2 | exo 2 | 89.71 | 33.05 |
| 3 | endo 1 & 2 | 103.19 | 34.02 |

**Supplementary Figure 45.** GC spectra of stereoiso-enriched (up) and stereoisomeric (down) mixture of **3h**

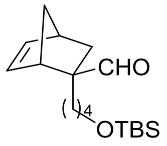


**3i**

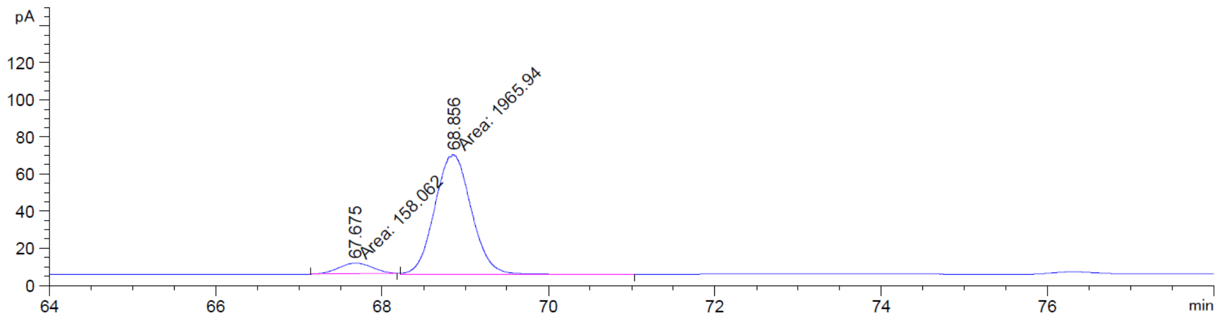


| Peak # | Name | Ret. Time/min | Area/% |
|---|---|---|---|
| 1 | exo 1 | 67.68 | 7.44 |
| 2 | exo 2 | 68.86 | 92.56 |

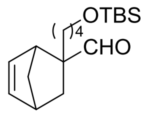


stereoisomeric mixture

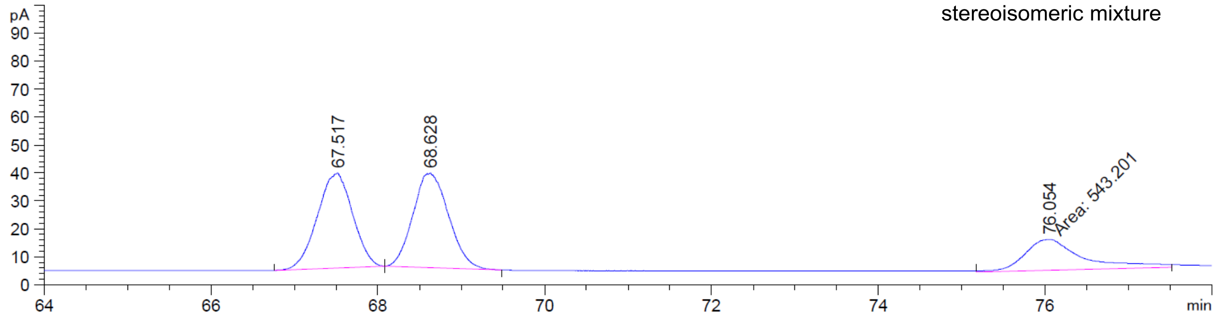


| Peak # | Name | Ret. Time/min | Area/% |
|---|---|---|---|
| 1 | exo 1 | 67.52 | 39.14 |
| 2 | exo 2 | 68.63 | 39.52 |
| 3 | endo 1 & 2 | 76.05 | 21.34 |

**Supplementary Figure 46.** GC spectra of stereoiso-enriched (up) and stereoisomeric (down) mixture of **3i**

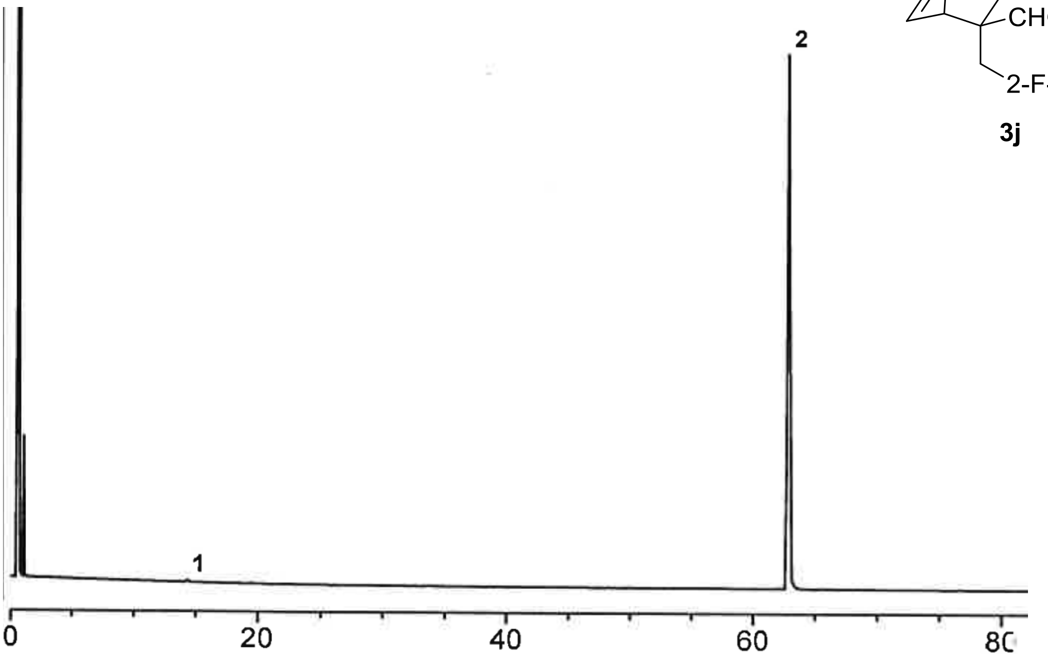


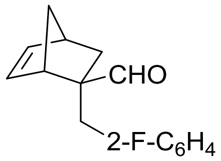


3j

| Peak # | Name | Ret. Time/min | Area/% |
|---|---|---|---|
| 2 | exo | 62.89 | 100.00 |

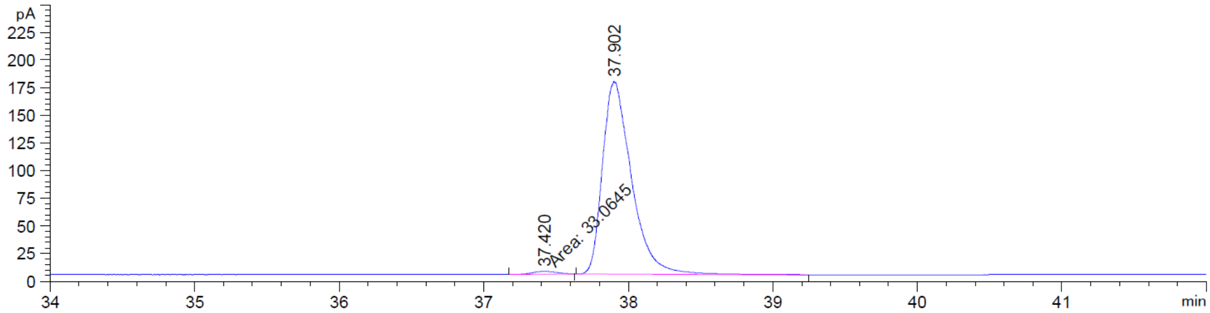


| Peak # | Name | Ret. Time/min | Area/% |
|---|---|---|---|
| 1 | exo 1 | 37.42 | 1.34 |
| 2 | exo 2 | 37.90 | 98.66 |

**Supplementary Figure 47.** GC spectra of stereoiso-enriched **3j** analyzed by the achiral stationary phase (up) and by the chiral stationary phase (down)

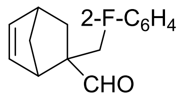


stereoisomeric mixture

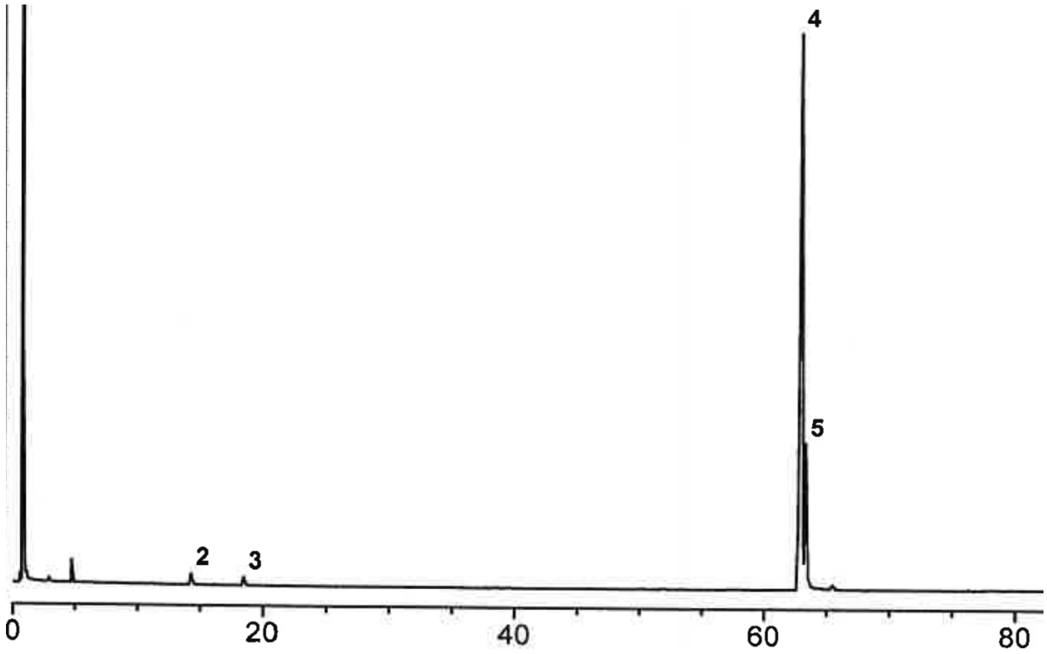


| Peak # | Name | Ret. Time/min | Area/% |
|---|---|---|---|
| 4 | exo | 62.99 | 80.39 |
| 5 | endo | 63.33 | 19.61 |

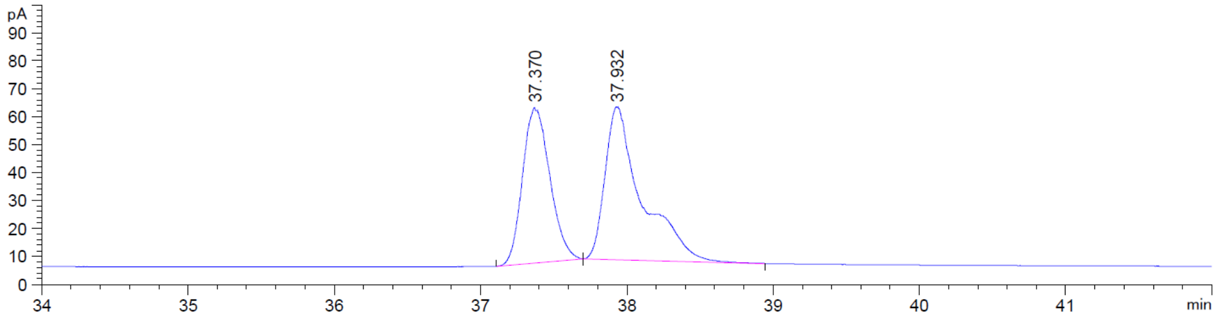


| Peak # | Name | Ret. Time/min | Area/% |
|---|---|---|---|
| 1 | exo 1 | 37.37 | 42.98 |
| 2 | exo 2 | 37.93 | |
| 3 | endo 1 & 2 | | |

**Supplementary Figure 48.** GC spectra of stereoisomeric mixture of **3j** analyzed by the achiral stationary phase (up) and by the chiral stationary phase (down)

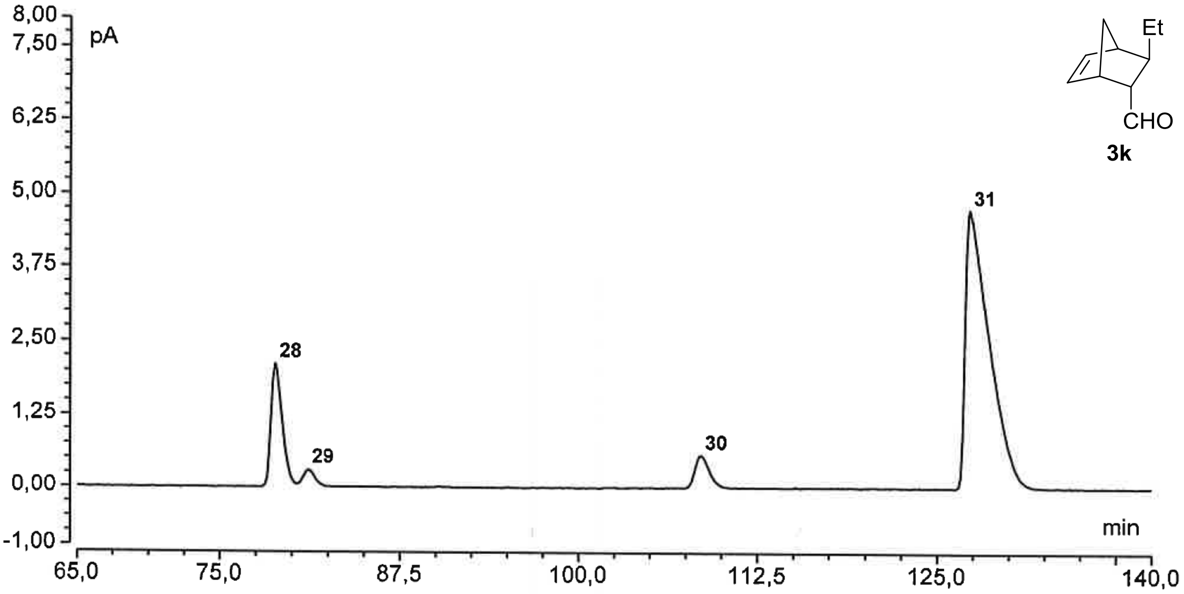


| Peak # | Name | Ret. Time/min | Area/% |
|---|---|---|---|
| 28 | exo 1 | 78.84 | 15.92 |
| 29 | exo 2 | 81.11 | 2.50 |
| 30 | endo 1 | 108.60 | 5.54 |
| 31 | endo 2 | 127.27 | 76.04 |

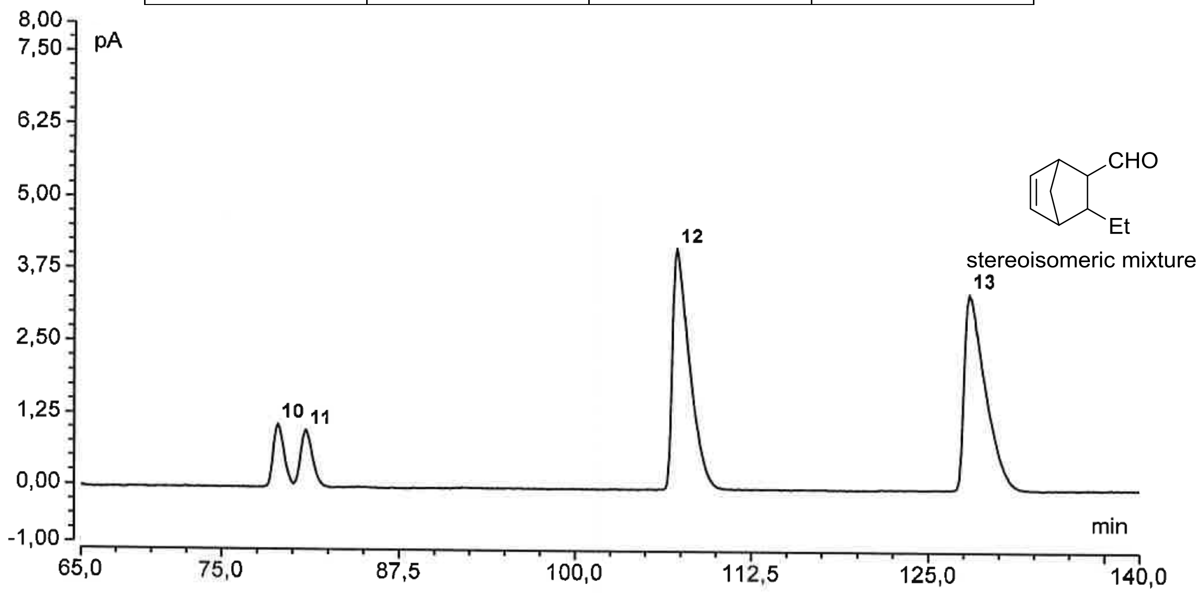


| Peak # | Name | Ret. Time/min | Area/% |
|---|---|---|---|
| 10 | exo 1 | 78.94 | 6.94 |
| 11 | exo 2 | 80.93 | 6.95 |
| 12 | endo 1 | 107.21 | 43.00 |
| 13 | endo 2 | 127.86 | 43.11 |

**Supplementary Figure 49.** GC spectra of stereoiso-enriched (up) and stereoisomeric (down) mixture of **3k**

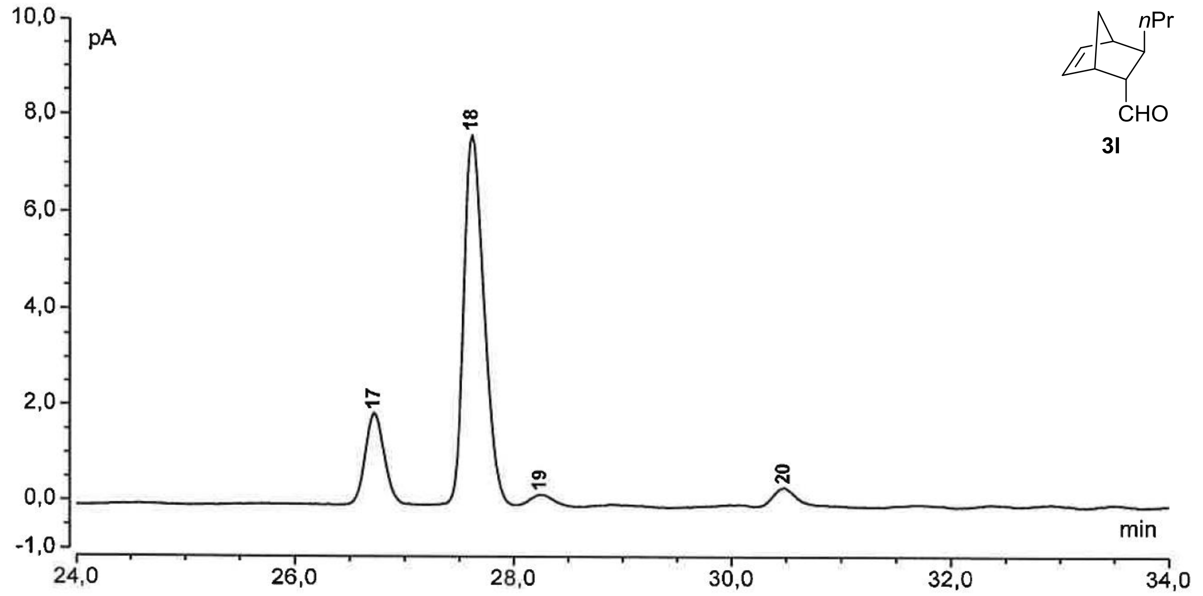


| Peak # | Name | Ret. Time/min | Area/% |
|---|---|---|---|
| 17 | exo 1 | 26.72 | 17.25 |
| 18 | endo 1 | 27.62 | 75.48 |
| 19 | exo 2 | 28.24 | 2.85 |
| 20 | endo 2 | 30.48 | 4.43 |

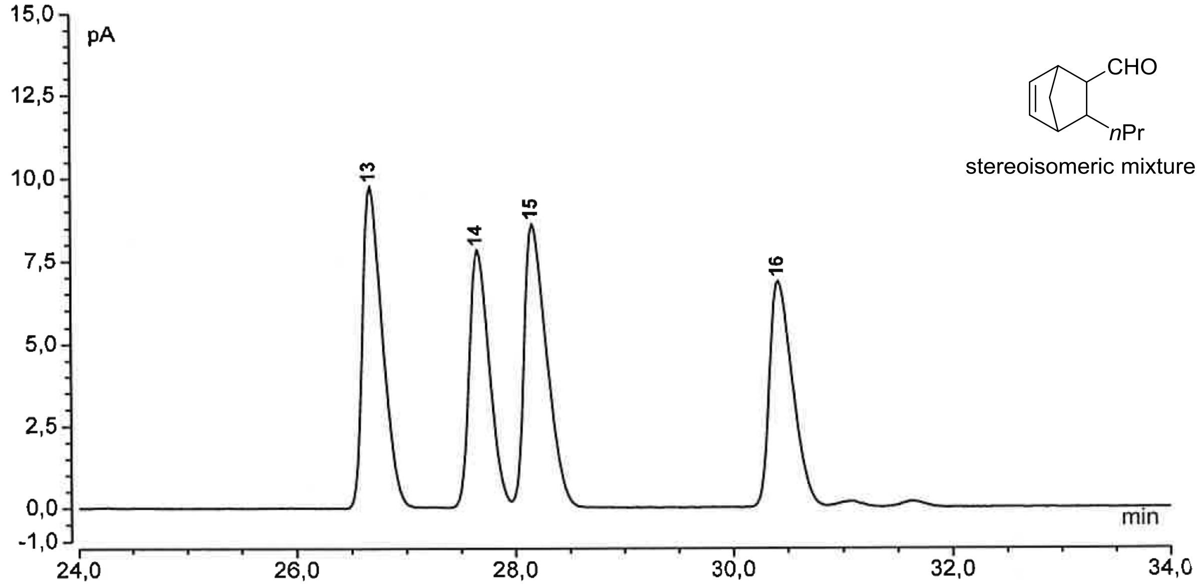


| Peak # | Name | Ret. Time/min | Area/% |
|---|---|---|---|
| 13 | exo 1 | 26.68 | 27.40 |
| 14 | endo 1 | 27.65 | 22.53 |
| 15 | exo 2 | 28.16 | 27.45 |
| 16 | endo 2 | 30.42 | 22.62 |

**Supplementary Figure 50.** GC spectra of stereoiso-enriched (up) and stereoisomeric (down) mixture of **3l**

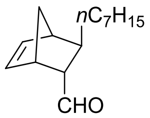


**3m**

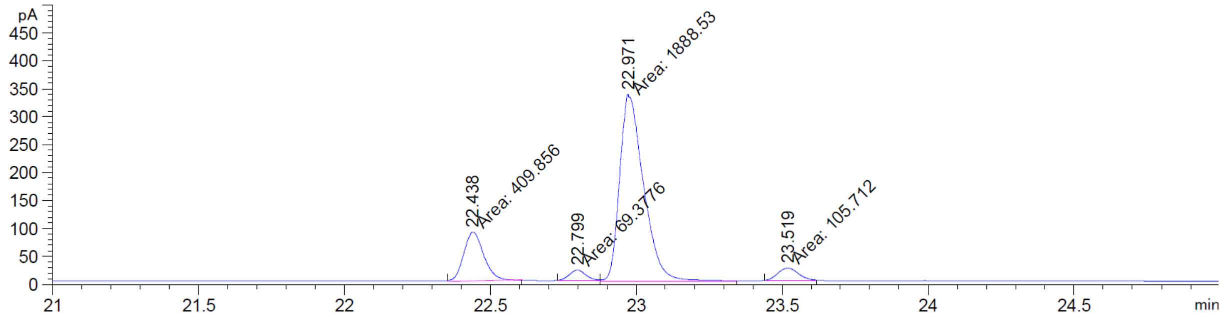


| Peak # | Name | Ret. Time/min | Area/% |
|---|---|---|---|
| 1 | exo 1 | 22.44 | 16.57 |
| 2 | exo 2 | 22.80 | 2.80 |
| 3 | endo 1 | 22.97 | 76.35 |
| 4 | endo 2 | 23.52 | 4.27 |

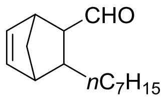


stereoisomeric mixture

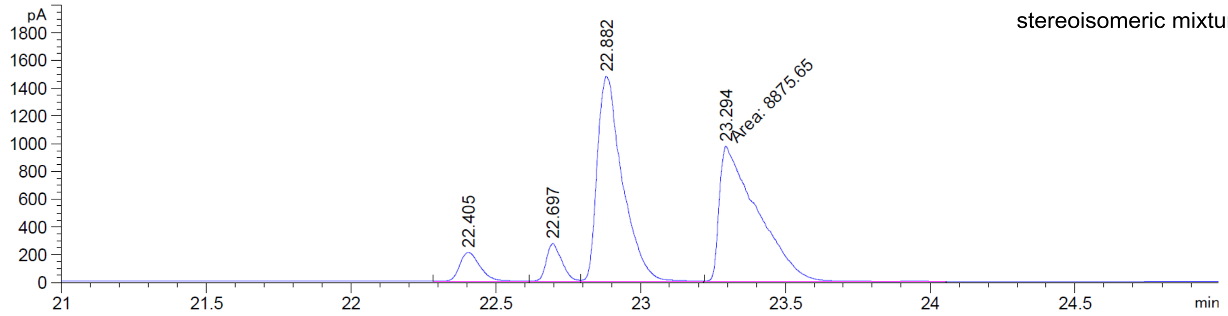


| Peak # | Name | Ret. Time/min | Area/% |
|---|---|---|---|
| 1 | exo 1 | 22.41 | 4.99 |
| 2 | exo 2 | 22.70 | 4.97 |
| 3 | endo 1 | 22.88 | 44.95 |
| 4 | endo 2 | 23.29 | 45.07 |

**Supplementary Figure 51.** GC spectra of stereoiso-enriched (up) and stereoisomeric (down) mixture of **3m**

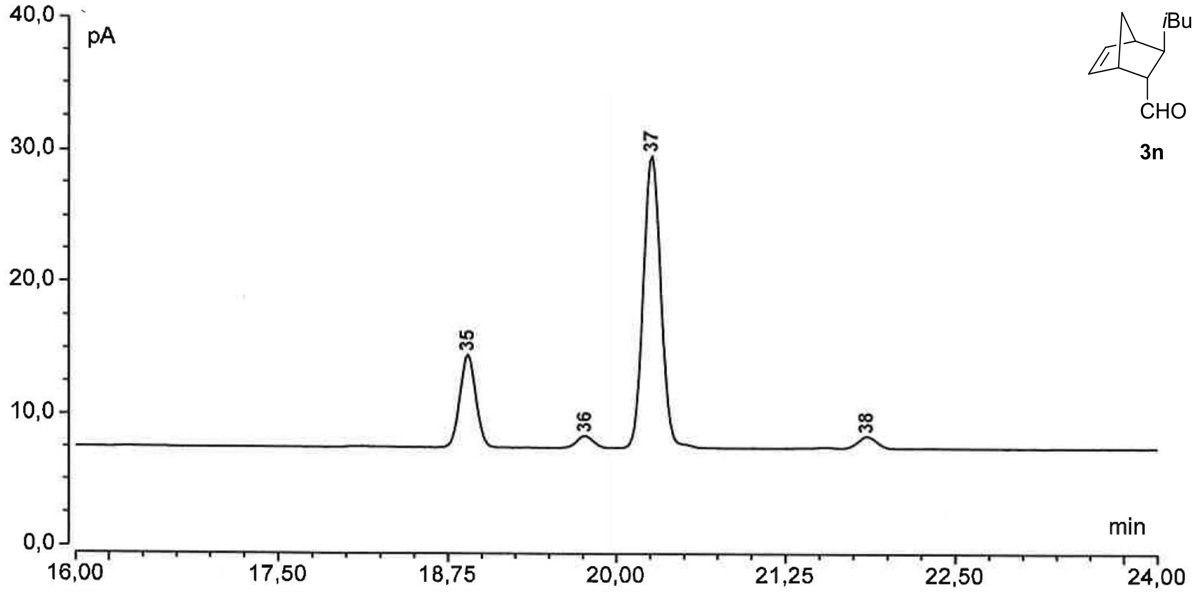


| Peak # | Name | Ret. Time/min | Area/% |
|---|---|---|---|
| 35 | exo 1 | 18.90 | 21.02 |
| 36 | exo 2 | 19.77 | 2.89 |
| 37 | endo 1 | 20.26 | 72.89 |
| 38 | endo 2 | 21.85 | 3.21 |

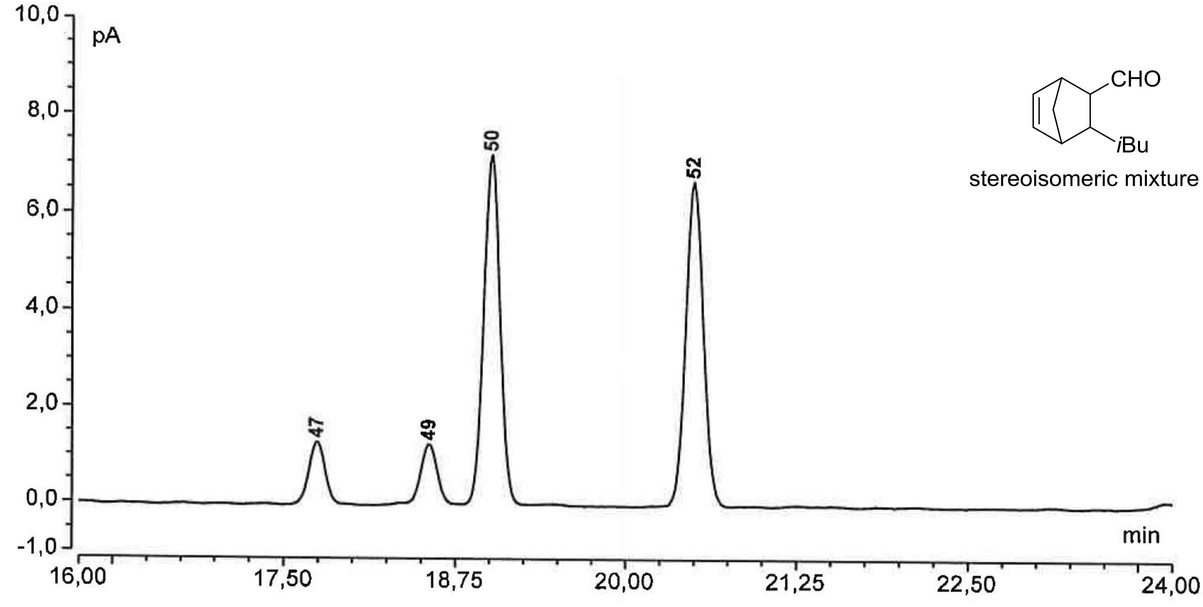


| Peak # | Name | Ret. Time/min | Area/% |
|---|---|---|---|
| 47 | exo 1 | 17.74 | 7.58 |
| 48 | exo 2 | 18.56 | 7.49 |
| 49 | endo 1 | 19.02 | 42.47 |
| 52 | endo 2 | 20.50 | 42.45 |

**Supplementary Figure 52.** GC spectra of stereoiso-enriched (up) and stereoisomeric (down) mixture of **3n**

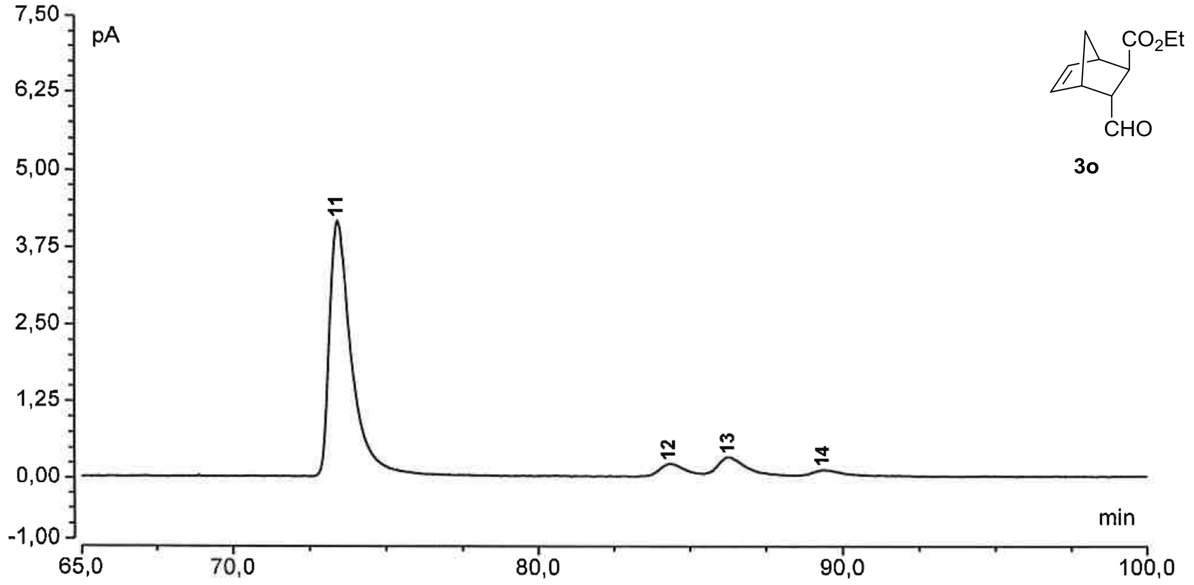


| Peak # | Name | Ret. Time/min | Area/% |
|---|---|---|---|
| 11 | endo 1 | 73.40 | 83.95 |
| 12 | exo 1 | 84.32 | 4.90 |
| 13 | endo 2 | 86.27 | 8.58 |
| 14 | exo 2 | 89.37 | 2.57 |

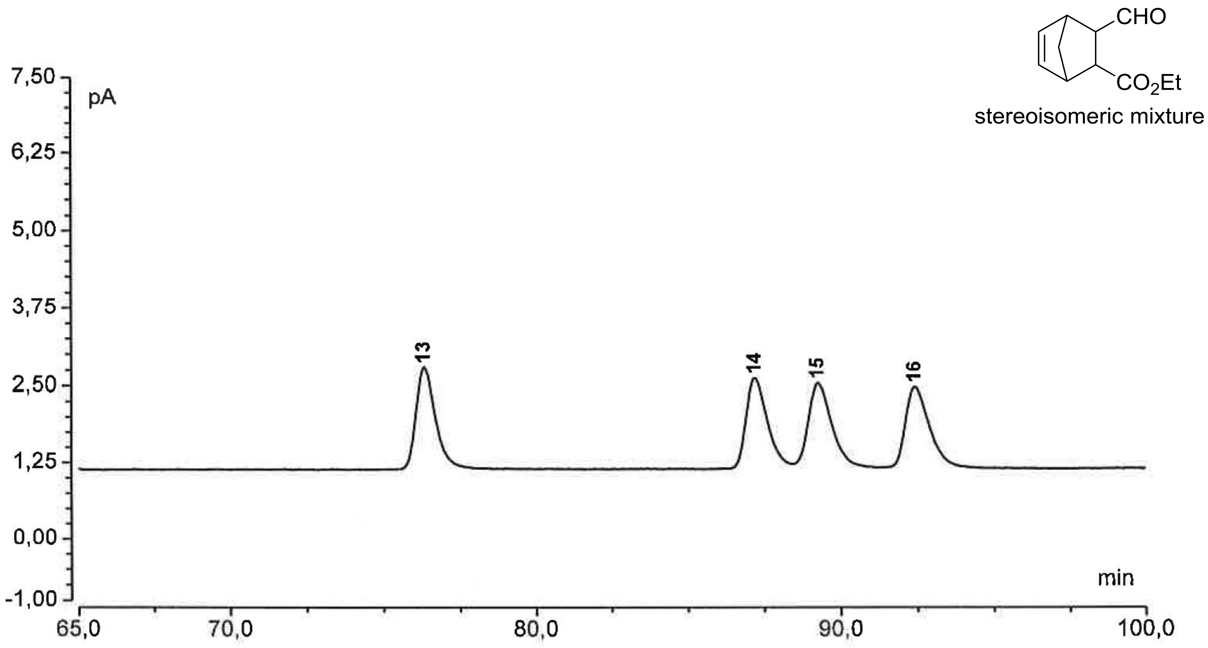


| Peak # | Name | Ret. Time/min | Area/% |
|---|---|---|---|
| 13 | endo 1 | 76.33 | 25.22 |
| 14 | exo 1 | 87.21 | 24.88 |
| 15 | endo 2 | 89.26 | 25.66 |
| 16 | exo 2 | 92.43 | 24.25 |

**Supplementary Figure 53.** GC spectra of stereoiso-enriched (up) and stereoisomeric (down) mixture of **3o**

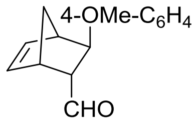


**3p**

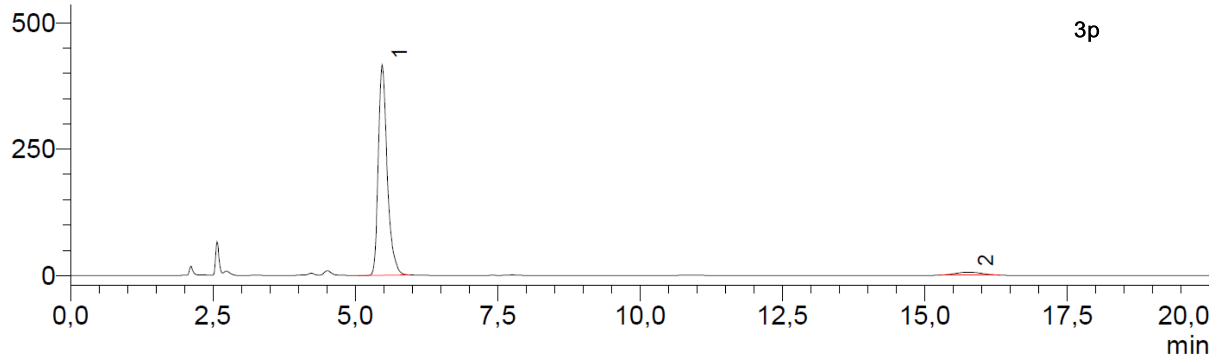


| Peak # | Name | Ret. Time/min | Area/% |
|---|---|---|---|
| 1 | endo 1 | 5.47 | 95.95 |
| 2 | endo 2 | 15.77 | 4.05 |

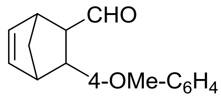


stereoisomeric mixture

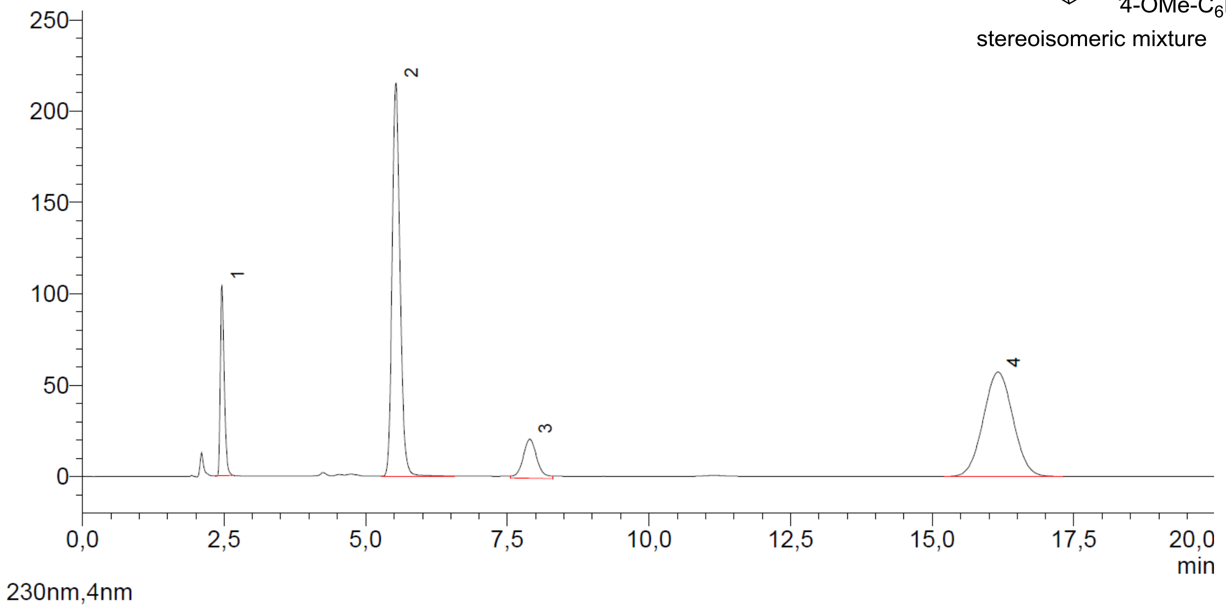


| Peak # | Name | Ret. Time/min | Area/% |
|---|---|---|---|
| 1 | exo 1 | 2.46 | 7.04 |
| 2 | endo 1 | 5.53 | 41.36 |
| 3 | exo 2 | 7.90 | 7.13 |
| 4 | endo 2 | 16.16 | 41.28 |

**Supplementary Figure 54.** HPLC spectra of stereoiso-enriched (up) and stereoisomeric (down) mixture of **3p**

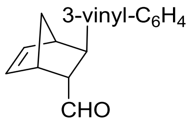


**3q**

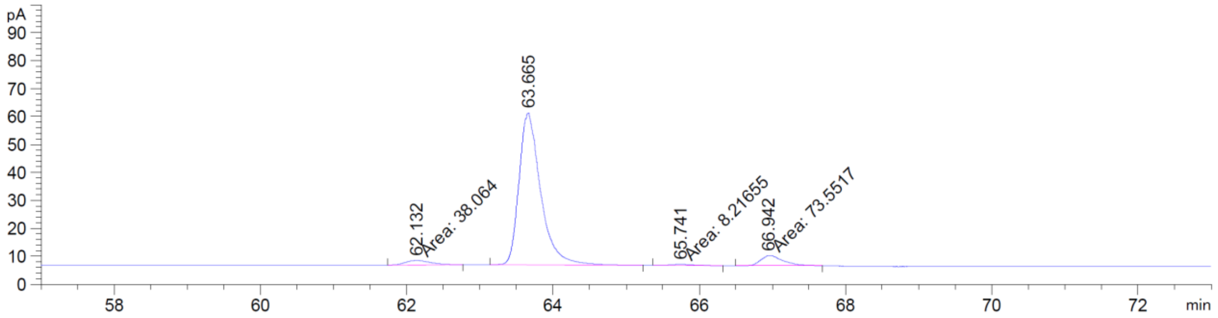


| Peak # | Name | Ret. Time/min | Area/% |
|---|---|---|---|
| 1 | exo 1 | 62.13 | 3.07 |
| 2 | endo 1 | 63.67 | 90.33 |
| 3 | exo 2 | 65.74 | 0.66 |
| 4 | endo 2 | 66.94 | 5.94 |

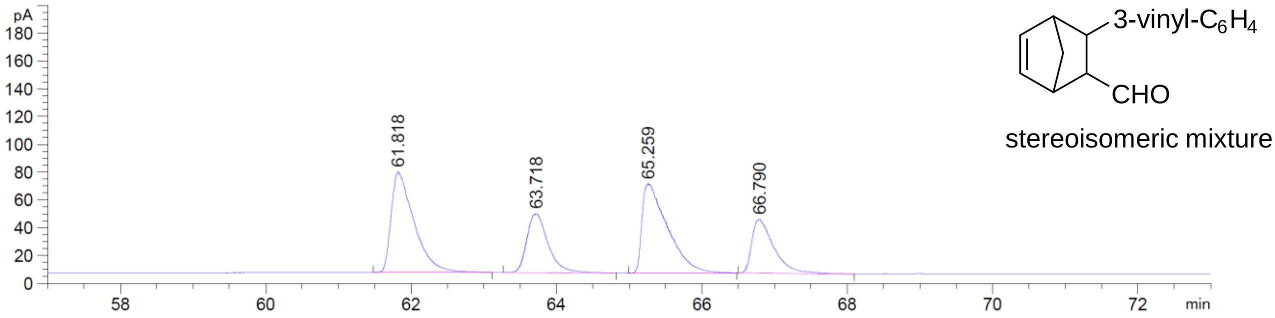


stereoisomeric mixture

| Peak # | Name | Ret. Time/min | Area/% |
|---|---|---|---|
| 1 | exo 1 | 61.82 | 32.96 |
| 2 | endo 1 | 63.72 | 17.82 |
| 3 | exo 2 | 65.26 | 32.44 |
| 4 | endo 2 | 66.79 | 16.78 |

**Supplementary Figure 55.** GC spectra of stereoiso-enriched (up) and stereoisomeric (down) mixture of **3q**

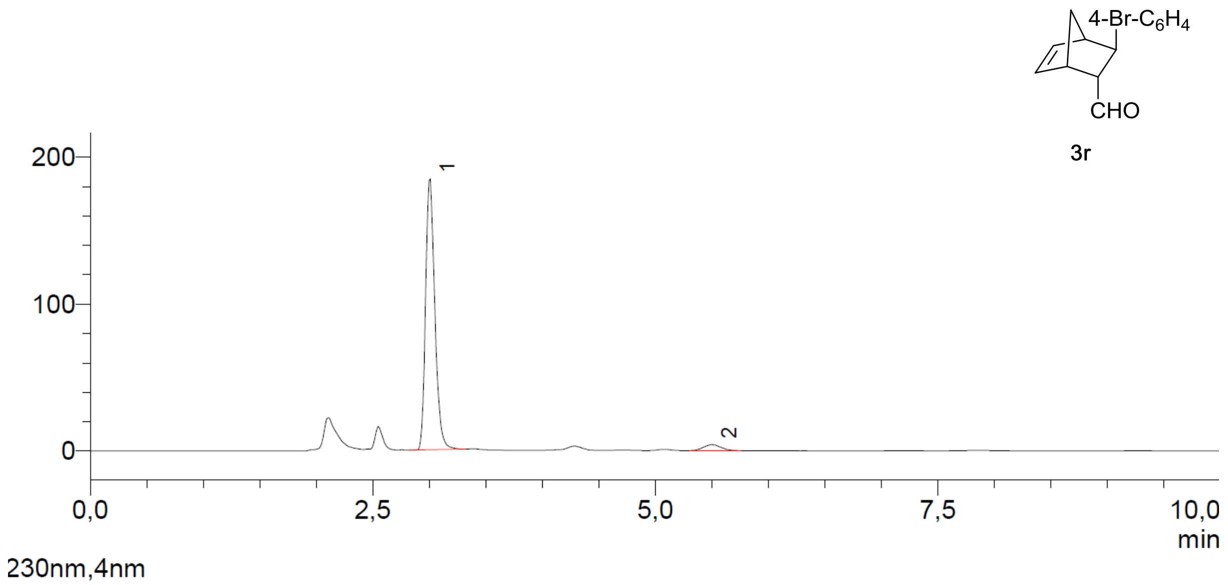


| Peak # | Name | Ret. Time/min | Area/% |
|---|---|---|---|
| 1 | endo 1 | 3.03 | 96.01 |
| 2 | endo 2 | 5.50 | 3.99 |

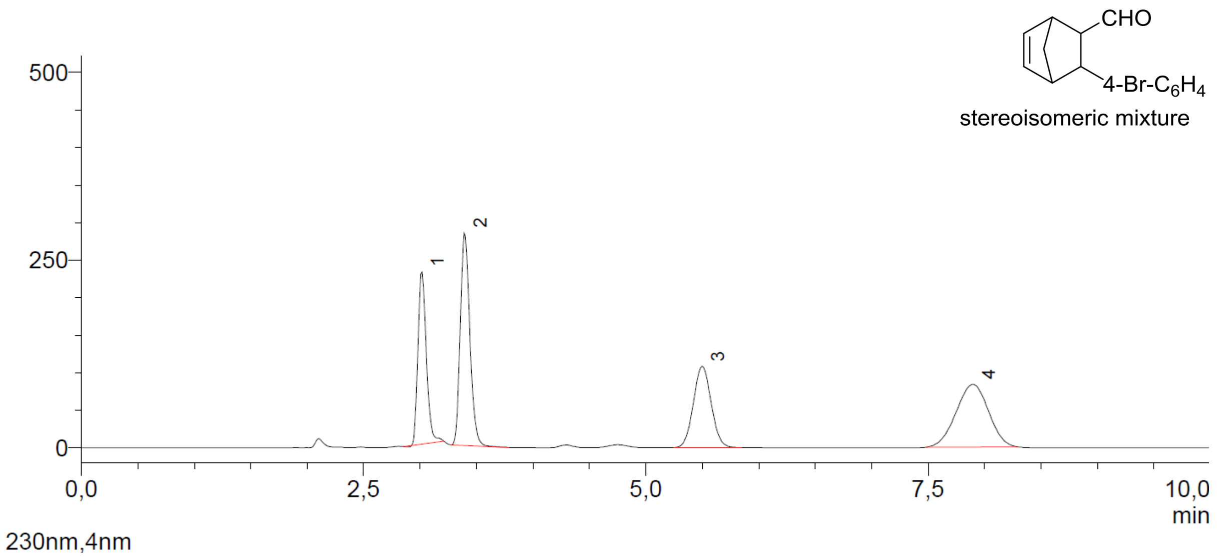


| Peak # | Name | Ret. Time/min | Area/% |
|---|---|---|---|
| 1 | endo 1 | 3.02 | 20.87 |
| 2 | exo 1 | 3.40 | 29.31 |
| 3 | endo 2 | 5.50 | 20.76 |
| 4 | exo 2 | 7.90 | 29.07 |

**Supplementary Figure 56.** HPLC spectra of stereoiso-enriched (up) and stereoisomeric (down) mixture of **3r**

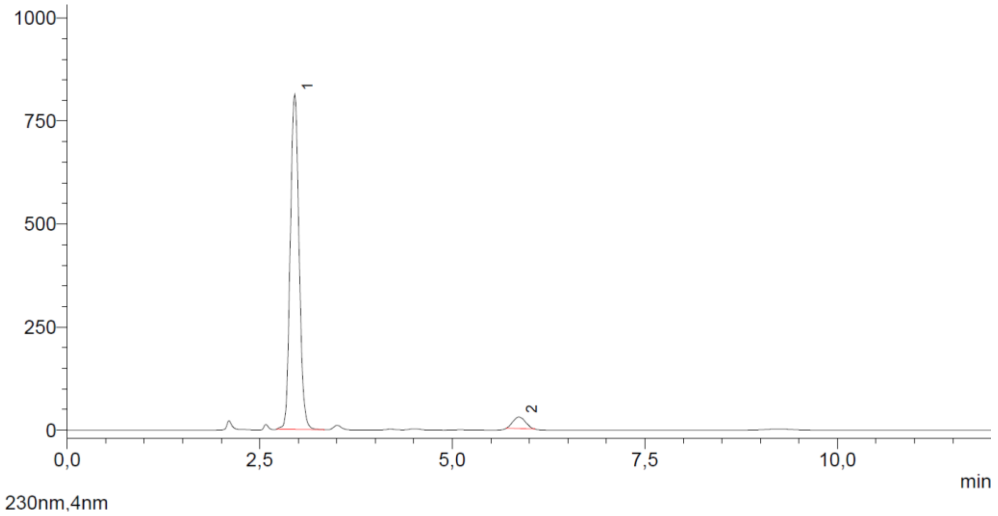


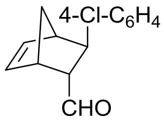


3s

| Peak # | Name | Ret. Time/min | Area/% |
|---|---|---|---|
| 1 | endo 1 | 2.95 | 95.42 |
| 2 | endo 2 | 5.89 | 4.58 |

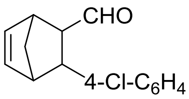


stereoisomeric mixture

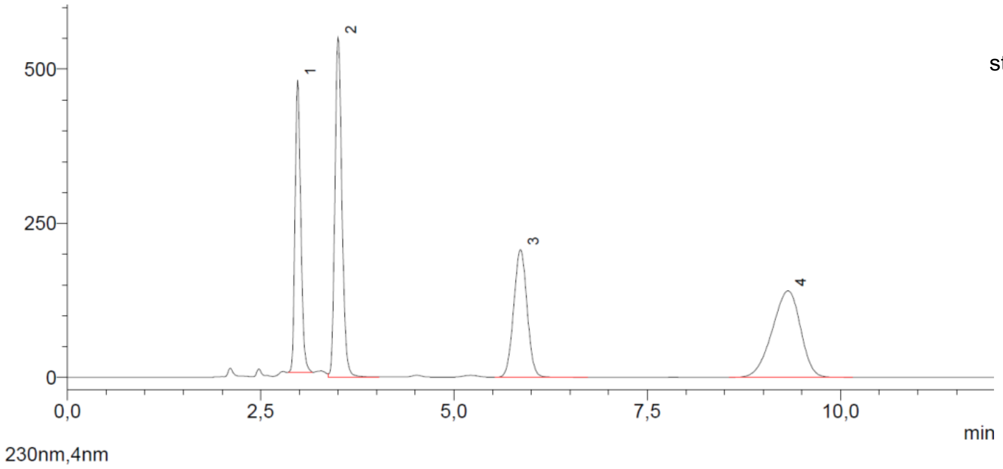


| Peak # | Name | Ret. Time/min | Area/% |
|---|---|---|---|
| 1 | endo 1 | 2.98 | 20.34 |
| 2 | exo 1 | 3.05 | 29.16 |
| 3 | endo 2 | 5.86 | 20.79 |
| 4 | exo 2 | 9.32 | 29.74 |

**Supplementary Figure 57.** HPLC spectra of stereoiso-enriched (up) and stereoisomeric (down) mixture of **3s**

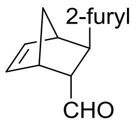


**3t**

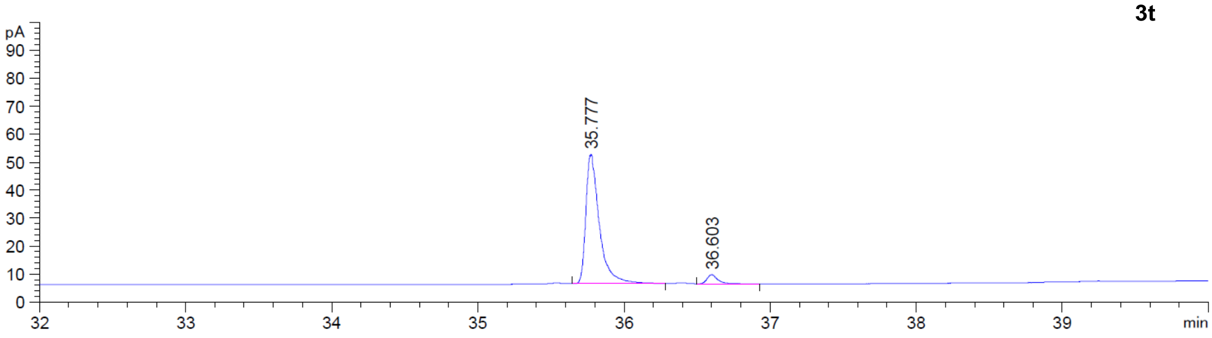


| Peak # | Name | Ret. Time/min | Area/% |
|---|---|---|---|
| 1 | endo 1 | 35.78 | 94.54 |
| 2 | endo 2 | 36.60 | 5.46 |

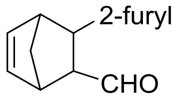


stereoisomeric mixture

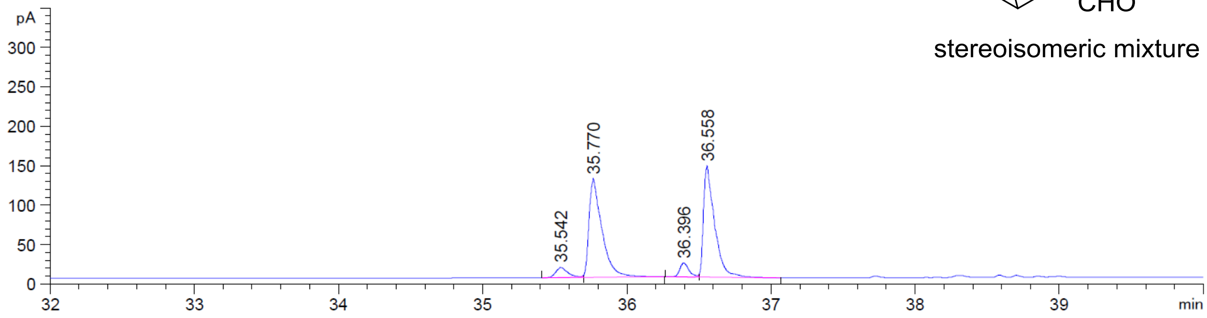


| Peak # | Name | Ret. Time/min | Area/% |
|---|---|---|---|
| 1 | exo 1 | 35.54 | 4.35 |
| 2 | endo 1 | 35.78 | 46.24 |
| 3 | exo 2 | 36.40 | 4.62 |
| 4 | endo 2 | 36.56 | 44.79 |

**Supplementary Figure 58.** GC spectra of stereoiso-enriched (up) and stereoisomeric (down) mixture of **3t**

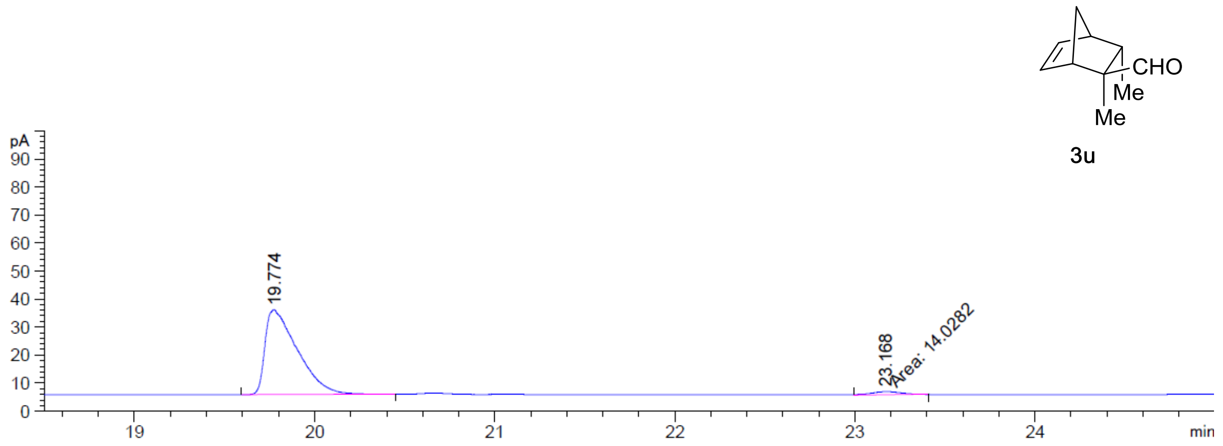


| Peak # | Name | Ret. Time/min | Area/% |
|---|---|---|---|
| 1 | exo 1 | 19.77 | 96.41 |
| 2 | exo 2 | 23.17 | 3.59 |

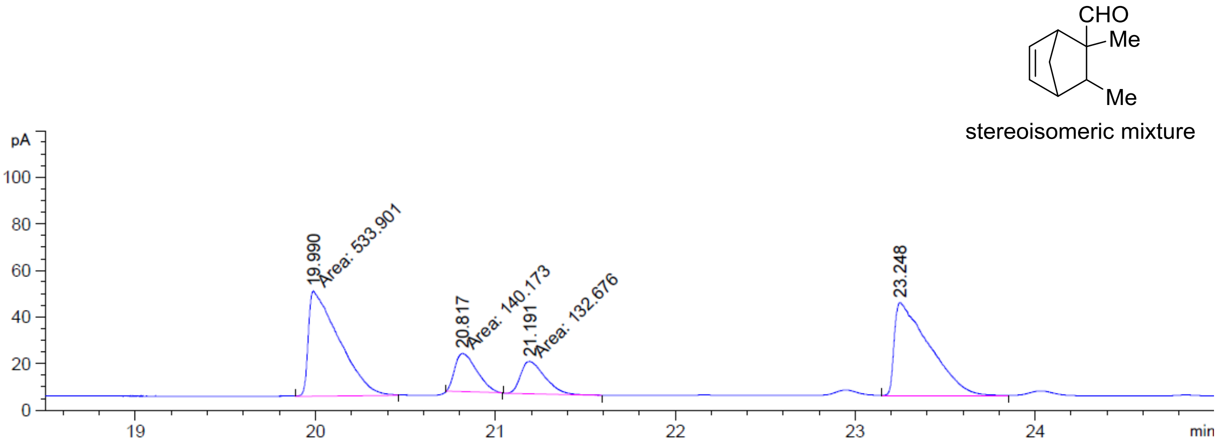


| Peak # | Name | Ret. Time/min | Area/% |
|---|---|---|---|
| 1 | exo 1 | 19.99 | 39.84 |
| 2 | endo 1 | 20.82 | 10.46 |
| 3 | endo 2 | 21.19 | 9.91 |
| 4 | exo 2 | 23.25 | 39.79 |

**Supplementary Figure 59.** GC spectra of stereoiso-enriched (up) and stereoisomeric (down) mixture of **3u**

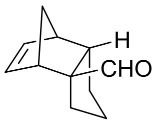


**3v**

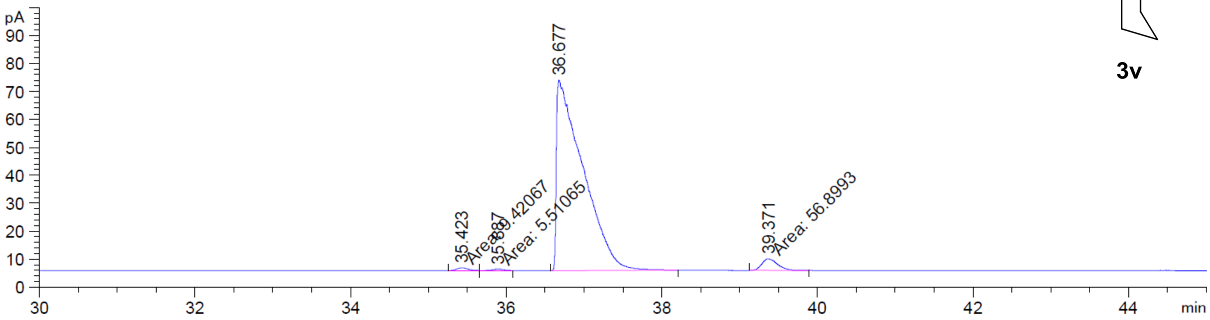


| Peak # | Name | Ret. Time/min | Area/% |
|---|---|---|---|
| 1 | endo 1 | 35.423 | 0.56 |
| 2 | endo 2 | 35.887 | 0.33 |
| 3 | exo 1 | 36.677 | 95.74 |
| 4 | exo 2 | 39.371 | 3.37 |

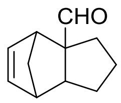


stereoisomeric mixture

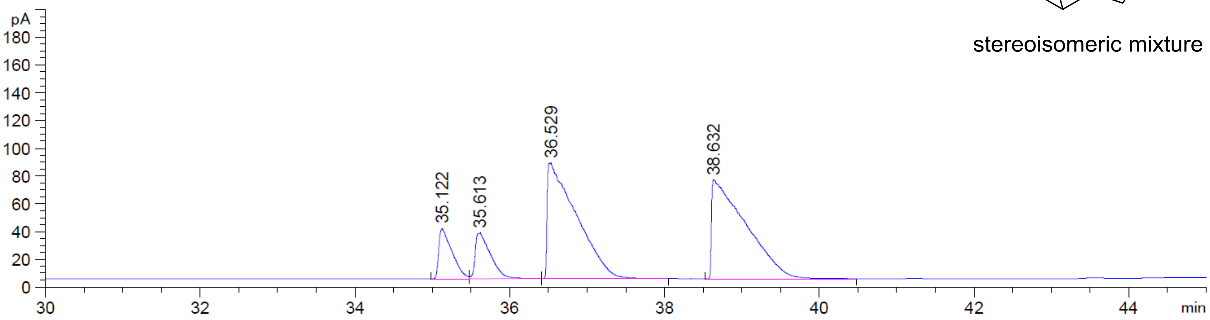


| Peak # | Name | Ret. Time/min | Area/% |
|---|---|---|---|
| 1 | endo 1 | 35.122 | 8.23 |
| 2 | endo 2 | 35.613 | 8.60 |
| 3 | exo 1 | 36.529 | 41.43 |
| 4 | exo 2 | 38.632 | 41.74 |

**Supplementary Figure 60.** GC spectra of stereoiso-enriched (up) and stereoisomeric (down) mixture of **3v**

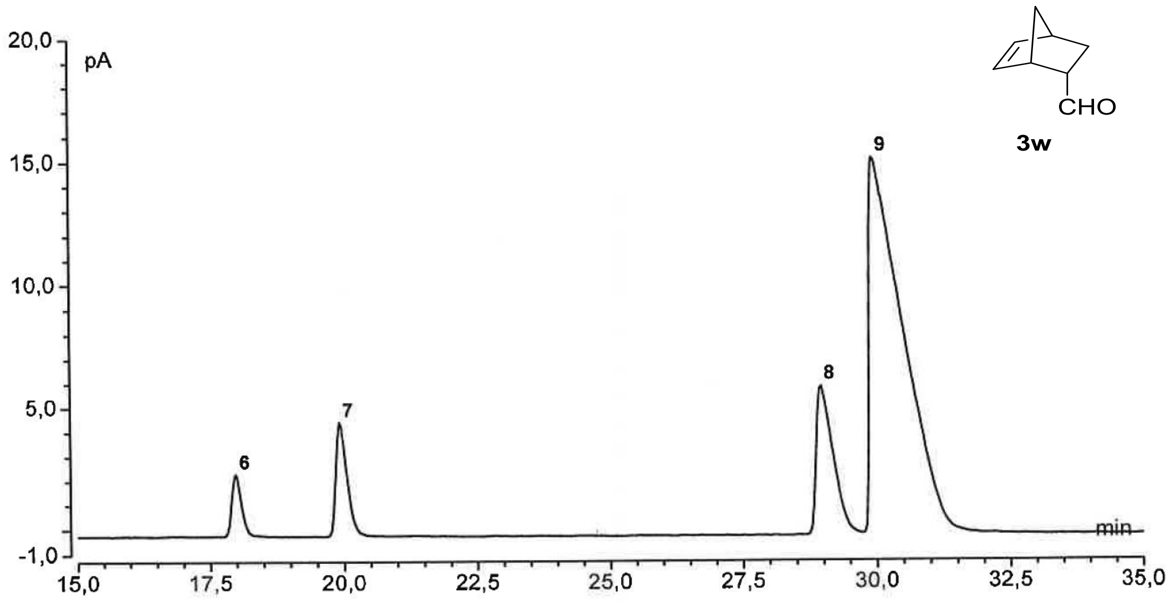


| Peak # | Name | Ret. Time/min | Area/% |
|---|---|---|---|
| 6 | exo 1 | 17.98 | 3.51 |
| 7 | exo 2 | 19.92 | 7.51 |
| 8 | endo 1 | 28.96 | 14.62 |
| 9 | endo 2 | 29.94 | 74.36 |

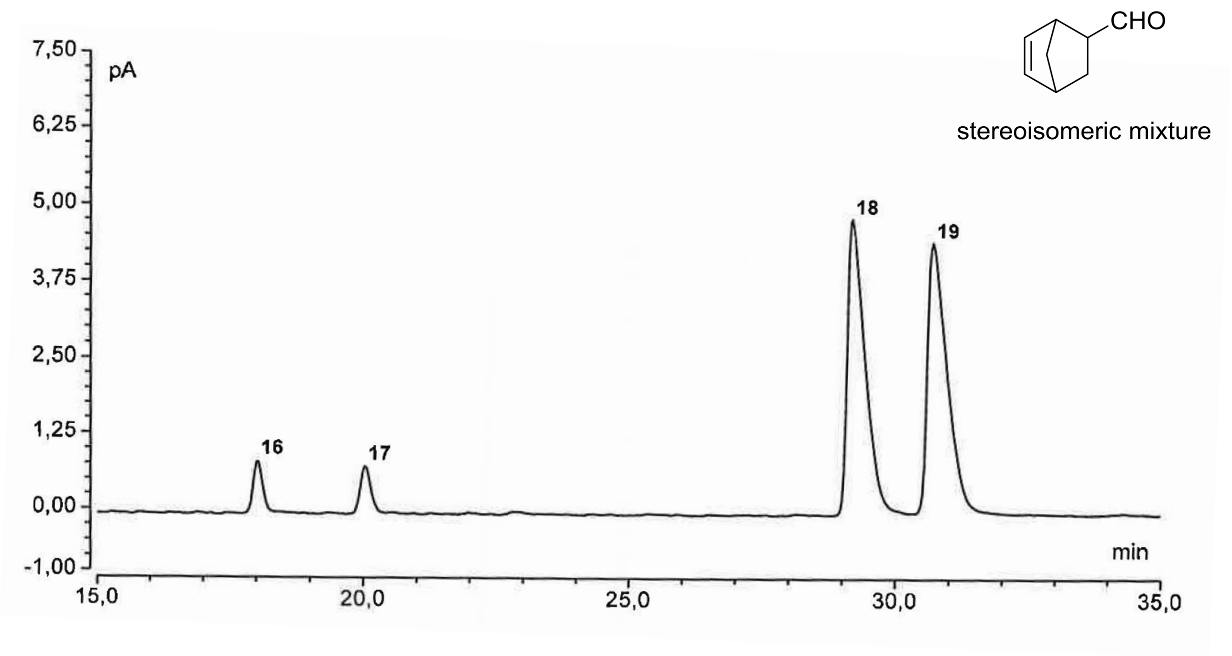


| Peak # | Name | Ret. Time/min | Area/% |
|---|---|---|---|
| 16 | exo 1 | 18.02 | 4.17 |
| 17 | exo 2 | 20.03 | 4.27 |
| 18 | endo 1 | 29.21 | 45.68 |
| 19 | endo 2 | 30.72 | 45.88 |

**Supplementary Figure 61.** GC spectra of stereoiso-enriched (up) and stereoisomeric (down) mixture of **3w**

**Supplementary Note 5.** NMR-Based Structural investigations of the catalysts (**4g**–**4i**)

All NMR spectra for structural investigations of catalysts **4g**–**4i** were recorded on an Avance III 500 spectrometer (499.87 MHz) equipped with a BBFO $^{1}H$/BB(incl. $^{19}F$) probehead from Bruker Biospin GmbH and for $^{19}F$,$^{13}C$-correlation and decoupling experiments a TBO $^{1}H/^{19}F$/BB probehead from Bruker Biospin GmbH. All NMR data were processed and analysed with MestReNova 12.0.2. Samples were prepared by dissolving **4g**–**4i** in $CDCl_3$, to a concentration of ca.  8 mM. All samples were introduced into 5mm tubes with caps. Full $^{1}H$, $^{13}C$, $^{31}P$ and $^{19}F$ assignment, including the two magnetically distinct halves of the chiral BINOL backbone with phenanthrene substituents, were obtained at 25 °C from standard 1D experiments, including $^{13}C\{^{1}H,^{19}F\}$, as well as 2D correlation experiments. The correlation experiments, measured with high digital resolution in both dimensions (typically 4k x 2k or 2k x 1k), included $^{1}H,^{1}H$ DQF-COSY, $^{1}H,^{1}H$-ROESY, $^{1}H,^{13}C$-HSQC, $^{1}H,^{13}C$-$^{n}J$-HMQC, $^{1}H,^{19}F$-HOESY, $^{19}F,^{19}F$-COSY, $^{19}F,^{19}F$-ROESY, $^{19}F,^{13}C$-HSQC, $^{19}F,^{13}C$-$^{n}J$-HSQC, $^{1}H,^{31}P$-$^{n}J$-HMQC. The $^{1}H$ chemical shift scale was referenced according to residual 1H solvent signal relative to TMS whereas the $\Xi$ values corresponding to TMS were applied for $^{13}C$ (25.145020%), $^{31}P$ (40.480742%) and $^{19}F$ (94.094011%).

The most important ROESY and HOESY correlations are depicted as blue arrows. Structurally it is most likely, that the HOEs occur between $^{19}F$ and the lower wing of the same half of the catalyst, and between $^{19}F$ and the upper wing of the opposite half of the catalyst. In the NMR time scale the structure presents itself as $C_2$-symmetrical, and the two halves of the molecule are isochronous. The higher order spin systems of C2, C1 and C3, which arise from J-couplings with P1 and P1’, prove the dimeric structure.

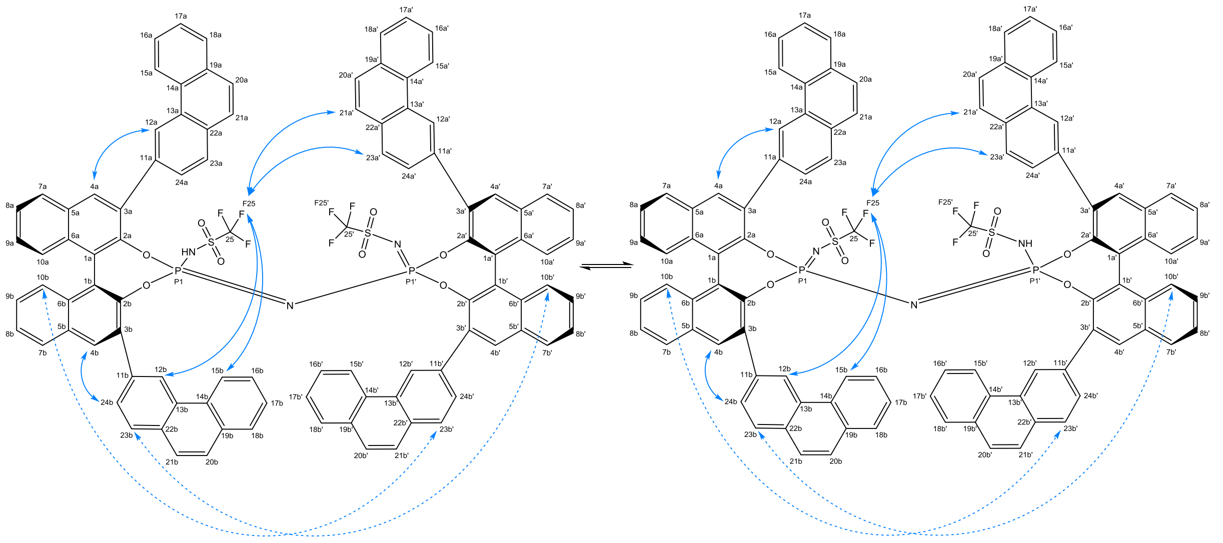

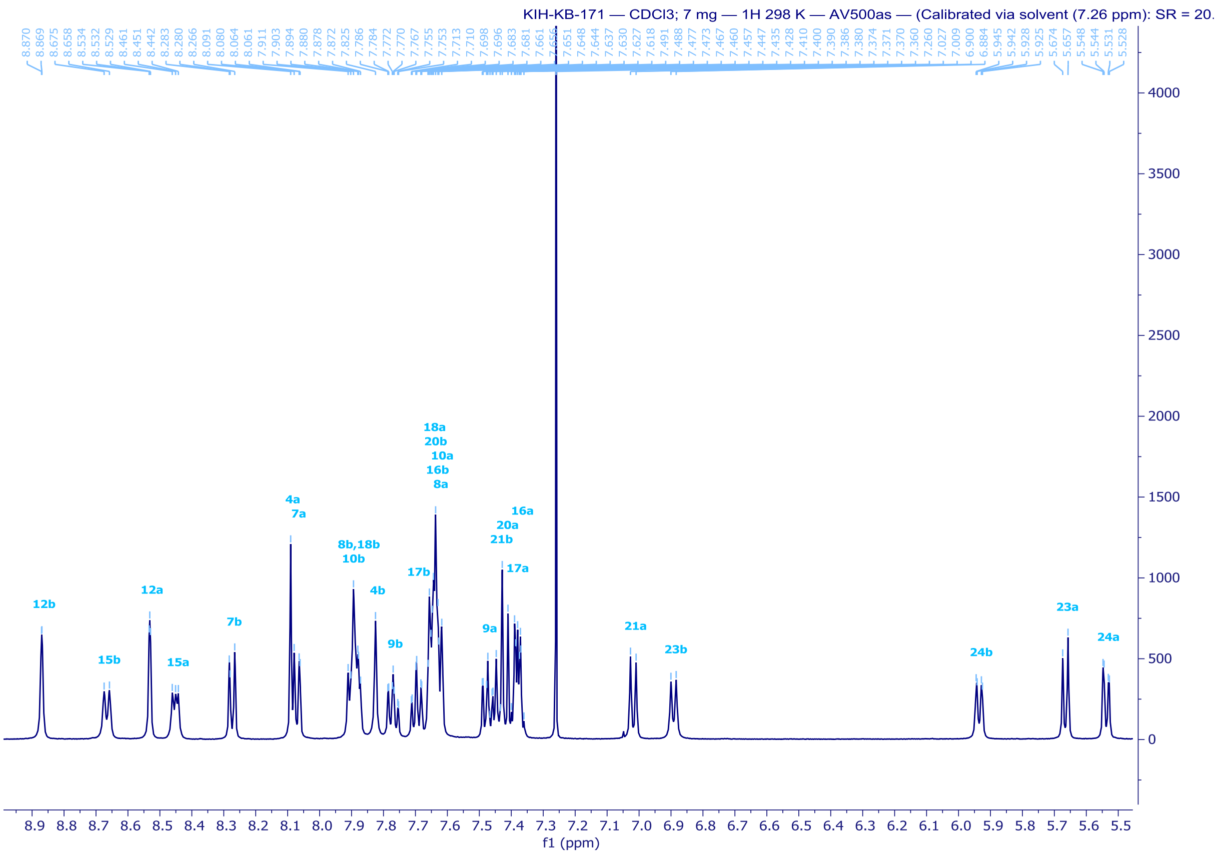


**Supplementary Figure 62.** Structure (up) and $^{1}$H NMR spectrum (down) of **4g**

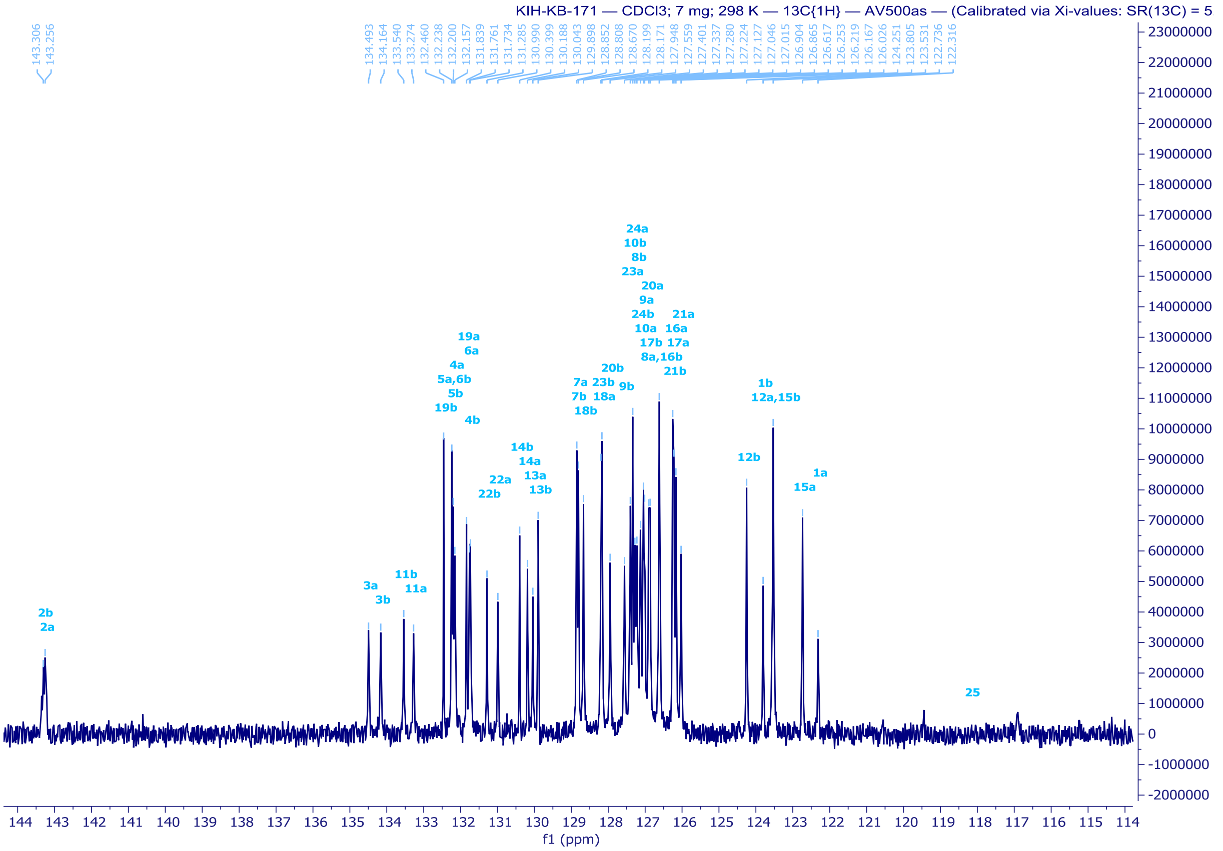


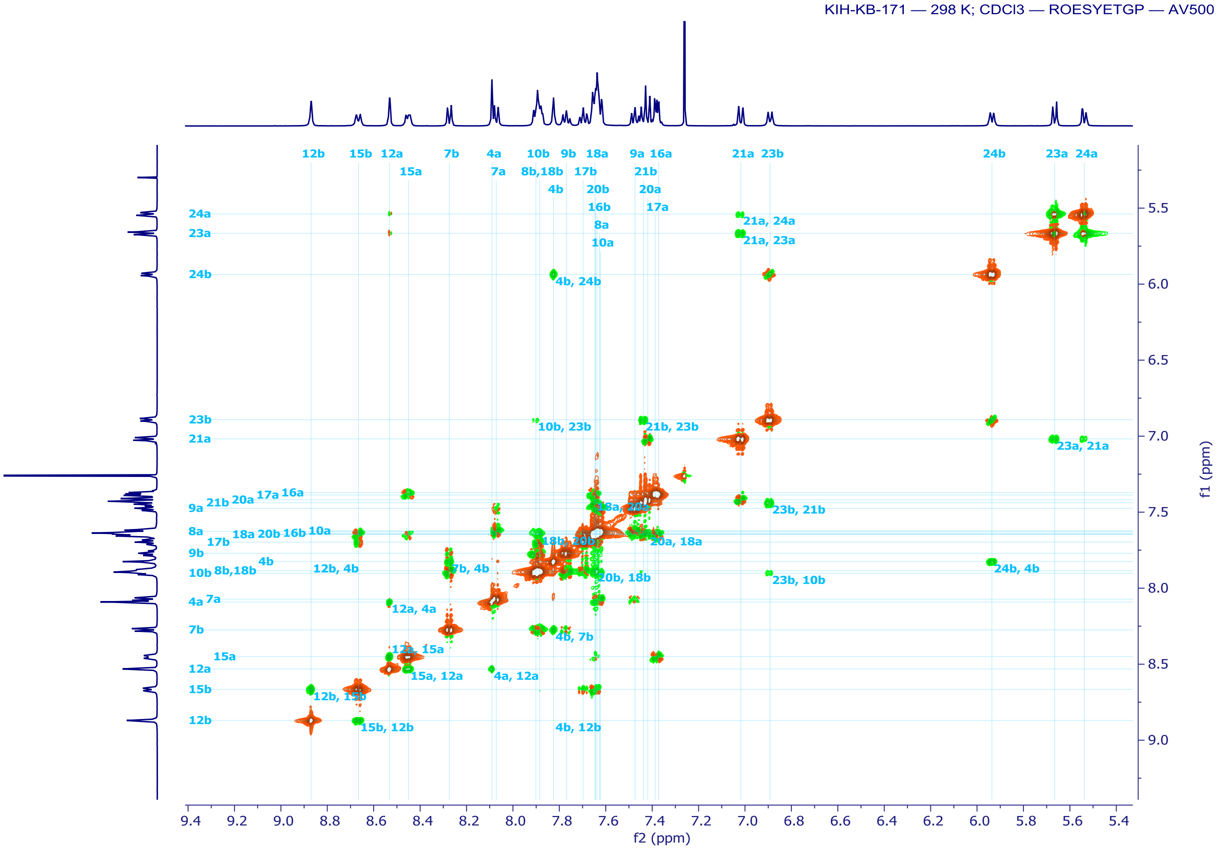


**Supplementary Figure 63.** $^{13}$C NMR (up) and ROESY (down) spectra of **4g**

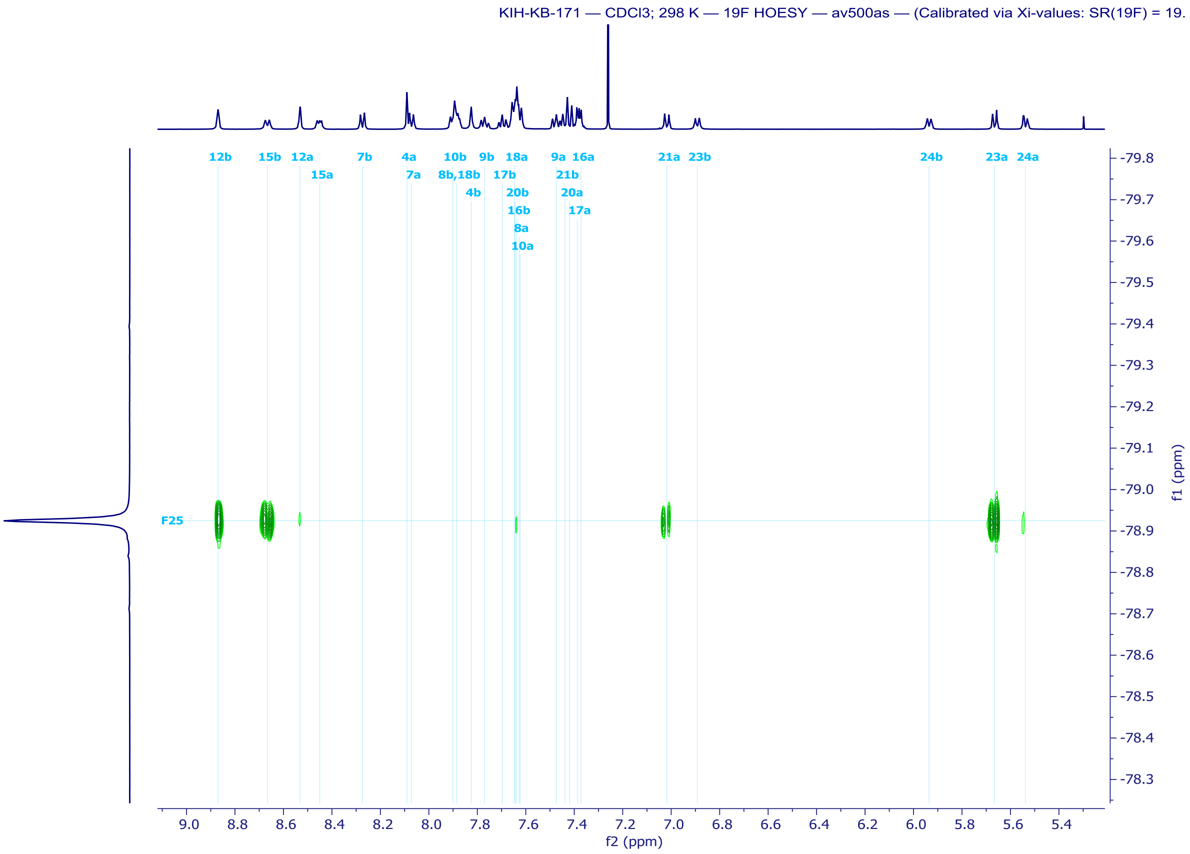


**Supplementary Figure 64.** $^{1}$H,$^{19}$F-HOESY spectrum of **4g**

**Supplementary Table 7.** Upper wings and midsection of the catalyst **4g**

| | δ | J | COSY | HSQC | $^{n}$J-HMQC | ROESY | $^{1}$H,$^{19}$F-HOESY |
|---|---|---|---|---|---|---|---|
| **C1a** | 122.32 | m (AA'X spin system; $^{3}J_{CP}$, $^{5}J_{CP}$) | | | 4a, 10a | | |
| **C2a** | 143.26 | m (AA'X spin system; $^{2}J_{CP}$, $^{4}J_{CP}$) | | | 4a | | |
| **C3a** | 134.49 | m (AA'X spin system; $^{3}J_{CP}$, $^{5}J_{CP}$) | | | 12a, 24a | | |
| **C4a** | 132.16 | | | 4a | 7a | | |
| **H4a** | 8.091 | s | 10a | 4a | 1a, 2a, 7a, 11a | 12a | |
| **C5a** | 132.24 | | | | 8a, 10a | | |
| **C6a** | 131.76 | | | | 7a, 9a | | |
| **C7a** | 128.81 | | | 7a | 4a, 9a | | |
| **H7a** | 8.072 | d 8.5(8a) | 8a, 9a | 7a | 4a, 6a, 9a | | |
| **C8a** | 126.62 | | | 8a | 9a, 10a | | |
| **H8a** | 7.627 | m | 7a, 9a | 8a | 5a, 10a | | |
| **C9a** | 127.02 | | | 9a | 7a, 9a | | |
| **H9a** | 7.474 | m | 7a, 8a, 10a | 9a | 6a, 7a, 8a, 9a, 10a | | |
| **C10a** | 127.05 | | | 10a | 8a, 9a | | |
| **H10a** | 7.623 | m | 4a, 9a | 10a | 1a, 5a, 8a | | |
| **C11a** | 133.27 | | | | 4a, 23a | | |
| **C12a** | 123.53 | | | 12a | 24a | | |
| **H12a** | 8.532 | s | 15a, 21a, 23a, 24a | 12a | 3a, 14a, 22a, 24a | 4a, 15a | (F25) |
| **C13a** | 130.04 | | | | 15a, 21a, 23a | | |
| **C14a** | 130.19 | | | | 12a, 16a, 18a, 20a | | |
| **C15a** | 122.74 | | | 15a | 17a | | |
| **H15a** | 8.450 | m | 12a, 16a, 18a | 15a | 13a, 17a, 19a | 12a | |
| **C16a** | 126.22 | | | 16a | 18a | | |
| **H16a** | 7.372 | m | 15a, 18a | 16a | 14a, 18a | | |
| **C17a** | 126.17 | | | 17a | 15a | | |
| **H17a** | 7.387 | m | 18a | 17a | 15a, 19a | | |
| **C18a** | 128.17 | | | 18a | 16a, 20a | | |
| **H18a** | 7.647 | m | 15a, 16a, 17a | 18a | 14a, 16a, 20a | 20a | |
| **C19a** | 131.84 | | | | 15a, 17a, 21a | | |
| **C20a** | 126.87 | | | 20a | 18a | | |
| **H20a** | 7.419 | d 8.8(21a) | 21a | 20a | 14a, 18a, 22a | 18a | |
| **C21a** | 126.03 | | | 21a | 23a | | |
| **H21a** | 7.018 | d 8.8(20a) | 12a, 20a, 23a | 21a | 13a, 19a, 22a, 23a | 23a, 24a | F25 |
| **C22a** | 130.99 | | | | 12a, 20a, 21a, 23a, 24a | | |
| **C23a** | 127.40 | | | 23a | 21a | | |
| **H23a** | 5.666 | d 8.3(24a) | 12a, 21a, 24a | 23a | 11a, 13a, 21a, 22a | 21a | F25 |
| **C24a** | 127.28 | | | 24a | 12a | | |
| **H24a** | 5.538 | d 8.3(23a), d 1.7(12a) | 12a, 23a | 24a | 3a, 12a, 22a | 21a | (F25) |
| **C25** | 118.19 | q 321.0(F25) | | | | | |
| **F25** | -78.93 | s (F25$_{13C\text{-bound}}$: -79.05 ppm; d 321(C25)) | | | | | 12b, 15b, (12a), (16b), 21a, 23a, (24a) |
| **P1** | -15.33 | | | | | | |

**Supplementary Table 8.** Lower wings of the catalyst **4g**

| Atom | δ | J | COSY | HSQC | $^{n}$J-HMQC | ROESY | $^{1}$H,$^{19}$F-HOESY |
|---|---|---|---|---|---|---|---|
| C1b | 123.81 | m (AA'X spin system; $^{3}J_{CP}$, $^{5}J_{CP}$) | | | 4b, 10b | | |
| C2b | 143.31 | m (AA'X spin system; $^{2}J_{CP}$, $^{4}J_{CP}$) | | | 4b | | |
| C3b | 134.16 | m (AA'X spin system; $^{3}J_{CP}$, $^{5}J_{CP}$) | | | 12b, 24b | | |
| C4b | 131.74 | | | 4b | 7b | | |
| H4b | 7.825 | s | | 4b | 1b, 2b, 6b, 7b, 11b | 7b, (12b), 24b | |
| C5b | 132.20 | | | | 8b, 10b | | |
| C6b | 132.24 | | | | 4b, 7b, 9b | | |
| C7b | 128.85 | | | 7b | 4b, 9b | | |
| H7b | 8.274 | d 8.3(8b) | 8b, 9b | 7b | 4b, 6b, 9b | 4b | |
| C8b | 127.22 | | | 8b | 10b | | |
| H8b | 7.885 | m | 7b, 9b | 8b | 5b, 10b | | |
| C9b | 127.56 | | | 9b | 7b | | |
| H9b | 7.770 | d 8.6(10b), d 7.0(8b), d 1.3(7b) | 7b, 8b, 10b | 9b | 6b, 7b | | |
| C10b | 127.34 | | | 10b | 8b | | |
| H10b | 7.901 | m | 9b | 10b | 1b, 5b, 8b | 23b' | |
| C11b | 133.54 | | | | 4b, 23b | | |
| C12b | 124.25 | | | 12b | 24b | | |
| H12b | 8.870 | s | 15b, 21b, 23b, 24b | 12b | 3b, 14b, 22b, 24b | (4b), 15b | 12b |
| C13b | 129.90 | | | | 15b, 21b, 23b | | |
| C14b | 130.40 | | | | 12b, 16b, 18b, 20b | | |
| C15b | 123.53 | | | 15b | 17b | | |
| H15b | 8.666 | d 8.4(16b) | 12b, 16b, 17b, 18b | 15b | 13b, 17b, 19b | 12b | 15b |
| C16b | 126.62 | | | 16b | 18b | | |
| H16b | 7.638 | m | 15b, 17b, 18b | 16b | 14b, 18b | | (16b) |
| C17b | 126.90 | | | 17b | 15b | | |
| H17b | 7.697 | d 7.8(18b), d 7.1(16b), d 1.3(15b) | 15b, 16b, 18b | 17b | 15b, 19b | | |
| C18b | 128.67 | | | 18b | 16b, 20b | | |
| H18b | 7.885 | m | 15b, 16b, 17b | 18b | 14b, 16b, 20b | 20b | |
| C19b | 132.46 | | | | 15b, 17b, 21b | | |
| C20b | 127.95 | | | 20b | 18b | | |
| H20b | 7.644 | m | 21b | 20b | 14b, 18b, 22b | 18b | |
| C21b | 126.25 | | | 21b | 23b | | |
| H21b | 7.438 | d 9.3(20b) | 12b, 20b | 21b | 13b, 19b, 23b | 23b | |
| C22b | 131.29 | | | | 12b, 20b, 24b | | |
| C23b | 128.20 | | | 23b | 21b | | |
| H23b | 6.892 | d 8.3(24b) | 12b, 24b | 23b | 11b, 13b, 21b | 10b', 21b | |
| C24b | 127.13 | | | 24b | 12b | | |
| H24b | 5.936 | d 8.3(23b), d 1.7(12b) | 12b, 23b | 24b | 3b, 12b, 22b | 4b | |

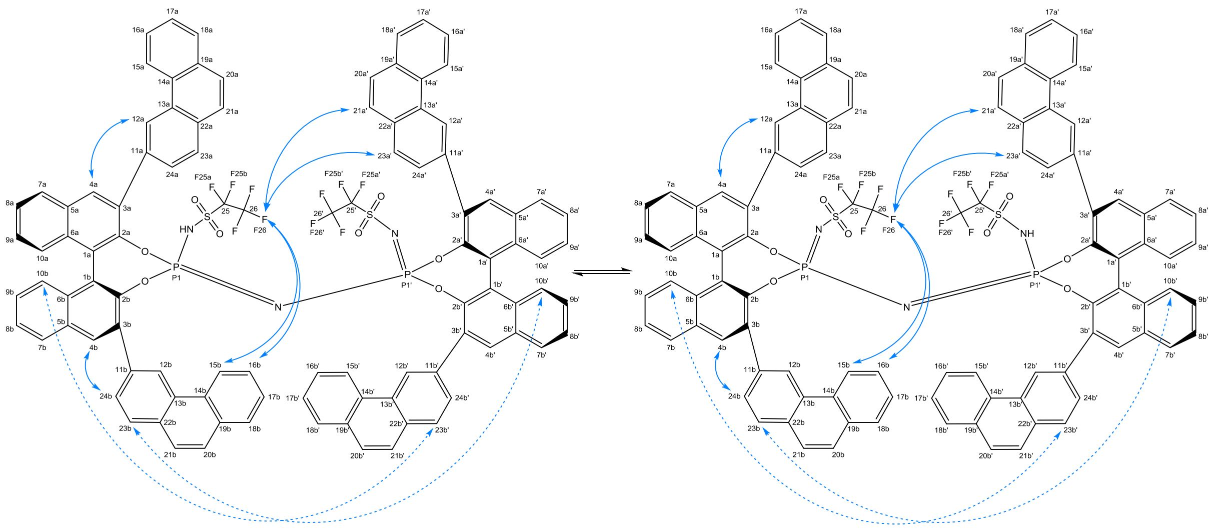

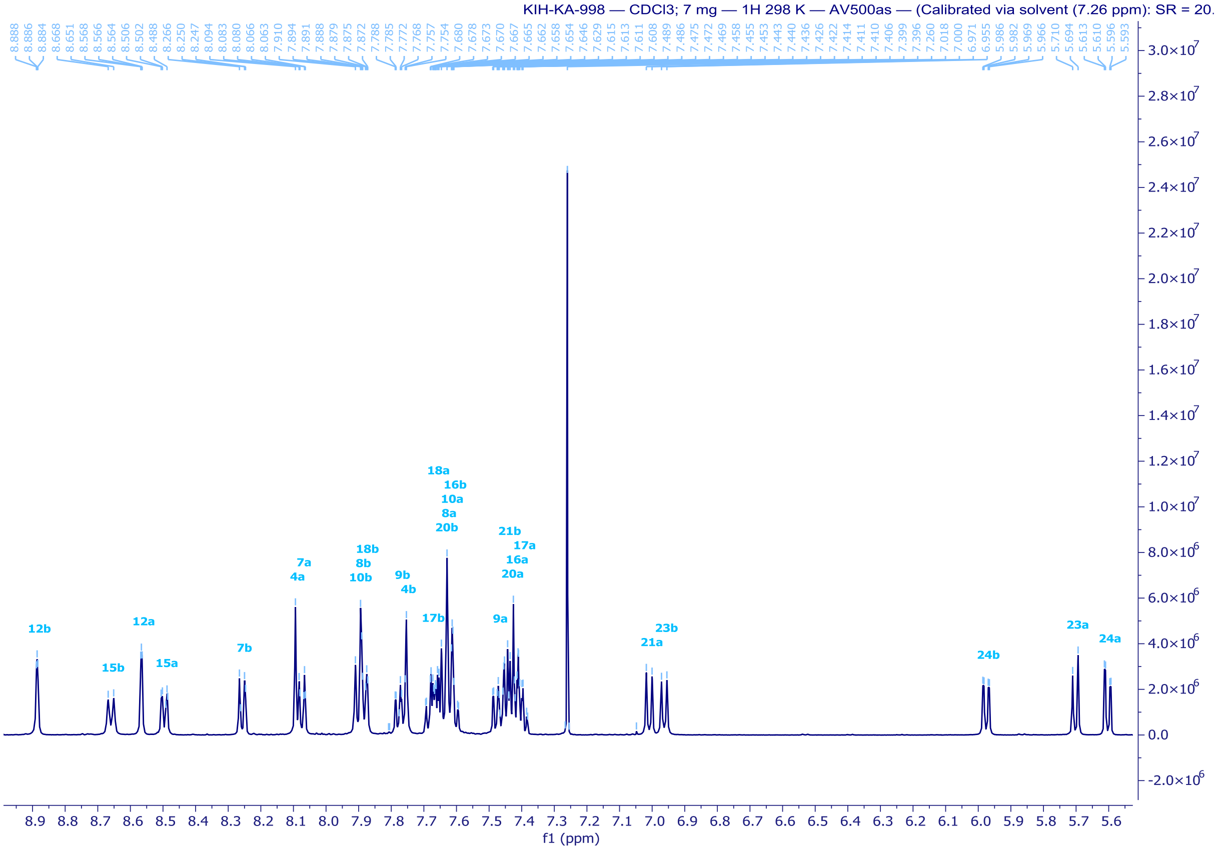


**Supplementary Figure 65.** Structure (up) and $^{1}$H NMR spectrum (down) of **4h**

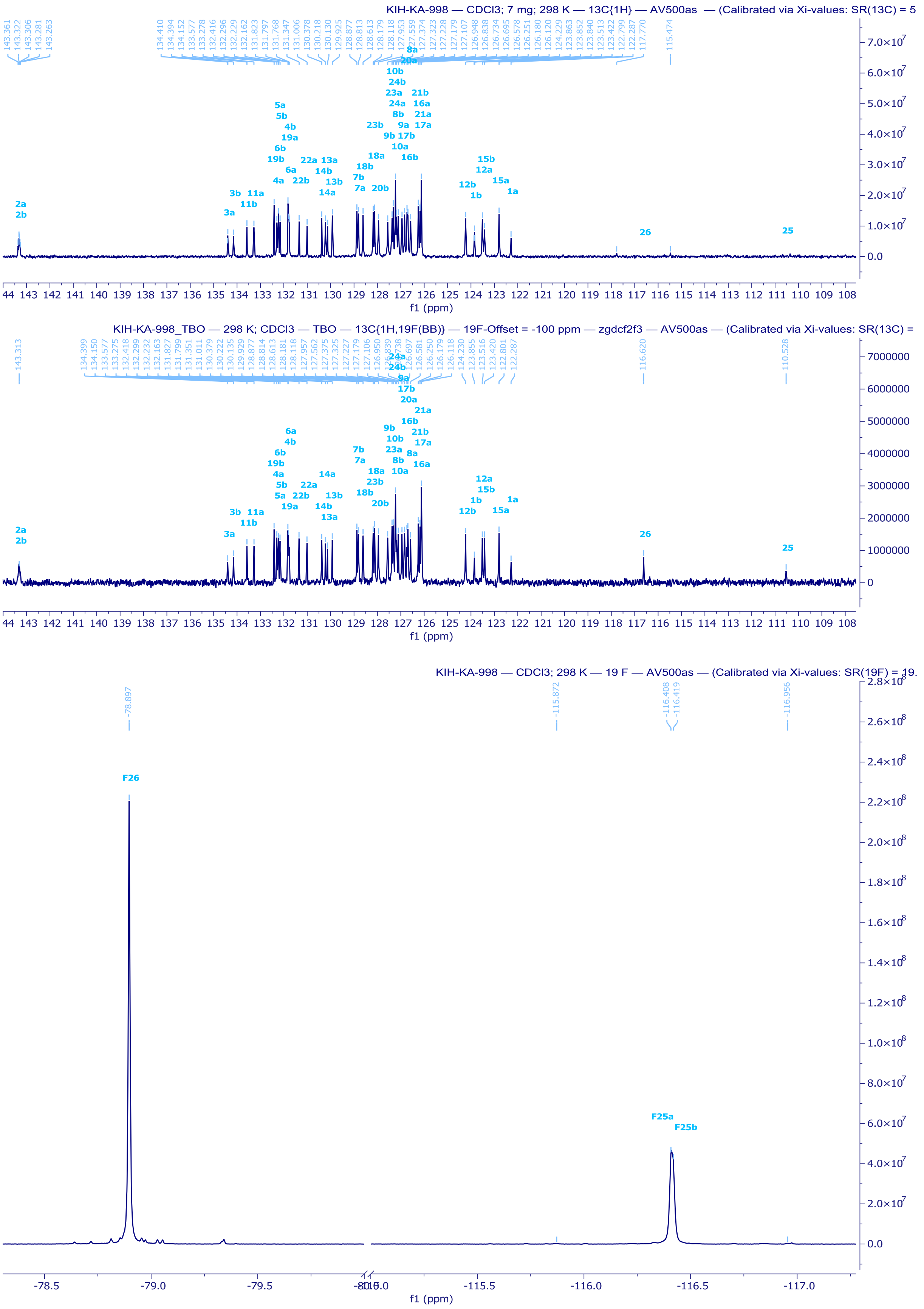


**Supplementary Figure 66.** $^{13}$C NMR (up) and $^{19}$F NMR (down) spectra of **4h**

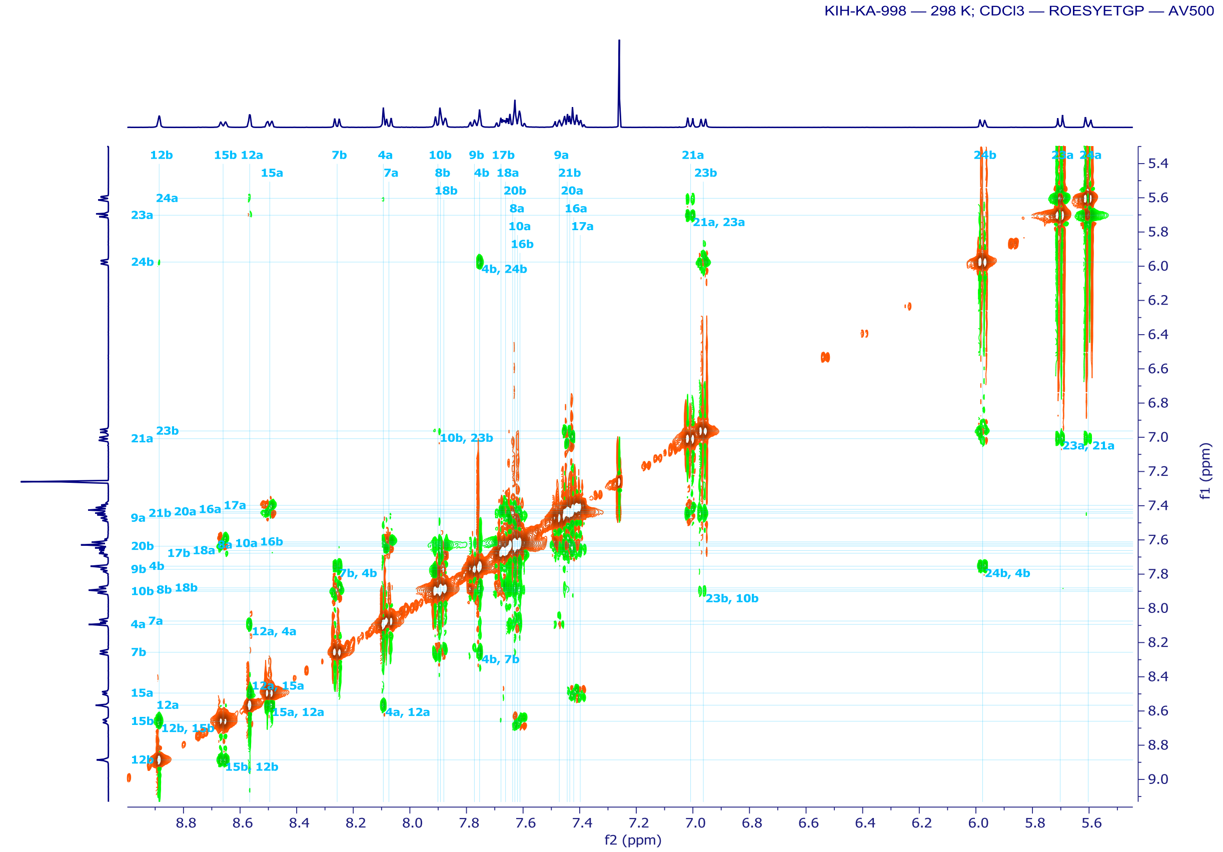


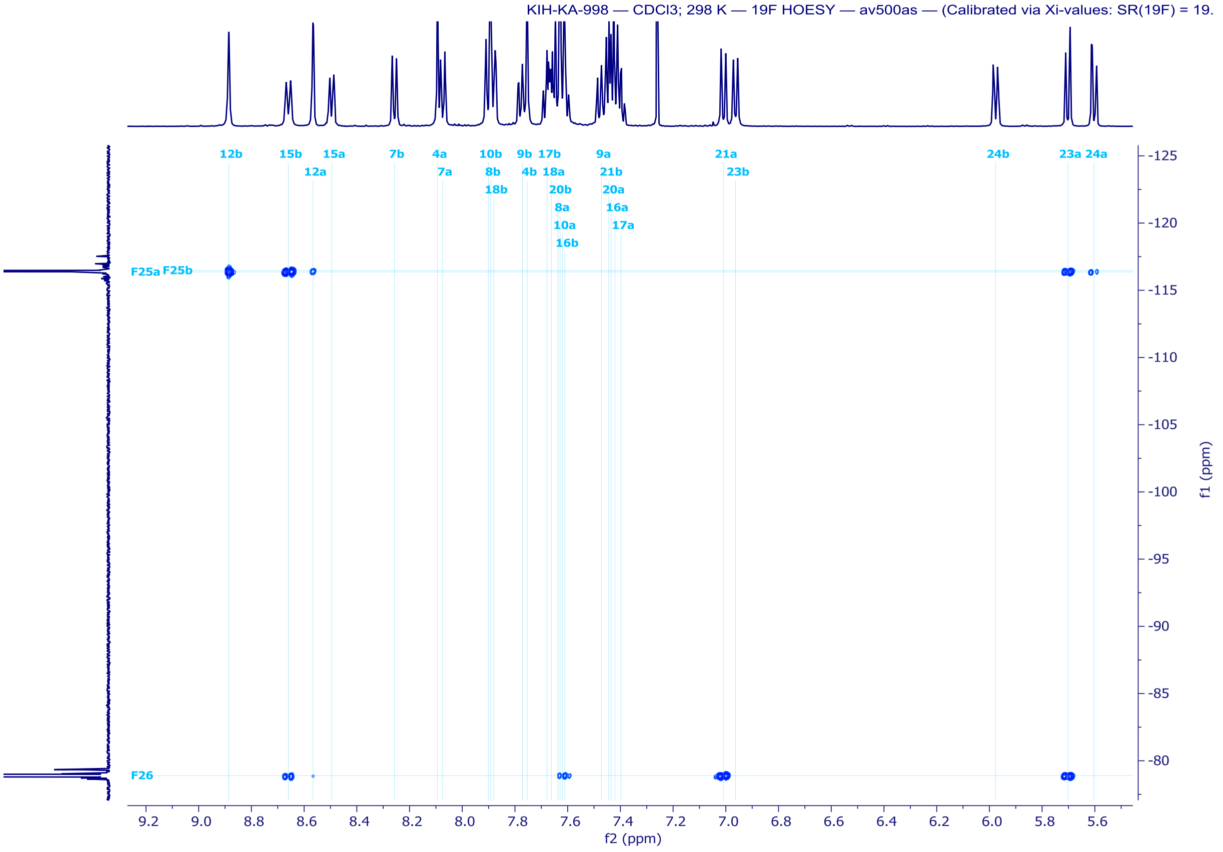


**Supplementary Figure 67.** ROESY (up) and $^{1}H,^{19}F$-HOESY (down) spectra of **4h**

**Supplementary Table 9.** Upper wings and midsection of the catalyst **4h**

| Atom | δ | J | COSY/$^{19}$F-COSY | HSQC/$^{19}$F,$^{13}$C-HSQC | HMQC | ROESY/HF-HOESY |
|---|---|---|---|---|---|---|
| C1a | 122.29 | m (AA'X spin system; $^3J_{CP}$, $^5J_{CP}$) | | | 4a, 10a | |
| C2a | 143.32 | m (AA'X spin system; $^2J_{CP}$, $^4J_{CP}$) | | | 4a | |
| C3a | 134.40 | m (AA'X spin system; $^3J_{CP}$, $^5J_{CP}$) | | | 12a, 24a | |
| C4a | 132.30 | | | 4a | 7a | |
| H4a | 8.094 | s | 10a | 4a | 1a, 2a, 6a, 7a, 11a | 12a |
| C5a | 132.23 | | | | 8a, 10a | |
| C6a | 131.77 | | | | 4a, 7a, 9a | |
| C7a | 128.81 | | | 7a | 4a, 9a | |
| H7a | 8.074 | d 8.3 Hz (H8a) | 8a, 9a | 7a | 4a, 6a, 9a | |
| C8a | 126.58 | | | 8a | 10a | |
| H8a | 7.629 | m | 7a | 8a | 5a, 10a | |
| C9a | 126.95 | | | 9a | 7a | |
| H9a | 7.472 | 3d 8.7 Hz (H10a), 6.9 Hz (H8a), 1.2 Hz (H7a) | 7a, 10a | 9a | 6a, 7a | |
| C10a | 127.11 | | | 10a | 8a | |
| H10a | 7.620 | m | 4a, 9a | 10a | 1a, 5a, 8a | |
| C11a | 133.28 | | | | 4a, 23a | |
| C12a | 123.51 | | | 12a | 12a, 23a, 24a | |
| H12a | 8.566 | s(m) | 15a, 21a, 23a, 24a | 12a | 3a, 12a, 14a, 22a, 24a | 4a, 15a, (F25a, F25b, F26) |
| C13a | 130.13 | | | | 15a, 21a, 23a | |
| C14a | 130.22 | | | | 12a, 16a, 18a | |
| C15a | 122.80 | | | 15a | 17a | |
| H15a | 8.495 | m | 12a, 16a, 17a, 18a | 15a | 13a, 17a, 19a | 12a |
| C16a | 126.18 | | | 16a | 18a | |
| H16a | 7.421 | m | 15a | 16a | 14a, 18a | |
| C17a | 126.12 | | | 17a | 15a | |
| H17a | 7.398 | m | 15a, 18a | 17a | 15a, 19a | |
| C18a | 128.12 | | | 18a | 16a | |
| H18a | 7.662 | m | 15a, 17a | 18a | 14a, 16a, 20a | |
| C19a | 131.82 | | | | 15a, 17a, 21a | |
| C20a | 126.73 | | | 20a | 18a | |
| H20a | 7.435 | m | 21a | 20a | 22a | |
| C21a | 126.12 | | | 21a | 23a | |
| H21a | 7.008 | d 8.9 Hz (H20a) | 12a, 20a, 23a | 21a | 13a, 19a, 22a, 23a | 23a, (F25a, F25b), F26 |
| C22a | 131.01 | | | | 12a, 20a, 21a, 23a, 24a | |
| C23a | 127.37 | | | 23a | 21a | |
| H23a | 5.702 | d 8.4 Hz (H24a) | 12a, 21a, 24a | 23a | 11a, 12a, 13a, 21a, 22a | 21a, F25a, F25b, F26 |
| C24a | 127.23 | | | 24a | 12a | |
| H24a | 5.603 | dd 8.4 Hz (H23a), 1.6 Hz (H12a) | 12a, 23a | 24a | 3a, 12a, 22a | F25a, F25b |
| C25 | 110.53 | tq 296 Hz (F25a,F25b), 41 Hz (F26) | | F25a, F25b | | |
| F25a | -116.36 | d 253 Hz (F25a; AB-System) | F26 | C25 | | (12a), 12b, 15b, (21a), 23a, 24a |
| F25b | -116.47 | d 253 Hz (F25b; AB-System) | F26 | C25 | | (12a), 12b, 15b, (21a), 23a, 24a |
| C26 | 116.62 | qt 289 Hz (F26), ~32 Hz (F25a,F25b) | | | | |
| F26 | -78.90 | s | F25a, F25b | | | (12a, 12b), 15b, 16b, 21a, 23a |
| P1 | -15.49 | | | | | |

**Supplementary Table 10.** Lower wings of the catalyst **4h**

| Atom | δ | J | COSY/$^{19}$F-COSY | HSQC/$^{19}$F,$^{13}$C-HSQC | HMQC | ROESY/HF-HOESY |
|---|---|---|---|---|---|---|
| C1b | 123.85 | m (AA'X spin system; $^{3}J_{CP}$, $^{5}J_{CP}$) | | | 4b, 10b | |
| C2b | 143.31 | m (AA'X spin system; $^{2}J_{CP}$, $^{4}J_{CP}$) | | | 4b | |
| C3b | 134.15 | m (AA'X spin system; $^{3}J_{CP}$, $^{5}J_{CP}$) | | | 12b, 24b | |
| C4b | 131.80 | | | 4b | 7b | |
| H4b | 7.754 | s | | 4b | 1b, 2b, 6b, 7b, 11b | 7b, 24b |
| C5b | 132.16 | | | | 8b, 10b | |
| C6b | 132.23 | | | | 4b, 7b, 9b | |
| C7b | 128.88 | | | 7b | 4b, 9b | |
| H7b | 8.257 | d 7.9 Hz (H8b) | 8b, 9b | 7b | 4b, 6b, 9b | 4b |
| C8b | 127.18 | | | 8b | 10b | |
| H8b | 7.892 | m | 7b, 9b | 8b | 5b, 10b | |
| C9b | 127.56 | | | 9b | 7b | |
| H9b | 7.772 | m | 7b, 8b, 10b | 9b | 6b, 7b | |
| C10b | 127.32 | | | 10b | 8b | |
| H10b | 7.901 | m | 9b | 10b | 1b, 5b, 8b | 23b' |
| C11b | 133.58 | | | | 4b, 23b | |
| C12b | 124.23 | | | 12b | 12b, 23b, 24b | |
| H12b | 8.886 | s(m) | 15b, 21b, 23b, 24b | 12b | 3b, 12b, 14b, 22b, 24b | 15b, F25a, F25b, (F26) |
| C13b | 129.93 | | | | 15b, 21b, 23b | |
| C14b | 130.38 | | | | 12b, 16b, 18b | |
| C15b | 123.42 | | | 15b | 17b | |
| H15b | 8.660 | d 8.4 Hz (H16b) | 12b, 16b, 17b, 18b | 15b | 13b, 17b, 19b | 12b, F25a, F25b, F26 |
| C16b | 126.70 | | | 16b | 18b | |
| H16b | 7.611 | m | 15b, 17b, 18b | 16b | 14b, 18b | F26 |
| C17b | 126.84 | | | 17b | 15b | |
| H17b | 7.678 | m | 15b, 16b, 18b | 17b | 15b, 19b | |
| C18b | 128.61 | | | 18b | 16b | |
| H18b | 7.880 | m | 15b, 16b, 17b | 18b | 14b, 16b, 20b | |
| C19b | 132.42 | | | | 15b, 17b, 21b | |
| C20b | 127.95 | | | 20b | 18b | |
| H20b | 7.637 | m | 21b | 20b | 22b | |
| C21b | 126.25 | | | 21b | 23b | |
| H21b | 7.444 | m | 12b, 20b | 21b | 13b, 19b, 22b, 23b | |
| C22b | 131.35 | | | | 12b, 20b, 21b, 24b | |
| C23b | 128.18 | | | 23b | 21b | |
| H23b | 6.963 | d 8.3 Hz (H24b) | 12b, 24b | 23b | 11b, 12b, 13b, 21b | 10b' |
| C24b | 127.23 | | | 24b | 12b | |
| H24b | 5.976 | dd 8.3 Hz (H23b), 1.7 Hz (H12b) | 12b, 23b | 24b | 3b, 12b, 22b | 4b |

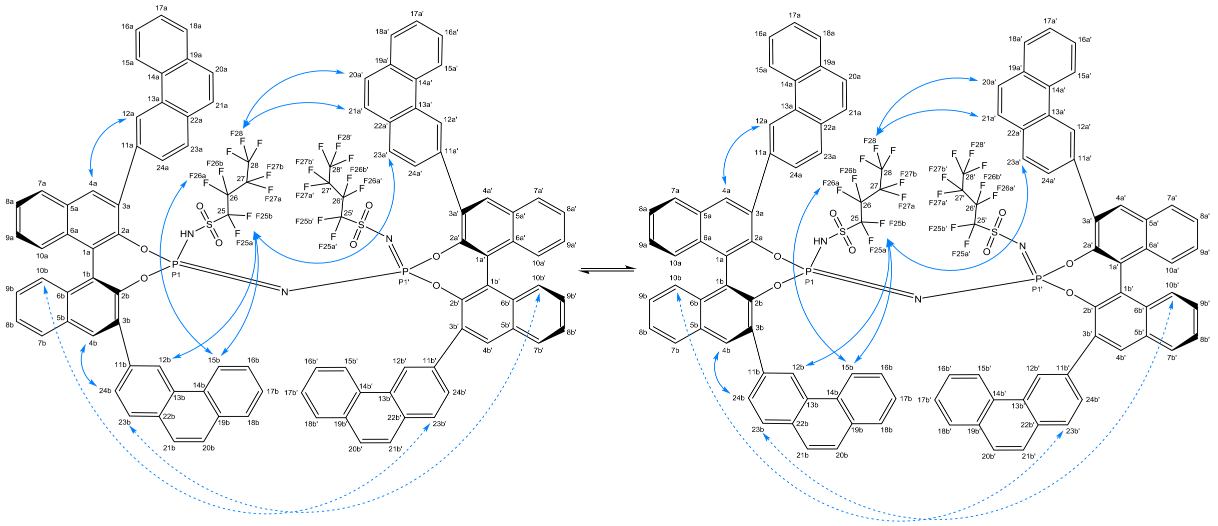

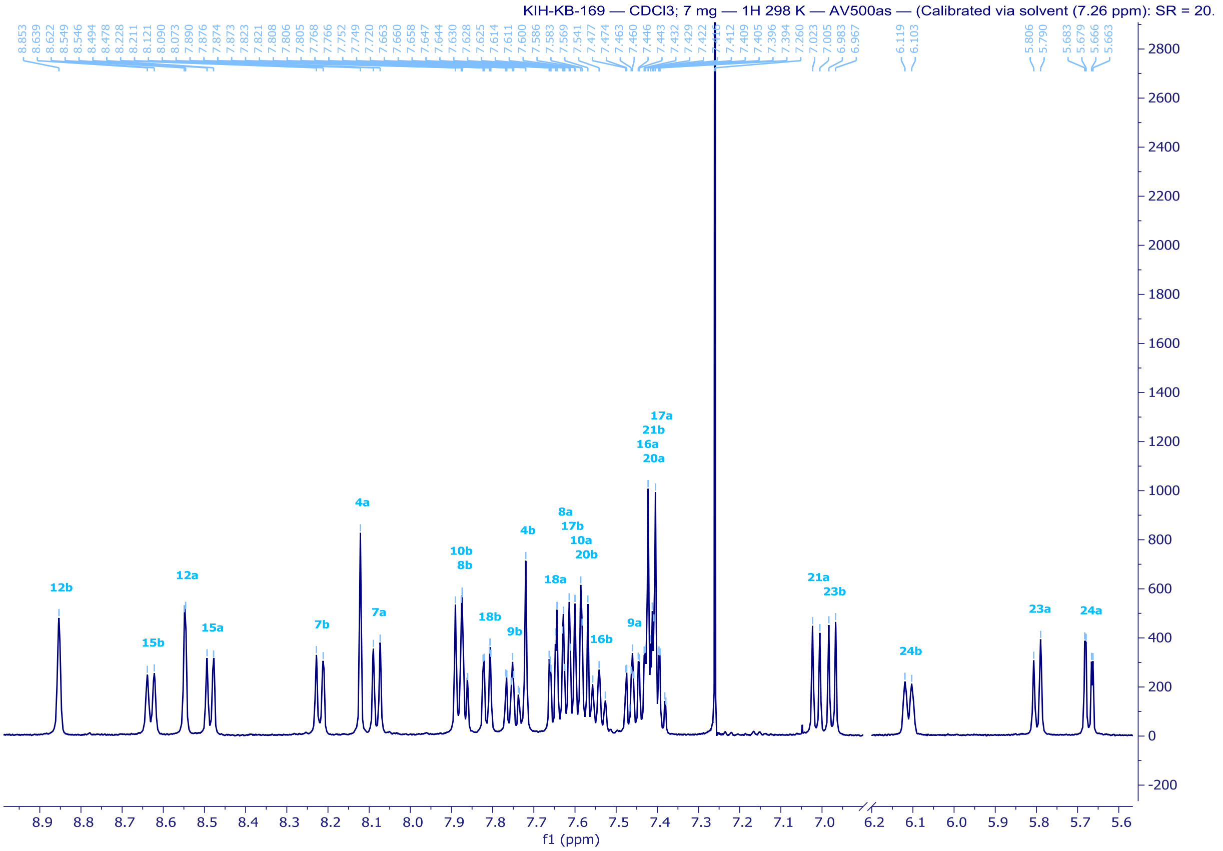

**Supplementary Figure 68.** Structure (up) and $^{1}$H NMR spectrum (down) of **4i**

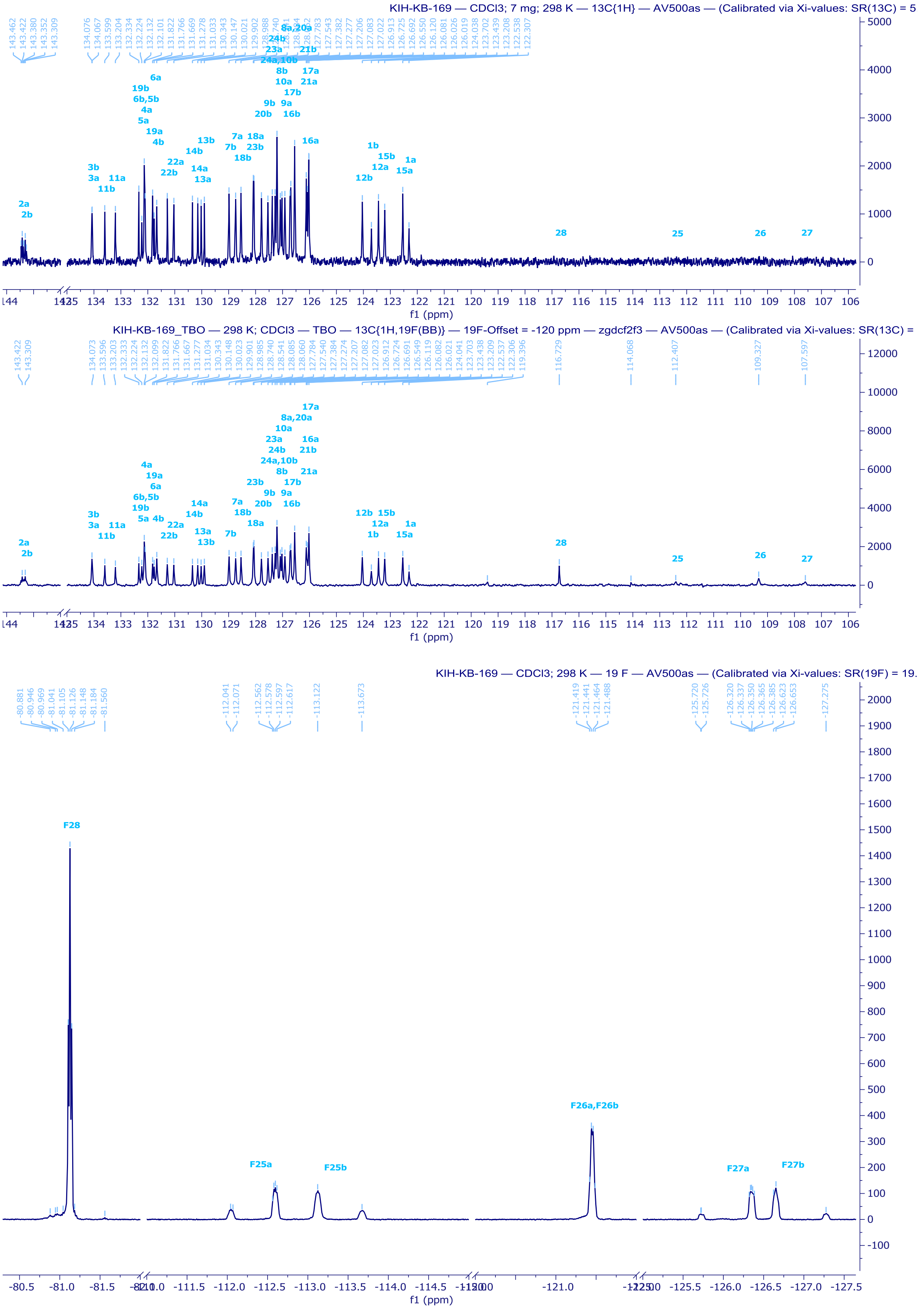


**Supplementary Figure 69.** $^{13}$C NMR (up) and $^{19}$F NMR (down) spectra of **4i**

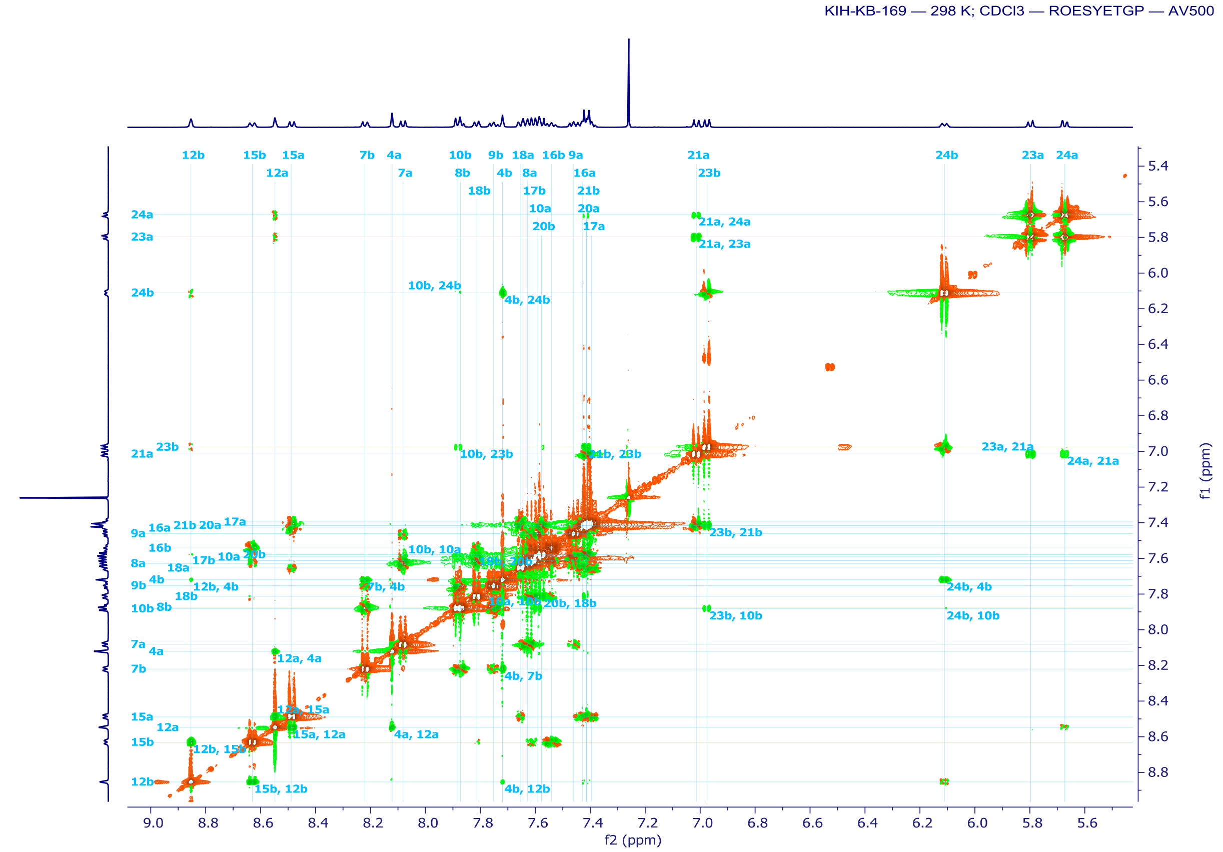


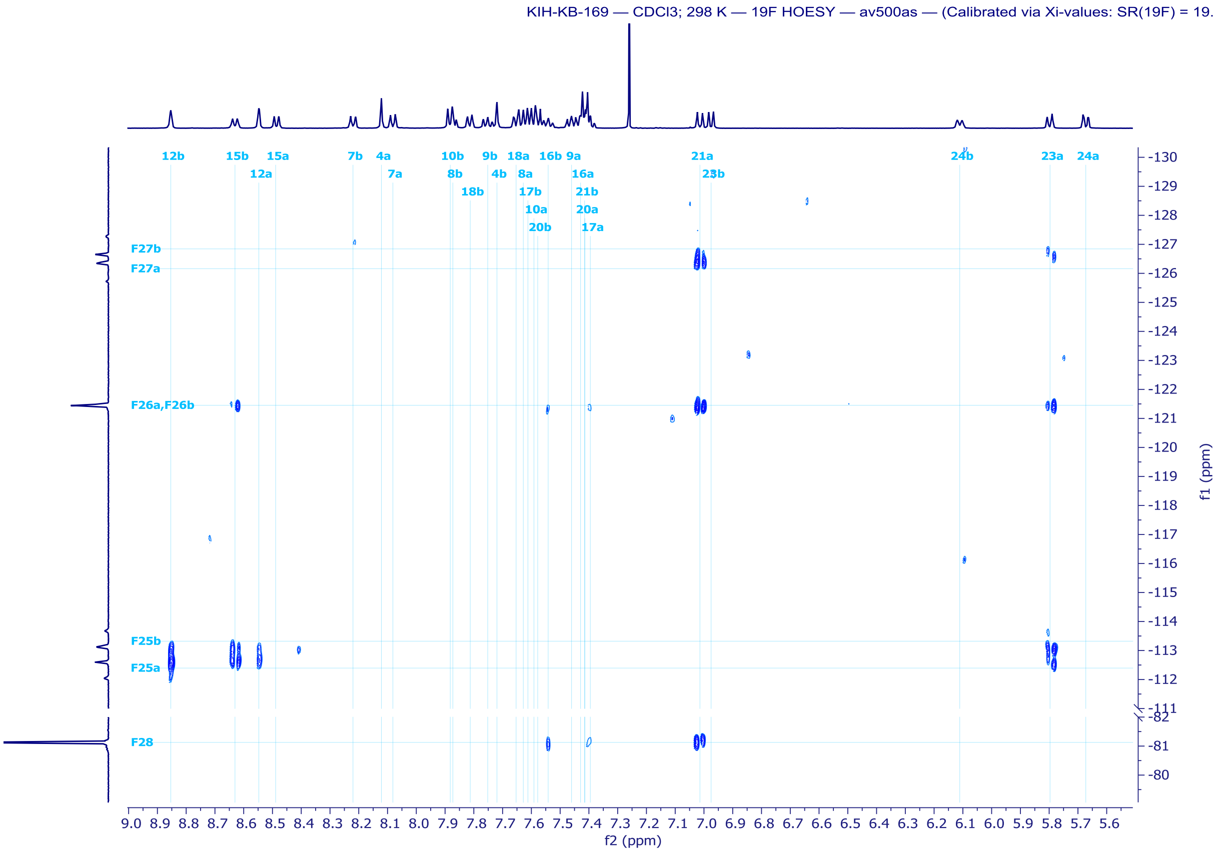


**Supplementary Figure 70.** ROESY (up) and $^{1}H$,$^{19}F$-HOESY (down) spectra of **4i**

## **Supplementary Table 11.** Upper wings and midsection of the catalyst **4i**

| Atom | δ [ppm] | J [Hz] | COSY/$^{19}$F-COSY | HSQC/F,C-HSQC | HMQC/$^{31}$P-HMQC/F,C-($^{n}$J)-HSQC | ROESY/$^{19}$F-ROESY/H,F-HOESY |
|---|---|---|---|---|---|---|
| C1a | 122.31 | m (AA'X spin system; $^3J_{CP}$, $^5J_{CP}$) | | | 4a, 10a | |
| C2a | 143.42 | m (AA'X spin system; $^2J_{CP}$, $^4J_{CP}$) | | | 4a | |
| C3a | 134.07 | m (AA'X spin system; $^3J_{CP}$, $^5J_{CP}$) | | | 12a, 24a | |
| C4a | 132.10 | | | 4a | | |
| H4a | 8.121 | s | 7a, 10a | 4a | 1a, 2a, 6a, 7a, 11a, P1 | 12a |
| C5a | 132.22 | | | | 8a, 10a | |
| C6a | 131.77 | | | | 4a, 7a, 9a | |
| C7a | 128.74 | | | 7a | 4a, 9a | |
| H7a | 8.081 | d 8.3 (H8a) | 4a, 8a, 9a | 7a | 6a, 9a | |
| C8a | 126.55 | | | 8a | 9a, 10a | |
| H8a | 7.628 | m | 7a, 9a | 8a | 5a, 10a | |
| C9a | 126.91 | | | 9a | 7a, 9a | |
| H9a | 7.460 | ddd 8.5 (H10a), 6.9 (H8a), 1.3 (H7a) | 7a, 8a, 10a | 9a | 6a, 7a, 8a, 9a | |
| C10a | 127.02 | | | 10a | 8a | |
| H10a | 7.591 | m | 4a, 9a | 10a | 1a, 5a, 8a, P1 | 10b |
| C11a | 133.20 | | | | 4a, 23a | |
| C12a | 123.44 | | | 12a | 12a, 24a | |
| H12a | 8.548 | d 1.7 (H24) | 15a, 21a, 23a, 24a | 12a | 3a, 12a, 14a, 22a, 24a | 4a, 15a, (F25a,b) |
| C13a | 130.02 | | | | 15a, 21a, 23a | |
| C14a | 130.15 | | | | 12a, 18a, 20a | |
| C15a | 122.54 | | | 15a | 17a | |
| H15a | 8.489 | d 8.0 (H16a) | 12a, 16a, 17a, 18a | 15a | 13a, 17a, 19a | 12a |
| C16a | 126.03 | | | 16a | 18a | |
| H16a | 7.429 | m | 15a, 17a, 18a | 16a | 18a | |
| C17a | 126.02 | | | 17a | 15a | |
| H17a | 7.395 | m | 15a, 16a, 18a | 17a | 15a, 19a | |
| C18a | 128.06 | | | 18a | 16a, 20a | |
| H18a | 7.653 | m | 15a, 16a, 17a | 18a | 14a, 16a, 20a | |
| C19a | 131.82 | | | | 15a, 17a, 21a | |
| C20a | 126.55 | | | 20a | 18a | |
| H20a | 7.414 | m | 21a | 20a | 14a, 18a, 22a | F26a,b, F28 |
| C21a | 126.08 | | | 21a | 23a | |
| H21a | 7.014 | d 8.8 (H20a) | 12a, 20a, 23a | 21a | 13a, 19a, 22a, 23a | 23a, 24a, F26a,b, F27a,b, F28 |
| C22a | 131.03 | | | | 12a, 20a, 21a, 23a, 24a | |
| C23a | 127.38 | | | 23a | 21a, 23a | |
| H23a | 5.797 | d 8.4 (H24a) | 12a, 21a, 24a | 23a | 11a, 13a, 21a, 22a, 23a | 21a, F25a,b, F26a,b, F27a,b |
| C24a | 127.21 | | | 24a | 12a | |
| H24a | 5.673 | dd 8.4 (H23a), 1.7 (H12a) | 12a, 23a | 24a | 3a, 12a, 22a | 21a |
| C25 | 112.4 (eqv) | s ($^{19}$F-decoupled) | | | | |
| F25a | -112.40 | d 259 (F25b; AB-System) | F27a,b | | | F26a,b, H12b, H15b, (H12a), H23a |
| F25b | -113.32 | d 259 (F25a; AB-System) | F27a,b | | | F26a,b, H12b, H15b, (H12a), H23a |
| C26 | 109.33 | s ($^{19}$F-decoupled) | | F26a,b | | |
| F26a, F26b | -121.45 | q 10.2 (F28) | F28, F27a,b | C26 | | F28, F27a,b, F25a,b, H15b, (H16b), H20a, H21a, H23a |
| C27 | 107.6 (eqv) | s ($^{19}$F-decoupled) | | | (F28) | |
| F27a | -126.16 | d 293 (F27b; AB-System) | F25a,b, F26a,b | | | F28, F26a,b, H21a, H23a |
| F27b | -126.84 | d 293 (F27a; AB-System) | F25a,b, F26a,b | | | F28, F26a,b, H21a, H23a |
| C28 | 116.73 | s ($^{19}$F-decoupled) | | F28 | | |
| F28 | -81.13 | t 10.2 (F26a,b) | F26a,b | C28 | (C27) | F26a,b, F27a,b, (H16b), H20a, H21a |
| P1 | -14.44 | | | | H4a, H4b, H10a | |

**Supplementary Table 12.** Lower wings of the catalyst **4i**

| Atom | δ [ppm] | J [Hz] | COSY | HSQC | HMQC/$^{31}$P-HMQC | ROESY/$^{19}$F-ROESY/H,F-HOESY |
|---|---|---|---|---|---|---|
| C1b | 123.70 | m (AA'X spin system; $^3J_{CP}$, $^5J_{CP}$) | | | 4b, 10b | |
| C2b | 143.31 | m (AA'X spin system; $^2J_{CP}$, $^4J_{CP}$) | | | 4b | |
| C3b | 134.08 | m (AA'X spin system; $^3J_{CP}$, $^5J_{CP}$) | | | 12b, 24b | |
| C4b | 131.67 | | | 4b | 7b | |
| H4b | 7.720 | s | 7b, 10b | 4b | 1b, 2b, 6b, 7b, 11b, P1 | 7b, (12b), 24b |
| C5b | 132.13 | | | | 8b, 10b | |
| C6b | 132.13 | | | | 4b, 7b, 9b | |
| C7b | 128.99 | | | 7b | 4b, 9b | |
| H7b | 8.220 | d 8.3 (H8b) | 4b, 8b, 9b | 7b | 4b, 6b, 9b | 4b |
| C8b | 127.08 | | | 8b | 10b | |
| H8b | 7.873 | m | 7b, 9b | 8b | 5b, 10b | |
| C9b | 127.54 | | | 9b | 7b | |
| H9b | 7.752 | ddd 8.5 (H10b), 6.9 (H8b), 1.3 (H7b) | 7b, 8b, 10b | 9b | 6b, 7b | |
| C10b | 127.21 | | | 10b | 8b | |
| H10b | 7.882 | m | 4b, 9b | 10b | 1b, 5b, 8b | 10a, 23b, 24b |
| C11b | 133.60 | | | | 4b, 23b | |
| C12b | 124.04 | | | 12b | 24b | |
| H12b | 8.853 | s (m) | 15b, 21b, 23b, 24b | 12b | 3b, 14b, 22b, 24b | (4b), 15b, F25a,b |
| C13b | 129.90 | | | | 15b, 21b, 23b | |
| C14b | 130.34 | | | | 12b, 16b, 18b, 20b | |
| C15b | 123.21 | | | 15b | 17b | |
| H15b | 8.630 | d 8.4 (H16b) | 12b, 16b, 17b, 18b | 15b | 13b, 17b, 19b | 12b, F25a,b, F26a,b |
| C16b | 126.72 | | | 16b | 18b | |
| H16b | 7.542 | m | 15b, 17b, 18b | 16b | 14b, 18b | (F26a,b), (F28) |
| C17b | 126.69 | | | 17b | 15b | |
| H17b | 7.612 | m | 15b, 16b, 18b | 17b | 15b, 19b | |
| C18b | 128.54 | | | 18b | 16b, 20b | |
| H18b | 7.813 | dd 8.0 (H17b), 1.3 (H16b) | 15b, 16b, 17b | 18b | 14b, 16b, 20b | 20b |
| C19b | 132.33 | | | | 15b, 17b, 21b | |
| C20b | 127.78 | | | 20b | 18b, 20b, 21b | |
| H20b | 7.578 | m | 21b | 20b | 14b, 18b, 20b, 21b, 22b | 18b |
| C21b | 126.12 | | | 21b | 20b, 21b, 23b | |
| H21b | 7.415 | m | 12b, 20b | 21b | 13b, 19b, 20b, 21b, 22b, 23b | 23b |
| C22b | 131.28 | | | | 12b, 20b, 21b, 24b | |
| C23b | 128.08 | | | 23b | 21b | |
| H23b | 6.975 | d 8.3 (H24b) | 12b, 24b | 23b | 11b, 13b, 21b | 10b, 21b |
| C24b | 127.28 | | | 24b | 12b | |
| H24b | 6.111 | d 8.3 (H23b) | 12b, 23b | 24b | 3b, 12b, 22b | 4b, 10b |

**Supplementary Discussion 1.** Molecular Dynamics Simulations

Molecular dynamics simulations were performed using the software GROMACS (v. 2016.4)[14] and the CHARMM general force field (CGenFF, v. 4.0).[15–17] The topology of the molecule was generated from the starting structure (CCDC code SEFWAE[2]) using the CGenFF ParaChem server (https://cgenff.paramchem.org/, interface v. 1.0.0, force field v. 3.0.1), and then modified manually to ensure the structure remained consistent with crystallography data (CCDC code SEFWAE). Specifically, the central P–N–P angle was set to 170° (vs. 135° in the original Parachem topology), the N–P–N–P and P–N–P–N dihedral angles were set to 180° (by fixing the improper angle defined by N–P(–N)–P–N to 180°), and the corresponding P–N and N–P bond distances were set to 1.50 Å (vs. 1.79 Å). Furthermore, the core of the molecule was stabilized by specifically defining an H-bond between H0 and O52 and setting the corresponding S–N–H–P improper dihedral angle to 0°.

The molecule was placed in a box of 396 trichloromethane molecules,[18] ensuring a minimum distance of 10 Å between of any IDPi atom and the edge of the box. All bonds were constrained using the LINCS method[19] and the timestep was 2 fs. The Verlet cutoff scheme[20] was used for the neighbor lists (10 Å short-range cutoffs for both Coulomb and van der Waals forces) and the particle mesh Ewald method[21,22] was employed for electrostatics. The contact information from the measured nuclear Overhauser effects (NOEs) was included as simple bond-type restraints (i.e. time averaging was not considered).

The system was first energy-minimized by steepest descent to a maximum force of 10 $kJ\cdot mol^{-1}\cdot nm^{-1}$, then equilibrated, first in the isothermal ensemble (NVT) at 300 K for 100 ps, then in the isothermal isobaric ensemble (NPT) for 100 ps at 300 K and 1 bar, then (only for the NOE-restrained production run) for an additional 100 ps under the same conditions with the NOE restraints applied. The two production simulations (one with and one without NOE restraints) were then performed for 10 ns in the same NPT ensemble. The temperature and pressure of the system were controlled using a Berendsen thermostat[23] and a Parrinello-Rahman barostat.[24]

Supplementary Table 13 shows that 16/20 of the NOE contacts are already satisfied in the unrestrained simulation. Adding them simply stabilizes the structure—the standard deviations and ranges of the distances are reduced— and ensures that the remaining four restraints are satisfied.

**Supplementary Table 13.** List of nuclear Overhauser effect contacts and the corresponding distances in molecular dynamics simulations. (For atom numbering, refer to Supplementary Figure 71)

| | Connected atoms | Estimated distance | MD distance | Restrained MD distance |
|---|---|---|---|---|
| 1 | H4A–H12B | 3.5 ± 1.5 | 2.6 ± 0.3 [1.9–4.3] | 2.7 ± 0.2 [2.0–3.7] |
| | H4B–H12A | 3.5 ± 1.5 | 2.6 ± 0.2 [1.0–3.8] | 2.8 ± 0.2 [2.0–3.8] |
| 2 | H54A–H74A | 3.5 ± 1.5 | 2.7 ± 0.3 [1.9–4.4] | 2.8 ± 0.3 [2.0–4.0] |
| | H54B–H74B | 3.5 ± 1.5 | 2.5 ± 0.3 [1.9–4.2] | 2.6 ± 0.2 [2.0–3.8] |
| 3 | H60A–H70B | 3.5 ± 1.5 | 3.2 ± 0.5 [2.0–8.0] | 3.1 ± 0.3 [2.2–4.4] |
| | H60B–H70A | 3.5 ± 1.5 | 3.3 ± 0.5 [2.1–6.7] | 3.1 ± 0.3 [2.1–4.6] |
| 4 | H60A–H74B | 4.0 ± 1.5 | 3.4 ± 0.3 [2.4–5.2] | 3.4 ± 0.3 [2.4–5.2] |
| | H60B–H74A | 4.0 ± 1.5 | 3.7 ± 0.5 [2.1–6.6] | 3.5 ± 0.3 [2.4–5.1] |
| 5 | H10A–H74B | 4.0 ± 1.5 | 5.7 ± 0.4 [4.7–9.2] | 5.4 ± 0.2 [4.6–6.5] |
| | H10B–H74A | 4.0 ± 1.5 | 5.7 ± 0.4 [4.7–8.1] | 5.4 ± 0.2 [4.6–6.5] |
| 6 | H59A–H73B | 4.0 ± 1.5 | 4.1 ± 0.6 [2.7–9.1] | 4.0 ± 0.4 [2.8–5.6] |
| | H59B–H73A | 4.0 ± 1.5 | 4.1 ± 0.6 [2.6–8.1] | 4.1 ± 0.4 [2.8–5.4] |
| 7 | H71A–H24B | 4.0 ± 1.5 | 3.0 ± 0.4 [1.9–4.7] | 3.0 ± 0.3 [2.1–4.2] |
| | H71B–H24A | 4.0 ± 1.5 | 3.2 ± 0.4 [2.0–5.4] | 3.2 ± 0.3 [2.2–4.5] |
| 8 | H71A–H23B | 4.0 ± 1.5 | 3.9 ± 0.5 [2.1–6.0] | 3.9 ± 0.4 [2.4–5.8] |
| | H71B–H23A | 4.0 ± 1.5 | 4.3 ± 0.6 [2.0–6.2] | 4.4 ± 0.4 [2.8–5.7] |
| 9 | C25A–H23B | 4.5 ± 1.5 | 4.5 ± 0.5 [3.1–7.2] | 4.6 ± 0.4 [3.4–6.7] |
| | C25B–H23A | 4.5 ± 1.5 | 6.0 ± 0.6 [4.3–9.3] | 5.7 ± 0.4 [4.4–7.4] |
| 10 | C25A–H16A | 4.5 ± 1.5 | 6.9 ± 0.7 [4.5–10.4] | 6.1 ± 0.3 [4.6–7.7] |
| | C25B–H16B | 4.5 ± 1.5 | 6.6 ± 0.5 [5.3–9.0] | 6.0 ± 0.3 [4.9–7.2] |

MD, molecular dynamics.
The values shown are mean ± standard deviation [minimum–maximum].

**Supplementary Discussion 2.** Structure Description

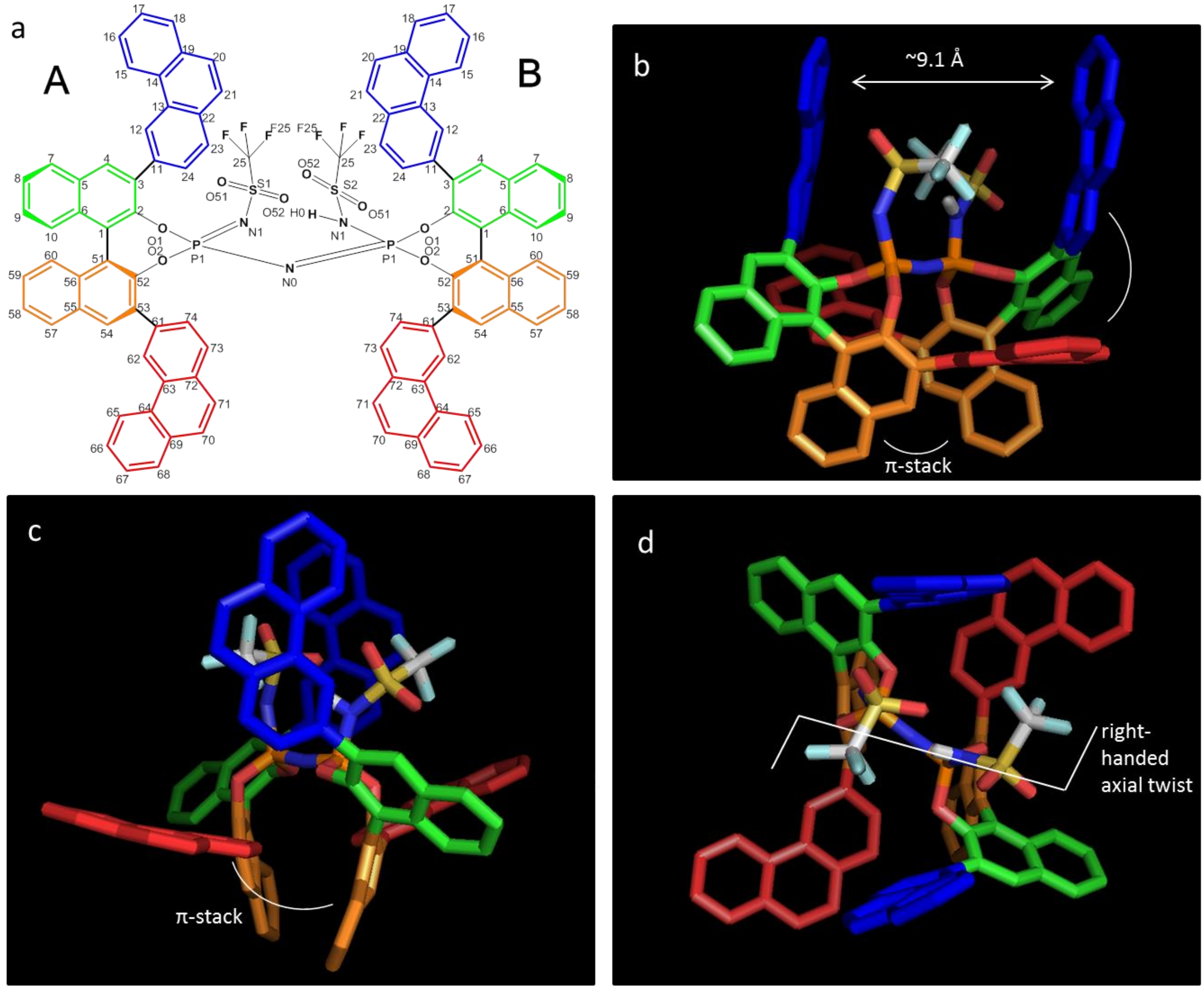


**Supplementary Figure 71**: a) Schematic representation of the pseudo-symmetric **4g** with atom numbering, monomer identification (**A** and B) and color coding of the aromatic moieties. b) Side view #1 of a selected model from the MD trajectory. For clarity, aromatics are color-coded as in (a) and hydrogens are omitted. c) Side view #2: Same as (b), rotated 90° axially. d) Top view: Same as (c), rotated 90° horizontally.

The overall shape of the molecular models extracted from the MD simulations are very similar to those already described by X-ray crystallography (CCDC code AWAHIR, AWAJAL and SEFWAE), albeit with additional interesting features (see Supplementary Figure 71). The two bottom BINOL naphthyl groups (orange, A and B side) meet at the lower side of the structure and stabilize the structure via a parallel-displaced π-stacking interaction (Supplementary Figure 71b). The bottom phenanthryl substituents (red) extend outwards and are rotated so that their outer "convex" edge contacts the bottom naphtyl group (orange) of the opposite side (Supplementary Figure 71d, based on NOE #3, #4 and #6 in Supplementary Table 13) through a T-shape π-stacking. In this position, the face of these bottom phenanthryl groups (red) also interact via a T-shaped π-stacking with the outer convex edge of the upper

phenanthryl group (blue) of the opposite side (Supplementary Figure 71d, based on NOEs #7 and #8 in Supplementary Table 13). In this stabilised conformation, the upper phenanthryl groups (blue) are extended upwards, and parallel to one another to form a boxed, sterically confined access of about 9Å in width to the catalytic phosphoramidimadate center (Supplementary Figure 71b).

The geometry of the N-P-N-P-N moiety, broadly derived from the available crystal structures was well accommodated in the overall fold of the backbone and substituents. The asymmetry of the catalytic pocket is mainly defined by the orientation of the -$CF_3$ groups. In our model, a right-handed axial twist of the S-CF3 bonds about the C2 axis (Supplementary Figure 71c) is defined by the heteronuclear NOEs between the -$CF_3$ and the opposite upper phenanthryl groups (blue) (Supplementary Table 13, NOE #9). According to the MD simulations, this chirality cannot be easily switched due to steric barriers.

It is important to note that although the crystal structures and DFT calculations[25] definitively predict the acid proton to be located on one of the side N, the C2-symmetry -- derived from the magnetically equivalent A and B halves according to the NMR data -- is incompatible with this static situation. Though protonation of the middle N would be congruent with the NMR-observed symmetry, the high energy and the inaccessibility of this proton is compatible neither with our structures nor with the activity of IDPis. Rather, a dynamic model where the $H^+$ is rapidly relayed between both side N positions is proposed. This relay could be assisted by the nearby O52 groups which are known to stabilise the side N-H via H-bonding. Note that N1A, N1B, O52A and O52B are within 3Å of one another in our models. This $H^+$ relay would be accommodated by electronic resonant structures and a slight rotation of the side P-N and N-S bonds as shown in Scheme **S2.**

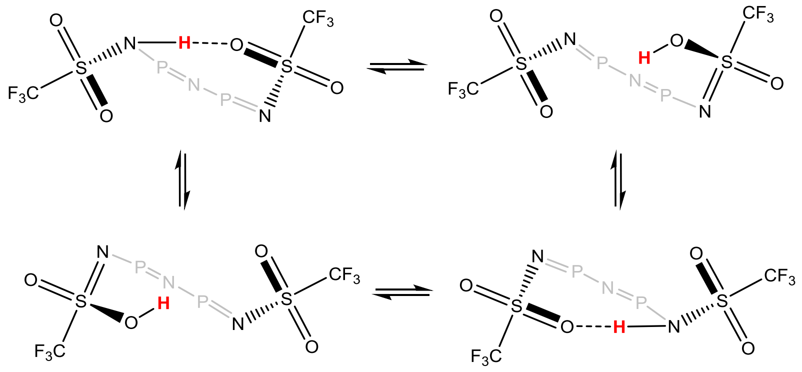

**Supplementary Figure 72.** Depiction of a conceivable $H^+$ relay mechanism compatible the NMR observed C2 symmetry of **4g.**

## Supplementary References


1. Yue, Y.; Turlington, M.; Yu, X. Q. & Pu, L. 3,3′-Anisyl-Substituted BINOL, $H_4$BINOL, and $H_8$BINOL Ligands: Asymmetric Synthesis of Diverse Propargylic Alcohols and Their Ring-Closing Metathesis to Chiral Cycloalkenes. *J. Org. Chem.*, **74**, 8681–8689 (2009).
2. Liu, L., Kim, H., Xie, Y., Farès, C., Kaib, P. S. J., Goddard, R. & List, B. Catalytic Asymmetric [4+2]-Cycloaddition of Dienes with Aldehydes. *J. Am. Chem. Soc.* **139**, 13656–13659 (2017).
3. Erkkilä, A. & Pihko, P. M. Mild Organocatalytic α-Methylenation of Aldehydes. *J. Org. Chem.*, **71**, 2538–2541 (2006).
4. Yatham, V. R.; Harnying, W.; Kootz, D.; Neudörfl, J.; Schlörer, N. E. & Berkessel, A. 1,4-Bis-Dipp/Mes-1,2,4-Triazolylidenes: Carbene Catalysts That Efficiently Overcome Steric Hindrance in the Redox Esterification of α- and β-Substituted α,β-Enals. *J. Am. Chem. Soc.* **138**, 2670–2677 (2016).
5. Hayashi, Y.; Rohde, J. J. & Corey, E. J. A Novel Chiral Super-Lewis Acidic Catalyst for Enantioselective Synthesis. *J. Am. Chem. Soc.* **118**, 5502–5503 (1996).
6. Sprott, K. T. & Corey, E. J. A New Cationic, Chiral Catalyst for Highly Enantioselective Diels−Alder Reactions. *Org. Lett.* **5**, 2465–2467 (2003).
7. Kano, T.; Tanaka, Y.; Osawa, K.; Yurino, T. & Maruoka, K. Catalytic Enantioselective Construction of All-Carbon Quaternary Stereocenters by an Organocatalytic Diels–Alder Reaction of α-Substituted α,β-Unsaturated Aldehydes. *Chem. Commun.* 1956–1958 (2009).
8. Ishihara, K.; Kurihara, H.; Masayuki Matsumoto, M. & Yamamoto, H. Design of Brønsted Acid-Assisted Chiral Lewis Acid (BLA) Catalysts for Highly Enantioselective Diels−Alder Reactions. *J. Am. Chem. Soc.* **120**, 6920–6930 (1998).
9. Ahrendt, K. A.; Borths, C. J. & MacMillan, D. W. C. New Strategies for Organic Catalysis: The First Highly Enantioselective Organocatalytic Diels−Alder Reaction. *J. Am. Chem. Soc.* **122**, 4243–4244 (2000).
10. Xuan, W.-J.; Botuha, C.; Hasenknopf, B. & Thorimbert, S. Chiral Dawson-Type Hybrid Polyoxometalate Catalyzes Enantioselective Diels-Alder Reactions. *Chem. Eur. J.* **21**, 16512–16516 (2015).
11. Chapuis, C.; Skuy, D.; Laumer, J.-Y. & Brauchli, R. *endo*/*exo* Stereoselectivity in Diels–Alder Reactions of *α,β-* Dialkylated Conjugated Enals to Cyclic 1,3- Dienes: Intermediates in the Synthesis of (−)- *β-* Santalol and Its Analogs. *Chemistry & Biodiversity* **11**, 1470–1516 (2014).

12. Zhang, J.; Han, X.; Wu, X.; Liu, Y. & Cui, Y. Multivariate Chiral Covalent Organic Frameworks with Controlled Crystallinity and Stability for Asymmetric Catalysis. *J. Am. Chem. Soc.* **139**, 8277–8285 (2017).
13. Shen, Z.-L.; Cheong, H.-L.; Lai, Y.-C. Loo, W.-Y. & Loh, T.-P. Application of Recyclable Ionic Liquid-Supported Imidazolidinone Catalyst in Enantioselective Diels–Alder Reactions. *Green Chem*. **14**, 2626–2630 (2012).
14. Abraham, M. J.; Murtola, T.; Schulz, R.; Páll, S.; Smith, J. C.; Hess, B. & Lindahl, E. GROMACS: High Performance Molecular Simulations through Multi-Level Parallelism from Laptops to Supercomputers. *SoftwareX* **1**, 19–25 (2015).
15. Vanommeslaeghe, K.; Hatcher, E.; Acharya, C.; Kundu, S.; Zhong, S.; Shim, J.; Darian, E.; Guvench, O.; Lopes, P.; Vorobyov, I. & Mackerell, A.D. CHARMM General Force Field: A Force Field for Drug-Like Molecules Compatible with the CHARMM All-Atom Additive Biological Force Fields. *J. Comput. Chem.* **31**, 671–690 (2010).
16. Vanommeslaeghe, K. & Mackerell, A.D. Automation of the CHARMM General Force Field (CGenFF) I: Bond Perception and Atom Typing. *J. Chem. Inf. Model.* **52**, 3144–3154 (2012).
17. Vanommeslaeghe, K.; Raman, E. P. & Mackerell, A.D. Automation of the CHARMM General Force Field (CGenFF) II: Assignment of Bonded Parameters and Partial Atomic Charges. *J. Chem. Inf. Model.* **52**, 3155–3168 (2012).
18. Fischer, N. M.; van Maaren, P. J.; Ditz, J. C.; Yildirim, A. & van der Spoel, D. Properties of Organic Liquids when Simulated with Long-Range Lennard-Jones Interactions. *J. Chem. Theory Comput.* **11**, 2938–2944 (2015).
19. Hess, B. J P-LINCS: A Parallel Linear Constraint Solver for Molecular Simulation. *J. Chem. Theory Comput.* **4**, 116–122 (2008).
20. Páll, S. & Hess, B. A Flexible Algorithm for Calculating Pair Interactions on SIMD Architectures. *Comput. Phys. Comm.* **184**, 2641–2650 (2013).
21. Darden, T.; York, D. & Pedersen, L.G. Particle mesh Ewald: An $N\cdot\log(N)$ Method for Ewald Sums in Large Systems. *J. Chem. Phys.* **98**, 10089–10092 (1993).
22. Essmann, U.; Perera, L.; Berkowitz, M. L.; Darden, T.; Lee, H. & Pedersen, L. G. A Smooth Particle Mesh Ewald Method. *J. Chem. Phys.* **103**, 8577–8593 (1995).
23. Berendsen H. J.; Postma J. V.; van Gunsteren W. F.; DiNola A. R. & Haak J. R. Molecular Dynamics with Coupling to an External Bath. *J. Chem. Phys.* **81**, 3684–3690 (1984).

24. Parrinello, M. & Rahman, A. Polymorphic Transitions in Single Crystals: A New Molecular Dynamics Method. *J. Appl. Phys.* **52**, 7182–7190 (1981).
25. Tsuji, N., Kennemur, J. L., Buyck, T., Lee, S., Prévost, S., Kaib, P. S. J., Bykov, D., Farès, C. & List, B. Activation of Olefins via Asymmetric Brønsted Acid Catalysis. *Science* **359**, 1501–1505 (2018).
