# Supplementary material for: A multi-substrate screening approach for the identification of a broadly applicable Diels–Alder catalyst
Source: Nat Commun. 2019 Feb 15;10:770. doi: 10.1038/s41467-019-08374-z (PMC6377681; doi:10.1038/s41467-019-08374-z)
Supplement: Supplementary file 2 — Description of Additional Supplementary Files [file 41467_2019_8374_MOESM2_ESM.pdf]

Description of Additional Supplementary Information

File Name: Supplementary Data 1

Description: Models from the production MD trajectory taken every 500 ps.
