# Supplementary material for: A multi-substrate screening approach for the identification of a broadly applicable Diels–Alder catalyst
Source: Nat Commun. 2019 Feb 15;10:770. doi: 10.1038/s41467-019-08374-z (PMC6377681; doi:10.1038/s41467-019-08374-z)
Supplement: Supplementary file 3 — Supplementary Data 1 [file 41467_2019_8374_MOESM3_ESM.docx]

**Supplementary Dataset.** Models from the production MD trajectory taken every 500 ps

REMARK GENERATED BY TRJCONV

TITLE KIH t= 0.00000

REMARK THIS IS A SIMULATION BOX

CRYST1 37.604 37.604 37.604 90.00 90.00 90.00 P 1 1

MODEL 1

ATOM 1 C1 KIH 1 16.220 19.820 17.210 1.00 0.00

ATOM 2 C2 KIH 1 16.350 20.110 18.540 1.00 0.00

ATOM 3 C3 KIH 1 15.300 20.050 19.440 1.00 0.00

ATOM 4 C4 KIH 1 14.070 19.720 18.930 1.00 0.00

ATOM 5 C5 KIH 1 13.920 19.340 17.620 1.00 0.00

ATOM 6 C6 KIH 1 14.980 19.450 16.760 1.00 0.00

ATOM 7 C7 KIH 1 12.670 18.900 17.260 1.00 0.00

ATOM 8 C8 KIH 1 12.510 18.470 15.980 1.00 0.00

ATOM 9 C9 KIH 1 13.530 18.600 15.060 1.00 0.00

ATOM 10 C10 KIH 1 14.760 19.060 15.450 1.00 0.00

ATOM 11 C11 KIH 1 15.500 20.300 20.890 1.00 0.00

ATOM 12 C12 KIH 1 15.210 19.410 21.890 1.00 0.00

ATOM 13 C13 KIH 1 15.520 19.700 23.200 1.00 0.00

ATOM 14 C14 KIH 1 15.480 18.710 24.160 1.00 0.00

ATOM 15 C15 KIH 1 15.020 17.440 23.880 1.00 0.00

ATOM 16 C16 KIH 1 14.750 16.540 24.890 1.00 0.00

ATOM 17 C17 KIH 1 15.110 16.870 26.180 1.00 0.00

ATOM 18 C18 KIH 1 15.640 18.110 26.460 1.00 0.00

ATOM 19 C19 KIH 1 15.800 19.040 25.460 1.00 0.00

ATOM 20 C20 KIH 1 16.160 20.320 25.820 1.00 0.00

ATOM 21 C21 KIH 1 16.370 21.250 24.840 1.00 0.00

ATOM 22 C22 KIH 1 16.030 20.930 23.560 1.00 0.00

ATOM 23 C23 KIH 1 16.330 21.840 22.570 1.00 0.00

ATOM 24 C24 KIH 1 16.070 21.500 21.260 1.00 0.00

ATOM 25 H1 KIH 1 13.220 19.840 19.600 1.00 0.00

ATOM 26 H2 KIH 1 11.830 18.890 17.960 1.00 0.00

ATOM 27 H3 KIH 1 11.510 18.290 15.630 1.00 0.00

ATOM 28 H4 KIH 1 13.310 18.180 14.090 1.00 0.00

ATOM 29 H5 KIH 1 15.590 18.980 14.760 1.00 0.00

ATOM 30 H6 KIH 1 14.820 18.490 21.470 1.00 0.00

ATOM 31 H7 KIH 1 14.870 17.150 22.850 1.00 0.00

ATOM 32 H8 KIH 1 14.350 15.560 24.680 1.00 0.00

ATOM 33 H9 KIH 1 14.920 16.190 27.000 1.00 0.00

ATOM 34 H10 KIH 1 15.870 18.390 27.470 1.00 0.00

ATOM 35 H11 KIH 1 16.300 20.640 26.840 1.00 0.00

ATOM 36 H12 KIH 1 16.710 22.260 25.030 1.00 0.00

ATOM 37 H13 KIH 1 16.790 22.780 22.850 1.00 0.00

ATOM 38 H14 KIH 1 16.420 22.170 20.480 1.00 0.00

ATOM 39 O1 KIH 1 17.630 20.310 19.040 1.00 0.00

ATOM 40 O2 KIH 1 19.090 18.520 22.800 1.00 0.00

ATOM 41 C25 KIH 1 17.360 20.130 16.300 1.00 0.00

ATOM 42 C26 KIH 1 18.500 19.400 16.490 1.00 0.00

ATOM 43 C27 KIH 1 19.540 19.660 15.630 1.00 0.00

ATOM 44 C28 KIH 1 19.450 20.530 14.560 1.00 0.00

ATOM 45 C29 KIH 1 18.330 21.340 14.490 1.00 0.00

ATOM 46 C30 KIH 1 17.310 21.170 15.400 1.00 0.00

ATOM 47 C31 KIH 1 18.220 22.240 13.470 1.00 0.00

ATOM 48 C32 KIH 1 17.100 23.030 13.350 1.00 0.00

ATOM 49 C33 KIH 1 16.190 23.060 14.380 1.00 0.00

ATOM 50 C34 KIH 1 16.280 22.080 15.350 1.00 0.00

ATOM 51 C35 KIH 1 20.850 18.920 15.630 1.00 0.00

ATOM 52 C36 KIH 1 20.980 17.620 15.200 1.00 0.00

ATOM 53 C37 KIH 1 22.160 16.910 15.210 1.00 0.00

ATOM 54 C38 KIH 1 22.320 15.590 14.860 1.00 0.00

ATOM 55 C39 KIH 1 21.250 14.830 14.420 1.00 0.00

ATOM 56 C40 KIH 1 21.390 13.480 14.220 1.00 0.00

ATOM 57 C41 KIH 1 22.630 12.890 14.280 1.00 0.00

ATOM 58 C42 KIH 1 23.700 13.640 14.700 1.00 0.00

ATOM 59 C43 KIH 1 23.540 14.980 14.970 1.00 0.00

ATOM 60 C44 KIH 1 24.610 15.650 15.520 1.00 0.00

ATOM 61 C45 KIH 1 24.480 16.990 15.780 1.00 0.00

ATOM 62 C46 KIH 1 23.250 17.590 15.690 1.00 0.00

ATOM 63 C47 KIH 1 23.180 18.900 16.080 1.00 0.00

ATOM 64 C48 KIH 1 21.970 19.560 16.100 1.00 0.00

ATOM 65 H15 KIH 1 20.280 20.670 13.880 1.00 0.00

ATOM 66 H16 KIH 1 18.930 22.280 12.650 1.00 0.00

ATOM 67 H17 KIH 1 17.030 23.790 12.580 1.00 0.00

ATOM 68 H18 KIH 1 15.350 23.740 14.470 1.00 0.00

ATOM 69 H19 KIH 1 15.470 21.960 16.040 1.00 0.00

ATOM 70 H20 KIH 1 20.050 17.130 14.960 1.00 0.00

ATOM 71 H21 KIH 1 20.240 15.200 14.460 1.00 0.00

ATOM 72 H22 KIH 1 20.640 12.840 13.760 1.00 0.00

ATOM 73 H23 KIH 1 22.680 11.870 13.920 1.00 0.00

ATOM 74 H24 KIH 1 24.690 13.200 14.690 1.00 0.00

ATOM 75 H25 KIH 1 25.560 15.130 15.580 1.00 0.00

ATOM 76 H26 KIH 1 25.410 17.490 16.060 1.00 0.00

ATOM 77 H27 KIH 1 24.080 19.470 16.320 1.00 0.00

ATOM 78 H28 KIH 1 21.900 20.540 16.550 1.00 0.00

ATOM 79 O3 KIH 1 18.680 18.560 17.570 1.00 0.00

ATOM 80 O4 KIH 1 17.600 16.600 22.520 1.00 0.00

ATOM 81 C49 KIH 1 19.970 16.510 21.600 1.00 0.00

ATOM 82 F1 KIH 1 20.870 17.170 20.860 1.00 0.00

ATOM 83 F2 KIH 1 20.640 15.970 22.620 1.00 0.00

ATOM 84 F3 KIH 1 19.550 15.410 20.950 1.00 0.00

ATOM 85 N1 KIH 1 18.080 18.080 20.390 1.00 0.00

ATOM 86 P1 KIH 1 18.670 19.120 19.050 1.00 0.00

ATOM 87 S1 KIH 1 18.530 17.520 21.920 1.00 0.00

ATOM 88 C50 KIH 1 23.850 21.900 19.460 1.00 0.00

ATOM 89 C51 KIH 1 23.640 20.600 19.860 1.00 0.00

ATOM 90 C52 KIH 1 24.600 19.870 20.510 1.00 0.00

ATOM 91 C53 KIH 1 25.730 20.550 20.910 1.00 0.00

ATOM 92 C54 KIH 1 25.960 21.850 20.570 1.00 0.00

ATOM 93 C55 KIH 1 24.980 22.570 19.910 1.00 0.00

ATOM 94 C56 KIH 1 27.090 22.520 20.970 1.00 0.00

ATOM 95 C57 KIH 1 27.250 23.880 20.790 1.00 0.00

ATOM 96 C58 KIH 1 26.290 24.580 20.090 1.00 0.00

ATOM 97 C59 KIH 1 25.170 23.910 19.660 1.00 0.00

ATOM 98 C60 KIH 1 24.430 18.410 20.780 1.00 0.00

ATOM 99 C61 KIH 1 24.180 18.010 22.070 1.00 0.00

ATOM 100 C62 KIH 1 23.890 16.720 22.430 1.00 0.00

ATOM 101 C63 KIH 1 23.580 16.330 23.720 1.00 0.00

ATOM 102 C64 KIH 1 23.680 17.150 24.820 1.00 0.00

ATOM 103 C65 KIH 1 23.500 16.650 26.090 1.00 0.00

ATOM 104 C66 KIH 1 23.290 15.290 26.260 1.00 0.00

ATOM 105 C67 KIH 1 23.180 14.460 25.170 1.00 0.00

ATOM 106 C68 KIH 1 23.350 14.980 23.910 1.00 0.00

ATOM 107 C69 KIH 1 23.170 14.130 22.860 1.00 0.00

ATOM 108 C70 KIH 1 23.360 14.550 21.560 1.00 0.00

ATOM 109 C71 KIH 1 23.800 15.840 21.370 1.00 0.00

ATOM 110 C72 KIH 1 24.200 16.200 20.100 1.00 0.00

ATOM 111 C73 KIH 1 24.360 17.520 19.740 1.00 0.00

ATOM 112 H29 KIH 1 26.510 19.980 21.400 1.00 0.00

ATOM 113 H30 KIH 1 27.900 21.990 21.440 1.00 0.00

ATOM 114 H31 KIH 1 28.090 24.330 21.290 1.00 0.00

ATOM 115 H32 KIH 1 26.420 25.640 19.910 1.00 0.00

ATOM 116 H33 KIH 1 24.340 24.430 19.220 1.00 0.00

ATOM 117 H34 KIH 1 24.310 18.800 22.800 1.00 0.00

ATOM 118 H35 KIH 1 23.950 18.190 24.680 1.00 0.00

ATOM 119 H36 KIH 1 23.600 17.330 26.920 1.00 0.00

ATOM 120 H37 KIH 1 23.140 14.850 27.230 1.00 0.00

ATOM 121 H38 KIH 1 22.930 13.430 25.340 1.00 0.00

ATOM 122 H39 KIH 1 22.870 13.090 22.960 1.00 0.00

ATOM 123 H40 KIH 1 23.190 13.910 20.710 1.00 0.00

ATOM 124 H41 KIH 1 24.130 15.530 19.270 1.00 0.00

ATOM 125 H42 KIH 1 24.370 17.860 18.720 1.00 0.00

ATOM 126 O5 KIH 1 22.400 20.030 19.650 1.00 0.00

ATOM 127 O6 KIH 1 21.850 19.070 23.790 1.00 0.00

ATOM 128 C74 KIH 1 22.870 22.570 18.570 1.00 0.00

ATOM 129 C75 KIH 1 21.560 22.720 18.990 1.00 0.00

ATOM 130 C76 KIH 1 20.630 23.390 18.230 1.00 0.00

ATOM 131 C77 KIH 1 21.020 23.790 16.970 1.00 0.00

ATOM 132 C78 KIH 1 22.260 23.450 16.470 1.00 0.00

ATOM 133 C79 KIH 1 23.230 22.920 17.290 1.00 0.00

ATOM 134 C80 KIH 1 22.530 23.810 15.160 1.00 0.00

ATOM 135 C81 KIH 1 23.800 23.650 14.660 1.00 0.00

ATOM 136 C82 KIH 1 24.790 23.050 15.410 1.00 0.00

ATOM 137 C83 KIH 1 24.480 22.790 16.720 1.00 0.00

ATOM 138 C84 KIH 1 19.290 23.860 18.690 1.00 0.00

ATOM 139 C85 KIH 1 19.080 24.320 19.980 1.00 0.00

ATOM 140 C86 KIH 1 17.860 24.780 20.380 1.00 0.00

ATOM 141 C87 KIH 1 17.570 25.210 21.670 1.00 0.00

ATOM 142 C88 KIH 1 18.550 25.420 22.610 1.00 0.00

ATOM 143 C89 KIH 1 18.280 26.100 23.770 1.00 0.00

ATOM 144 C90 KIH 1 16.980 26.390 24.110 1.00 0.00

ATOM 145 C91 KIH 1 15.960 26.130 23.220 1.00 0.00

ATOM 146 C92 KIH 1 16.290 25.580 22.000 1.00 0.00

ATOM 147 C93 KIH 1 15.240 25.420 21.120 1.00 0.00

ATOM 148 C94 KIH 1 15.550 25.000 19.850 1.00 0.00

ATOM 149 C95 KIH 1 16.820 24.600 19.510 1.00 0.00

ATOM 150 C96 KIH 1 16.940 24.110 18.230 1.00 0.00

ATOM 151 C97 KIH 1 18.180 23.610 17.920 1.00 0.00

ATOM 152 H43 KIH 1 20.360 24.380 16.360 1.00 0.00

ATOM 153 H44 KIH 1 21.820 24.380 14.610 1.00 0.00

ATOM 154 H45 KIH 1 24.100 24.000 13.680 1.00 0.00

ATOM 155 H46 KIH 1 25.790 22.930 15.030 1.00 0.00

ATOM 156 H47 KIH 1 25.240 22.370 17.360 1.00 0.00

ATOM 157 H48 KIH 1 19.930 24.290 20.640 1.00 0.00

ATOM 158 H49 KIH 1 19.580 25.170 22.380 1.00 0.00

ATOM 159 H50 KIH 1 19.040 26.210 24.520 1.00 0.00

ATOM 160 H51 KIH 1 16.860 26.850 25.080 1.00 0.00

ATOM 161 H52 KIH 1 15.000 26.380 23.620 1.00 0.00

ATOM 162 H53 KIH 1 14.200 25.550 21.340 1.00 0.00

ATOM 163 H54 KIH 1 14.790 25.120 19.080 1.00 0.00

ATOM 164 H55 KIH 1 16.110 23.990 17.540 1.00 0.00

ATOM 165 H56 KIH 1 18.300 23.260 16.910 1.00 0.00

ATOM 166 O7 KIH 1 21.130 22.160 20.170 1.00 0.00

ATOM 167 O8 KIH 1 22.960 21.140 22.890 1.00 0.00

ATOM 168 C98 KIH 1 20.680 21.380 24.210 1.00 0.00

ATOM 169 F4 KIH 1 21.250 22.000 25.260 1.00 0.00

ATOM 170 F5 KIH 1 20.150 22.360 23.470 1.00 0.00

ATOM 171 F6 KIH 1 19.680 20.660 24.730 1.00 0.00

ATOM 172 N2 KIH 1 21.000 20.120 21.770 1.00 0.00

ATOM 173 P2 KIH 1 21.020 20.590 20.190 1.00 0.00

ATOM 174 S2 KIH 1 21.780 20.390 23.200 1.00 0.00

ATOM 175 N3 KIH 1 19.870 19.890 19.520 1.00 0.00

ATOM 176 H57 KIH 1 20.370 19.330 21.920 1.00 0.00

TER

ENDMDL

REMARK GENERATED BY TRJCONV

TITLE KIH t= 500.00000

REMARK THIS IS A SIMULATION BOX

CRYST1 38.315 38.315 38.315 90.00 90.00 90.00 P 1 1

MODEL 2

ATOM 1 C1 KIH 1 16.130 19.770 17.320 1.00 0.00

ATOM 2 C2 KIH 1 16.360 20.080 18.650 1.00 0.00

ATOM 3 C3 KIH 1 15.330 20.060 19.540 1.00 0.00

ATOM 4 C4 KIH 1 14.040 19.850 19.080 1.00 0.00

ATOM 5 C5 KIH 1 13.830 19.530 17.770 1.00 0.00

ATOM 6 C6 KIH 1 14.860 19.500 16.860 1.00 0.00

ATOM 7 C7 KIH 1 12.540 19.300 17.350 1.00 0.00

ATOM 8 C8 KIH 1 12.280 18.880 16.070 1.00 0.00

ATOM 9 C9 KIH 1 13.310 18.840 15.170 1.00 0.00

ATOM 10 C10 KIH 1 14.590 19.140 15.560 1.00 0.00

ATOM 11 C11 KIH 1 15.630 20.250 20.990 1.00 0.00

ATOM 12 C12 KIH 1 15.270 19.350 21.970 1.00 0.00

ATOM 13 C13 KIH 1 15.440 19.600 23.310 1.00 0.00

ATOM 14 C14 KIH 1 15.080 18.780 24.360 1.00 0.00

ATOM 15 C15 KIH 1 14.730 17.480 24.050 1.00 0.00

ATOM 16 C16 KIH 1 14.330 16.630 25.050 1.00 0.00

ATOM 17 C17 KIH 1 14.320 17.040 26.360 1.00 0.00

ATOM 18 C18 KIH 1 14.780 18.290 26.720 1.00 0.00

ATOM 19 C19 KIH 1 15.200 19.110 25.700 1.00 0.00

ATOM 20 C20 KIH 1 15.800 20.310 25.970 1.00 0.00

ATOM 21 C21 KIH 1 16.190 21.180 24.980 1.00 0.00

ATOM 22 C22 KIH 1 16.040 20.780 23.660 1.00 0.00

ATOM 23 C23 KIH 1 16.410 21.710 22.720 1.00 0.00

ATOM 24 C24 KIH 1 16.130 21.460 21.400 1.00 0.00

ATOM 25 H1 KIH 1 13.230 19.920 19.790 1.00 0.00

ATOM 26 H2 KIH 1 11.700 19.350 18.030 1.00 0.00

ATOM 27 H3 KIH 1 11.260 18.700 15.750 1.00 0.00

ATOM 28 H4 KIH 1 13.130 18.530 14.140 1.00 0.00

ATOM 29 H5 KIH 1 15.340 19.140 14.780 1.00 0.00

ATOM 30 H6 KIH 1 14.620 18.570 21.630 1.00 0.00

ATOM 31 H7 KIH 1 14.720 17.090 23.050 1.00 0.00

ATOM 32 H8 KIH 1 14.070 15.600 24.840 1.00 0.00

ATOM 33 H9 KIH 1 14.000 16.300 27.070 1.00 0.00

ATOM 34 H10 KIH 1 14.880 18.520 27.770 1.00 0.00

ATOM 35 H11 KIH 1 15.990 20.620 26.990 1.00 0.00

ATOM 36 H12 KIH 1 16.640 22.120 25.260 1.00 0.00

ATOM 37 H13 KIH 1 16.980 22.590 22.970 1.00 0.00

ATOM 38 H14 KIH 1 16.350 22.210 20.640 1.00 0.00

ATOM 39 O1 KIH 1 17.660 20.380 19.010 1.00 0.00

ATOM 40 O2 KIH 1 19.190 18.330 22.710 1.00 0.00

ATOM 41 C25 KIH 1 17.220 20.010 16.320 1.00 0.00

ATOM 42 C26 KIH 1 18.380 19.290 16.510 1.00 0.00

ATOM 43 C27 KIH 1 19.400 19.480 15.620 1.00 0.00

ATOM 44 C28 KIH 1 19.380 20.590 14.810 1.00 0.00

ATOM 45 C29 KIH 1 18.270 21.400 14.760 1.00 0.00

ATOM 46 C30 KIH 1 17.150 21.110 15.500 1.00 0.00

ATOM 47 C31 KIH 1 18.350 22.510 13.950 1.00 0.00

ATOM 48 C32 KIH 1 17.260 23.350 13.870 1.00 0.00

ATOM 49 C33 KIH 1 16.130 23.120 14.620 1.00 0.00

ATOM 50 C34 KIH 1 16.100 22.000 15.420 1.00 0.00

ATOM 51 C35 KIH 1 20.640 18.650 15.660 1.00 0.00

ATOM 52 C36 KIH 1 20.710 17.290 15.500 1.00 0.00

ATOM 53 C37 KIH 1 21.870 16.590 15.710 1.00 0.00

ATOM 54 C38 KIH 1 21.990 15.260 15.370 1.00 0.00

ATOM 55 C39 KIH 1 20.920 14.520 14.940 1.00 0.00

ATOM 56 C40 KIH 1 20.990 13.150 14.830 1.00 0.00

ATOM 57 C41 KIH 1 22.150 12.480 15.130 1.00 0.00

ATOM 58 C42 KIH 1 23.230 13.210 15.580 1.00 0.00

ATOM 59 C43 KIH 1 23.150 14.580 15.640 1.00 0.00

ATOM 60 C44 KIH 1 24.240 15.270 16.150 1.00 0.00

ATOM 61 C45 KIH 1 24.160 16.640 16.240 1.00 0.00

ATOM 62 C46 KIH 1 22.970 17.320 16.090 1.00 0.00

ATOM 63 C47 KIH 1 22.900 18.670 16.340 1.00 0.00

ATOM 64 C48 KIH 1 21.700 19.330 16.240 1.00 0.00

ATOM 65 H15 KIH 1 20.290 20.830 14.280 1.00 0.00

ATOM 66 H16 KIH 1 19.270 22.560 13.400 1.00 0.00

ATOM 67 H17 KIH 1 17.240 24.170 13.170 1.00 0.00

ATOM 68 H18 KIH 1 15.360 23.870 14.680 1.00 0.00

ATOM 69 H19 KIH 1 15.220 21.970 16.040 1.00 0.00

ATOM 70 H20 KIH 1 19.770 16.970 15.070 1.00 0.00

ATOM 71 H21 KIH 1 19.950 14.970 14.810 1.00 0.00

ATOM 72 H22 KIH 1 20.150 12.530 14.540 1.00 0.00

ATOM 73 H23 KIH 1 22.180 11.410 15.250 1.00 0.00

ATOM 74 H24 KIH 1 24.150 12.730 15.860 1.00 0.00

ATOM 75 H25 KIH 1 25.190 14.770 16.290 1.00 0.00

ATOM 76 H26 KIH 1 25.020 17.270 16.330 1.00 0.00

ATOM 77 H27 KIH 1 23.820 19.050 16.760 1.00 0.00

ATOM 78 H28 KIH 1 21.690 20.370 16.520 1.00 0.00

ATOM 79 O3 KIH 1 18.690 18.550 17.640 1.00 0.00

ATOM 80 O4 KIH 1 17.420 16.740 22.610 1.00 0.00

ATOM 81 C49 KIH 1 19.660 16.150 21.510 1.00 0.00

ATOM 82 F1 KIH 1 19.150 15.190 20.720 1.00 0.00

ATOM 83 F2 KIH 1 20.660 16.670 20.780 1.00 0.00

ATOM 84 F3 KIH 1 20.210 15.580 22.590 1.00 0.00

ATOM 85 N1 KIH 1 18.020 18.060 20.360 1.00 0.00

ATOM 86 P1 KIH 1 18.660 19.160 19.100 1.00 0.00

ATOM 87 S1 KIH 1 18.460 17.420 21.880 1.00 0.00

ATOM 88 C50 KIH 1 23.660 22.070 19.240 1.00 0.00

ATOM 89 C51 KIH 1 23.610 20.770 19.700 1.00 0.00

ATOM 90 C52 KIH 1 24.700 20.140 20.260 1.00 0.00

ATOM 91 C53 KIH 1 25.870 20.860 20.270 1.00 0.00

ATOM 92 C54 KIH 1 25.880 22.210 20.020 1.00 0.00

ATOM 93 C55 KIH 1 24.800 22.830 19.450 1.00 0.00

ATOM 94 C56 KIH 1 27.070 22.880 20.140 1.00 0.00

ATOM 95 C57 KIH 1 27.210 24.210 19.830 1.00 0.00

ATOM 96 C58 KIH 1 26.080 24.810 19.320 1.00 0.00

ATOM 97 C59 KIH 1 24.900 24.150 19.050 1.00 0.00

ATOM 98 C60 KIH 1 24.670 18.690 20.580 1.00 0.00

ATOM 99 C61 KIH 1 24.640 18.230 21.870 1.00 0.00

ATOM 100 C62 KIH 1 24.580 16.910 22.240 1.00 0.00

ATOM 101 C63 KIH 1 24.570 16.480 23.550 1.00 0.00

ATOM 102 C64 KIH 1 24.500 17.320 24.630 1.00 0.00

ATOM 103 C65 KIH 1 24.170 16.900 25.900 1.00 0.00

ATOM 104 C66 KIH 1 24.100 15.550 26.070 1.00 0.00

ATOM 105 C67 KIH 1 24.220 14.640 25.050 1.00 0.00

ATOM 106 C68 KIH 1 24.420 15.140 23.780 1.00 0.00

ATOM 107 C69 KIH 1 24.330 14.240 22.740 1.00 0.00

ATOM 108 C70 KIH 1 24.410 14.710 21.440 1.00 0.00

ATOM 109 C71 KIH 1 24.520 16.050 21.170 1.00 0.00

ATOM 110 C72 KIH 1 24.430 16.460 19.860 1.00 0.00

ATOM 111 C73 KIH 1 24.530 17.790 19.540 1.00 0.00

ATOM 112 H29 KIH 1 26.820 20.540 20.680 1.00 0.00

ATOM 113 H30 KIH 1 27.920 22.490 20.660 1.00 0.00

ATOM 114 H31 KIH 1 28.110 24.770 20.080 1.00 0.00

ATOM 115 H32 KIH 1 26.190 25.870 19.090 1.00 0.00

ATOM 116 H33 KIH 1 24.120 24.620 18.480 1.00 0.00

ATOM 117 H34 KIH 1 24.700 19.010 22.630 1.00 0.00

ATOM 118 H35 KIH 1 24.510 18.410 24.620 1.00 0.00

ATOM 119 H36 KIH 1 24.090 17.610 26.720 1.00 0.00

ATOM 120 H37 KIH 1 23.990 15.190 27.080 1.00 0.00

ATOM 121 H38 KIH 1 24.070 13.580 25.250 1.00 0.00

ATOM 122 H39 KIH 1 24.360 13.200 23.000 1.00 0.00

ATOM 123 H40 KIH 1 24.360 13.910 20.720 1.00 0.00

ATOM 124 H41 KIH 1 24.230 15.760 19.060 1.00 0.00

ATOM 125 H42 KIH 1 24.610 18.160 18.530 1.00 0.00

ATOM 126 O5 KIH 1 22.440 20.040 19.650 1.00 0.00

ATOM 127 O6 KIH 1 22.140 19.390 24.040 1.00 0.00

ATOM 128 C74 KIH 1 22.560 22.560 18.380 1.00 0.00

ATOM 129 C75 KIH 1 21.330 22.760 18.980 1.00 0.00

ATOM 130 C76 KIH 1 20.370 23.480 18.310 1.00 0.00

ATOM 131 C77 KIH 1 20.490 23.710 16.960 1.00 0.00

ATOM 132 C78 KIH 1 21.720 23.490 16.390 1.00 0.00

ATOM 133 C79 KIH 1 22.760 22.900 17.060 1.00 0.00

ATOM 134 C80 KIH 1 21.900 23.950 15.100 1.00 0.00

ATOM 135 C81 KIH 1 23.160 23.810 14.540 1.00 0.00

ATOM 136 C82 KIH 1 24.200 23.180 15.180 1.00 0.00

ATOM 137 C83 KIH 1 23.960 22.660 16.430 1.00 0.00

ATOM 138 C84 KIH 1 19.110 23.760 19.070 1.00 0.00

ATOM 139 C85 KIH 1 19.080 24.450 20.250 1.00 0.00

ATOM 140 C86 KIH 1 17.930 24.880 20.870 1.00 0.00

ATOM 141 C87 KIH 1 17.870 25.550 22.080 1.00 0.00

ATOM 142 C88 KIH 1 18.960 25.670 22.910 1.00 0.00

ATOM 143 C89 KIH 1 18.880 26.320 24.120 1.00 0.00

ATOM 144 C90 KIH 1 17.680 26.740 24.620 1.00 0.00

ATOM 145 C91 KIH 1 16.560 26.590 23.840 1.00 0.00

ATOM 146 C92 KIH 1 16.680 25.980 22.600 1.00 0.00

ATOM 147 C93 KIH 1 15.500 25.860 21.900 1.00 0.00

ATOM 148 C94 KIH 1 15.600 25.210 20.680 1.00 0.00

ATOM 149 C95 KIH 1 16.760 24.680 20.190 1.00 0.00

ATOM 150 C96 KIH 1 16.740 24.050 18.960 1.00 0.00

ATOM 151 C97 KIH 1 17.920 23.560 18.440 1.00 0.00

ATOM 152 H43 KIH 1 19.720 24.330 16.520 1.00 0.00

ATOM 153 H44 KIH 1 21.090 24.370 14.530 1.00 0.00

ATOM 154 H45 KIH 1 23.340 24.250 13.580 1.00 0.00

ATOM 155 H46 KIH 1 25.090 23.020 14.590 1.00 0.00

ATOM 156 H47 KIH 1 24.780 22.160 16.920 1.00 0.00

ATOM 157 H48 KIH 1 20.030 24.700 20.700 1.00 0.00

ATOM 158 H49 KIH 1 19.920 25.230 22.670 1.00 0.00

ATOM 159 H50 KIH 1 19.740 26.420 24.770 1.00 0.00

ATOM 160 H51 KIH 1 17.790 27.250 25.560 1.00 0.00

ATOM 161 H52 KIH 1 15.630 26.850 24.310 1.00 0.00

ATOM 162 H53 KIH 1 14.620 26.310 22.300 1.00 0.00

ATOM 163 H54 KIH 1 14.630 25.090 20.220 1.00 0.00

ATOM 164 H55 KIH 1 15.790 24.110 18.460 1.00 0.00

ATOM 165 H56 KIH 1 18.070 23.310 17.400 1.00 0.00

ATOM 166 O7 KIH 1 21.020 22.130 20.170 1.00 0.00

ATOM 167 O8 KIH 1 22.880 21.420 22.850 1.00 0.00

ATOM 168 C98 KIH 1 20.660 21.410 24.220 1.00 0.00

ATOM 169 F4 KIH 1 21.170 21.750 25.410 1.00 0.00

ATOM 170 F5 KIH 1 20.370 22.600 23.660 1.00 0.00

ATOM 171 F6 KIH 1 19.500 20.840 24.530 1.00 0.00

ATOM 172 N2 KIH 1 21.070 20.090 21.780 1.00 0.00

ATOM 173 P2 KIH 1 21.060 20.550 20.200 1.00 0.00

ATOM 174 S2 KIH 1 21.820 20.500 23.190 1.00 0.00

ATOM 175 N3 KIH 1 19.870 19.910 19.530 1.00 0.00

ATOM 176 H57 KIH 1 20.430 19.320 21.920 1.00 0.00

TER

ENDMDL

REMARK GENERATED BY TRJCONV

TITLE KIH t= 1000.00000

REMARK THIS IS A SIMULATION BOX

CRYST1 38.658 38.658 38.658 90.00 90.00 90.00 P 1 1

MODEL 3

ATOM 1 C1 KIH 1 16.330 19.810 16.980 1.00 0.00

ATOM 2 C2 KIH 1 16.340 20.040 18.330 1.00 0.00

ATOM 3 C3 KIH 1 15.210 19.900 19.100 1.00 0.00

ATOM 4 C4 KIH 1 14.020 19.650 18.470 1.00 0.00

ATOM 5 C5 KIH 1 13.980 19.550 17.100 1.00 0.00

ATOM 6 C6 KIH 1 15.140 19.560 16.350 1.00 0.00

ATOM 7 C7 KIH 1 12.770 19.200 16.560 1.00 0.00

ATOM 8 C8 KIH 1 12.640 18.950 15.210 1.00 0.00

ATOM 9 C9 KIH 1 13.780 19.000 14.450 1.00 0.00

ATOM 10 C10 KIH 1 15.000 19.250 15.020 1.00 0.00

ATOM 11 C11 KIH 1 15.200 20.280 20.550 1.00 0.00

ATOM 12 C12 KIH 1 15.060 19.380 21.570 1.00 0.00

ATOM 13 C13 KIH 1 15.290 19.670 22.890 1.00 0.00

ATOM 14 C14 KIH 1 15.220 18.860 24.000 1.00 0.00

ATOM 15 C15 KIH 1 14.900 17.530 23.830 1.00 0.00

ATOM 16 C16 KIH 1 14.810 16.630 24.870 1.00 0.00

ATOM 17 C17 KIH 1 14.950 17.100 26.160 1.00 0.00

ATOM 18 C18 KIH 1 15.190 18.440 26.360 1.00 0.00

ATOM 19 C19 KIH 1 15.420 19.290 25.290 1.00 0.00

ATOM 20 C20 KIH 1 15.820 20.580 25.500 1.00 0.00

ATOM 21 C21 KIH 1 15.940 21.430 24.420 1.00 0.00

ATOM 22 C22 KIH 1 15.650 20.980 23.160 1.00 0.00

ATOM 23 C23 KIH 1 15.780 21.910 22.160 1.00 0.00

ATOM 24 C24 KIH 1 15.620 21.560 20.830 1.00 0.00

ATOM 25 H1 KIH 1 13.100 19.760 19.030 1.00 0.00

ATOM 26 H2 KIH 1 11.820 19.110 17.070 1.00 0.00

ATOM 27 H3 KIH 1 11.710 18.660 14.750 1.00 0.00

ATOM 28 H4 KIH 1 13.630 18.670 13.430 1.00 0.00

ATOM 29 H5 KIH 1 15.820 19.270 14.310 1.00 0.00

ATOM 30 H6 KIH 1 14.720 18.370 21.360 1.00 0.00

ATOM 31 H7 KIH 1 14.810 17.060 22.870 1.00 0.00

ATOM 32 H8 KIH 1 14.530 15.620 24.630 1.00 0.00

ATOM 33 H9 KIH 1 14.870 16.470 27.030 1.00 0.00

ATOM 34 H10 KIH 1 15.450 18.770 27.360 1.00 0.00

ATOM 35 H11 KIH 1 16.060 20.960 26.480 1.00 0.00

ATOM 36 H12 KIH 1 16.280 22.450 24.570 1.00 0.00

ATOM 37 H13 KIH 1 15.990 22.960 22.300 1.00 0.00

ATOM 38 H14 KIH 1 15.780 22.300 20.060 1.00 0.00

ATOM 39 O1 KIH 1 17.520 20.330 18.990 1.00 0.00

ATOM 40 O2 KIH 1 19.070 18.570 22.690 1.00 0.00

ATOM 41 C25 KIH 1 17.580 20.140 16.230 1.00 0.00

ATOM 42 C26 KIH 1 18.710 19.390 16.500 1.00 0.00

ATOM 43 C27 KIH 1 19.830 19.630 15.750 1.00 0.00

ATOM 44 C28 KIH 1 19.910 20.580 14.770 1.00 0.00

ATOM 45 C29 KIH 1 18.850 21.440 14.630 1.00 0.00

ATOM 46 C30 KIH 1 17.730 21.240 15.400 1.00 0.00

ATOM 47 C31 KIH 1 18.930 22.510 13.760 1.00 0.00

ATOM 48 C32 KIH 1 17.950 23.480 13.730 1.00 0.00

ATOM 49 C33 KIH 1 16.760 23.130 14.330 1.00 0.00

ATOM 50 C34 KIH 1 16.660 22.070 15.200 1.00 0.00

ATOM 51 C35 KIH 1 21.070 18.820 15.970 1.00 0.00

ATOM 52 C36 KIH 1 21.120 17.550 15.420 1.00 0.00

ATOM 53 C37 KIH 1 22.290 16.850 15.330 1.00 0.00

ATOM 54 C38 KIH 1 22.440 15.620 14.740 1.00 0.00

ATOM 55 C39 KIH 1 21.300 14.960 14.340 1.00 0.00

ATOM 56 C40 KIH 1 21.390 13.800 13.600 1.00 0.00

ATOM 57 C41 KIH 1 22.620 13.250 13.360 1.00 0.00

ATOM 58 C42 KIH 1 23.780 13.890 13.730 1.00 0.00

ATOM 59 C43 KIH 1 23.660 15.050 14.440 1.00 0.00

ATOM 60 C44 KIH 1 24.780 15.600 15.050 1.00 0.00

ATOM 61 C45 KIH 1 24.640 16.790 15.730 1.00 0.00

ATOM 62 C46 KIH 1 23.430 17.440 15.830 1.00 0.00

ATOM 63 C47 KIH 1 23.360 18.670 16.460 1.00 0.00

ATOM 64 C48 KIH 1 22.160 19.330 16.620 1.00 0.00

ATOM 65 H15 KIH 1 20.830 20.760 14.210 1.00 0.00

ATOM 66 H16 KIH 1 19.810 22.720 13.170 1.00 0.00

ATOM 67 H17 KIH 1 17.960 24.290 13.020 1.00 0.00

ATOM 68 H18 KIH 1 15.900 23.780 14.200 1.00 0.00

ATOM 69 H19 KIH 1 15.780 21.960 15.810 1.00 0.00

ATOM 70 H20 KIH 1 20.210 17.190 14.960 1.00 0.00

ATOM 71 H21 KIH 1 20.340 15.140 14.790 1.00 0.00

ATOM 72 H22 KIH 1 20.520 13.220 13.340 1.00 0.00

ATOM 73 H23 KIH 1 22.720 12.290 12.870 1.00 0.00

ATOM 74 H24 KIH 1 24.780 13.470 13.620 1.00 0.00

ATOM 75 H25 KIH 1 25.750 15.130 15.050 1.00 0.00

ATOM 76 H26 KIH 1 25.480 17.160 16.290 1.00 0.00

ATOM 77 H27 KIH 1 24.240 19.060 16.920 1.00 0.00

ATOM 78 H28 KIH 1 22.230 20.360 16.940 1.00 0.00

ATOM 79 O3 KIH 1 18.740 18.570 17.620 1.00 0.00

ATOM 80 O4 KIH 1 17.630 16.590 22.530 1.00 0.00

ATOM 81 C49 KIH 1 19.960 16.510 21.400 1.00 0.00

ATOM 82 F1 KIH 1 19.610 15.390 20.740 1.00 0.00

ATOM 83 F2 KIH 1 20.830 17.190 20.650 1.00 0.00

ATOM 84 F3 KIH 1 20.630 16.100 22.490 1.00 0.00

ATOM 85 N1 KIH 1 18.070 18.080 20.370 1.00 0.00

ATOM 86 P1 KIH 1 18.610 19.190 19.070 1.00 0.00

ATOM 87 S1 KIH 1 18.550 17.490 21.890 1.00 0.00

ATOM 88 C50 KIH 1 24.030 21.590 19.830 1.00 0.00

ATOM 89 C51 KIH 1 23.600 20.290 20.010 1.00 0.00

ATOM 90 C52 KIH 1 24.430 19.370 20.600 1.00 0.00

ATOM 91 C53 KIH 1 25.700 19.760 20.970 1.00 0.00

ATOM 92 C54 KIH 1 26.100 21.070 20.830 1.00 0.00

ATOM 93 C55 KIH 1 25.260 22.010 20.260 1.00 0.00

ATOM 94 C56 KIH 1 27.390 21.370 21.180 1.00 0.00

ATOM 95 C57 KIH 1 27.850 22.640 20.950 1.00 0.00

ATOM 96 C58 KIH 1 27.040 23.610 20.370 1.00 0.00

ATOM 97 C59 KIH 1 25.750 23.270 20.020 1.00 0.00

ATOM 98 C60 KIH 1 24.150 17.950 20.930 1.00 0.00

ATOM 99 C61 KIH 1 23.820 17.490 22.190 1.00 0.00

ATOM 100 C62 KIH 1 23.540 16.180 22.510 1.00 0.00

ATOM 101 C63 KIH 1 23.300 15.720 23.780 1.00 0.00

ATOM 102 C64 KIH 1 23.350 16.500 24.910 1.00 0.00

ATOM 103 C65 KIH 1 23.090 16.040 26.180 1.00 0.00

ATOM 104 C66 KIH 1 22.910 14.690 26.400 1.00 0.00

ATOM 105 C67 KIH 1 23.050 13.860 25.320 1.00 0.00

ATOM 106 C68 KIH 1 23.130 14.380 24.060 1.00 0.00

ATOM 107 C69 KIH 1 23.200 13.490 23.010 1.00 0.00

ATOM 108 C70 KIH 1 23.430 13.910 21.720 1.00 0.00

ATOM 109 C71 KIH 1 23.670 15.250 21.510 1.00 0.00

ATOM 110 C72 KIH 1 23.820 15.670 20.210 1.00 0.00

ATOM 111 C73 KIH 1 24.140 16.980 19.950 1.00 0.00

ATOM 112 H29 KIH 1 26.370 19.070 21.470 1.00 0.00

ATOM 113 H30 KIH 1 27.990 20.600 21.670 1.00 0.00

ATOM 114 H31 KIH 1 28.830 22.830 21.320 1.00 0.00

ATOM 115 H32 KIH 1 27.440 24.600 20.190 1.00 0.00

ATOM 116 H33 KIH 1 25.030 23.960 19.620 1.00 0.00

ATOM 117 H34 KIH 1 23.890 18.260 22.960 1.00 0.00

ATOM 118 H35 KIH 1 23.540 17.560 24.830 1.00 0.00

ATOM 119 H36 KIH 1 23.140 16.650 27.070 1.00 0.00

ATOM 120 H37 KIH 1 22.690 14.330 27.390 1.00 0.00

ATOM 121 H38 KIH 1 22.890 12.810 25.530 1.00 0.00

ATOM 122 H39 KIH 1 23.090 12.430 23.190 1.00 0.00

ATOM 123 H40 KIH 1 23.480 13.150 20.950 1.00 0.00

ATOM 124 H41 KIH 1 24.050 14.970 19.420 1.00 0.00

ATOM 125 H42 KIH 1 24.500 17.280 18.980 1.00 0.00

ATOM 126 O5 KIH 1 22.350 19.850 19.610 1.00 0.00

ATOM 127 O6 KIH 1 21.880 19.400 24.110 1.00 0.00

ATOM 128 C74 KIH 1 23.200 22.340 18.830 1.00 0.00

ATOM 129 C75 KIH 1 21.900 22.660 19.100 1.00 0.00

ATOM 130 C76 KIH 1 21.150 23.450 18.250 1.00 0.00

ATOM 131 C77 KIH 1 21.720 23.850 17.070 1.00 0.00

ATOM 132 C78 KIH 1 22.980 23.400 16.720 1.00 0.00

ATOM 133 C79 KIH 1 23.730 22.620 17.590 1.00 0.00

ATOM 134 C80 KIH 1 23.420 23.730 15.460 1.00 0.00

ATOM 135 C81 KIH 1 24.590 23.200 14.960 1.00 0.00

ATOM 136 C82 KIH 1 25.340 22.380 15.770 1.00 0.00

ATOM 137 C83 KIH 1 24.910 22.160 17.060 1.00 0.00

ATOM 138 C84 KIH 1 19.760 23.830 18.620 1.00 0.00

ATOM 139 C85 KIH 1 19.490 24.510 19.770 1.00 0.00

ATOM 140 C86 KIH 1 18.240 24.920 20.200 1.00 0.00

ATOM 141 C87 KIH 1 18.020 25.510 21.420 1.00 0.00

ATOM 142 C88 KIH 1 19.020 25.670 22.340 1.00 0.00

ATOM 143 C89 KIH 1 18.700 26.160 23.570 1.00 0.00

ATOM 144 C90 KIH 1 17.440 26.590 23.910 1.00 0.00

ATOM 145 C91 KIH 1 16.480 26.540 22.920 1.00 0.00

ATOM 146 C92 KIH 1 16.740 25.920 21.720 1.00 0.00

ATOM 147 C93 KIH 1 15.730 25.800 20.800 1.00 0.00

ATOM 148 C94 KIH 1 15.930 25.110 19.620 1.00 0.00

ATOM 149 C95 KIH 1 17.200 24.660 19.330 1.00 0.00

ATOM 150 C96 KIH 1 17.400 23.980 18.150 1.00 0.00

ATOM 151 C97 KIH 1 18.670 23.600 17.810 1.00 0.00

ATOM 152 H43 KIH 1 21.110 24.450 16.420 1.00 0.00

ATOM 153 H44 KIH 1 22.790 24.330 14.820 1.00 0.00

ATOM 154 H45 KIH 1 24.830 23.340 13.920 1.00 0.00

ATOM 155 H46 KIH 1 26.300 21.970 15.530 1.00 0.00

ATOM 156 H47 KIH 1 25.640 21.530 17.560 1.00 0.00

ATOM 157 H48 KIH 1 20.390 24.700 20.340 1.00 0.00

ATOM 158 H49 KIH 1 20.070 25.430 22.210 1.00 0.00

ATOM 159 H50 KIH 1 19.520 26.270 24.270 1.00 0.00

ATOM 160 H51 KIH 1 17.190 26.920 24.910 1.00 0.00

ATOM 161 H52 KIH 1 15.490 26.840 23.220 1.00 0.00

ATOM 162 H53 KIH 1 14.750 26.220 20.970 1.00 0.00

ATOM 163 H54 KIH 1 15.110 24.830 18.990 1.00 0.00

ATOM 164 H55 KIH 1 16.590 23.820 17.450 1.00 0.00

ATOM 165 H56 KIH 1 18.830 23.070 16.890 1.00 0.00

ATOM 166 O7 KIH 1 21.210 22.050 20.140 1.00 0.00

ATOM 167 O8 KIH 1 22.870 21.380 22.960 1.00 0.00

ATOM 168 C98 KIH 1 20.450 21.550 23.830 1.00 0.00

ATOM 169 F4 KIH 1 20.600 22.140 25.030 1.00 0.00

ATOM 170 F5 KIH 1 20.280 22.570 22.970 1.00 0.00

ATOM 171 F6 KIH 1 19.300 20.870 23.940 1.00 0.00

ATOM 172 N2 KIH 1 21.090 19.970 21.790 1.00 0.00

ATOM 173 P2 KIH 1 21.040 20.480 20.220 1.00 0.00

ATOM 174 S2 KIH 1 21.760 20.500 23.210 1.00 0.00

ATOM 175 N3 KIH 1 19.840 19.940 19.490 1.00 0.00

ATOM 176 H57 KIH 1 20.520 19.140 21.870 1.00 0.00

TER

ENDMDL

REMARK GENERATED BY TRJCONV

TITLE KIH t= 1500.00000

REMARK THIS IS A SIMULATION BOX

CRYST1 38.368 38.368 38.368 90.00 90.00 90.00 P 1 1

MODEL 4

ATOM 1 C1 KIH 1 16.250 19.620 17.110 1.00 0.00

ATOM 2 C2 KIH 1 16.310 19.790 18.470 1.00 0.00

ATOM 3 C3 KIH 1 15.230 19.540 19.290 1.00 0.00

ATOM 4 C4 KIH 1 14.040 19.150 18.730 1.00 0.00

ATOM 5 C5 KIH 1 13.930 19.010 17.370 1.00 0.00

ATOM 6 C6 KIH 1 15.030 19.250 16.580 1.00 0.00

ATOM 7 C7 KIH 1 12.680 18.770 16.860 1.00 0.00

ATOM 8 C8 KIH 1 12.490 18.630 15.510 1.00 0.00

ATOM 9 C9 KIH 1 13.610 18.670 14.700 1.00 0.00

ATOM 10 C10 KIH 1 14.830 18.990 15.240 1.00 0.00

ATOM 11 C11 KIH 1 15.340 19.690 20.780 1.00 0.00

ATOM 12 C12 KIH 1 15.120 18.630 21.620 1.00 0.00

ATOM 13 C13 KIH 1 15.200 18.740 22.990 1.00 0.00

ATOM 14 C14 KIH 1 14.970 17.670 23.820 1.00 0.00

ATOM 15 C15 KIH 1 14.500 16.430 23.460 1.00 0.00

ATOM 16 C16 KIH 1 14.170 15.480 24.400 1.00 0.00

ATOM 17 C17 KIH 1 14.330 15.750 25.740 1.00 0.00

ATOM 18 C18 KIH 1 14.830 16.980 26.080 1.00 0.00

ATOM 19 C19 KIH 1 15.180 17.940 25.160 1.00 0.00

ATOM 20 C20 KIH 1 15.820 19.050 25.650 1.00 0.00

ATOM 21 C21 KIH 1 16.050 20.080 24.780 1.00 0.00

ATOM 22 C22 KIH 1 15.690 19.950 23.450 1.00 0.00

ATOM 23 C23 KIH 1 15.910 21.030 22.650 1.00 0.00

ATOM 24 C24 KIH 1 15.650 20.920 21.310 1.00 0.00

ATOM 25 H1 KIH 1 13.280 18.870 19.440 1.00 0.00

ATOM 26 H2 KIH 1 11.880 18.420 17.510 1.00 0.00

ATOM 27 H3 KIH 1 11.500 18.340 15.180 1.00 0.00

ATOM 28 H4 KIH 1 13.600 18.280 13.690 1.00 0.00

ATOM 29 H5 KIH 1 15.620 18.950 14.510 1.00 0.00

ATOM 30 H6 KIH 1 14.780 17.710 21.170 1.00 0.00

ATOM 31 H7 KIH 1 14.530 16.190 22.410 1.00 0.00

ATOM 32 H8 KIH 1 13.910 14.520 23.970 1.00 0.00

ATOM 33 H9 KIH 1 14.330 14.980 26.500 1.00 0.00

ATOM 34 H10 KIH 1 15.200 17.090 27.100 1.00 0.00

ATOM 35 H11 KIH 1 15.950 19.310 26.690 1.00 0.00

ATOM 36 H12 KIH 1 16.390 21.020 25.180 1.00 0.00

ATOM 37 H13 KIH 1 16.220 21.990 23.040 1.00 0.00

ATOM 38 H14 KIH 1 15.810 21.820 20.740 1.00 0.00

ATOM 39 O1 KIH 1 17.520 20.220 18.970 1.00 0.00

ATOM 40 O2 KIH 1 19.070 18.620 22.750 1.00 0.00

ATOM 41 C25 KIH 1 17.450 19.990 16.290 1.00 0.00

ATOM 42 C26 KIH 1 18.610 19.300 16.500 1.00 0.00

ATOM 43 C27 KIH 1 19.750 19.550 15.760 1.00 0.00

ATOM 44 C28 KIH 1 19.720 20.630 14.900 1.00 0.00

ATOM 45 C29 KIH 1 18.550 21.300 14.600 1.00 0.00

ATOM 46 C30 KIH 1 17.450 21.030 15.380 1.00 0.00

ATOM 47 C31 KIH 1 18.590 22.370 13.740 1.00 0.00

ATOM 48 C32 KIH 1 17.410 23.040 13.510 1.00 0.00

ATOM 49 C33 KIH 1 16.300 22.810 14.290 1.00 0.00

ATOM 50 C34 KIH 1 16.350 21.840 15.260 1.00 0.00

ATOM 51 C35 KIH 1 20.990 18.740 15.860 1.00 0.00

ATOM 52 C36 KIH 1 20.980 17.460 15.340 1.00 0.00

ATOM 53 C37 KIH 1 22.080 16.650 15.250 1.00 0.00

ATOM 54 C38 KIH 1 22.080 15.440 14.570 1.00 0.00

ATOM 55 C39 KIH 1 20.940 14.960 13.980 1.00 0.00

ATOM 56 C40 KIH 1 20.910 13.730 13.370 1.00 0.00

ATOM 57 C41 KIH 1 21.980 12.870 13.510 1.00 0.00

ATOM 58 C42 KIH 1 23.150 13.390 14.010 1.00 0.00

ATOM 59 C43 KIH 1 23.220 14.670 14.510 1.00 0.00

ATOM 60 C44 KIH 1 24.340 15.210 15.090 1.00 0.00

ATOM 61 C45 KIH 1 24.380 16.370 15.820 1.00 0.00

ATOM 62 C46 KIH 1 23.230 17.120 15.830 1.00 0.00

ATOM 63 C47 KIH 1 23.290 18.360 16.420 1.00 0.00

ATOM 64 C48 KIH 1 22.170 19.150 16.440 1.00 0.00

ATOM 65 H15 KIH 1 20.660 20.890 14.460 1.00 0.00

ATOM 66 H16 KIH 1 19.540 22.520 13.260 1.00 0.00

ATOM 67 H17 KIH 1 17.400 23.870 12.820 1.00 0.00

ATOM 68 H18 KIH 1 15.440 23.470 14.300 1.00 0.00

ATOM 69 H19 KIH 1 15.490 21.610 15.860 1.00 0.00

ATOM 70 H20 KIH 1 20.030 17.240 14.870 1.00 0.00

ATOM 71 H21 KIH 1 19.980 15.470 14.010 1.00 0.00

ATOM 72 H22 KIH 1 19.970 13.400 12.990 1.00 0.00

ATOM 73 H23 KIH 1 21.920 11.870 13.090 1.00 0.00

ATOM 74 H24 KIH 1 24.020 12.770 13.920 1.00 0.00

ATOM 75 H25 KIH 1 25.230 14.610 14.920 1.00 0.00

ATOM 76 H26 KIH 1 25.280 16.720 16.320 1.00 0.00

ATOM 77 H27 KIH 1 24.230 18.740 16.800 1.00 0.00

ATOM 78 H28 KIH 1 22.070 20.130 16.900 1.00 0.00

ATOM 79 O3 KIH 1 18.750 18.510 17.620 1.00 0.00

ATOM 80 O4 KIH 1 17.730 16.550 22.530 1.00 0.00

ATOM 81 C49 KIH 1 20.060 16.580 21.660 1.00 0.00

ATOM 82 F1 KIH 1 19.840 15.410 21.060 1.00 0.00

ATOM 83 F2 KIH 1 20.880 17.250 20.850 1.00 0.00

ATOM 84 F3 KIH 1 20.730 16.280 22.790 1.00 0.00

ATOM 85 N1 KIH 1 18.080 18.090 20.410 1.00 0.00

ATOM 86 P1 KIH 1 18.660 19.140 19.070 1.00 0.00

ATOM 87 S1 KIH 1 18.580 17.540 21.930 1.00 0.00

ATOM 88 C50 KIH 1 23.720 21.880 19.240 1.00 0.00

ATOM 89 C51 KIH 1 23.500 20.590 19.660 1.00 0.00

ATOM 90 C52 KIH 1 24.490 19.880 20.310 1.00 0.00

ATOM 91 C53 KIH 1 25.670 20.530 20.610 1.00 0.00

ATOM 92 C54 KIH 1 25.840 21.840 20.200 1.00 0.00

ATOM 93 C55 KIH 1 24.910 22.540 19.460 1.00 0.00

ATOM 94 C56 KIH 1 27.060 22.380 20.520 1.00 0.00

ATOM 95 C57 KIH 1 27.320 23.690 20.200 1.00 0.00

ATOM 96 C58 KIH 1 26.410 24.380 19.430 1.00 0.00

ATOM 97 C59 KIH 1 25.230 23.810 19.040 1.00 0.00

ATOM 98 C60 KIH 1 24.280 18.470 20.720 1.00 0.00

ATOM 99 C61 KIH 1 24.360 18.010 22.010 1.00 0.00

ATOM 100 C62 KIH 1 24.140 16.710 22.380 1.00 0.00

ATOM 101 C63 KIH 1 24.180 16.280 23.690 1.00 0.00

ATOM 102 C64 KIH 1 24.030 17.180 24.720 1.00 0.00

ATOM 103 C65 KIH 1 24.110 16.710 26.010 1.00 0.00

ATOM 104 C66 KIH 1 24.140 15.370 26.290 1.00 0.00

ATOM 105 C67 KIH 1 24.130 14.490 25.220 1.00 0.00

ATOM 106 C68 KIH 1 24.210 14.920 23.920 1.00 0.00

ATOM 107 C69 KIH 1 24.070 14.010 22.890 1.00 0.00

ATOM 108 C70 KIH 1 23.910 14.480 21.610 1.00 0.00

ATOM 109 C71 KIH 1 24.020 15.820 21.340 1.00 0.00

ATOM 110 C72 KIH 1 24.070 16.180 20.010 1.00 0.00

ATOM 111 C73 KIH 1 24.270 17.520 19.730 1.00 0.00

ATOM 112 H29 KIH 1 26.410 20.050 21.240 1.00 0.00

ATOM 113 H30 KIH 1 27.810 21.860 21.100 1.00 0.00

ATOM 114 H31 KIH 1 28.250 24.130 20.530 1.00 0.00

ATOM 115 H32 KIH 1 26.680 25.390 19.170 1.00 0.00

ATOM 116 H33 KIH 1 24.530 24.350 18.410 1.00 0.00

ATOM 117 H34 KIH 1 24.490 18.800 22.750 1.00 0.00

ATOM 118 H35 KIH 1 23.850 18.210 24.480 1.00 0.00

ATOM 119 H36 KIH 1 24.090 17.440 26.820 1.00 0.00

ATOM 120 H37 KIH 1 24.170 15.050 27.310 1.00 0.00

ATOM 121 H38 KIH 1 24.090 13.450 25.520 1.00 0.00

ATOM 122 H39 KIH 1 23.990 12.970 23.130 1.00 0.00

ATOM 123 H40 KIH 1 23.890 13.710 20.840 1.00 0.00

ATOM 124 H41 KIH 1 24.000 15.470 19.200 1.00 0.00

ATOM 125 H42 KIH 1 24.460 17.870 18.730 1.00 0.00

ATOM 126 O5 KIH 1 22.300 19.990 19.410 1.00 0.00

ATOM 127 O6 KIH 1 21.840 19.120 23.870 1.00 0.00

ATOM 128 C74 KIH 1 22.710 22.470 18.300 1.00 0.00

ATOM 129 C75 KIH 1 21.480 22.720 18.880 1.00 0.00

ATOM 130 C76 KIH 1 20.510 23.410 18.200 1.00 0.00

ATOM 131 C77 KIH 1 20.740 23.700 16.880 1.00 0.00

ATOM 132 C78 KIH 1 21.960 23.420 16.290 1.00 0.00

ATOM 133 C79 KIH 1 22.920 22.710 16.960 1.00 0.00

ATOM 134 C80 KIH 1 21.990 23.640 14.930 1.00 0.00

ATOM 135 C81 KIH 1 23.070 23.180 14.210 1.00 0.00

ATOM 136 C82 KIH 1 24.080 22.530 14.870 1.00 0.00

ATOM 137 C83 KIH 1 23.980 22.250 16.220 1.00 0.00

ATOM 138 C84 KIH 1 19.160 23.750 18.710 1.00 0.00

ATOM 139 C85 KIH 1 18.970 24.400 19.910 1.00 0.00

ATOM 140 C86 KIH 1 17.730 24.720 20.430 1.00 0.00

ATOM 141 C87 KIH 1 17.450 25.110 21.720 1.00 0.00

ATOM 142 C88 KIH 1 18.410 25.400 22.660 1.00 0.00

ATOM 143 C89 KIH 1 18.090 25.820 23.930 1.00 0.00

ATOM 144 C90 KIH 1 16.770 25.950 24.310 1.00 0.00

ATOM 145 C91 KIH 1 15.800 25.720 23.380 1.00 0.00

ATOM 146 C92 KIH 1 16.150 25.300 22.120 1.00 0.00

ATOM 147 C93 KIH 1 15.080 25.050 21.280 1.00 0.00

ATOM 148 C94 KIH 1 15.350 24.660 19.980 1.00 0.00

ATOM 149 C95 KIH 1 16.660 24.510 19.600 1.00 0.00

ATOM 150 C96 KIH 1 16.820 23.930 18.360 1.00 0.00

ATOM 151 C97 KIH 1 18.070 23.600 17.890 1.00 0.00

ATOM 152 H43 KIH 1 19.990 24.180 16.270 1.00 0.00

ATOM 153 H44 KIH 1 21.220 24.170 14.380 1.00 0.00

ATOM 154 H45 KIH 1 23.120 23.330 13.140 1.00 0.00

ATOM 155 H46 KIH 1 24.910 22.120 14.310 1.00 0.00

ATOM 156 H47 KIH 1 24.760 21.620 16.610 1.00 0.00

ATOM 157 H48 KIH 1 19.870 24.560 20.470 1.00 0.00

ATOM 158 H49 KIH 1 19.440 25.120 22.510 1.00 0.00

ATOM 159 H50 KIH 1 18.820 25.990 24.710 1.00 0.00

ATOM 160 H51 KIH 1 16.590 26.280 25.320 1.00 0.00

ATOM 161 H52 KIH 1 14.760 25.930 23.620 1.00 0.00

ATOM 162 H53 KIH 1 14.090 25.210 21.650 1.00 0.00

ATOM 163 H54 KIH 1 14.480 24.530 19.350 1.00 0.00

ATOM 164 H55 KIH 1 15.970 23.800 17.700 1.00 0.00

ATOM 165 H56 KIH 1 18.150 23.230 16.880 1.00 0.00

ATOM 166 O7 KIH 1 21.150 22.140 20.090 1.00 0.00

ATOM 167 O8 KIH 1 22.900 21.160 22.950 1.00 0.00

ATOM 168 C98 KIH 1 20.570 21.470 24.040 1.00 0.00

ATOM 169 F4 KIH 1 20.790 21.590 25.360 1.00 0.00

ATOM 170 F5 KIH 1 20.620 22.720 23.530 1.00 0.00

ATOM 171 F6 KIH 1 19.270 21.150 24.000 1.00 0.00

ATOM 172 N2 KIH 1 21.010 20.060 21.750 1.00 0.00

ATOM 173 P2 KIH 1 21.040 20.560 20.170 1.00 0.00

ATOM 174 S2 KIH 1 21.720 20.380 23.200 1.00 0.00

ATOM 175 N3 KIH 1 19.850 19.950 19.490 1.00 0.00

ATOM 176 H57 KIH 1 20.400 19.240 21.800 1.00 0.00

TER

ENDMDL

REMARK GENERATED BY TRJCONV

TITLE KIH t= 2000.00000

REMARK THIS IS A SIMULATION BOX

CRYST1 38.650 38.650 38.650 90.00 90.00 90.00 P 1 1

MODEL 5

ATOM 1 C1 KIH 1 16.310 20.040 16.970 1.00 0.00

ATOM 2 C2 KIH 1 16.370 20.200 18.340 1.00 0.00

ATOM 3 C3 KIH 1 15.300 20.070 19.190 1.00 0.00

ATOM 4 C4 KIH 1 14.160 19.640 18.550 1.00 0.00

ATOM 5 C5 KIH 1 14.040 19.430 17.190 1.00 0.00

ATOM 6 C6 KIH 1 15.140 19.620 16.380 1.00 0.00

ATOM 7 C7 KIH 1 12.820 19.090 16.670 1.00 0.00

ATOM 8 C8 KIH 1 12.760 18.890 15.320 1.00 0.00

ATOM 9 C9 KIH 1 13.810 19.100 14.460 1.00 0.00

ATOM 10 C10 KIH 1 15.000 19.500 15.010 1.00 0.00

ATOM 11 C11 KIH 1 15.450 20.180 20.670 1.00 0.00

ATOM 12 C12 KIH 1 15.070 19.120 21.450 1.00 0.00

ATOM 13 C13 KIH 1 15.080 19.160 22.830 1.00 0.00

ATOM 14 C14 KIH 1 14.650 18.090 23.570 1.00 0.00

ATOM 15 C15 KIH 1 14.390 16.820 23.140 1.00 0.00

ATOM 16 C16 KIH 1 13.990 15.760 23.920 1.00 0.00

ATOM 17 C17 KIH 1 13.830 16.000 25.260 1.00 0.00

ATOM 18 C18 KIH 1 14.260 17.200 25.790 1.00 0.00

ATOM 19 C19 KIH 1 14.640 18.220 24.950 1.00 0.00

ATOM 20 C20 KIH 1 15.240 19.320 25.520 1.00 0.00

ATOM 21 C21 KIH 1 15.640 20.390 24.760 1.00 0.00

ATOM 22 C22 KIH 1 15.530 20.340 23.400 1.00 0.00

ATOM 23 C23 KIH 1 16.060 21.390 22.670 1.00 0.00

ATOM 24 C24 KIH 1 15.960 21.290 21.300 1.00 0.00

ATOM 25 H1 KIH 1 13.330 19.430 19.210 1.00 0.00

ATOM 26 H2 KIH 1 11.940 18.870 17.260 1.00 0.00

ATOM 27 H3 KIH 1 11.820 18.670 14.820 1.00 0.00

ATOM 28 H4 KIH 1 13.700 19.040 13.390 1.00 0.00

ATOM 29 H5 KIH 1 15.770 19.780 14.320 1.00 0.00

ATOM 30 H6 KIH 1 14.750 18.230 20.930 1.00 0.00

ATOM 31 H7 KIH 1 14.640 16.630 22.110 1.00 0.00

ATOM 32 H8 KIH 1 13.830 14.790 23.470 1.00 0.00

ATOM 33 H9 KIH 1 13.540 15.180 25.900 1.00 0.00

ATOM 34 H10 KIH 1 14.300 17.260 26.860 1.00 0.00

ATOM 35 H11 KIH 1 15.370 19.440 26.590 1.00 0.00

ATOM 36 H12 KIH 1 16.020 21.240 25.300 1.00 0.00

ATOM 37 H13 KIH 1 16.590 22.200 23.140 1.00 0.00

ATOM 38 H14 KIH 1 16.360 22.150 20.790 1.00 0.00

ATOM 39 O1 KIH 1 17.640 20.380 18.860 1.00 0.00

ATOM 40 O2 KIH 1 19.020 18.630 22.670 1.00 0.00

ATOM 41 C25 KIH 1 17.580 20.340 16.260 1.00 0.00

ATOM 42 C26 KIH 1 18.630 19.490 16.530 1.00 0.00

ATOM 43 C27 KIH 1 19.830 19.790 15.920 1.00 0.00

ATOM 44 C28 KIH 1 19.960 20.890 15.100 1.00 0.00

ATOM 45 C29 KIH 1 18.870 21.670 14.810 1.00 0.00

ATOM 46 C30 KIH 1 17.670 21.430 15.420 1.00 0.00

ATOM 47 C31 KIH 1 19.050 22.840 14.100 1.00 0.00

ATOM 48 C32 KIH 1 18.020 23.740 14.010 1.00 0.00

ATOM 49 C33 KIH 1 16.780 23.470 14.550 1.00 0.00

ATOM 50 C34 KIH 1 16.620 22.310 15.280 1.00 0.00

ATOM 51 C35 KIH 1 20.930 18.800 16.040 1.00 0.00

ATOM 52 C36 KIH 1 20.840 17.490 15.620 1.00 0.00

ATOM 53 C37 KIH 1 21.920 16.640 15.500 1.00 0.00

ATOM 54 C38 KIH 1 21.860 15.400 14.900 1.00 0.00

ATOM 55 C39 KIH 1 20.780 14.960 14.180 1.00 0.00

ATOM 56 C40 KIH 1 20.620 13.660 13.790 1.00 0.00

ATOM 57 C41 KIH 1 21.680 12.800 14.000 1.00 0.00

ATOM 58 C42 KIH 1 22.870 13.230 14.560 1.00 0.00

ATOM 59 C43 KIH 1 22.920 14.530 15.000 1.00 0.00

ATOM 60 C44 KIH 1 24.060 14.910 15.680 1.00 0.00

ATOM 61 C45 KIH 1 24.180 16.220 16.090 1.00 0.00

ATOM 62 C46 KIH 1 23.090 17.060 16.080 1.00 0.00

ATOM 63 C47 KIH 1 23.240 18.340 16.550 1.00 0.00

ATOM 64 C48 KIH 1 22.170 19.210 16.490 1.00 0.00

ATOM 65 H15 KIH 1 20.930 21.030 14.660 1.00 0.00

ATOM 66 H16 KIH 1 19.940 23.110 13.550 1.00 0.00

ATOM 67 H17 KIH 1 18.130 24.660 13.440 1.00 0.00

ATOM 68 H18 KIH 1 15.950 24.160 14.510 1.00 0.00

ATOM 69 H19 KIH 1 15.640 22.160 15.700 1.00 0.00

ATOM 70 H20 KIH 1 19.870 17.250 15.220 1.00 0.00

ATOM 71 H21 KIH 1 19.900 15.580 14.010 1.00 0.00

ATOM 72 H22 KIH 1 19.730 13.300 13.300 1.00 0.00

ATOM 73 H23 KIH 1 21.510 11.800 13.640 1.00 0.00

ATOM 74 H24 KIH 1 23.650 12.490 14.570 1.00 0.00

ATOM 75 H25 KIH 1 24.890 14.220 15.670 1.00 0.00

ATOM 76 H26 KIH 1 25.130 16.560 16.470 1.00 0.00

ATOM 77 H27 KIH 1 24.190 18.580 17.000 1.00 0.00

ATOM 78 H28 KIH 1 22.400 20.240 16.700 1.00 0.00

ATOM 79 O3 KIH 1 18.670 18.620 17.610 1.00 0.00

ATOM 80 O4 KIH 1 17.620 16.550 22.500 1.00 0.00

ATOM 81 C49 KIH 1 20.020 16.500 21.730 1.00 0.00

ATOM 82 F1 KIH 1 19.900 15.420 20.960 1.00 0.00

ATOM 83 F2 KIH 1 20.960 17.280 21.190 1.00 0.00

ATOM 84 F3 KIH 1 20.520 15.980 22.860 1.00 0.00

ATOM 85 N1 KIH 1 18.090 18.060 20.380 1.00 0.00

ATOM 86 P1 KIH 1 18.640 19.170 19.080 1.00 0.00

ATOM 87 S1 KIH 1 18.540 17.500 21.920 1.00 0.00

ATOM 88 C50 KIH 1 24.020 21.500 19.420 1.00 0.00

ATOM 89 C51 KIH 1 23.570 20.260 19.790 1.00 0.00

ATOM 90 C52 KIH 1 24.350 19.550 20.690 1.00 0.00

ATOM 91 C53 KIH 1 25.530 20.080 21.150 1.00 0.00

ATOM 92 C54 KIH 1 25.960 21.330 20.820 1.00 0.00

ATOM 93 C55 KIH 1 25.210 22.000 19.890 1.00 0.00

ATOM 94 C56 KIH 1 27.050 21.860 21.470 1.00 0.00

ATOM 95 C57 KIH 1 27.510 23.090 21.070 1.00 0.00

ATOM 96 C58 KIH 1 26.840 23.850 20.130 1.00 0.00

ATOM 97 C59 KIH 1 25.700 23.270 19.620 1.00 0.00

ATOM 98 C60 KIH 1 23.990 18.170 21.100 1.00 0.00

ATOM 99 C61 KIH 1 23.830 17.850 22.430 1.00 0.00

ATOM 100 C62 KIH 1 23.540 16.580 22.860 1.00 0.00

ATOM 101 C63 KIH 1 23.380 16.210 24.180 1.00 0.00

ATOM 102 C64 KIH 1 23.470 17.080 25.240 1.00 0.00

ATOM 103 C65 KIH 1 23.360 16.700 26.550 1.00 0.00

ATOM 104 C66 KIH 1 22.960 15.410 26.810 1.00 0.00

ATOM 105 C67 KIH 1 22.770 14.510 25.790 1.00 0.00

ATOM 106 C68 KIH 1 23.040 14.910 24.490 1.00 0.00

ATOM 107 C69 KIH 1 23.050 13.940 23.520 1.00 0.00

ATOM 108 C70 KIH 1 23.290 14.250 22.200 1.00 0.00

ATOM 109 C71 KIH 1 23.510 15.580 21.920 1.00 0.00

ATOM 110 C72 KIH 1 23.640 15.910 20.590 1.00 0.00

ATOM 111 C73 KIH 1 23.870 17.190 20.160 1.00 0.00

ATOM 112 H29 KIH 1 26.110 19.470 21.830 1.00 0.00

ATOM 113 H30 KIH 1 27.610 21.310 22.220 1.00 0.00

ATOM 114 H31 KIH 1 28.240 23.620 21.650 1.00 0.00

ATOM 115 H32 KIH 1 27.130 24.860 19.960 1.00 0.00

ATOM 116 H33 KIH 1 25.130 23.830 18.900 1.00 0.00

ATOM 117 H34 KIH 1 23.940 18.640 23.160 1.00 0.00

ATOM 118 H35 KIH 1 23.820 18.100 25.070 1.00 0.00

ATOM 119 H36 KIH 1 23.520 17.390 27.360 1.00 0.00

ATOM 120 H37 KIH 1 22.740 15.090 27.830 1.00 0.00

ATOM 121 H38 KIH 1 22.530 13.500 26.060 1.00 0.00

ATOM 122 H39 KIH 1 22.860 12.880 23.700 1.00 0.00

ATOM 123 H40 KIH 1 23.360 13.470 21.460 1.00 0.00

ATOM 124 H41 KIH 1 23.470 15.180 19.800 1.00 0.00

ATOM 125 H42 KIH 1 23.960 17.350 19.080 1.00 0.00

ATOM 126 O5 KIH 1 22.290 19.780 19.590 1.00 0.00

ATOM 127 O6 KIH 1 21.780 19.150 23.860 1.00 0.00

ATOM 128 C74 KIH 1 23.170 22.300 18.490 1.00 0.00

ATOM 129 C75 KIH 1 21.920 22.610 18.970 1.00 0.00

ATOM 130 C76 KIH 1 21.180 23.550 18.290 1.00 0.00

ATOM 131 C77 KIH 1 21.560 24.090 17.090 1.00 0.00

ATOM 132 C78 KIH 1 22.700 23.580 16.510 1.00 0.00

ATOM 133 C79 KIH 1 23.540 22.790 17.250 1.00 0.00

ATOM 134 C80 KIH 1 23.070 23.920 15.230 1.00 0.00

ATOM 135 C81 KIH 1 24.240 23.450 14.690 1.00 0.00

ATOM 136 C82 KIH 1 25.070 22.640 15.440 1.00 0.00

ATOM 137 C83 KIH 1 24.730 22.320 16.730 1.00 0.00

ATOM 138 C84 KIH 1 19.840 23.990 18.790 1.00 0.00

ATOM 139 C85 KIH 1 19.700 24.680 19.970 1.00 0.00

ATOM 140 C86 KIH 1 18.490 25.080 20.500 1.00 0.00

ATOM 141 C87 KIH 1 18.380 25.780 21.680 1.00 0.00

ATOM 142 C88 KIH 1 19.460 26.280 22.350 1.00 0.00

ATOM 143 C89 KIH 1 19.320 27.060 23.470 1.00 0.00

ATOM 144 C90 KIH 1 18.070 27.250 24.040 1.00 0.00

ATOM 145 C91 KIH 1 16.960 26.840 23.330 1.00 0.00

ATOM 146 C92 KIH 1 17.140 26.110 22.180 1.00 0.00

ATOM 147 C93 KIH 1 15.980 25.630 21.610 1.00 0.00

ATOM 148 C94 KIH 1 16.090 24.940 20.430 1.00 0.00

ATOM 149 C95 KIH 1 17.330 24.600 19.940 1.00 0.00

ATOM 150 C96 KIH 1 17.440 23.860 18.790 1.00 0.00

ATOM 151 C97 KIH 1 18.660 23.610 18.200 1.00 0.00

ATOM 152 H43 KIH 1 20.930 24.790 16.560 1.00 0.00

ATOM 153 H44 KIH 1 22.420 24.610 14.700 1.00 0.00

ATOM 154 H45 KIH 1 24.750 23.870 13.830 1.00 0.00

ATOM 155 H46 KIH 1 26.030 22.360 15.060 1.00 0.00

ATOM 156 H47 KIH 1 25.480 21.670 17.170 1.00 0.00

ATOM 157 H48 KIH 1 20.610 24.910 20.500 1.00 0.00

ATOM 158 H49 KIH 1 20.500 26.240 22.070 1.00 0.00

ATOM 159 H50 KIH 1 20.200 27.440 23.950 1.00 0.00

ATOM 160 H51 KIH 1 17.840 27.830 24.910 1.00 0.00

ATOM 161 H52 KIH 1 15.960 27.100 23.670 1.00 0.00

ATOM 162 H53 KIH 1 15.010 25.790 22.050 1.00 0.00

ATOM 163 H54 KIH 1 15.210 24.580 19.910 1.00 0.00

ATOM 164 H55 KIH 1 16.630 23.350 18.290 1.00 0.00

ATOM 165 H56 KIH 1 18.680 23.030 17.290 1.00 0.00

ATOM 166 O7 KIH 1 21.330 22.070 20.100 1.00 0.00

ATOM 167 O8 KIH 1 22.960 21.170 22.930 1.00 0.00

ATOM 168 C98 KIH 1 20.660 21.540 24.090 1.00 0.00

ATOM 169 F4 KIH 1 21.230 21.940 25.230 1.00 0.00

ATOM 170 F5 KIH 1 20.360 22.650 23.410 1.00 0.00

ATOM 171 F6 KIH 1 19.510 20.940 24.420 1.00 0.00

ATOM 172 N2 KIH 1 21.030 20.080 21.780 1.00 0.00

ATOM 173 P2 KIH 1 21.030 20.530 20.190 1.00 0.00

ATOM 174 S2 KIH 1 21.760 20.430 23.210 1.00 0.00

ATOM 175 N3 KIH 1 19.890 19.930 19.430 1.00 0.00

ATOM 176 H57 KIH 1 20.460 19.240 21.890 1.00 0.00

TER

ENDMDL

REMARK GENERATED BY TRJCONV

TITLE KIH t= 2500.00000

REMARK THIS IS A SIMULATION BOX

CRYST1 38.518 38.518 38.518 90.00 90.00 90.00 P 1 1

MODEL 6

ATOM 1 C1 KIH 1 16.170 19.730 17.060 1.00 0.00

ATOM 2 C2 KIH 1 16.250 20.080 18.390 1.00 0.00

ATOM 3 C3 KIH 1 15.160 19.860 19.180 1.00 0.00

ATOM 4 C4 KIH 1 13.970 19.400 18.660 1.00 0.00

ATOM 5 C5 KIH 1 13.890 19.140 17.310 1.00 0.00

ATOM 6 C6 KIH 1 15.000 19.250 16.520 1.00 0.00

ATOM 7 C7 KIH 1 12.650 18.850 16.770 1.00 0.00

ATOM 8 C8 KIH 1 12.530 18.570 15.430 1.00 0.00

ATOM 9 C9 KIH 1 13.670 18.630 14.650 1.00 0.00

ATOM 10 C10 KIH 1 14.880 19.000 15.170 1.00 0.00

ATOM 11 C11 KIH 1 15.240 20.070 20.660 1.00 0.00

ATOM 12 C12 KIH 1 15.130 19.020 21.540 1.00 0.00

ATOM 13 C13 KIH 1 15.110 19.160 22.910 1.00 0.00

ATOM 14 C14 KIH 1 14.940 18.150 23.830 1.00 0.00

ATOM 15 C15 KIH 1 14.640 16.880 23.420 1.00 0.00

ATOM 16 C16 KIH 1 14.450 15.870 24.330 1.00 0.00

ATOM 17 C17 KIH 1 14.430 16.180 25.670 1.00 0.00

ATOM 18 C18 KIH 1 14.690 17.450 26.130 1.00 0.00

ATOM 19 C19 KIH 1 14.910 18.430 25.180 1.00 0.00

ATOM 20 C20 KIH 1 15.130 19.710 25.610 1.00 0.00

ATOM 21 C21 KIH 1 15.290 20.750 24.720 1.00 0.00

ATOM 22 C22 KIH 1 15.260 20.450 23.390 1.00 0.00

ATOM 23 C23 KIH 1 15.510 21.520 22.550 1.00 0.00

ATOM 24 C24 KIH 1 15.510 21.310 21.180 1.00 0.00

ATOM 25 H1 KIH 1 13.140 19.440 19.360 1.00 0.00

ATOM 26 H2 KIH 1 11.750 18.850 17.360 1.00 0.00

ATOM 27 H3 KIH 1 11.570 18.350 14.990 1.00 0.00

ATOM 28 H4 KIH 1 13.690 18.400 13.590 1.00 0.00

ATOM 29 H5 KIH 1 15.740 19.100 14.530 1.00 0.00

ATOM 30 H6 KIH 1 15.000 18.070 21.040 1.00 0.00

ATOM 31 H7 KIH 1 14.670 16.530 22.400 1.00 0.00

ATOM 32 H8 KIH 1 14.280 14.850 24.040 1.00 0.00

ATOM 33 H9 KIH 1 14.230 15.400 26.380 1.00 0.00

ATOM 34 H10 KIH 1 14.600 17.740 27.160 1.00 0.00

ATOM 35 H11 KIH 1 15.130 19.900 26.670 1.00 0.00

ATOM 36 H12 KIH 1 15.560 21.740 25.080 1.00 0.00

ATOM 37 H13 KIH 1 15.750 22.510 22.880 1.00 0.00

ATOM 38 H14 KIH 1 15.780 22.120 20.520 1.00 0.00

ATOM 39 O1 KIH 1 17.480 20.310 19.000 1.00 0.00

ATOM 40 O2 KIH 1 19.120 18.460 22.720 1.00 0.00

ATOM 41 C25 KIH 1 17.410 19.980 16.270 1.00 0.00

ATOM 42 C26 KIH 1 18.570 19.280 16.540 1.00 0.00

ATOM 43 C27 KIH 1 19.690 19.610 15.820 1.00 0.00

ATOM 44 C28 KIH 1 19.650 20.670 14.950 1.00 0.00

ATOM 45 C29 KIH 1 18.520 21.440 14.790 1.00 0.00

ATOM 46 C30 KIH 1 17.370 21.080 15.460 1.00 0.00

ATOM 47 C31 KIH 1 18.540 22.560 14.000 1.00 0.00

ATOM 48 C32 KIH 1 17.450 23.400 13.900 1.00 0.00

ATOM 49 C33 KIH 1 16.300 23.090 14.580 1.00 0.00

ATOM 50 C34 KIH 1 16.310 21.950 15.350 1.00 0.00

ATOM 51 C35 KIH 1 20.980 18.860 15.920 1.00 0.00

ATOM 52 C36 KIH 1 21.100 17.540 15.580 1.00 0.00

ATOM 53 C37 KIH 1 22.290 16.840 15.620 1.00 0.00

ATOM 54 C38 KIH 1 22.400 15.540 15.190 1.00 0.00

ATOM 55 C39 KIH 1 21.410 14.700 14.730 1.00 0.00

ATOM 56 C40 KIH 1 21.550 13.360 14.430 1.00 0.00

ATOM 57 C41 KIH 1 22.810 12.830 14.540 1.00 0.00

ATOM 58 C42 KIH 1 23.820 13.570 15.100 1.00 0.00

ATOM 59 C43 KIH 1 23.610 14.900 15.380 1.00 0.00

ATOM 60 C44 KIH 1 24.690 15.560 15.910 1.00 0.00

ATOM 61 C45 KIH 1 24.610 16.890 16.240 1.00 0.00

ATOM 62 C46 KIH 1 23.390 17.510 16.120 1.00 0.00

ATOM 63 C47 KIH 1 23.270 18.820 16.460 1.00 0.00

ATOM 64 C48 KIH 1 22.080 19.510 16.420 1.00 0.00

ATOM 65 H15 KIH 1 20.530 20.860 14.360 1.00 0.00

ATOM 66 H16 KIH 1 19.450 22.750 13.450 1.00 0.00

ATOM 67 H17 KIH 1 17.440 24.250 13.220 1.00 0.00

ATOM 68 H18 KIH 1 15.390 23.660 14.500 1.00 0.00

ATOM 69 H19 KIH 1 15.380 21.720 15.850 1.00 0.00

ATOM 70 H20 KIH 1 20.220 17.010 15.260 1.00 0.00

ATOM 71 H21 KIH 1 20.390 15.030 14.590 1.00 0.00

ATOM 72 H22 KIH 1 20.700 12.850 13.990 1.00 0.00

ATOM 73 H23 KIH 1 22.960 11.790 14.260 1.00 0.00

ATOM 74 H24 KIH 1 24.750 13.130 15.410 1.00 0.00

ATOM 75 H25 KIH 1 25.610 15.010 16.010 1.00 0.00

ATOM 76 H26 KIH 1 25.560 17.360 16.460 1.00 0.00

ATOM 77 H27 KIH 1 24.150 19.320 16.850 1.00 0.00

ATOM 78 H28 KIH 1 22.030 20.530 16.780 1.00 0.00

ATOM 79 O3 KIH 1 18.710 18.510 17.680 1.00 0.00

ATOM 80 O4 KIH 1 17.480 16.600 22.470 1.00 0.00

ATOM 81 C49 KIH 1 19.920 16.380 21.570 1.00 0.00

ATOM 82 F1 KIH 1 19.660 15.260 20.880 1.00 0.00

ATOM 83 F2 KIH 1 20.940 16.990 20.960 1.00 0.00

ATOM 84 F3 KIH 1 20.410 16.060 22.780 1.00 0.00

ATOM 85 N1 KIH 1 18.130 18.100 20.390 1.00 0.00

ATOM 86 P1 KIH 1 18.610 19.230 19.080 1.00 0.00

ATOM 87 S1 KIH 1 18.510 17.440 21.910 1.00 0.00

ATOM 88 C50 KIH 1 23.890 21.850 19.320 1.00 0.00

ATOM 89 C51 KIH 1 23.580 20.570 19.750 1.00 0.00

ATOM 90 C52 KIH 1 24.570 19.820 20.330 1.00 0.00

ATOM 91 C53 KIH 1 25.700 20.430 20.810 1.00 0.00

ATOM 92 C54 KIH 1 25.880 21.790 20.650 1.00 0.00

ATOM 93 C55 KIH 1 25.030 22.460 19.800 1.00 0.00

ATOM 94 C56 KIH 1 26.980 22.480 21.120 1.00 0.00

ATOM 95 C57 KIH 1 27.230 23.790 20.790 1.00 0.00

ATOM 96 C58 KIH 1 26.330 24.450 20.000 1.00 0.00

ATOM 97 C59 KIH 1 25.260 23.770 19.470 1.00 0.00

ATOM 98 C60 KIH 1 24.250 18.430 20.760 1.00 0.00

ATOM 99 C61 KIH 1 24.300 18.110 22.100 1.00 0.00

ATOM 100 C62 KIH 1 24.140 16.880 22.680 1.00 0.00

ATOM 101 C63 KIH 1 24.140 16.660 24.040 1.00 0.00

ATOM 102 C64 KIH 1 24.270 17.650 24.980 1.00 0.00

ATOM 103 C65 KIH 1 24.190 17.400 26.330 1.00 0.00

ATOM 104 C66 KIH 1 23.970 16.100 26.730 1.00 0.00

ATOM 105 C67 KIH 1 23.980 15.070 25.820 1.00 0.00

ATOM 106 C68 KIH 1 24.000 15.370 24.480 1.00 0.00

ATOM 107 C69 KIH 1 23.760 14.380 23.550 1.00 0.00

ATOM 108 C70 KIH 1 23.720 14.590 22.190 1.00 0.00

ATOM 109 C71 KIH 1 24.000 15.860 21.750 1.00 0.00

ATOM 110 C72 KIH 1 23.910 16.120 20.410 1.00 0.00

ATOM 111 C73 KIH 1 24.020 17.390 19.890 1.00 0.00

ATOM 112 H29 KIH 1 26.490 19.890 21.310 1.00 0.00

ATOM 113 H30 KIH 1 27.730 22.010 21.730 1.00 0.00

ATOM 114 H31 KIH 1 28.000 24.360 21.300 1.00 0.00

ATOM 115 H32 KIH 1 26.640 25.460 19.780 1.00 0.00

ATOM 116 H33 KIH 1 24.620 24.300 18.770 1.00 0.00

ATOM 117 H34 KIH 1 24.410 18.980 22.730 1.00 0.00

ATOM 118 H35 KIH 1 24.450 18.670 24.690 1.00 0.00

ATOM 119 H36 KIH 1 24.400 18.250 26.960 1.00 0.00

ATOM 120 H37 KIH 1 23.870 15.890 27.790 1.00 0.00

ATOM 121 H38 KIH 1 23.840 14.040 26.130 1.00 0.00

ATOM 122 H39 KIH 1 23.480 13.370 23.810 1.00 0.00

ATOM 123 H40 KIH 1 23.560 13.760 21.510 1.00 0.00

ATOM 124 H41 KIH 1 23.640 15.370 19.680 1.00 0.00

ATOM 125 H42 KIH 1 23.920 17.570 18.830 1.00 0.00

ATOM 126 O5 KIH 1 22.360 19.960 19.450 1.00 0.00

ATOM 127 O6 KIH 1 21.830 19.390 24.040 1.00 0.00

ATOM 128 C74 KIH 1 22.810 22.540 18.580 1.00 0.00

ATOM 129 C75 KIH 1 21.590 22.750 19.180 1.00 0.00

ATOM 130 C76 KIH 1 20.590 23.380 18.460 1.00 0.00

ATOM 131 C77 KIH 1 20.870 23.830 17.200 1.00 0.00

ATOM 132 C78 KIH 1 22.040 23.520 16.540 1.00 0.00

ATOM 133 C79 KIH 1 23.010 22.870 17.250 1.00 0.00

ATOM 134 C80 KIH 1 22.250 23.850 15.230 1.00 0.00

ATOM 135 C81 KIH 1 23.400 23.560 14.540 1.00 0.00

ATOM 136 C82 KIH 1 24.370 22.930 15.270 1.00 0.00

ATOM 137 C83 KIH 1 24.190 22.580 16.600 1.00 0.00

ATOM 138 C84 KIH 1 19.210 23.620 18.970 1.00 0.00

ATOM 139 C85 KIH 1 19.060 24.140 20.230 1.00 0.00

ATOM 140 C86 KIH 1 17.880 24.620 20.780 1.00 0.00

ATOM 141 C87 KIH 1 17.820 25.350 21.940 1.00 0.00

ATOM 142 C88 KIH 1 18.900 25.640 22.730 1.00 0.00

ATOM 143 C89 KIH 1 18.870 26.500 23.810 1.00 0.00

ATOM 144 C90 KIH 1 17.670 27.090 24.150 1.00 0.00

ATOM 145 C91 KIH 1 16.570 26.810 23.370 1.00 0.00

ATOM 146 C92 KIH 1 16.650 25.960 22.300 1.00 0.00

ATOM 147 C93 KIH 1 15.560 25.890 21.460 1.00 0.00

ATOM 148 C94 KIH 1 15.590 25.120 20.310 1.00 0.00

ATOM 149 C95 KIH 1 16.770 24.460 20.000 1.00 0.00

ATOM 150 C96 KIH 1 16.860 23.880 18.750 1.00 0.00

ATOM 151 C97 KIH 1 18.060 23.450 18.230 1.00 0.00

ATOM 152 H43 KIH 1 20.080 24.280 16.620 1.00 0.00

ATOM 153 H44 KIH 1 21.580 24.460 14.630 1.00 0.00

ATOM 154 H45 KIH 1 23.670 23.910 13.550 1.00 0.00

ATOM 155 H46 KIH 1 25.300 22.640 14.810 1.00 0.00

ATOM 156 H47 KIH 1 25.030 22.060 17.040 1.00 0.00

ATOM 157 H48 KIH 1 20.000 24.150 20.760 1.00 0.00

ATOM 158 H49 KIH 1 19.840 25.130 22.600 1.00 0.00

ATOM 159 H50 KIH 1 19.650 26.610 24.530 1.00 0.00

ATOM 160 H51 KIH 1 17.720 27.720 25.030 1.00 0.00

ATOM 161 H52 KIH 1 15.640 27.310 23.620 1.00 0.00

ATOM 162 H53 KIH 1 14.660 26.420 21.740 1.00 0.00

ATOM 163 H54 KIH 1 14.700 25.020 19.720 1.00 0.00

ATOM 164 H55 KIH 1 15.980 23.720 18.140 1.00 0.00

ATOM 165 H56 KIH 1 18.190 23.060 17.230 1.00 0.00

ATOM 166 O7 KIH 1 21.240 22.060 20.320 1.00 0.00

ATOM 167 O8 KIH 1 22.900 21.240 22.730 1.00 0.00

ATOM 168 C98 KIH 1 20.540 21.650 23.930 1.00 0.00

ATOM 169 F4 KIH 1 20.900 22.130 25.130 1.00 0.00

ATOM 170 F5 KIH 1 20.390 22.760 23.200 1.00 0.00

ATOM 171 F6 KIH 1 19.300 21.140 23.970 1.00 0.00

ATOM 172 N2 KIH 1 21.120 19.930 21.750 1.00 0.00

ATOM 173 P2 KIH 1 21.080 20.490 20.210 1.00 0.00

ATOM 174 S2 KIH 1 21.720 20.520 23.170 1.00 0.00

ATOM 175 N3 KIH 1 19.860 19.940 19.530 1.00 0.00

ATOM 176 H57 KIH 1 20.500 19.130 21.860 1.00 0.00

TER

ENDMDL

REMARK GENERATED BY TRJCONV

TITLE KIH t= 3000.00000

REMARK THIS IS A SIMULATION BOX

CRYST1 38.484 38.484 38.484 90.00 90.00 90.00 P 1 1

MODEL 7

ATOM 1 C1 KIH 1 15.970 19.880 17.450 1.00 0.00

ATOM 2 C2 KIH 1 16.220 20.080 18.780 1.00 0.00

ATOM 3 C3 KIH 1 15.240 19.850 19.720 1.00 0.00

ATOM 4 C4 KIH 1 14.060 19.310 19.270 1.00 0.00

ATOM 5 C5 KIH 1 13.830 18.950 17.960 1.00 0.00

ATOM 6 C6 KIH 1 14.800 19.260 17.040 1.00 0.00

ATOM 7 C7 KIH 1 12.650 18.360 17.560 1.00 0.00

ATOM 8 C8 KIH 1 12.410 18.150 16.220 1.00 0.00

ATOM 9 C9 KIH 1 13.410 18.390 15.310 1.00 0.00

ATOM 10 C10 KIH 1 14.610 18.900 15.740 1.00 0.00

ATOM 11 C11 KIH 1 15.280 20.210 21.160 1.00 0.00

ATOM 12 C12 KIH 1 15.020 19.240 22.100 1.00 0.00

ATOM 13 C13 KIH 1 15.010 19.410 23.460 1.00 0.00

ATOM 14 C14 KIH 1 14.640 18.470 24.390 1.00 0.00

ATOM 15 C15 KIH 1 14.230 17.200 24.040 1.00 0.00

ATOM 16 C16 KIH 1 13.940 16.180 24.920 1.00 0.00

ATOM 17 C17 KIH 1 14.030 16.440 26.260 1.00 0.00

ATOM 18 C18 KIH 1 14.210 17.760 26.610 1.00 0.00

ATOM 19 C19 KIH 1 14.650 18.730 25.740 1.00 0.00

ATOM 20 C20 KIH 1 14.940 20.020 26.120 1.00 0.00

ATOM 21 C21 KIH 1 15.240 21.000 25.190 1.00 0.00

ATOM 22 C22 KIH 1 15.290 20.700 23.850 1.00 0.00

ATOM 23 C23 KIH 1 15.670 21.720 23.000 1.00 0.00

ATOM 24 C24 KIH 1 15.660 21.440 21.660 1.00 0.00

ATOM 25 H1 KIH 1 13.310 19.060 20.010 1.00 0.00

ATOM 26 H2 KIH 1 11.850 18.130 18.230 1.00 0.00

ATOM 27 H3 KIH 1 11.460 17.690 15.980 1.00 0.00

ATOM 28 H4 KIH 1 13.380 18.180 14.250 1.00 0.00

ATOM 29 H5 KIH 1 15.400 18.960 15.000 1.00 0.00

ATOM 30 H6 KIH 1 14.730 18.290 21.680 1.00 0.00

ATOM 31 H7 KIH 1 14.090 16.870 23.030 1.00 0.00

ATOM 32 H8 KIH 1 13.740 15.160 24.620 1.00 0.00

ATOM 33 H9 KIH 1 13.850 15.720 27.040 1.00 0.00

ATOM 34 H10 KIH 1 14.030 18.040 27.640 1.00 0.00

ATOM 35 H11 KIH 1 15.080 20.260 27.160 1.00 0.00

ATOM 36 H12 KIH 1 15.580 21.970 25.540 1.00 0.00

ATOM 37 H13 KIH 1 16.050 22.660 23.370 1.00 0.00

ATOM 38 H14 KIH 1 15.880 22.250 20.970 1.00 0.00

ATOM 39 O1 KIH 1 17.470 20.480 19.190 1.00 0.00

ATOM 40 O2 KIH 1 19.540 17.990 22.630 1.00 0.00

ATOM 41 C25 KIH 1 17.090 20.280 16.540 1.00 0.00

ATOM 42 C26 KIH 1 18.270 19.570 16.680 1.00 0.00

ATOM 43 C27 KIH 1 19.310 19.690 15.800 1.00 0.00

ATOM 44 C28 KIH 1 19.230 20.650 14.810 1.00 0.00

ATOM 45 C29 KIH 1 18.080 21.390 14.720 1.00 0.00

ATOM 46 C30 KIH 1 17.010 21.220 15.560 1.00 0.00

ATOM 47 C31 KIH 1 18.040 22.410 13.790 1.00 0.00

ATOM 48 C32 KIH 1 16.920 23.190 13.630 1.00 0.00

ATOM 49 C33 KIH 1 15.810 22.970 14.420 1.00 0.00

ATOM 50 C34 KIH 1 15.920 22.060 15.450 1.00 0.00

ATOM 51 C35 KIH 1 20.560 18.880 15.900 1.00 0.00

ATOM 52 C36 KIH 1 20.580 17.590 15.440 1.00 0.00

ATOM 53 C37 KIH 1 21.770 16.900 15.350 1.00 0.00

ATOM 54 C38 KIH 1 21.830 15.580 14.950 1.00 0.00

ATOM 55 C39 KIH 1 20.760 14.850 14.480 1.00 0.00

ATOM 56 C40 KIH 1 20.790 13.550 14.020 1.00 0.00

ATOM 57 C41 KIH 1 22.020 12.970 13.870 1.00 0.00

ATOM 58 C42 KIH 1 23.120 13.700 14.260 1.00 0.00

ATOM 59 C43 KIH 1 23.050 14.960 14.800 1.00 0.00

ATOM 60 C44 KIH 1 24.180 15.520 15.340 1.00 0.00

ATOM 61 C45 KIH 1 24.090 16.790 15.860 1.00 0.00

ATOM 62 C46 KIH 1 22.910 17.480 15.850 1.00 0.00

ATOM 63 C47 KIH 1 22.910 18.780 16.310 1.00 0.00

ATOM 64 C48 KIH 1 21.720 19.460 16.360 1.00 0.00

ATOM 65 H15 KIH 1 20.050 20.700 14.110 1.00 0.00

ATOM 66 H16 KIH 1 18.960 22.600 13.230 1.00 0.00

ATOM 67 H17 KIH 1 16.670 23.870 12.840 1.00 0.00

ATOM 68 H18 KIH 1 14.980 23.640 14.310 1.00 0.00

ATOM 69 H19 KIH 1 15.080 21.920 16.120 1.00 0.00

ATOM 70 H20 KIH 1 19.660 17.110 15.170 1.00 0.00

ATOM 71 H21 KIH 1 19.760 15.250 14.450 1.00 0.00

ATOM 72 H22 KIH 1 19.920 13.040 13.620 1.00 0.00

ATOM 73 H23 KIH 1 22.120 11.900 13.750 1.00 0.00

ATOM 74 H24 KIH 1 24.070 13.200 14.160 1.00 0.00

ATOM 75 H25 KIH 1 25.110 14.980 15.250 1.00 0.00

ATOM 76 H26 KIH 1 25.000 17.190 16.300 1.00 0.00

ATOM 77 H27 KIH 1 23.810 19.230 16.710 1.00 0.00

ATOM 78 H28 KIH 1 21.700 20.430 16.820 1.00 0.00

ATOM 79 O3 KIH 1 18.460 18.720 17.750 1.00 0.00

ATOM 80 O4 KIH 1 17.490 16.720 22.600 1.00 0.00

ATOM 81 C49 KIH 1 19.630 15.980 21.210 1.00 0.00

ATOM 82 F1 KIH 1 19.100 15.260 20.210 1.00 0.00

ATOM 83 F2 KIH 1 20.740 16.530 20.680 1.00 0.00

ATOM 84 F3 KIH 1 20.050 15.090 22.110 1.00 0.00

ATOM 85 N1 KIH 1 18.060 18.310 20.570 1.00 0.00

ATOM 86 P1 KIH 1 18.570 19.340 19.200 1.00 0.00

ATOM 87 S1 KIH 1 18.570 17.290 21.830 1.00 0.00

ATOM 88 C50 KIH 1 24.010 21.600 19.420 1.00 0.00

ATOM 89 C51 KIH 1 23.730 20.290 19.710 1.00 0.00

ATOM 90 C52 KIH 1 24.620 19.550 20.460 1.00 0.00

ATOM 91 C53 KIH 1 25.770 20.130 20.940 1.00 0.00

ATOM 92 C54 KIH 1 26.000 21.470 20.730 1.00 0.00

ATOM 93 C55 KIH 1 25.140 22.190 19.940 1.00 0.00

ATOM 94 C56 KIH 1 27.040 22.050 21.420 1.00 0.00

ATOM 95 C57 KIH 1 27.410 23.320 21.050 1.00 0.00

ATOM 96 C58 KIH 1 26.590 24.070 20.260 1.00 0.00

ATOM 97 C59 KIH 1 25.450 23.520 19.730 1.00 0.00

ATOM 98 C60 KIH 1 24.250 18.170 20.880 1.00 0.00

ATOM 99 C61 KIH 1 24.060 17.810 22.190 1.00 0.00

ATOM 100 C62 KIH 1 23.700 16.530 22.570 1.00 0.00

ATOM 101 C63 KIH 1 23.380 16.180 23.860 1.00 0.00

ATOM 102 C64 KIH 1 23.420 17.090 24.900 1.00 0.00

ATOM 103 C65 KIH 1 23.190 16.710 26.190 1.00 0.00

ATOM 104 C66 KIH 1 22.750 15.430 26.430 1.00 0.00

ATOM 105 C67 KIH 1 22.820 14.480 25.430 1.00 0.00

ATOM 106 C68 KIH 1 23.070 14.870 24.140 1.00 0.00

ATOM 107 C69 KIH 1 22.970 13.920 23.150 1.00 0.00

ATOM 108 C70 KIH 1 23.220 14.310 21.840 1.00 0.00

ATOM 109 C71 KIH 1 23.550 15.600 21.560 1.00 0.00

ATOM 110 C72 KIH 1 23.820 15.930 20.250 1.00 0.00

ATOM 111 C73 KIH 1 24.010 17.240 19.890 1.00 0.00

ATOM 112 H29 KIH 1 26.460 19.580 21.570 1.00 0.00

ATOM 113 H30 KIH 1 27.600 21.500 22.160 1.00 0.00

ATOM 114 H31 KIH 1 28.350 23.750 21.380 1.00 0.00

ATOM 115 H32 KIH 1 26.890 25.080 20.040 1.00 0.00

ATOM 116 H33 KIH 1 24.890 24.150 19.040 1.00 0.00

ATOM 117 H34 KIH 1 24.090 18.640 22.860 1.00 0.00

ATOM 118 H35 KIH 1 23.770 18.100 24.790 1.00 0.00

ATOM 119 H36 KIH 1 23.510 17.390 26.970 1.00 0.00

ATOM 120 H37 KIH 1 22.520 15.060 27.420 1.00 0.00

ATOM 121 H38 KIH 1 22.470 13.480 25.620 1.00 0.00

ATOM 122 H39 KIH 1 22.810 12.880 23.370 1.00 0.00

ATOM 123 H40 KIH 1 23.190 13.610 21.020 1.00 0.00

ATOM 124 H41 KIH 1 23.620 15.240 19.450 1.00 0.00

ATOM 125 H42 KIH 1 24.050 17.570 18.860 1.00 0.00

ATOM 126 O5 KIH 1 22.480 19.780 19.410 1.00 0.00

ATOM 127 O6 KIH 1 21.820 19.560 24.070 1.00 0.00

ATOM 128 C74 KIH 1 23.050 22.340 18.540 1.00 0.00

ATOM 129 C75 KIH 1 21.800 22.620 19.050 1.00 0.00

ATOM 130 C76 KIH 1 20.890 23.240 18.230 1.00 0.00

ATOM 131 C77 KIH 1 21.170 23.430 16.900 1.00 0.00

ATOM 132 C78 KIH 1 22.450 23.180 16.460 1.00 0.00

ATOM 133 C79 KIH 1 23.420 22.620 17.250 1.00 0.00

ATOM 134 C80 KIH 1 22.770 23.510 15.170 1.00 0.00

ATOM 135 C81 KIH 1 24.020 23.290 14.640 1.00 0.00

ATOM 136 C82 KIH 1 24.920 22.590 15.420 1.00 0.00

ATOM 137 C83 KIH 1 24.630 22.260 16.720 1.00 0.00

ATOM 138 C84 KIH 1 19.500 23.460 18.740 1.00 0.00

ATOM 139 C85 KIH 1 19.160 23.950 19.980 1.00 0.00

ATOM 140 C86 KIH 1 17.920 24.370 20.380 1.00 0.00

ATOM 141 C87 KIH 1 17.650 24.890 21.630 1.00 0.00

ATOM 142 C88 KIH 1 18.630 25.070 22.580 1.00 0.00

ATOM 143 C89 KIH 1 18.240 25.650 23.770 1.00 0.00

ATOM 144 C90 KIH 1 16.950 26.020 24.070 1.00 0.00

ATOM 145 C91 KIH 1 16.040 25.940 23.050 1.00 0.00

ATOM 146 C92 KIH 1 16.370 25.340 21.860 1.00 0.00

ATOM 147 C93 KIH 1 15.370 25.310 20.920 1.00 0.00

ATOM 148 C94 KIH 1 15.660 24.800 19.670 1.00 0.00

ATOM 149 C95 KIH 1 16.940 24.370 19.420 1.00 0.00

ATOM 150 C96 KIH 1 17.200 23.900 18.140 1.00 0.00

ATOM 151 C97 KIH 1 18.460 23.440 17.840 1.00 0.00

ATOM 152 H43 KIH 1 20.440 23.960 16.320 1.00 0.00

ATOM 153 H44 KIH 1 22.040 23.840 14.430 1.00 0.00

ATOM 154 H45 KIH 1 24.180 23.370 13.580 1.00 0.00

ATOM 155 H46 KIH 1 25.820 22.230 14.950 1.00 0.00

ATOM 156 H47 KIH 1 25.390 21.720 17.260 1.00 0.00

ATOM 157 H48 KIH 1 20.030 24.000 20.620 1.00 0.00

ATOM 158 H49 KIH 1 19.650 24.740 22.530 1.00 0.00

ATOM 159 H50 KIH 1 18.990 25.750 24.540 1.00 0.00

ATOM 160 H51 KIH 1 16.760 26.540 25.010 1.00 0.00

ATOM 161 H52 KIH 1 15.090 26.390 23.240 1.00 0.00

ATOM 162 H53 KIH 1 14.390 25.680 21.150 1.00 0.00

ATOM 163 H54 KIH 1 14.910 24.820 18.900 1.00 0.00

ATOM 164 H55 KIH 1 16.470 23.850 17.350 1.00 0.00

ATOM 165 H56 KIH 1 18.610 23.050 16.840 1.00 0.00

ATOM 166 O7 KIH 1 21.320 21.940 20.150 1.00 0.00

ATOM 167 O8 KIH 1 22.620 21.570 22.950 1.00 0.00

ATOM 168 C98 KIH 1 20.010 21.410 23.590 1.00 0.00

ATOM 169 F4 KIH 1 20.120 22.310 24.570 1.00 0.00

ATOM 170 F5 KIH 1 19.560 22.140 22.560 1.00 0.00

ATOM 171 F6 KIH 1 19.020 20.610 24.020 1.00 0.00

ATOM 172 N2 KIH 1 21.230 19.870 21.710 1.00 0.00

ATOM 173 P2 KIH 1 21.200 20.360 20.140 1.00 0.00

ATOM 174 S2 KIH 1 21.550 20.620 23.150 1.00 0.00

ATOM 175 N3 KIH 1 19.910 19.940 19.510 1.00 0.00

ATOM 176 H57 KIH 1 20.810 18.960 21.880 1.00 0.00

TER

ENDMDL

REMARK GENERATED BY TRJCONV

TITLE KIH t= 3500.00000

REMARK THIS IS A SIMULATION BOX

CRYST1 38.616 38.616 38.616 90.00 90.00 90.00 P 1 1

MODEL 8

ATOM 1 C1 KIH 1 16.180 19.620 17.060 1.00 0.00

ATOM 2 C2 KIH 1 16.290 19.950 18.400 1.00 0.00

ATOM 3 C3 KIH 1 15.220 19.860 19.260 1.00 0.00

ATOM 4 C4 KIH 1 14.010 19.360 18.830 1.00 0.00

ATOM 5 C5 KIH 1 13.920 18.950 17.520 1.00 0.00

ATOM 6 C6 KIH 1 14.980 19.070 16.650 1.00 0.00

ATOM 7 C7 KIH 1 12.710 18.510 17.050 1.00 0.00

ATOM 8 C8 KIH 1 12.570 18.120 15.740 1.00 0.00

ATOM 9 C9 KIH 1 13.580 18.300 14.840 1.00 0.00

ATOM 10 C10 KIH 1 14.780 18.760 15.330 1.00 0.00

ATOM 11 C11 KIH 1 15.460 20.370 20.630 1.00 0.00

ATOM 12 C12 KIH 1 15.340 19.530 21.710 1.00 0.00

ATOM 13 C13 KIH 1 15.700 19.910 22.990 1.00 0.00

ATOM 14 C14 KIH 1 15.590 19.020 24.040 1.00 0.00

ATOM 15 C15 KIH 1 15.280 17.700 23.890 1.00 0.00

ATOM 16 C16 KIH 1 15.260 16.860 24.970 1.00 0.00

ATOM 17 C17 KIH 1 15.660 17.300 26.210 1.00 0.00

ATOM 18 C18 KIH 1 15.950 18.640 26.350 1.00 0.00

ATOM 19 C19 KIH 1 15.860 19.520 25.300 1.00 0.00

ATOM 20 C20 KIH 1 16.130 20.860 25.500 1.00 0.00

ATOM 21 C21 KIH 1 16.140 21.760 24.470 1.00 0.00

ATOM 22 C22 KIH 1 15.910 21.250 23.210 1.00 0.00

ATOM 23 C23 KIH 1 15.890 22.190 22.210 1.00 0.00

ATOM 24 C24 KIH 1 15.690 21.700 20.940 1.00 0.00

ATOM 25 H1 KIH 1 13.200 19.320 19.550 1.00 0.00

ATOM 26 H2 KIH 1 11.870 18.430 17.730 1.00 0.00

ATOM 27 H3 KIH 1 11.610 17.690 15.460 1.00 0.00

ATOM 28 H4 KIH 1 13.500 18.180 13.760 1.00 0.00

ATOM 29 H5 KIH 1 15.620 18.960 14.680 1.00 0.00

ATOM 30 H6 KIH 1 15.010 18.550 21.380 1.00 0.00

ATOM 31 H7 KIH 1 14.960 17.330 22.910 1.00 0.00

ATOM 32 H8 KIH 1 15.130 15.830 24.630 1.00 0.00

ATOM 33 H9 KIH 1 15.630 16.630 27.050 1.00 0.00

ATOM 34 H10 KIH 1 16.230 19.030 27.320 1.00 0.00

ATOM 35 H11 KIH 1 16.190 21.210 26.520 1.00 0.00

ATOM 36 H12 KIH 1 16.370 22.810 24.580 1.00 0.00

ATOM 37 H13 KIH 1 15.910 23.260 22.360 1.00 0.00

ATOM 38 H14 KIH 1 15.870 22.280 20.060 1.00 0.00

ATOM 39 O1 KIH 1 17.520 20.260 18.940 1.00 0.00

ATOM 40 O2 KIH 1 19.210 18.560 22.640 1.00 0.00

ATOM 41 C25 KIH 1 17.370 19.940 16.230 1.00 0.00

ATOM 42 C26 KIH 1 18.560 19.320 16.530 1.00 0.00

ATOM 43 C27 KIH 1 19.670 19.680 15.790 1.00 0.00

ATOM 44 C28 KIH 1 19.650 20.640 14.810 1.00 0.00

ATOM 45 C29 KIH 1 18.450 21.260 14.570 1.00 0.00

ATOM 46 C30 KIH 1 17.330 20.970 15.310 1.00 0.00

ATOM 47 C31 KIH 1 18.420 22.230 13.590 1.00 0.00

ATOM 48 C32 KIH 1 17.280 22.990 13.440 1.00 0.00

ATOM 49 C33 KIH 1 16.150 22.750 14.170 1.00 0.00

ATOM 50 C34 KIH 1 16.260 21.810 15.170 1.00 0.00

ATOM 51 C35 KIH 1 20.950 18.920 15.840 1.00 0.00

ATOM 52 C36 KIH 1 21.150 17.640 15.350 1.00 0.00

ATOM 53 C37 KIH 1 22.370 17.010 15.310 1.00 0.00

ATOM 54 C38 KIH 1 22.530 15.730 14.840 1.00 0.00

ATOM 55 C39 KIH 1 21.450 14.950 14.490 1.00 0.00

ATOM 56 C40 KIH 1 21.630 13.650 14.070 1.00 0.00

ATOM 57 C41 KIH 1 22.860 13.020 14.130 1.00 0.00

ATOM 58 C42 KIH 1 23.940 13.780 14.510 1.00 0.00

ATOM 59 C43 KIH 1 23.770 15.120 14.800 1.00 0.00

ATOM 60 C44 KIH 1 24.850 15.850 15.230 1.00 0.00

ATOM 61 C45 KIH 1 24.740 17.160 15.630 1.00 0.00

ATOM 62 C46 KIH 1 23.470 17.710 15.740 1.00 0.00

ATOM 63 C47 KIH 1 23.360 18.990 16.220 1.00 0.00

ATOM 64 C48 KIH 1 22.090 19.520 16.300 1.00 0.00

ATOM 65 H15 KIH 1 20.540 20.930 14.260 1.00 0.00

ATOM 66 H16 KIH 1 19.230 22.440 12.910 1.00 0.00

ATOM 67 H17 KIH 1 17.230 23.830 12.740 1.00 0.00

ATOM 68 H18 KIH 1 15.320 23.440 14.120 1.00 0.00

ATOM 69 H19 KIH 1 15.320 21.620 15.680 1.00 0.00

ATOM 70 H20 KIH 1 20.240 17.210 14.980 1.00 0.00

ATOM 71 H21 KIH 1 20.430 15.280 14.610 1.00 0.00

ATOM 72 H22 KIH 1 20.740 13.090 13.870 1.00 0.00

ATOM 73 H23 KIH 1 22.990 11.960 13.960 1.00 0.00

ATOM 74 H24 KIH 1 24.910 13.310 14.580 1.00 0.00

ATOM 75 H25 KIH 1 25.860 15.480 15.270 1.00 0.00

ATOM 76 H26 KIH 1 25.580 17.670 16.070 1.00 0.00

ATOM 77 H27 KIH 1 24.220 19.510 16.630 1.00 0.00

ATOM 78 H28 KIH 1 21.900 20.520 16.650 1.00 0.00

ATOM 79 O3 KIH 1 18.720 18.540 17.660 1.00 0.00

ATOM 80 O4 KIH 1 17.540 16.740 22.590 1.00 0.00

ATOM 81 C49 KIH 1 19.800 16.270 21.490 1.00 0.00

ATOM 82 F1 KIH 1 19.420 15.350 20.580 1.00 0.00

ATOM 83 F2 KIH 1 20.770 16.940 20.860 1.00 0.00

ATOM 84 F3 KIH 1 20.340 15.680 22.550 1.00 0.00

ATOM 85 N1 KIH 1 18.020 18.110 20.410 1.00 0.00

ATOM 86 P1 KIH 1 18.650 19.150 19.100 1.00 0.00

ATOM 87 S1 KIH 1 18.570 17.490 21.910 1.00 0.00

ATOM 88 C50 KIH 1 23.670 21.990 19.490 1.00 0.00

ATOM 89 C51 KIH 1 23.590 20.680 19.900 1.00 0.00

ATOM 90 C52 KIH 1 24.570 20.120 20.670 1.00 0.00

ATOM 91 C53 KIH 1 25.490 20.960 21.270 1.00 0.00

ATOM 92 C54 KIH 1 25.600 22.270 20.880 1.00 0.00

ATOM 93 C55 KIH 1 24.670 22.790 20.000 1.00 0.00

ATOM 94 C56 KIH 1 26.570 23.060 21.440 1.00 0.00

ATOM 95 C57 KIH 1 26.590 24.420 21.220 1.00 0.00

ATOM 96 C58 KIH 1 25.640 24.920 20.370 1.00 0.00

ATOM 97 C59 KIH 1 24.660 24.150 19.780 1.00 0.00

ATOM 98 C60 KIH 1 24.550 18.640 20.850 1.00 0.00

ATOM 99 C61 KIH 1 24.680 18.060 22.090 1.00 0.00

ATOM 100 C62 KIH 1 24.710 16.700 22.250 1.00 0.00

ATOM 101 C63 KIH 1 24.630 16.090 23.480 1.00 0.00

ATOM 102 C64 KIH 1 24.490 16.750 24.690 1.00 0.00

ATOM 103 C65 KIH 1 24.480 16.090 25.890 1.00 0.00

ATOM 104 C66 KIH 1 24.660 14.730 25.980 1.00 0.00

ATOM 105 C67 KIH 1 24.900 14.070 24.790 1.00 0.00

ATOM 106 C68 KIH 1 24.860 14.730 23.590 1.00 0.00

ATOM 107 C69 KIH 1 24.970 13.990 22.440 1.00 0.00

ATOM 108 C70 KIH 1 24.810 14.540 21.190 1.00 0.00

ATOM 109 C71 KIH 1 24.660 15.910 21.120 1.00 0.00

ATOM 110 C72 KIH 1 24.650 16.440 19.860 1.00 0.00

ATOM 111 C73 KIH 1 24.520 17.810 19.750 1.00 0.00

ATOM 112 H29 KIH 1 26.320 20.470 21.730 1.00 0.00

ATOM 113 H30 KIH 1 27.260 22.660 22.160 1.00 0.00

ATOM 114 H31 KIH 1 27.500 24.910 21.560 1.00 0.00

ATOM 115 H32 KIH 1 25.630 25.990 20.230 1.00 0.00

ATOM 116 H33 KIH 1 23.980 24.610 19.080 1.00 0.00

ATOM 117 H34 KIH 1 24.570 18.730 22.930 1.00 0.00

ATOM 118 H35 KIH 1 24.320 17.810 24.800 1.00 0.00

ATOM 119 H36 KIH 1 24.150 16.700 26.710 1.00 0.00

ATOM 120 H37 KIH 1 24.770 14.190 26.910 1.00 0.00

ATOM 121 H38 KIH 1 25.220 13.040 24.860 1.00 0.00

ATOM 122 H39 KIH 1 25.150 12.920 22.480 1.00 0.00

ATOM 123 H40 KIH 1 24.930 13.920 20.320 1.00 0.00

ATOM 124 H41 KIH 1 24.640 15.850 18.960 1.00 0.00

ATOM 125 H42 KIH 1 24.490 18.210 18.760 1.00 0.00

ATOM 126 O5 KIH 1 22.450 19.960 19.640 1.00 0.00

ATOM 127 O6 KIH 1 22.030 19.140 23.820 1.00 0.00

ATOM 128 C74 KIH 1 22.730 22.540 18.460 1.00 0.00

ATOM 129 C75 KIH 1 21.420 22.740 18.870 1.00 0.00

ATOM 130 C76 KIH 1 20.580 23.580 18.180 1.00 0.00

ATOM 131 C77 KIH 1 20.940 23.970 16.910 1.00 0.00

ATOM 132 C78 KIH 1 22.130 23.520 16.390 1.00 0.00

ATOM 133 C79 KIH 1 23.070 22.920 17.190 1.00 0.00

ATOM 134 C80 KIH 1 22.350 23.720 15.040 1.00 0.00

ATOM 135 C81 KIH 1 23.590 23.400 14.520 1.00 0.00

ATOM 136 C82 KIH 1 24.600 22.940 15.340 1.00 0.00

ATOM 137 C83 KIH 1 24.340 22.740 16.680 1.00 0.00

ATOM 138 C84 KIH 1 19.220 23.900 18.690 1.00 0.00

ATOM 139 C85 KIH 1 19.110 24.730 19.780 1.00 0.00

ATOM 140 C86 KIH 1 17.900 25.190 20.220 1.00 0.00

ATOM 141 C87 KIH 1 17.710 26.000 21.310 1.00 0.00

ATOM 142 C88 KIH 1 18.740 26.410 22.110 1.00 0.00

ATOM 143 C89 KIH 1 18.540 27.200 23.220 1.00 0.00

ATOM 144 C90 KIH 1 17.250 27.530 23.580 1.00 0.00

ATOM 145 C91 KIH 1 16.170 27.160 22.810 1.00 0.00

ATOM 146 C92 KIH 1 16.440 26.360 21.720 1.00 0.00

ATOM 147 C93 KIH 1 15.330 26.050 20.980 1.00 0.00

ATOM 148 C94 KIH 1 15.520 25.300 19.840 1.00 0.00

ATOM 149 C95 KIH 1 16.770 24.840 19.500 1.00 0.00

ATOM 150 C96 KIH 1 16.870 24.060 18.360 1.00 0.00

ATOM 151 C97 KIH 1 18.090 23.520 18.010 1.00 0.00

ATOM 152 H43 KIH 1 20.200 24.460 16.290 1.00 0.00

ATOM 153 H44 KIH 1 21.630 24.180 14.390 1.00 0.00

ATOM 154 H45 KIH 1 23.920 23.600 13.520 1.00 0.00

ATOM 155 H46 KIH 1 25.590 22.800 14.940 1.00 0.00

ATOM 156 H47 KIH 1 25.170 22.300 17.220 1.00 0.00

ATOM 157 H48 KIH 1 20.030 24.890 20.340 1.00 0.00

ATOM 158 H49 KIH 1 19.770 26.080 22.000 1.00 0.00

ATOM 159 H50 KIH 1 19.410 27.350 23.850 1.00 0.00

ATOM 160 H51 KIH 1 16.990 27.910 24.550 1.00 0.00

ATOM 161 H52 KIH 1 15.150 27.370 23.100 1.00 0.00

ATOM 162 H53 KIH 1 14.310 26.300 21.220 1.00 0.00

ATOM 163 H54 KIH 1 14.610 25.010 19.350 1.00 0.00

ATOM 164 H55 KIH 1 15.970 23.810 17.830 1.00 0.00

ATOM 165 H56 KIH 1 18.020 22.810 17.190 1.00 0.00

ATOM 166 O7 KIH 1 20.990 22.130 20.040 1.00 0.00

ATOM 167 O8 KIH 1 22.790 21.340 23.050 1.00 0.00

ATOM 168 C98 KIH 1 20.550 21.230 24.300 1.00 0.00

ATOM 169 F4 KIH 1 21.120 21.670 25.440 1.00 0.00

ATOM 170 F5 KIH 1 20.150 22.300 23.600 1.00 0.00

ATOM 171 F6 KIH 1 19.490 20.500 24.660 1.00 0.00

ATOM 172 N2 KIH 1 21.030 20.090 21.730 1.00 0.00

ATOM 173 P2 KIH 1 21.070 20.560 20.150 1.00 0.00

ATOM 174 S2 KIH 1 21.710 20.410 23.210 1.00 0.00

ATOM 175 N3 KIH 1 19.880 19.920 19.500 1.00 0.00

ATOM 176 H57 KIH 1 20.570 19.180 21.740 1.00 0.00

TER

ENDMDL

REMARK GENERATED BY TRJCONV

TITLE KIH t= 4000.00000

REMARK THIS IS A SIMULATION BOX

CRYST1 38.341 38.341 38.341 90.00 90.00 90.00 P 1 1

MODEL 9

ATOM 1 C1 KIH 1 16.300 20.050 17.050 1.00 0.00

ATOM 2 C2 KIH 1 16.400 20.400 18.380 1.00 0.00

ATOM 3 C3 KIH 1 15.260 20.570 19.120 1.00 0.00

ATOM 4 C4 KIH 1 14.010 20.340 18.580 1.00 0.00

ATOM 5 C5 KIH 1 13.960 19.780 17.320 1.00 0.00

ATOM 6 C6 KIH 1 15.080 19.770 16.500 1.00 0.00

ATOM 7 C7 KIH 1 12.750 19.340 16.830 1.00 0.00

ATOM 8 C8 KIH 1 12.680 18.770 15.590 1.00 0.00

ATOM 9 C9 KIH 1 13.790 18.690 14.770 1.00 0.00

ATOM 10 C10 KIH 1 14.960 19.230 15.260 1.00 0.00

ATOM 11 C11 KIH 1 15.280 20.860 20.580 1.00 0.00

ATOM 12 C12 KIH 1 15.140 19.880 21.540 1.00 0.00

ATOM 13 C13 KIH 1 15.220 20.040 22.900 1.00 0.00

ATOM 14 C14 KIH 1 14.950 19.060 23.840 1.00 0.00

ATOM 15 C15 KIH 1 14.660 17.760 23.500 1.00 0.00

ATOM 16 C16 KIH 1 14.360 16.800 24.440 1.00 0.00

ATOM 17 C17 KIH 1 14.430 17.070 25.780 1.00 0.00

ATOM 18 C18 KIH 1 14.670 18.390 26.110 1.00 0.00

ATOM 19 C19 KIH 1 14.920 19.370 25.180 1.00 0.00

ATOM 20 C20 KIH 1 15.110 20.670 25.590 1.00 0.00

ATOM 21 C21 KIH 1 15.590 21.550 24.660 1.00 0.00

ATOM 22 C22 KIH 1 15.570 21.320 23.310 1.00 0.00

ATOM 23 C23 KIH 1 15.810 22.340 22.430 1.00 0.00

ATOM 24 C24 KIH 1 15.660 22.080 21.090 1.00 0.00

ATOM 25 H1 KIH 1 13.140 20.320 19.210 1.00 0.00

ATOM 26 H2 KIH 1 11.790 19.350 17.330 1.00 0.00

ATOM 27 H3 KIH 1 11.740 18.560 15.110 1.00 0.00

ATOM 28 H4 KIH 1 13.660 18.290 13.780 1.00 0.00

ATOM 29 H5 KIH 1 15.820 19.250 14.600 1.00 0.00

ATOM 30 H6 KIH 1 14.810 18.910 21.200 1.00 0.00

ATOM 31 H7 KIH 1 14.740 17.400 22.490 1.00 0.00

ATOM 32 H8 KIH 1 14.250 15.760 24.140 1.00 0.00

ATOM 33 H9 KIH 1 14.270 16.310 26.540 1.00 0.00

ATOM 34 H10 KIH 1 14.640 18.690 27.150 1.00 0.00

ATOM 35 H11 KIH 1 14.910 20.930 26.620 1.00 0.00

ATOM 36 H12 KIH 1 15.870 22.530 25.020 1.00 0.00

ATOM 37 H13 KIH 1 16.010 23.350 22.740 1.00 0.00

ATOM 38 H14 KIH 1 15.860 22.910 20.420 1.00 0.00

ATOM 39 O1 KIH 1 17.600 20.490 19.060 1.00 0.00

ATOM 40 O2 KIH 1 19.440 18.160 22.740 1.00 0.00

ATOM 41 C25 KIH 1 17.560 20.200 16.250 1.00 0.00

ATOM 42 C26 KIH 1 18.680 19.470 16.590 1.00 0.00

ATOM 43 C27 KIH 1 19.780 19.430 15.760 1.00 0.00

ATOM 44 C28 KIH 1 19.870 20.250 14.660 1.00 0.00

ATOM 45 C29 KIH 1 18.790 21.060 14.410 1.00 0.00

ATOM 46 C30 KIH 1 17.670 21.090 15.200 1.00 0.00

ATOM 47 C31 KIH 1 18.920 21.900 13.320 1.00 0.00

ATOM 48 C32 KIH 1 17.960 22.820 12.990 1.00 0.00

ATOM 49 C33 KIH 1 16.860 22.910 13.810 1.00 0.00

ATOM 50 C34 KIH 1 16.740 22.050 14.870 1.00 0.00

ATOM 51 C35 KIH 1 20.930 18.560 16.120 1.00 0.00

ATOM 52 C36 KIH 1 20.830 17.220 16.410 1.00 0.00

ATOM 53 C37 KIH 1 21.930 16.410 16.550 1.00 0.00

ATOM 54 C38 KIH 1 21.870 15.100 16.970 1.00 0.00

ATOM 55 C39 KIH 1 20.680 14.520 17.320 1.00 0.00

ATOM 56 C40 KIH 1 20.690 13.230 17.780 1.00 0.00

ATOM 57 C41 KIH 1 21.840 12.490 18.000 1.00 0.00

ATOM 58 C42 KIH 1 23.010 13.120 17.630 1.00 0.00

ATOM 59 C43 KIH 1 23.040 14.420 17.170 1.00 0.00

ATOM 60 C44 KIH 1 24.270 14.940 16.900 1.00 0.00

ATOM 61 C45 KIH 1 24.320 16.300 16.630 1.00 0.00

ATOM 62 C46 KIH 1 23.160 17.030 16.500 1.00 0.00

ATOM 63 C47 KIH 1 23.350 18.370 16.300 1.00 0.00

ATOM 64 C48 KIH 1 22.190 19.110 16.130 1.00 0.00

ATOM 65 H15 KIH 1 20.760 20.250 14.050 1.00 0.00

ATOM 66 H16 KIH 1 19.770 21.870 12.660 1.00 0.00

ATOM 67 H17 KIH 1 18.080 23.440 12.110 1.00 0.00

ATOM 68 H18 KIH 1 16.080 23.620 13.620 1.00 0.00

ATOM 69 H19 KIH 1 15.790 22.090 15.370 1.00 0.00

ATOM 70 H20 KIH 1 19.820 16.820 16.440 1.00 0.00

ATOM 71 H21 KIH 1 19.770 15.100 17.260 1.00 0.00

ATOM 72 H22 KIH 1 19.790 12.770 18.140 1.00 0.00

ATOM 73 H23 KIH 1 21.960 11.500 18.400 1.00 0.00

ATOM 74 H24 KIH 1 23.920 12.550 17.620 1.00 0.00

ATOM 75 H25 KIH 1 25.180 14.370 17.040 1.00 0.00

ATOM 76 H26 KIH 1 25.320 16.680 16.520 1.00 0.00

ATOM 77 H27 KIH 1 24.310 18.870 16.230 1.00 0.00

ATOM 78 H28 KIH 1 22.340 20.150 15.860 1.00 0.00

ATOM 79 O3 KIH 1 18.690 18.650 17.700 1.00 0.00

ATOM 80 O4 KIH 1 17.550 16.590 22.440 1.00 0.00

ATOM 81 C49 KIH 1 19.760 16.170 21.270 1.00 0.00

ATOM 82 F1 KIH 1 20.650 16.680 20.400 1.00 0.00

ATOM 83 F2 KIH 1 20.440 15.540 22.230 1.00 0.00

ATOM 84 F3 KIH 1 19.100 15.280 20.520 1.00 0.00

ATOM 85 N1 KIH 1 18.030 18.220 20.500 1.00 0.00

ATOM 86 P1 KIH 1 18.590 19.260 19.150 1.00 0.00

ATOM 87 S1 KIH 1 18.600 17.390 21.870 1.00 0.00

ATOM 88 C50 KIH 1 23.970 21.550 19.010 1.00 0.00

ATOM 89 C51 KIH 1 23.630 20.370 19.630 1.00 0.00

ATOM 90 C52 KIH 1 24.550 19.740 20.450 1.00 0.00

ATOM 91 C53 KIH 1 25.800 20.290 20.680 1.00 0.00

ATOM 92 C54 KIH 1 26.050 21.510 20.130 1.00 0.00

ATOM 93 C55 KIH 1 25.170 22.170 19.290 1.00 0.00

ATOM 94 C56 KIH 1 27.280 22.080 20.390 1.00 0.00

ATOM 95 C57 KIH 1 27.590 23.320 19.890 1.00 0.00

ATOM 96 C58 KIH 1 26.710 23.960 19.060 1.00 0.00

ATOM 97 C59 KIH 1 25.480 23.410 18.800 1.00 0.00

ATOM 98 C60 KIH 1 24.150 18.440 21.050 1.00 0.00

ATOM 99 C61 KIH 1 24.130 18.150 22.390 1.00 0.00

ATOM 100 C62 KIH 1 23.720 16.930 22.890 1.00 0.00

ATOM 101 C63 KIH 1 23.570 16.690 24.240 1.00 0.00

ATOM 102 C64 KIH 1 23.650 17.640 25.230 1.00 0.00

ATOM 103 C65 KIH 1 23.430 17.330 26.550 1.00 0.00

ATOM 104 C66 KIH 1 23.260 16.030 26.960 1.00 0.00

ATOM 105 C67 KIH 1 23.250 15.050 25.990 1.00 0.00

ATOM 106 C68 KIH 1 23.290 15.410 24.670 1.00 0.00

ATOM 107 C69 KIH 1 23.150 14.370 23.770 1.00 0.00

ATOM 108 C70 KIH 1 23.190 14.720 22.440 1.00 0.00

ATOM 109 C71 KIH 1 23.410 15.980 21.960 1.00 0.00

ATOM 110 C72 KIH 1 23.450 16.210 20.600 1.00 0.00

ATOM 111 C73 KIH 1 23.870 17.440 20.150 1.00 0.00

ATOM 112 H29 KIH 1 26.440 19.720 21.350 1.00 0.00

ATOM 113 H30 KIH 1 28.050 21.590 20.970 1.00 0.00

ATOM 114 H31 KIH 1 28.550 23.630 20.280 1.00 0.00

ATOM 115 H32 KIH 1 26.950 24.960 18.710 1.00 0.00

ATOM 116 H33 KIH 1 24.780 24.040 18.260 1.00 0.00

ATOM 117 H34 KIH 1 24.390 18.930 23.100 1.00 0.00

ATOM 118 H35 KIH 1 23.880 18.650 24.950 1.00 0.00

ATOM 119 H36 KIH 1 23.630 18.090 27.290 1.00 0.00

ATOM 120 H37 KIH 1 23.230 15.870 28.030 1.00 0.00

ATOM 121 H38 KIH 1 23.320 14.020 26.300 1.00 0.00

ATOM 122 H39 KIH 1 22.910 13.350 24.030 1.00 0.00

ATOM 123 H40 KIH 1 23.020 13.890 21.760 1.00 0.00

ATOM 124 H41 KIH 1 23.180 15.420 19.900 1.00 0.00

ATOM 125 H42 KIH 1 23.890 17.740 19.130 1.00 0.00

ATOM 126 O5 KIH 1 22.400 19.790 19.380 1.00 0.00

ATOM 127 O6 KIH 1 21.530 19.410 24.120 1.00 0.00

ATOM 128 C74 KIH 1 22.950 22.220 18.160 1.00 0.00

ATOM 129 C75 KIH 1 21.710 22.440 18.720 1.00 0.00

ATOM 130 C76 KIH 1 20.760 23.120 17.980 1.00 0.00

ATOM 131 C77 KIH 1 20.990 23.450 16.670 1.00 0.00

ATOM 132 C78 KIH 1 22.140 23.010 16.050 1.00 0.00

ATOM 133 C79 KIH 1 23.160 22.480 16.820 1.00 0.00

ATOM 134 C80 KIH 1 22.300 23.260 14.710 1.00 0.00

ATOM 135 C81 KIH 1 23.460 22.890 14.060 1.00 0.00

ATOM 136 C82 KIH 1 24.440 22.200 14.750 1.00 0.00

ATOM 137 C83 KIH 1 24.260 22.100 16.100 1.00 0.00

ATOM 138 C84 KIH 1 19.490 23.620 18.550 1.00 0.00

ATOM 139 C85 KIH 1 19.530 24.420 19.660 1.00 0.00

ATOM 140 C86 KIH 1 18.500 25.040 20.340 1.00 0.00

ATOM 141 C87 KIH 1 18.600 25.780 21.480 1.00 0.00

ATOM 142 C88 KIH 1 19.690 25.720 22.310 1.00 0.00

ATOM 143 C89 KIH 1 19.720 26.450 23.480 1.00 0.00

ATOM 144 C90 KIH 1 18.630 27.200 23.860 1.00 0.00

ATOM 145 C91 KIH 1 17.580 27.350 22.990 1.00 0.00

ATOM 146 C92 KIH 1 17.540 26.580 21.850 1.00 0.00

ATOM 147 C93 KIH 1 16.350 26.570 21.150 1.00 0.00

ATOM 148 C94 KIH 1 16.230 25.670 20.120 1.00 0.00

ATOM 149 C95 KIH 1 17.310 24.970 19.650 1.00 0.00

ATOM 150 C96 KIH 1 17.170 24.140 18.550 1.00 0.00

ATOM 151 C97 KIH 1 18.240 23.500 17.980 1.00 0.00

ATOM 152 H43 KIH 1 20.210 24.010 16.180 1.00 0.00

ATOM 153 H44 KIH 1 21.590 23.810 14.100 1.00 0.00

ATOM 154 H45 KIH 1 23.550 23.080 13.000 1.00 0.00

ATOM 155 H46 KIH 1 25.370 21.930 14.260 1.00 0.00

ATOM 156 H47 KIH 1 25.150 21.780 16.620 1.00 0.00

ATOM 157 H48 KIH 1 20.560 24.570 19.990 1.00 0.00

ATOM 158 H49 KIH 1 20.480 25.020 22.110 1.00 0.00

ATOM 159 H50 KIH 1 20.620 26.340 24.080 1.00 0.00

ATOM 160 H51 KIH 1 18.690 27.730 24.810 1.00 0.00

ATOM 161 H52 KIH 1 16.640 27.840 23.210 1.00 0.00

ATOM 162 H53 KIH 1 15.500 27.190 21.420 1.00 0.00

ATOM 163 H54 KIH 1 15.280 25.600 19.600 1.00 0.00

ATOM 164 H55 KIH 1 16.210 24.070 18.070 1.00 0.00

ATOM 165 H56 KIH 1 18.010 22.850 17.130 1.00 0.00

ATOM 166 O7 KIH 1 21.360 21.990 19.980 1.00 0.00

ATOM 167 O8 KIH 1 22.770 21.340 23.070 1.00 0.00

ATOM 168 C98 KIH 1 20.200 21.530 23.690 1.00 0.00

ATOM 169 F4 KIH 1 19.970 21.660 25.000 1.00 0.00

ATOM 170 F5 KIH 1 20.410 22.760 23.220 1.00 0.00

ATOM 171 F6 KIH 1 19.050 21.170 23.110 1.00 0.00

ATOM 172 N2 KIH 1 21.150 19.980 21.710 1.00 0.00

ATOM 173 P2 KIH 1 21.160 20.430 20.120 1.00 0.00

ATOM 174 S2 KIH 1 21.610 20.520 23.200 1.00 0.00

ATOM 175 N3 KIH 1 19.870 19.930 19.520 1.00 0.00

ATOM 176 H57 KIH 1 20.600 19.150 21.860 1.00 0.00

TER

ENDMDL

REMARK GENERATED BY TRJCONV

TITLE KIH t= 4500.00000

REMARK THIS IS A SIMULATION BOX

CRYST1 38.353 38.353 38.353 90.00 90.00 90.00 P 1 1

MODEL 10

ATOM 1 C1 KIH 1 16.290 20.280 17.100 1.00 0.00

ATOM 2 C2 KIH 1 16.400 20.340 18.470 1.00 0.00

ATOM 3 C3 KIH 1 15.290 20.230 19.270 1.00 0.00

ATOM 4 C4 KIH 1 14.070 20.060 18.680 1.00 0.00

ATOM 5 C5 KIH 1 13.920 20.090 17.310 1.00 0.00

ATOM 6 C6 KIH 1 15.050 20.080 16.520 1.00 0.00

ATOM 7 C7 KIH 1 12.670 20.110 16.740 1.00 0.00

ATOM 8 C8 KIH 1 12.510 19.950 15.380 1.00 0.00

ATOM 9 C9 KIH 1 13.660 20.000 14.620 1.00 0.00

ATOM 10 C10 KIH 1 14.920 20.070 15.150 1.00 0.00

ATOM 11 C11 KIH 1 15.410 20.320 20.760 1.00 0.00

ATOM 12 C12 KIH 1 15.250 19.180 21.510 1.00 0.00

ATOM 13 C13 KIH 1 15.510 19.200 22.870 1.00 0.00

ATOM 14 C14 KIH 1 15.300 18.140 23.710 1.00 0.00

ATOM 15 C15 KIH 1 14.720 16.950 23.310 1.00 0.00

ATOM 16 C16 KIH 1 14.400 15.970 24.220 1.00 0.00

ATOM 17 C17 KIH 1 14.770 16.100 25.530 1.00 0.00

ATOM 18 C18 KIH 1 15.370 17.270 25.960 1.00 0.00

ATOM 19 C19 KIH 1 15.620 18.260 25.040 1.00 0.00

ATOM 20 C20 KIH 1 16.110 19.430 25.570 1.00 0.00

ATOM 21 C21 KIH 1 16.290 20.490 24.720 1.00 0.00

ATOM 22 C22 KIH 1 16.000 20.380 23.380 1.00 0.00

ATOM 23 C23 KIH 1 16.090 21.550 22.650 1.00 0.00

ATOM 24 C24 KIH 1 15.830 21.500 21.310 1.00 0.00

ATOM 25 H1 KIH 1 13.190 19.970 19.310 1.00 0.00

ATOM 26 H2 KIH 1 11.830 20.090 17.420 1.00 0.00

ATOM 27 H3 KIH 1 11.510 19.980 14.990 1.00 0.00

ATOM 28 H4 KIH 1 13.530 19.920 13.560 1.00 0.00

ATOM 29 H5 KIH 1 15.720 20.130 14.420 1.00 0.00

ATOM 30 H6 KIH 1 14.950 18.310 20.960 1.00 0.00

ATOM 31 H7 KIH 1 14.330 16.760 22.320 1.00 0.00

ATOM 32 H8 KIH 1 14.010 15.050 23.810 1.00 0.00

ATOM 33 H9 KIH 1 14.610 15.310 26.240 1.00 0.00

ATOM 34 H10 KIH 1 15.620 17.350 27.010 1.00 0.00

ATOM 35 H11 KIH 1 16.280 19.580 26.630 1.00 0.00

ATOM 36 H12 KIH 1 16.690 21.440 25.040 1.00 0.00

ATOM 37 H13 KIH 1 16.450 22.500 23.000 1.00 0.00

ATOM 38 H14 KIH 1 15.910 22.420 20.730 1.00 0.00

ATOM 39 O1 KIH 1 17.650 20.450 19.040 1.00 0.00

ATOM 40 O2 KIH 1 19.100 18.550 22.830 1.00 0.00

ATOM 41 C25 KIH 1 17.550 20.510 16.330 1.00 0.00

ATOM 42 C26 KIH 1 18.600 19.660 16.560 1.00 0.00

ATOM 43 C27 KIH 1 19.760 19.820 15.840 1.00 0.00

ATOM 44 C28 KIH 1 19.850 20.900 14.980 1.00 0.00

ATOM 45 C29 KIH 1 18.890 21.880 14.910 1.00 0.00

ATOM 46 C30 KIH 1 17.690 21.620 15.540 1.00 0.00

ATOM 47 C31 KIH 1 19.230 23.050 14.270 1.00 0.00

ATOM 48 C32 KIH 1 18.270 24.040 14.160 1.00 0.00

ATOM 49 C33 KIH 1 17.010 23.730 14.610 1.00 0.00

ATOM 50 C34 KIH 1 16.730 22.570 15.310 1.00 0.00

ATOM 51 C35 KIH 1 20.900 18.860 15.850 1.00 0.00

ATOM 52 C36 KIH 1 20.830 17.650 15.220 1.00 0.00

ATOM 53 C37 KIH 1 21.930 16.860 14.970 1.00 0.00

ATOM 54 C38 KIH 1 21.800 15.700 14.250 1.00 0.00

ATOM 55 C39 KIH 1 20.650 15.340 13.570 1.00 0.00

ATOM 56 C40 KIH 1 20.580 14.090 13.010 1.00 0.00

ATOM 57 C41 KIH 1 21.680 13.260 13.020 1.00 0.00

ATOM 58 C42 KIH 1 22.890 13.710 13.480 1.00 0.00

ATOM 59 C43 KIH 1 22.890 14.860 14.230 1.00 0.00

ATOM 60 C44 KIH 1 24.030 15.200 14.920 1.00 0.00

ATOM 61 C45 KIH 1 24.170 16.380 15.620 1.00 0.00

ATOM 62 C46 KIH 1 23.090 17.250 15.590 1.00 0.00

ATOM 63 C47 KIH 1 23.220 18.450 16.230 1.00 0.00

ATOM 64 C48 KIH 1 22.120 19.260 16.360 1.00 0.00

ATOM 65 H15 KIH 1 20.780 21.060 14.460 1.00 0.00

ATOM 66 H16 KIH 1 20.240 23.270 13.950 1.00 0.00

ATOM 67 H17 KIH 1 18.470 25.020 13.760 1.00 0.00

ATOM 68 H18 KIH 1 16.200 24.440 14.620 1.00 0.00

ATOM 69 H19 KIH 1 15.740 22.450 15.720 1.00 0.00

ATOM 70 H20 KIH 1 19.850 17.490 14.800 1.00 0.00

ATOM 71 H21 KIH 1 19.800 15.990 13.450 1.00 0.00

ATOM 72 H22 KIH 1 19.630 13.850 12.560 1.00 0.00

ATOM 73 H23 KIH 1 21.720 12.250 12.640 1.00 0.00

ATOM 74 H24 KIH 1 23.720 13.010 13.470 1.00 0.00

ATOM 75 H25 KIH 1 24.890 14.540 14.910 1.00 0.00

ATOM 76 H26 KIH 1 25.140 16.600 16.030 1.00 0.00

ATOM 77 H27 KIH 1 24.210 18.770 16.540 1.00 0.00

ATOM 78 H28 KIH 1 22.400 20.200 16.810 1.00 0.00

ATOM 79 O3 KIH 1 18.590 18.720 17.580 1.00 0.00

ATOM 80 O4 KIH 1 17.570 16.670 22.470 1.00 0.00

ATOM 81 C49 KIH 1 20.040 16.510 21.770 1.00 0.00

ATOM 82 F1 KIH 1 20.970 16.890 20.880 1.00 0.00

ATOM 83 F2 KIH 1 20.710 16.360 22.930 1.00 0.00

ATOM 84 F3 KIH 1 19.700 15.270 21.360 1.00 0.00

ATOM 85 N1 KIH 1 18.160 18.090 20.400 1.00 0.00

ATOM 86 P1 KIH 1 18.620 19.210 19.080 1.00 0.00

ATOM 87 S1 KIH 1 18.590 17.530 21.940 1.00 0.00

ATOM 88 C50 KIH 1 23.800 22.130 19.510 1.00 0.00

ATOM 89 C51 KIH 1 23.630 20.820 19.890 1.00 0.00

ATOM 90 C52 KIH 1 24.620 20.130 20.540 1.00 0.00

ATOM 91 C53 KIH 1 25.700 20.840 21.020 1.00 0.00

ATOM 92 C54 KIH 1 25.850 22.180 20.760 1.00 0.00

ATOM 93 C55 KIH 1 24.920 22.800 19.970 1.00 0.00

ATOM 94 C56 KIH 1 26.920 22.900 21.240 1.00 0.00

ATOM 95 C57 KIH 1 27.040 24.250 21.030 1.00 0.00

ATOM 96 C58 KIH 1 26.090 24.890 20.270 1.00 0.00

ATOM 97 C59 KIH 1 25.050 24.160 19.760 1.00 0.00

ATOM 98 C60 KIH 1 24.470 18.670 20.810 1.00 0.00

ATOM 99 C61 KIH 1 24.440 18.170 22.080 1.00 0.00

ATOM 100 C62 KIH 1 24.230 16.850 22.400 1.00 0.00

ATOM 101 C63 KIH 1 23.960 16.410 23.670 1.00 0.00

ATOM 102 C64 KIH 1 24.270 17.150 24.790 1.00 0.00

ATOM 103 C65 KIH 1 24.070 16.640 26.050 1.00 0.00

ATOM 104 C66 KIH 1 23.570 15.370 26.220 1.00 0.00

ATOM 105 C67 KIH 1 23.410 14.540 25.140 1.00 0.00

ATOM 106 C68 KIH 1 23.550 15.110 23.890 1.00 0.00

ATOM 107 C69 KIH 1 23.590 14.210 22.860 1.00 0.00

ATOM 108 C70 KIH 1 23.860 14.650 21.580 1.00 0.00

ATOM 109 C71 KIH 1 24.160 15.970 21.350 1.00 0.00

ATOM 110 C72 KIH 1 24.330 16.450 20.070 1.00 0.00

ATOM 111 C73 KIH 1 24.510 17.770 19.760 1.00 0.00

ATOM 112 H29 KIH 1 26.420 20.330 21.630 1.00 0.00

ATOM 113 H30 KIH 1 27.670 22.400 21.830 1.00 0.00

ATOM 114 H31 KIH 1 27.870 24.810 21.460 1.00 0.00

ATOM 115 H32 KIH 1 26.120 25.940 20.030 1.00 0.00

ATOM 116 H33 KIH 1 24.300 24.590 19.110 1.00 0.00

ATOM 117 H34 KIH 1 24.350 18.970 22.800 1.00 0.00

ATOM 118 H35 KIH 1 24.770 18.110 24.760 1.00 0.00

ATOM 119 H36 KIH 1 24.260 17.380 26.820 1.00 0.00

ATOM 120 H37 KIH 1 23.500 15.110 27.260 1.00 0.00

ATOM 121 H38 KIH 1 23.160 13.520 25.340 1.00 0.00

ATOM 122 H39 KIH 1 23.340 13.180 23.060 1.00 0.00

ATOM 123 H40 KIH 1 23.800 13.980 20.730 1.00 0.00

ATOM 124 H41 KIH 1 24.250 15.700 19.300 1.00 0.00

ATOM 125 H42 KIH 1 24.540 18.030 18.710 1.00 0.00

ATOM 126 O5 KIH 1 22.460 20.140 19.570 1.00 0.00

ATOM 127 O6 KIH 1 21.920 19.280 24.020 1.00 0.00

ATOM 128 C74 KIH 1 22.730 22.630 18.610 1.00 0.00

ATOM 129 C75 KIH 1 21.450 22.800 19.080 1.00 0.00

ATOM 130 C76 KIH 1 20.560 23.560 18.350 1.00 0.00

ATOM 131 C77 KIH 1 20.970 24.180 17.190 1.00 0.00

ATOM 132 C78 KIH 1 22.150 23.780 16.630 1.00 0.00

ATOM 133 C79 KIH 1 23.080 23.080 17.360 1.00 0.00

ATOM 134 C80 KIH 1 22.440 24.300 15.380 1.00 0.00

ATOM 135 C81 KIH 1 23.690 24.070 14.830 1.00 0.00

ATOM 136 C82 KIH 1 24.570 23.250 15.470 1.00 0.00

ATOM 137 C83 KIH 1 24.290 22.860 16.760 1.00 0.00

ATOM 138 C84 KIH 1 19.280 24.060 18.920 1.00 0.00

ATOM 139 C85 KIH 1 19.210 24.590 20.180 1.00 0.00

ATOM 140 C86 KIH 1 18.030 25.060 20.720 1.00 0.00

ATOM 141 C87 KIH 1 17.920 25.560 21.990 1.00 0.00

ATOM 142 C88 KIH 1 18.980 25.850 22.820 1.00 0.00

ATOM 143 C89 KIH 1 18.860 26.320 24.110 1.00 0.00

ATOM 144 C90 KIH 1 17.570 26.490 24.550 1.00 0.00

ATOM 145 C91 KIH 1 16.470 26.440 23.730 1.00 0.00

ATOM 146 C92 KIH 1 16.680 25.830 22.510 1.00 0.00

ATOM 147 C93 KIH 1 15.530 25.620 21.790 1.00 0.00

ATOM 148 C94 KIH 1 15.640 25.120 20.510 1.00 0.00

ATOM 149 C95 KIH 1 16.880 24.870 19.980 1.00 0.00

ATOM 150 C96 KIH 1 16.890 24.360 18.700 1.00 0.00

ATOM 151 C97 KIH 1 18.110 23.980 18.190 1.00 0.00

ATOM 152 H43 KIH 1 20.270 24.750 16.590 1.00 0.00

ATOM 153 H44 KIH 1 21.820 25.100 15.020 1.00 0.00

ATOM 154 H45 KIH 1 23.900 24.600 13.910 1.00 0.00

ATOM 155 H46 KIH 1 25.510 22.940 15.020 1.00 0.00

ATOM 156 H47 KIH 1 25.110 22.250 17.120 1.00 0.00

ATOM 157 H48 KIH 1 20.140 24.640 20.730 1.00 0.00

ATOM 158 H49 KIH 1 20.010 25.800 22.500 1.00 0.00

ATOM 159 H50 KIH 1 19.690 26.380 24.790 1.00 0.00

ATOM 160 H51 KIH 1 17.400 26.890 25.550 1.00 0.00

ATOM 161 H52 KIH 1 15.440 26.540 24.070 1.00 0.00

ATOM 162 H53 KIH 1 14.510 25.730 22.130 1.00 0.00

ATOM 163 H54 KIH 1 14.750 24.990 19.900 1.00 0.00

ATOM 164 H55 KIH 1 16.010 24.230 18.090 1.00 0.00

ATOM 165 H56 KIH 1 18.170 23.590 17.190 1.00 0.00

ATOM 166 O7 KIH 1 20.910 22.090 20.140 1.00 0.00

ATOM 167 O8 KIH 1 22.850 21.230 22.900 1.00 0.00

ATOM 168 C98 KIH 1 20.510 21.530 23.950 1.00 0.00

ATOM 169 F4 KIH 1 20.920 22.090 25.110 1.00 0.00

ATOM 170 F5 KIH 1 20.200 22.570 23.180 1.00 0.00

ATOM 171 F6 KIH 1 19.310 20.970 24.190 1.00 0.00

ATOM 172 N2 KIH 1 21.010 19.960 21.740 1.00 0.00

ATOM 173 P2 KIH 1 21.060 20.520 20.190 1.00 0.00

ATOM 174 S2 KIH 1 21.680 20.420 23.180 1.00 0.00

ATOM 175 N3 KIH 1 19.880 19.910 19.490 1.00 0.00

ATOM 176 H57 KIH 1 20.440 19.110 21.850 1.00 0.00

TER

ENDMDL

REMARK GENERATED BY TRJCONV

TITLE KIH t= 5000.00000

REMARK THIS IS A SIMULATION BOX

CRYST1 38.271 38.271 38.271 90.00 90.00 90.00 P 1 1

MODEL 11

ATOM 1 C1 KIH 1 16.280 19.920 17.200 1.00 0.00

ATOM 2 C2 KIH 1 16.410 20.340 18.500 1.00 0.00

ATOM 3 C3 KIH 1 15.330 20.250 19.340 1.00 0.00

ATOM 4 C4 KIH 1 14.100 19.770 18.940 1.00 0.00

ATOM 5 C5 KIH 1 13.990 19.340 17.640 1.00 0.00

ATOM 6 C6 KIH 1 15.060 19.450 16.780 1.00 0.00

ATOM 7 C7 KIH 1 12.760 18.880 17.210 1.00 0.00

ATOM 8 C8 KIH 1 12.620 18.500 15.890 1.00 0.00

ATOM 9 C9 KIH 1 13.690 18.490 15.020 1.00 0.00

ATOM 10 C10 KIH 1 14.880 18.970 15.500 1.00 0.00

ATOM 11 C11 KIH 1 15.450 20.740 20.750 1.00 0.00

ATOM 12 C12 KIH 1 15.480 20.000 21.910 1.00 0.00

ATOM 13 C13 KIH 1 15.660 20.570 23.140 1.00 0.00

ATOM 14 C14 KIH 1 15.540 19.820 24.290 1.00 0.00

ATOM 15 C15 KIH 1 15.460 18.450 24.320 1.00 0.00

ATOM 16 C16 KIH 1 15.400 17.720 25.480 1.00 0.00

ATOM 17 C17 KIH 1 15.310 18.390 26.670 1.00 0.00

ATOM 18 C18 KIH 1 15.330 19.770 26.680 1.00 0.00

ATOM 19 C19 KIH 1 15.500 20.470 25.500 1.00 0.00

ATOM 20 C20 KIH 1 15.710 21.820 25.610 1.00 0.00

ATOM 21 C21 KIH 1 15.940 22.520 24.460 1.00 0.00

ATOM 22 C22 KIH 1 15.850 21.930 23.220 1.00 0.00

ATOM 23 C23 KIH 1 15.870 22.670 22.060 1.00 0.00

ATOM 24 C24 KIH 1 15.620 22.100 20.830 1.00 0.00

ATOM 25 H1 KIH 1 13.250 19.660 19.590 1.00 0.00

ATOM 26 H2 KIH 1 11.880 18.880 17.830 1.00 0.00

ATOM 27 H3 KIH 1 11.650 18.170 15.560 1.00 0.00

ATOM 28 H4 KIH 1 13.580 18.250 13.980 1.00 0.00

ATOM 29 H5 KIH 1 15.710 18.970 14.820 1.00 0.00

ATOM 30 H6 KIH 1 15.470 18.930 21.720 1.00 0.00

ATOM 31 H7 KIH 1 15.590 17.820 23.440 1.00 0.00

ATOM 32 H8 KIH 1 15.440 16.640 25.430 1.00 0.00

ATOM 33 H9 KIH 1 15.210 17.850 27.600 1.00 0.00

ATOM 34 H10 KIH 1 15.340 20.300 27.610 1.00 0.00

ATOM 35 H11 KIH 1 15.800 22.370 26.540 1.00 0.00

ATOM 36 H12 KIH 1 16.130 23.580 24.590 1.00 0.00

ATOM 37 H13 KIH 1 16.070 23.710 22.260 1.00 0.00

ATOM 38 H14 KIH 1 15.670 22.660 19.910 1.00 0.00

ATOM 39 O1 KIH 1 17.660 20.530 19.060 1.00 0.00

ATOM 40 O2 KIH 1 19.220 18.360 22.760 1.00 0.00

ATOM 41 C25 KIH 1 17.450 20.130 16.290 1.00 0.00

ATOM 42 C26 KIH 1 18.580 19.380 16.550 1.00 0.00

ATOM 43 C27 KIH 1 19.690 19.570 15.770 1.00 0.00

ATOM 44 C28 KIH 1 19.790 20.540 14.790 1.00 0.00

ATOM 45 C29 KIH 1 18.660 21.310 14.590 1.00 0.00

ATOM 46 C30 KIH 1 17.500 21.080 15.300 1.00 0.00

ATOM 47 C31 KIH 1 18.680 22.320 13.670 1.00 0.00

ATOM 48 C32 KIH 1 17.640 23.200 13.500 1.00 0.00

ATOM 49 C33 KIH 1 16.500 22.950 14.210 1.00 0.00

ATOM 50 C34 KIH 1 16.410 21.880 15.080 1.00 0.00

ATOM 51 C35 KIH 1 20.910 18.760 16.050 1.00 0.00

ATOM 52 C36 KIH 1 20.960 17.440 15.670 1.00 0.00

ATOM 53 C37 KIH 1 22.040 16.610 15.780 1.00 0.00

ATOM 54 C38 KIH 1 22.000 15.290 15.370 1.00 0.00

ATOM 55 C39 KIH 1 20.850 14.720 14.880 1.00 0.00

ATOM 56 C40 KIH 1 20.700 13.380 14.640 1.00 0.00

ATOM 57 C41 KIH 1 21.790 12.560 14.810 1.00 0.00

ATOM 58 C42 KIH 1 23.000 13.130 15.140 1.00 0.00

ATOM 59 C43 KIH 1 23.080 14.460 15.490 1.00 0.00

ATOM 60 C44 KIH 1 24.250 15.000 15.970 1.00 0.00

ATOM 61 C45 KIH 1 24.280 16.310 16.370 1.00 0.00

ATOM 62 C46 KIH 1 23.190 17.150 16.310 1.00 0.00

ATOM 63 C47 KIH 1 23.200 18.440 16.800 1.00 0.00

ATOM 64 C48 KIH 1 22.080 19.210 16.620 1.00 0.00

ATOM 65 H15 KIH 1 20.690 20.710 14.230 1.00 0.00

ATOM 66 H16 KIH 1 19.640 22.560 13.220 1.00 0.00

ATOM 67 H17 KIH 1 17.680 23.940 12.710 1.00 0.00

ATOM 68 H18 KIH 1 15.690 23.630 13.970 1.00 0.00

ATOM 69 H19 KIH 1 15.540 21.710 15.700 1.00 0.00

ATOM 70 H20 KIH 1 20.100 17.040 15.140 1.00 0.00

ATOM 71 H21 KIH 1 19.900 15.210 14.690 1.00 0.00

ATOM 72 H22 KIH 1 19.770 13.020 14.210 1.00 0.00

ATOM 73 H23 KIH 1 21.760 11.510 14.580 1.00 0.00

ATOM 74 H24 KIH 1 23.800 12.470 15.440 1.00 0.00

ATOM 75 H25 KIH 1 25.100 14.380 16.220 1.00 0.00

ATOM 76 H26 KIH 1 25.230 16.650 16.780 1.00 0.00

ATOM 77 H27 KIH 1 24.080 18.770 17.340 1.00 0.00

ATOM 78 H28 KIH 1 22.110 20.260 16.910 1.00 0.00

ATOM 79 O3 KIH 1 18.650 18.600 17.690 1.00 0.00

ATOM 80 O4 KIH 1 17.490 16.650 22.500 1.00 0.00

ATOM 81 C49 KIH 1 19.880 16.270 21.530 1.00 0.00

ATOM 82 F1 KIH 1 20.920 16.920 20.990 1.00 0.00

ATOM 83 F2 KIH 1 20.330 15.600 22.610 1.00 0.00

ATOM 84 F3 KIH 1 19.540 15.280 20.690 1.00 0.00

ATOM 85 N1 KIH 1 18.090 18.140 20.430 1.00 0.00

ATOM 86 P1 KIH 1 18.600 19.260 19.130 1.00 0.00

ATOM 87 S1 KIH 1 18.550 17.420 21.900 1.00 0.00

ATOM 88 C50 KIH 1 23.740 21.900 19.360 1.00 0.00

ATOM 89 C51 KIH 1 23.640 20.610 19.820 1.00 0.00

ATOM 90 C52 KIH 1 24.620 20.070 20.640 1.00 0.00

ATOM 91 C53 KIH 1 25.700 20.810 21.020 1.00 0.00

ATOM 92 C54 KIH 1 25.720 22.140 20.670 1.00 0.00

ATOM 93 C55 KIH 1 24.720 22.700 19.900 1.00 0.00

ATOM 94 C56 KIH 1 26.770 22.880 21.170 1.00 0.00

ATOM 95 C57 KIH 1 26.770 24.230 20.960 1.00 0.00

ATOM 96 C58 KIH 1 25.830 24.820 20.160 1.00 0.00

ATOM 97 C59 KIH 1 24.800 24.050 19.650 1.00 0.00

ATOM 98 C60 KIH 1 24.410 18.720 21.240 1.00 0.00

ATOM 99 C61 KIH 1 24.580 18.600 22.600 1.00 0.00

ATOM 100 C62 KIH 1 24.340 17.410 23.240 1.00 0.00

ATOM 101 C63 KIH 1 24.140 17.410 24.600 1.00 0.00

ATOM 102 C64 KIH 1 24.350 18.450 25.470 1.00 0.00

ATOM 103 C65 KIH 1 24.020 18.370 26.800 1.00 0.00

ATOM 104 C66 KIH 1 23.580 17.190 27.330 1.00 0.00

ATOM 105 C67 KIH 1 23.410 16.110 26.490 1.00 0.00

ATOM 106 C68 KIH 1 23.700 16.230 25.150 1.00 0.00

ATOM 107 C69 KIH 1 23.400 15.080 24.450 1.00 0.00

ATOM 108 C70 KIH 1 23.540 15.140 23.080 1.00 0.00

ATOM 109 C71 KIH 1 23.970 16.300 22.500 1.00 0.00

ATOM 110 C72 KIH 1 23.930 16.400 21.120 1.00 0.00

ATOM 111 C73 KIH 1 24.130 17.610 20.500 1.00 0.00

ATOM 112 H29 KIH 1 26.510 20.360 21.580 1.00 0.00

ATOM 113 H30 KIH 1 27.540 22.420 21.780 1.00 0.00

ATOM 114 H31 KIH 1 27.510 24.870 21.430 1.00 0.00

ATOM 115 H32 KIH 1 25.890 25.890 20.050 1.00 0.00

ATOM 116 H33 KIH 1 24.010 24.630 19.190 1.00 0.00

ATOM 117 H34 KIH 1 24.720 19.490 23.210 1.00 0.00

ATOM 118 H35 KIH 1 24.780 19.360 25.090 1.00 0.00

ATOM 119 H36 KIH 1 24.300 19.190 27.450 1.00 0.00

ATOM 120 H37 KIH 1 23.500 17.050 28.400 1.00 0.00

ATOM 121 H38 KIH 1 23.000 15.240 26.970 1.00 0.00

ATOM 122 H39 KIH 1 22.990 14.170 24.870 1.00 0.00

ATOM 123 H40 KIH 1 23.120 14.350 22.470 1.00 0.00

ATOM 124 H41 KIH 1 23.720 15.570 20.480 1.00 0.00

ATOM 125 H42 KIH 1 23.990 17.640 19.420 1.00 0.00

ATOM 126 O5 KIH 1 22.470 19.890 19.640 1.00 0.00

ATOM 127 O6 KIH 1 21.880 19.330 23.980 1.00 0.00

ATOM 128 C74 KIH 1 22.750 22.510 18.430 1.00 0.00

ATOM 129 C75 KIH 1 21.450 22.650 18.830 1.00 0.00

ATOM 130 C76 KIH 1 20.540 23.320 18.050 1.00 0.00

ATOM 131 C77 KIH 1 20.840 23.510 16.720 1.00 0.00

ATOM 132 C78 KIH 1 22.110 23.240 16.280 1.00 0.00

ATOM 133 C79 KIH 1 23.110 22.810 17.120 1.00 0.00

ATOM 134 C80 KIH 1 22.340 23.330 14.920 1.00 0.00

ATOM 135 C81 KIH 1 23.600 23.090 14.460 1.00 0.00

ATOM 136 C82 KIH 1 24.630 22.730 15.300 1.00 0.00

ATOM 137 C83 KIH 1 24.350 22.520 16.630 1.00 0.00

ATOM 138 C84 KIH 1 19.230 23.850 18.520 1.00 0.00

ATOM 139 C85 KIH 1 19.180 24.640 19.640 1.00 0.00

ATOM 140 C86 KIH 1 18.050 25.200 20.190 1.00 0.00

ATOM 141 C87 KIH 1 17.940 25.890 21.380 1.00 0.00

ATOM 142 C88 KIH 1 19.020 26.150 22.200 1.00 0.00

ATOM 143 C89 KIH 1 18.940 26.850 23.390 1.00 0.00

ATOM 144 C90 KIH 1 17.710 27.440 23.600 1.00 0.00

ATOM 145 C91 KIH 1 16.590 27.200 22.830 1.00 0.00

ATOM 146 C92 KIH 1 16.730 26.480 21.680 1.00 0.00

ATOM 147 C93 KIH 1 15.600 26.340 20.890 1.00 0.00

ATOM 148 C94 KIH 1 15.710 25.590 19.740 1.00 0.00

ATOM 149 C95 KIH 1 16.930 25.060 19.420 1.00 0.00

ATOM 150 C96 KIH 1 16.900 24.330 18.250 1.00 0.00

ATOM 151 C97 KIH 1 18.060 23.740 17.790 1.00 0.00

ATOM 152 H43 KIH 1 20.140 23.930 16.010 1.00 0.00

ATOM 153 H44 KIH 1 21.580 23.770 14.280 1.00 0.00

ATOM 154 H45 KIH 1 23.830 23.350 13.440 1.00 0.00

ATOM 155 H46 KIH 1 25.620 22.570 14.900 1.00 0.00

ATOM 156 H47 KIH 1 25.220 22.240 17.220 1.00 0.00

ATOM 157 H48 KIH 1 20.050 24.600 20.290 1.00 0.00

ATOM 158 H49 KIH 1 19.960 25.680 21.960 1.00 0.00

ATOM 159 H50 KIH 1 19.900 26.970 23.850 1.00 0.00

ATOM 160 H51 KIH 1 17.600 28.150 24.400 1.00 0.00

ATOM 161 H52 KIH 1 15.790 27.870 23.120 1.00 0.00

ATOM 162 H53 KIH 1 14.640 26.730 21.190 1.00 0.00

ATOM 163 H54 KIH 1 14.800 25.510 19.170 1.00 0.00

ATOM 164 H55 KIH 1 16.010 24.300 17.650 1.00 0.00

ATOM 165 H56 KIH 1 18.100 23.130 16.900 1.00 0.00

ATOM 166 O7 KIH 1 21.000 22.040 19.980 1.00 0.00

ATOM 167 O8 KIH 1 22.840 21.290 22.810 1.00 0.00

ATOM 168 C98 KIH 1 20.340 21.430 23.910 1.00 0.00

ATOM 169 F4 KIH 1 20.690 21.830 25.140 1.00 0.00

ATOM 170 F5 KIH 1 20.050 22.540 23.210 1.00 0.00

ATOM 171 F6 KIH 1 19.160 20.830 24.050 1.00 0.00

ATOM 172 N2 KIH 1 21.050 19.980 21.750 1.00 0.00

ATOM 173 P2 KIH 1 21.100 20.470 20.180 1.00 0.00

ATOM 174 S2 KIH 1 21.690 20.510 23.180 1.00 0.00

ATOM 175 N3 KIH 1 19.920 19.880 19.480 1.00 0.00

ATOM 176 H57 KIH 1 20.520 19.110 21.840 1.00 0.00

TER

ENDMDL

REMARK GENERATED BY TRJCONV

TITLE KIH t= 5500.00000

REMARK THIS IS A SIMULATION BOX

CRYST1 38.240 38.240 38.240 90.00 90.00 90.00 P 1 1

MODEL 12

ATOM 1 C1 KIH 1 16.130 19.990 17.490 1.00 0.00

ATOM 2 C2 KIH 1 16.380 20.230 18.810 1.00 0.00

ATOM 3 C3 KIH 1 15.370 20.220 19.740 1.00 0.00

ATOM 4 C4 KIH 1 14.090 19.960 19.310 1.00 0.00

ATOM 5 C5 KIH 1 13.840 19.690 17.990 1.00 0.00

ATOM 6 C6 KIH 1 14.880 19.520 17.120 1.00 0.00

ATOM 7 C7 KIH 1 12.560 19.460 17.530 1.00 0.00

ATOM 8 C8 KIH 1 12.350 18.980 16.270 1.00 0.00

ATOM 9 C9 KIH 1 13.440 18.720 15.450 1.00 0.00

ATOM 10 C10 KIH 1 14.720 18.990 15.860 1.00 0.00

ATOM 11 C11 KIH 1 15.730 20.690 21.100 1.00 0.00

ATOM 12 C12 KIH 1 15.410 19.930 22.200 1.00 0.00

ATOM 13 C13 KIH 1 15.640 20.280 23.510 1.00 0.00

ATOM 14 C14 KIH 1 15.370 19.520 24.620 1.00 0.00

ATOM 15 C15 KIH 1 14.870 18.240 24.560 1.00 0.00

ATOM 16 C16 KIH 1 14.470 17.570 25.690 1.00 0.00

ATOM 17 C17 KIH 1 14.610 18.130 26.930 1.00 0.00

ATOM 18 C18 KIH 1 15.150 19.400 27.020 1.00 0.00

ATOM 19 C19 KIH 1 15.430 20.090 25.870 1.00 0.00

ATOM 20 C20 KIH 1 15.950 21.350 26.040 1.00 0.00

ATOM 21 C21 KIH 1 16.450 22.030 24.960 1.00 0.00

ATOM 22 C22 KIH 1 16.190 21.540 23.700 1.00 0.00

ATOM 23 C23 KIH 1 16.590 22.280 22.620 1.00 0.00

ATOM 24 C24 KIH 1 16.390 21.890 21.310 1.00 0.00

ATOM 25 H1 KIH 1 13.270 20.110 20.010 1.00 0.00

ATOM 26 H2 KIH 1 11.750 19.500 18.240 1.00 0.00

ATOM 27 H3 KIH 1 11.370 18.630 15.970 1.00 0.00

ATOM 28 H4 KIH 1 13.250 18.190 14.530 1.00 0.00

ATOM 29 H5 KIH 1 15.570 18.800 15.220 1.00 0.00

ATOM 30 H6 KIH 1 15.020 18.970 21.910 1.00 0.00

ATOM 31 H7 KIH 1 14.750 17.800 23.580 1.00 0.00

ATOM 32 H8 KIH 1 14.110 16.550 25.620 1.00 0.00

ATOM 33 H9 KIH 1 14.180 17.600 27.770 1.00 0.00

ATOM 34 H10 KIH 1 15.460 19.850 27.960 1.00 0.00

ATOM 35 H11 KIH 1 16.110 21.820 27.000 1.00 0.00

ATOM 36 H12 KIH 1 17.080 22.880 25.140 1.00 0.00

ATOM 37 H13 KIH 1 16.920 23.300 22.760 1.00 0.00

ATOM 38 H14 KIH 1 16.780 22.540 20.540 1.00 0.00

ATOM 39 O1 KIH 1 17.700 20.430 19.200 1.00 0.00

ATOM 40 O2 KIH 1 19.330 18.410 22.540 1.00 0.00

ATOM 41 C25 KIH 1 17.210 20.260 16.500 1.00 0.00

ATOM 42 C26 KIH 1 18.400 19.590 16.690 1.00 0.00

ATOM 43 C27 KIH 1 19.490 19.840 15.880 1.00 0.00

ATOM 44 C28 KIH 1 19.290 20.650 14.790 1.00 0.00

ATOM 45 C29 KIH 1 18.110 21.330 14.580 1.00 0.00

ATOM 46 C30 KIH 1 17.100 21.200 15.500 1.00 0.00

ATOM 47 C31 KIH 1 17.970 22.120 13.460 1.00 0.00

ATOM 48 C32 KIH 1 16.850 22.900 13.300 1.00 0.00

ATOM 49 C33 KIH 1 15.850 22.810 14.230 1.00 0.00

ATOM 50 C34 KIH 1 15.980 21.990 15.330 1.00 0.00

ATOM 51 C35 KIH 1 20.790 19.110 15.970 1.00 0.00

ATOM 52 C36 KIH 1 20.920 17.810 15.540 1.00 0.00

ATOM 53 C37 KIH 1 22.110 17.130 15.530 1.00 0.00

ATOM 54 C38 KIH 1 22.250 15.880 14.970 1.00 0.00

ATOM 55 C39 KIH 1 21.200 15.120 14.520 1.00 0.00

ATOM 56 C40 KIH 1 21.360 13.800 14.160 1.00 0.00

ATOM 57 C41 KIH 1 22.620 13.280 14.010 1.00 0.00

ATOM 58 C42 KIH 1 23.690 14.050 14.410 1.00 0.00

ATOM 59 C43 KIH 1 23.510 15.320 14.890 1.00 0.00

ATOM 60 C44 KIH 1 24.560 16.020 15.440 1.00 0.00

ATOM 61 C45 KIH 1 24.420 17.200 16.120 1.00 0.00

ATOM 62 C46 KIH 1 23.180 17.790 16.080 1.00 0.00

ATOM 63 C47 KIH 1 23.060 19.060 16.600 1.00 0.00

ATOM 64 C48 KIH 1 21.840 19.710 16.610 1.00 0.00

ATOM 65 H15 KIH 1 20.140 20.860 14.150 1.00 0.00

ATOM 66 H16 KIH 1 18.710 22.040 12.680 1.00 0.00

ATOM 67 H17 KIH 1 16.730 23.470 12.380 1.00 0.00

ATOM 68 H18 KIH 1 14.890 23.230 13.980 1.00 0.00

ATOM 69 H19 KIH 1 15.100 21.930 15.940 1.00 0.00

ATOM 70 H20 KIH 1 19.990 17.470 15.100 1.00 0.00

ATOM 71 H21 KIH 1 20.170 15.460 14.610 1.00 0.00

ATOM 72 H22 KIH 1 20.520 13.200 13.840 1.00 0.00

ATOM 73 H23 KIH 1 22.730 12.290 13.600 1.00 0.00

ATOM 74 H24 KIH 1 24.690 13.650 14.360 1.00 0.00

ATOM 75 H25 KIH 1 25.540 15.550 15.460 1.00 0.00

ATOM 76 H26 KIH 1 25.230 17.690 16.640 1.00 0.00

ATOM 77 H27 KIH 1 23.860 19.650 17.030 1.00 0.00

ATOM 78 H28 KIH 1 21.740 20.770 16.830 1.00 0.00

ATOM 79 O3 KIH 1 18.560 18.650 17.670 1.00 0.00

ATOM 80 O4 KIH 1 17.330 16.950 22.700 1.00 0.00

ATOM 81 C49 KIH 1 19.650 16.100 21.740 1.00 0.00

ATOM 82 F1 KIH 1 20.650 16.390 20.900 1.00 0.00

ATOM 83 F2 KIH 1 20.220 15.810 22.910 1.00 0.00

ATOM 84 F3 KIH 1 19.110 14.960 21.320 1.00 0.00

ATOM 85 N1 KIH 1 18.110 18.030 20.390 1.00 0.00

ATOM 86 P1 KIH 1 18.680 19.200 19.160 1.00 0.00

ATOM 87 S1 KIH 1 18.460 17.430 21.950 1.00 0.00

ATOM 88 C50 KIH 1 23.770 22.070 19.580 1.00 0.00

ATOM 89 C51 KIH 1 23.610 20.720 19.790 1.00 0.00

ATOM 90 C52 KIH 1 24.610 20.060 20.480 1.00 0.00

ATOM 91 C53 KIH 1 25.740 20.690 20.930 1.00 0.00

ATOM 92 C54 KIH 1 25.790 22.050 20.770 1.00 0.00

ATOM 93 C55 KIH 1 24.800 22.780 20.160 1.00 0.00

ATOM 94 C56 KIH 1 26.800 22.700 21.440 1.00 0.00

ATOM 95 C57 KIH 1 26.920 24.070 21.420 1.00 0.00

ATOM 96 C58 KIH 1 26.050 24.780 20.620 1.00 0.00

ATOM 97 C59 KIH 1 24.970 24.140 20.050 1.00 0.00

ATOM 98 C60 KIH 1 24.390 18.660 20.930 1.00 0.00

ATOM 99 C61 KIH 1 24.300 18.310 22.260 1.00 0.00

ATOM 100 C62 KIH 1 24.020 17.020 22.660 1.00 0.00

ATOM 101 C63 KIH 1 23.810 16.630 23.960 1.00 0.00

ATOM 102 C64 KIH 1 23.940 17.530 25.000 1.00 0.00

ATOM 103 C65 KIH 1 23.750 17.090 26.280 1.00 0.00

ATOM 104 C66 KIH 1 23.480 15.770 26.530 1.00 0.00

ATOM 105 C67 KIH 1 23.590 14.810 25.550 1.00 0.00

ATOM 106 C68 KIH 1 23.710 15.290 24.270 1.00 0.00

ATOM 107 C69 KIH 1 23.620 14.350 23.280 1.00 0.00

ATOM 108 C70 KIH 1 23.720 14.700 21.950 1.00 0.00

ATOM 109 C71 KIH 1 23.970 16.040 21.690 1.00 0.00

ATOM 110 C72 KIH 1 24.190 16.360 20.380 1.00 0.00

ATOM 111 C73 KIH 1 24.410 17.660 19.980 1.00 0.00

ATOM 112 H29 KIH 1 26.480 20.110 21.470 1.00 0.00

ATOM 113 H30 KIH 1 27.500 22.130 22.040 1.00 0.00

ATOM 114 H31 KIH 1 27.710 24.570 21.960 1.00 0.00

ATOM 115 H32 KIH 1 26.210 25.840 20.460 1.00 0.00

ATOM 116 H33 KIH 1 24.280 24.820 19.570 1.00 0.00

ATOM 117 H34 KIH 1 24.350 19.120 22.970 1.00 0.00

ATOM 118 H35 KIH 1 24.040 18.600 24.880 1.00 0.00

ATOM 119 H36 KIH 1 23.720 17.880 27.030 1.00 0.00

ATOM 120 H37 KIH 1 23.380 15.440 27.560 1.00 0.00

ATOM 121 H38 KIH 1 23.520 13.750 25.720 1.00 0.00

ATOM 122 H39 KIH 1 23.540 13.280 23.470 1.00 0.00

ATOM 123 H40 KIH 1 23.830 14.070 21.100 1.00 0.00

ATOM 124 H41 KIH 1 24.060 15.600 19.620 1.00 0.00

ATOM 125 H42 KIH 1 24.590 17.930 18.950 1.00 0.00

ATOM 126 O5 KIH 1 22.440 20.050 19.550 1.00 0.00

ATOM 127 O6 KIH 1 21.710 19.260 23.960 1.00 0.00

ATOM 128 C74 KIH 1 22.840 22.790 18.670 1.00 0.00

ATOM 129 C75 KIH 1 21.550 22.950 19.110 1.00 0.00

ATOM 130 C76 KIH 1 20.680 23.750 18.400 1.00 0.00

ATOM 131 C77 KIH 1 21.150 24.290 17.220 1.00 0.00

ATOM 132 C78 KIH 1 22.420 24.090 16.720 1.00 0.00

ATOM 133 C79 KIH 1 23.260 23.290 17.460 1.00 0.00

ATOM 134 C80 KIH 1 22.850 24.740 15.590 1.00 0.00

ATOM 135 C81 KIH 1 24.110 24.490 15.090 1.00 0.00

ATOM 136 C82 KIH 1 24.990 23.770 15.850 1.00 0.00

ATOM 137 C83 KIH 1 24.550 23.130 16.990 1.00 0.00

ATOM 138 C84 KIH 1 19.310 24.170 18.790 1.00 0.00

ATOM 139 C85 KIH 1 19.070 24.750 20.020 1.00 0.00

ATOM 140 C86 KIH 1 17.820 25.200 20.360 1.00 0.00

ATOM 141 C87 KIH 1 17.490 25.850 21.530 1.00 0.00

ATOM 142 C88 KIH 1 18.430 26.090 22.510 1.00 0.00

ATOM 143 C89 KIH 1 18.130 26.840 23.620 1.00 0.00

ATOM 144 C90 KIH 1 16.880 27.390 23.790 1.00 0.00

ATOM 145 C91 KIH 1 15.980 27.250 22.760 1.00 0.00

ATOM 146 C92 KIH 1 16.250 26.420 21.690 1.00 0.00

ATOM 147 C93 KIH 1 15.220 26.220 20.820 1.00 0.00

ATOM 148 C94 KIH 1 15.530 25.420 19.750 1.00 0.00

ATOM 149 C95 KIH 1 16.780 24.910 19.500 1.00 0.00

ATOM 150 C96 KIH 1 16.960 24.380 18.240 1.00 0.00

ATOM 151 C97 KIH 1 18.240 23.950 17.960 1.00 0.00

ATOM 152 H43 KIH 1 20.480 25.000 16.760 1.00 0.00

ATOM 153 H44 KIH 1 22.140 25.360 15.080 1.00 0.00

ATOM 154 H45 KIH 1 24.430 25.000 14.190 1.00 0.00

ATOM 155 H46 KIH 1 26.010 23.790 15.480 1.00 0.00

ATOM 156 H47 KIH 1 25.300 22.520 17.470 1.00 0.00

ATOM 157 H48 KIH 1 19.940 24.900 20.630 1.00 0.00

ATOM 158 H49 KIH 1 19.480 25.860 22.390 1.00 0.00

ATOM 159 H50 KIH 1 18.810 26.980 24.460 1.00 0.00

ATOM 160 H51 KIH 1 16.820 28.080 24.610 1.00 0.00

ATOM 161 H52 KIH 1 15.020 27.750 22.800 1.00 0.00

ATOM 162 H53 KIH 1 14.200 26.590 20.830 1.00 0.00

ATOM 163 H54 KIH 1 14.640 25.270 19.160 1.00 0.00

ATOM 164 H55 KIH 1 16.160 24.280 17.530 1.00 0.00

ATOM 165 H56 KIH 1 18.420 23.480 17.000 1.00 0.00

ATOM 166 O7 KIH 1 20.990 22.190 20.120 1.00 0.00

ATOM 167 O8 KIH 1 22.900 21.200 22.900 1.00 0.00

ATOM 168 C98 KIH 1 20.410 21.440 23.890 1.00 0.00

ATOM 169 F4 KIH 1 20.080 22.500 23.150 1.00 0.00

ATOM 170 F5 KIH 1 19.250 20.800 24.120 1.00 0.00

ATOM 171 F6 KIH 1 20.780 21.940 25.070 1.00 0.00

ATOM 172 N2 KIH 1 21.070 19.960 21.680 1.00 0.00

ATOM 173 P2 KIH 1 21.090 20.620 20.170 1.00 0.00

ATOM 174 S2 KIH 1 21.690 20.440 23.130 1.00 0.00

ATOM 175 N3 KIH 1 19.920 19.950 19.520 1.00 0.00

ATOM 176 H57 KIH 1 20.770 18.980 21.730 1.00 0.00

TER

ENDMDL

REMARK GENERATED BY TRJCONV

TITLE KIH t= 6000.00000

REMARK THIS IS A SIMULATION BOX

CRYST1 38.654 38.654 38.654 90.00 90.00 90.00 P 1 1

MODEL 13

ATOM 1 C1 KIH 1 16.440 19.940 16.960 1.00 0.00

ATOM 2 C2 KIH 1 16.420 20.260 18.300 1.00 0.00

ATOM 3 C3 KIH 1 15.290 20.200 19.080 1.00 0.00

ATOM 4 C4 KIH 1 14.110 19.910 18.440 1.00 0.00

ATOM 5 C5 KIH 1 14.130 19.510 17.120 1.00 0.00

ATOM 6 C6 KIH 1 15.260 19.560 16.340 1.00 0.00

ATOM 7 C7 KIH 1 12.920 19.110 16.620 1.00 0.00

ATOM 8 C8 KIH 1 12.830 19.050 15.240 1.00 0.00

ATOM 9 C9 KIH 1 13.930 19.160 14.430 1.00 0.00

ATOM 10 C10 KIH 1 15.170 19.240 15.010 1.00 0.00

ATOM 11 C11 KIH 1 15.230 20.300 20.570 1.00 0.00

ATOM 12 C12 KIH 1 14.820 19.260 21.360 1.00 0.00

ATOM 13 C13 KIH 1 14.890 19.330 22.730 1.00 0.00

ATOM 14 C14 KIH 1 14.630 18.280 23.580 1.00 0.00

ATOM 15 C15 KIH 1 14.440 17.010 23.080 1.00 0.00

ATOM 16 C16 KIH 1 14.420 15.950 23.960 1.00 0.00

ATOM 17 C17 KIH 1 14.370 16.170 25.310 1.00 0.00

ATOM 18 C18 KIH 1 14.360 17.460 25.790 1.00 0.00

ATOM 19 C19 KIH 1 14.620 18.510 24.940 1.00 0.00

ATOM 20 C20 KIH 1 14.840 19.730 25.520 1.00 0.00

ATOM 21 C21 KIH 1 15.070 20.730 24.610 1.00 0.00

ATOM 22 C22 KIH 1 15.220 20.560 23.260 1.00 0.00

ATOM 23 C23 KIH 1 15.570 21.650 22.510 1.00 0.00

ATOM 24 C24 KIH 1 15.630 21.500 21.140 1.00 0.00

ATOM 25 H1 KIH 1 13.210 19.790 19.020 1.00 0.00

ATOM 26 H2 KIH 1 12.110 18.890 17.300 1.00 0.00

ATOM 27 H3 KIH 1 11.910 18.830 14.720 1.00 0.00

ATOM 28 H4 KIH 1 13.890 18.970 13.370 1.00 0.00

ATOM 29 H5 KIH 1 16.080 19.230 14.420 1.00 0.00

ATOM 30 H6 KIH 1 14.610 18.330 20.840 1.00 0.00

ATOM 31 H7 KIH 1 14.440 16.800 22.030 1.00 0.00

ATOM 32 H8 KIH 1 14.480 14.940 23.580 1.00 0.00

ATOM 33 H9 KIH 1 14.450 15.280 25.920 1.00 0.00

ATOM 34 H10 KIH 1 14.470 17.570 26.860 1.00 0.00

ATOM 35 H11 KIH 1 14.730 19.920 26.580 1.00 0.00

ATOM 36 H12 KIH 1 15.060 21.730 25.050 1.00 0.00

ATOM 37 H13 KIH 1 15.780 22.640 22.880 1.00 0.00

ATOM 38 H14 KIH 1 15.980 22.270 20.490 1.00 0.00

ATOM 39 O1 KIH 1 17.630 20.450 18.940 1.00 0.00

ATOM 40 O2 KIH 1 19.210 18.300 22.680 1.00 0.00

ATOM 41 C25 KIH 1 17.710 20.090 16.210 1.00 0.00

ATOM 42 C26 KIH 1 18.800 19.330 16.550 1.00 0.00

ATOM 43 C27 KIH 1 19.970 19.440 15.840 1.00 0.00

ATOM 44 C28 KIH 1 20.050 20.360 14.810 1.00 0.00

ATOM 45 C29 KIH 1 18.960 21.150 14.540 1.00 0.00

ATOM 46 C30 KIH 1 17.800 21.090 15.260 1.00 0.00

ATOM 47 C31 KIH 1 19.020 22.030 13.480 1.00 0.00

ATOM 48 C32 KIH 1 18.000 22.920 13.220 1.00 0.00

ATOM 49 C33 KIH 1 16.890 22.970 14.020 1.00 0.00

ATOM 50 C34 KIH 1 16.810 22.020 15.010 1.00 0.00

ATOM 51 C35 KIH 1 21.090 18.460 15.930 1.00 0.00

ATOM 52 C36 KIH 1 20.940 17.100 15.890 1.00 0.00

ATOM 53 C37 KIH 1 21.960 16.180 15.960 1.00 0.00

ATOM 54 C38 KIH 1 21.730 14.820 15.920 1.00 0.00

ATOM 55 C39 KIH 1 20.560 14.210 15.530 1.00 0.00

ATOM 56 C40 KIH 1 20.360 12.850 15.570 1.00 0.00

ATOM 57 C41 KIH 1 21.400 11.990 15.850 1.00 0.00

ATOM 58 C42 KIH 1 22.590 12.580 16.230 1.00 0.00

ATOM 59 C43 KIH 1 22.760 13.940 16.160 1.00 0.00

ATOM 60 C44 KIH 1 24.020 14.450 16.360 1.00 0.00

ATOM 61 C45 KIH 1 24.250 15.810 16.270 1.00 0.00

ATOM 62 C46 KIH 1 23.200 16.700 16.220 1.00 0.00

ATOM 63 C47 KIH 1 23.390 18.070 16.290 1.00 0.00

ATOM 64 C48 KIH 1 22.350 18.950 16.170 1.00 0.00

ATOM 65 H15 KIH 1 20.970 20.330 14.240 1.00 0.00

ATOM 66 H16 KIH 1 19.910 22.110 12.880 1.00 0.00

ATOM 67 H17 KIH 1 18.050 23.470 12.280 1.00 0.00

ATOM 68 H18 KIH 1 16.110 23.670 13.780 1.00 0.00

ATOM 69 H19 KIH 1 15.910 22.020 15.620 1.00 0.00

ATOM 70 H20 KIH 1 19.920 16.840 15.610 1.00 0.00

ATOM 71 H21 KIH 1 19.680 14.780 15.290 1.00 0.00

ATOM 72 H22 KIH 1 19.530 12.440 15.020 1.00 0.00

ATOM 73 H23 KIH 1 21.410 10.930 15.690 1.00 0.00

ATOM 74 H24 KIH 1 23.410 11.930 16.470 1.00 0.00

ATOM 75 H25 KIH 1 24.860 13.790 16.440 1.00 0.00

ATOM 76 H26 KIH 1 25.220 16.250 16.340 1.00 0.00

ATOM 77 H27 KIH 1 24.390 18.460 16.460 1.00 0.00

ATOM 78 H28 KIH 1 22.480 20.020 16.210 1.00 0.00

ATOM 79 O3 KIH 1 18.850 18.570 17.700 1.00 0.00

ATOM 80 O4 KIH 1 17.360 16.680 22.380 1.00 0.00

ATOM 81 C49 KIH 1 19.630 16.070 21.330 1.00 0.00

ATOM 82 F1 KIH 1 20.710 16.540 20.680 1.00 0.00

ATOM 83 F2 KIH 1 20.090 15.350 22.350 1.00 0.00

ATOM 84 F3 KIH 1 19.080 15.210 20.470 1.00 0.00

ATOM 85 N1 KIH 1 18.020 18.160 20.420 1.00 0.00

ATOM 86 P1 KIH 1 18.620 19.230 19.120 1.00 0.00

ATOM 87 S1 KIH 1 18.500 17.370 21.840 1.00 0.00

ATOM 88 C50 KIH 1 23.930 21.640 19.440 1.00 0.00

ATOM 89 C51 KIH 1 23.680 20.350 19.820 1.00 0.00

ATOM 90 C52 KIH 1 24.550 19.600 20.580 1.00 0.00

ATOM 91 C53 KIH 1 25.670 20.250 21.030 1.00 0.00

ATOM 92 C54 KIH 1 25.930 21.560 20.690 1.00 0.00

ATOM 93 C55 KIH 1 25.080 22.260 19.880 1.00 0.00

ATOM 94 C56 KIH 1 27.100 22.150 21.130 1.00 0.00

ATOM 95 C57 KIH 1 27.380 23.460 20.810 1.00 0.00

ATOM 96 C58 KIH 1 26.530 24.140 19.970 1.00 0.00

ATOM 97 C59 KIH 1 25.360 23.560 19.530 1.00 0.00

ATOM 98 C60 KIH 1 24.340 18.210 21.090 1.00 0.00

ATOM 99 C61 KIH 1 24.080 17.810 22.380 1.00 0.00

ATOM 100 C62 KIH 1 23.800 16.530 22.800 1.00 0.00

ATOM 101 C63 KIH 1 23.620 16.230 24.120 1.00 0.00

ATOM 102 C64 KIH 1 23.660 17.120 25.170 1.00 0.00

ATOM 103 C65 KIH 1 23.400 16.760 26.470 1.00 0.00

ATOM 104 C66 KIH 1 23.200 15.440 26.780 1.00 0.00

ATOM 105 C67 KIH 1 23.260 14.520 25.760 1.00 0.00

ATOM 106 C68 KIH 1 23.450 14.900 24.460 1.00 0.00

ATOM 107 C69 KIH 1 23.260 13.950 23.490 1.00 0.00

ATOM 108 C70 KIH 1 23.390 14.270 22.170 1.00 0.00

ATOM 109 C71 KIH 1 23.640 15.570 21.820 1.00 0.00

ATOM 110 C72 KIH 1 23.910 15.880 20.510 1.00 0.00

ATOM 111 C73 KIH 1 24.180 17.190 20.180 1.00 0.00

ATOM 112 H29 KIH 1 26.510 19.780 21.520 1.00 0.00

ATOM 113 H30 KIH 1 27.900 21.700 21.690 1.00 0.00

ATOM 114 H31 KIH 1 28.310 23.940 21.060 1.00 0.00

ATOM 115 H32 KIH 1 26.760 25.140 19.620 1.00 0.00

ATOM 116 H33 KIH 1 24.730 24.120 18.850 1.00 0.00

ATOM 117 H34 KIH 1 24.230 18.600 23.080 1.00 0.00

ATOM 118 H35 KIH 1 23.920 18.150 24.960 1.00 0.00

ATOM 119 H36 KIH 1 23.470 17.520 27.240 1.00 0.00

ATOM 120 H37 KIH 1 23.040 15.170 27.820 1.00 0.00

ATOM 121 H38 KIH 1 23.080 13.480 26.020 1.00 0.00

ATOM 122 H39 KIH 1 22.980 12.930 23.780 1.00 0.00

ATOM 123 H40 KIH 1 23.110 13.580 21.380 1.00 0.00

ATOM 124 H41 KIH 1 23.880 15.140 19.720 1.00 0.00

ATOM 125 H42 KIH 1 24.230 17.420 19.110 1.00 0.00

ATOM 126 O5 KIH 1 22.430 19.780 19.650 1.00 0.00

ATOM 127 O6 KIH 1 21.940 19.460 24.020 1.00 0.00

ATOM 128 C74 KIH 1 22.950 22.370 18.570 1.00 0.00

ATOM 129 C75 KIH 1 21.750 22.700 19.160 1.00 0.00

ATOM 130 C76 KIH 1 20.810 23.340 18.390 1.00 0.00

ATOM 131 C77 KIH 1 20.980 23.540 17.030 1.00 0.00

ATOM 132 C78 KIH 1 22.190 23.230 16.440 1.00 0.00

ATOM 133 C79 KIH 1 23.130 22.610 17.230 1.00 0.00

ATOM 134 C80 KIH 1 22.390 23.470 15.110 1.00 0.00

ATOM 135 C81 KIH 1 23.580 23.120 14.500 1.00 0.00

ATOM 136 C82 KIH 1 24.510 22.440 15.250 1.00 0.00

ATOM 137 C83 KIH 1 24.290 22.230 16.600 1.00 0.00

ATOM 138 C84 KIH 1 19.450 23.670 18.880 1.00 0.00

ATOM 139 C85 KIH 1 19.290 24.520 19.960 1.00 0.00

ATOM 140 C86 KIH 1 18.080 24.930 20.450 1.00 0.00

ATOM 141 C87 KIH 1 18.010 25.660 21.610 1.00 0.00

ATOM 142 C88 KIH 1 19.060 26.010 22.410 1.00 0.00

ATOM 143 C89 KIH 1 18.930 26.770 23.550 1.00 0.00

ATOM 144 C90 KIH 1 17.680 27.160 23.940 1.00 0.00

ATOM 145 C91 KIH 1 16.590 26.800 23.170 1.00 0.00

ATOM 146 C92 KIH 1 16.760 25.980 22.090 1.00 0.00

ATOM 147 C93 KIH 1 15.640 25.600 21.380 1.00 0.00

ATOM 148 C94 KIH 1 15.750 24.810 20.250 1.00 0.00

ATOM 149 C95 KIH 1 16.980 24.570 19.700 1.00 0.00

ATOM 150 C96 KIH 1 17.100 23.810 18.560 1.00 0.00

ATOM 151 C97 KIH 1 18.320 23.310 18.180 1.00 0.00

ATOM 152 H43 KIH 1 20.200 24.030 16.480 1.00 0.00

ATOM 153 H44 KIH 1 21.690 23.970 14.470 1.00 0.00

ATOM 154 H45 KIH 1 23.750 23.310 13.450 1.00 0.00

ATOM 155 H46 KIH 1 25.410 22.140 14.750 1.00 0.00

ATOM 156 H47 KIH 1 25.110 21.760 17.120 1.00 0.00

ATOM 157 H48 KIH 1 20.220 24.870 20.390 1.00 0.00

ATOM 158 H49 KIH 1 20.070 25.860 22.040 1.00 0.00

ATOM 159 H50 KIH 1 19.850 27.100 24.030 1.00 0.00

ATOM 160 H51 KIH 1 17.540 27.750 24.830 1.00 0.00

ATOM 161 H52 KIH 1 15.630 27.180 23.500 1.00 0.00

ATOM 162 H53 KIH 1 14.630 25.800 21.720 1.00 0.00

ATOM 163 H54 KIH 1 14.840 24.520 19.730 1.00 0.00

ATOM 164 H55 KIH 1 16.190 23.470 18.090 1.00 0.00

ATOM 165 H56 KIH 1 18.300 22.740 17.270 1.00 0.00

ATOM 166 O7 KIH 1 21.250 22.030 20.260 1.00 0.00

ATOM 167 O8 KIH 1 22.920 21.290 22.600 1.00 0.00

ATOM 168 C98 KIH 1 20.670 21.760 23.910 1.00 0.00

ATOM 169 F4 KIH 1 19.410 21.300 23.960 1.00 0.00

ATOM 170 F5 KIH 1 21.010 22.220 25.120 1.00 0.00

ATOM 171 F6 KIH 1 20.630 22.870 23.160 1.00 0.00

ATOM 172 N2 KIH 1 21.010 19.990 21.810 1.00 0.00

ATOM 173 P2 KIH 1 21.110 20.450 20.230 1.00 0.00

ATOM 174 S2 KIH 1 21.770 20.580 23.140 1.00 0.00

ATOM 175 N3 KIH 1 19.900 19.910 19.530 1.00 0.00

ATOM 176 H57 KIH 1 20.580 19.070 21.890 1.00 0.00

TER

ENDMDL

REMARK GENERATED BY TRJCONV

TITLE KIH t= 6500.00000

REMARK THIS IS A SIMULATION BOX

CRYST1 38.311 38.311 38.311 90.00 90.00 90.00 P 1 1

MODEL 14

ATOM 1 C1 KIH 1 16.230 19.810 17.120 1.00 0.00

ATOM 2 C2 KIH 1 16.290 20.130 18.450 1.00 0.00

ATOM 3 C3 KIH 1 15.230 19.990 19.330 1.00 0.00

ATOM 4 C4 KIH 1 14.070 19.560 18.720 1.00 0.00

ATOM 5 C5 KIH 1 13.910 19.340 17.370 1.00 0.00

ATOM 6 C6 KIH 1 15.050 19.310 16.600 1.00 0.00

ATOM 7 C7 KIH 1 12.690 18.950 16.880 1.00 0.00

ATOM 8 C8 KIH 1 12.590 18.670 15.540 1.00 0.00

ATOM 9 C9 KIH 1 13.720 18.560 14.760 1.00 0.00

ATOM 10 C10 KIH 1 14.940 18.870 15.310 1.00 0.00

ATOM 11 C11 KIH 1 15.340 20.190 20.800 1.00 0.00

ATOM 12 C12 KIH 1 15.120 19.150 21.670 1.00 0.00

ATOM 13 C13 KIH 1 15.350 19.160 23.030 1.00 0.00

ATOM 14 C14 KIH 1 15.150 18.120 23.910 1.00 0.00

ATOM 15 C15 KIH 1 14.560 16.970 23.490 1.00 0.00

ATOM 16 C16 KIH 1 14.280 15.950 24.370 1.00 0.00

ATOM 17 C17 KIH 1 14.590 16.150 25.700 1.00 0.00

ATOM 18 C18 KIH 1 15.160 17.320 26.150 1.00 0.00

ATOM 19 C19 KIH 1 15.400 18.320 25.250 1.00 0.00

ATOM 20 C20 KIH 1 15.900 19.490 25.770 1.00 0.00

ATOM 21 C21 KIH 1 16.020 20.520 24.860 1.00 0.00

ATOM 22 C22 KIH 1 15.790 20.360 23.520 1.00 0.00

ATOM 23 C23 KIH 1 16.020 21.430 22.680 1.00 0.00

ATOM 24 C24 KIH 1 15.820 21.370 21.310 1.00 0.00

ATOM 25 H1 KIH 1 13.230 19.540 19.390 1.00 0.00

ATOM 26 H2 KIH 1 11.770 18.910 17.450 1.00 0.00

ATOM 27 H3 KIH 1 11.600 18.430 15.180 1.00 0.00

ATOM 28 H4 KIH 1 13.670 18.440 13.690 1.00 0.00

ATOM 29 H5 KIH 1 15.780 18.810 14.620 1.00 0.00

ATOM 30 H6 KIH 1 14.730 18.250 21.210 1.00 0.00

ATOM 31 H7 KIH 1 14.360 16.790 22.440 1.00 0.00

ATOM 32 H8 KIH 1 13.780 15.060 24.010 1.00 0.00

ATOM 33 H9 KIH 1 14.370 15.380 26.430 1.00 0.00

ATOM 34 H10 KIH 1 15.490 17.330 27.180 1.00 0.00

ATOM 35 H11 KIH 1 16.110 19.620 26.820 1.00 0.00

ATOM 36 H12 KIH 1 16.160 21.490 25.330 1.00 0.00

ATOM 37 H13 KIH 1 16.270 22.390 23.070 1.00 0.00

ATOM 38 H14 KIH 1 16.010 22.170 20.620 1.00 0.00

ATOM 39 O1 KIH 1 17.520 20.400 19.020 1.00 0.00

ATOM 40 O2 KIH 1 19.210 18.400 22.730 1.00 0.00

ATOM 41 C25 KIH 1 17.410 20.150 16.280 1.00 0.00

ATOM 42 C26 KIH 1 18.500 19.330 16.470 1.00 0.00

ATOM 43 C27 KIH 1 19.400 19.210 15.450 1.00 0.00

ATOM 44 C28 KIH 1 19.350 20.100 14.400 1.00 0.00

ATOM 45 C29 KIH 1 18.380 21.070 14.300 1.00 0.00

ATOM 46 C30 KIH 1 17.380 21.050 15.240 1.00 0.00

ATOM 47 C31 KIH 1 18.410 22.030 13.310 1.00 0.00

ATOM 48 C32 KIH 1 17.370 22.930 13.240 1.00 0.00

ATOM 49 C33 KIH 1 16.320 22.880 14.140 1.00 0.00

ATOM 50 C34 KIH 1 16.340 21.950 15.130 1.00 0.00

ATOM 51 C35 KIH 1 20.530 18.230 15.530 1.00 0.00

ATOM 52 C36 KIH 1 20.380 16.920 15.150 1.00 0.00

ATOM 53 C37 KIH 1 21.460 16.060 15.130 1.00 0.00

ATOM 54 C38 KIH 1 21.440 14.750 14.730 1.00 0.00

ATOM 55 C39 KIH 1 20.360 14.150 14.100 1.00 0.00

ATOM 56 C40 KIH 1 20.420 12.860 13.650 1.00 0.00

ATOM 57 C41 KIH 1 21.520 12.080 13.880 1.00 0.00

ATOM 58 C42 KIH 1 22.610 12.650 14.500 1.00 0.00

ATOM 59 C43 KIH 1 22.560 13.960 14.890 1.00 0.00

ATOM 60 C44 KIH 1 23.720 14.480 15.400 1.00 0.00

ATOM 61 C45 KIH 1 23.790 15.820 15.720 1.00 0.00

ATOM 62 C46 KIH 1 22.650 16.570 15.590 1.00 0.00

ATOM 63 C47 KIH 1 22.830 17.900 15.890 1.00 0.00

ATOM 64 C48 KIH 1 21.750 18.760 15.870 1.00 0.00

ATOM 65 H15 KIH 1 20.140 20.110 13.680 1.00 0.00

ATOM 66 H16 KIH 1 19.240 22.090 12.610 1.00 0.00

ATOM 67 H17 KIH 1 17.330 23.690 12.470 1.00 0.00

ATOM 68 H18 KIH 1 15.580 23.680 14.070 1.00 0.00

ATOM 69 H19 KIH 1 15.660 22.100 15.960 1.00 0.00

ATOM 70 H20 KIH 1 19.420 16.520 14.880 1.00 0.00

ATOM 71 H21 KIH 1 19.440 14.630 13.820 1.00 0.00

ATOM 72 H22 KIH 1 19.600 12.510 13.030 1.00 0.00

ATOM 73 H23 KIH 1 21.580 11.090 13.430 1.00 0.00

ATOM 74 H24 KIH 1 23.510 12.060 14.630 1.00 0.00

ATOM 75 H25 KIH 1 24.540 13.820 15.610 1.00 0.00

ATOM 76 H26 KIH 1 24.740 16.280 15.960 1.00 0.00

ATOM 77 H27 KIH 1 23.710 18.440 16.210 1.00 0.00

ATOM 78 H28 KIH 1 21.980 19.790 16.080 1.00 0.00

ATOM 79 O3 KIH 1 18.710 18.660 17.660 1.00 0.00

ATOM 80 O4 KIH 1 17.480 16.700 22.520 1.00 0.00

ATOM 81 C49 KIH 1 19.850 16.270 21.560 1.00 0.00

ATOM 82 F1 KIH 1 20.930 16.920 21.140 1.00 0.00

ATOM 83 F2 KIH 1 20.260 15.600 22.650 1.00 0.00

ATOM 84 F3 KIH 1 19.450 15.360 20.660 1.00 0.00

ATOM 85 N1 KIH 1 18.080 18.100 20.390 1.00 0.00

ATOM 86 P1 KIH 1 18.600 19.240 19.120 1.00 0.00

ATOM 87 S1 KIH 1 18.550 17.440 21.890 1.00 0.00

ATOM 88 C50 KIH 1 23.880 21.540 19.210 1.00 0.00

ATOM 89 C51 KIH 1 23.580 20.300 19.700 1.00 0.00

ATOM 90 C52 KIH 1 24.510 19.610 20.450 1.00 0.00

ATOM 91 C53 KIH 1 25.670 20.280 20.800 1.00 0.00

ATOM 92 C54 KIH 1 25.920 21.570 20.390 1.00 0.00

ATOM 93 C55 KIH 1 25.090 22.160 19.460 1.00 0.00

ATOM 94 C56 KIH 1 27.110 22.190 20.720 1.00 0.00

ATOM 95 C57 KIH 1 27.400 23.420 20.200 1.00 0.00

ATOM 96 C58 KIH 1 26.520 24.080 19.390 1.00 0.00

ATOM 97 C59 KIH 1 25.400 23.390 18.950 1.00 0.00

ATOM 98 C60 KIH 1 24.170 18.280 21.000 1.00 0.00

ATOM 99 C61 KIH 1 24.220 17.860 22.320 1.00 0.00

ATOM 100 C62 KIH 1 23.890 16.580 22.680 1.00 0.00

ATOM 101 C63 KIH 1 23.890 16.100 23.970 1.00 0.00

ATOM 102 C64 KIH 1 24.000 16.920 25.060 1.00 0.00

ATOM 103 C65 KIH 1 23.950 16.430 26.340 1.00 0.00

ATOM 104 C66 KIH 1 23.980 15.090 26.570 1.00 0.00

ATOM 105 C67 KIH 1 23.860 14.240 25.490 1.00 0.00

ATOM 106 C68 KIH 1 23.760 14.740 24.210 1.00 0.00

ATOM 107 C69 KIH 1 23.660 13.840 23.170 1.00 0.00

ATOM 108 C70 KIH 1 23.480 14.300 21.880 1.00 0.00

ATOM 109 C71 KIH 1 23.710 15.640 21.690 1.00 0.00

ATOM 110 C72 KIH 1 23.600 16.060 20.380 1.00 0.00

ATOM 111 C73 KIH 1 23.830 17.360 20.030 1.00 0.00

ATOM 112 H29 KIH 1 26.460 19.840 21.390 1.00 0.00

ATOM 113 H30 KIH 1 27.840 21.650 21.320 1.00 0.00

ATOM 114 H31 KIH 1 28.370 23.900 20.260 1.00 0.00

ATOM 115 H32 KIH 1 26.580 25.100 19.020 1.00 0.00

ATOM 116 H33 KIH 1 24.760 23.920 18.260 1.00 0.00

ATOM 117 H34 KIH 1 24.460 18.640 23.030 1.00 0.00

ATOM 118 H35 KIH 1 23.990 18.010 24.990 1.00 0.00

ATOM 119 H36 KIH 1 23.970 17.110 27.190 1.00 0.00

ATOM 120 H37 KIH 1 23.910 14.680 27.570 1.00 0.00

ATOM 121 H38 KIH 1 23.750 13.190 25.700 1.00 0.00

ATOM 122 H39 KIH 1 23.700 12.790 23.390 1.00 0.00

ATOM 123 H40 KIH 1 23.210 13.610 21.100 1.00 0.00

ATOM 124 H41 KIH 1 23.450 15.380 19.550 1.00 0.00

ATOM 125 H42 KIH 1 23.960 17.540 18.970 1.00 0.00

ATOM 126 O5 KIH 1 22.340 19.720 19.550 1.00 0.00

ATOM 127 O6 KIH 1 22.080 19.320 23.880 1.00 0.00

ATOM 128 C74 KIH 1 22.880 22.160 18.290 1.00 0.00

ATOM 129 C75 KIH 1 21.620 22.480 18.750 1.00 0.00

ATOM 130 C76 KIH 1 20.740 23.230 18.010 1.00 0.00

ATOM 131 C77 KIH 1 21.060 23.560 16.710 1.00 0.00

ATOM 132 C78 KIH 1 22.240 23.110 16.190 1.00 0.00

ATOM 133 C79 KIH 1 23.130 22.400 16.960 1.00 0.00

ATOM 134 C80 KIH 1 22.520 23.410 14.870 1.00 0.00

ATOM 135 C81 KIH 1 23.760 23.120 14.360 1.00 0.00

ATOM 136 C82 KIH 1 24.640 22.310 15.070 1.00 0.00

ATOM 137 C83 KIH 1 24.280 21.960 16.340 1.00 0.00

ATOM 138 C84 KIH 1 19.470 23.770 18.580 1.00 0.00

ATOM 139 C85 KIH 1 19.540 24.870 19.400 1.00 0.00

ATOM 140 C86 KIH 1 18.360 25.420 19.830 1.00 0.00

ATOM 141 C87 KIH 1 18.490 26.590 20.550 1.00 0.00

ATOM 142 C88 KIH 1 19.660 27.170 20.960 1.00 0.00

ATOM 143 C89 KIH 1 19.660 28.270 21.790 1.00 0.00

ATOM 144 C90 KIH 1 18.480 28.930 22.050 1.00 0.00

ATOM 145 C91 KIH 1 17.300 28.330 21.680 1.00 0.00

ATOM 146 C92 KIH 1 17.300 27.200 20.900 1.00 0.00

ATOM 147 C93 KIH 1 16.060 26.740 20.500 1.00 0.00

ATOM 148 C94 KIH 1 15.950 25.510 19.900 1.00 0.00

ATOM 149 C95 KIH 1 17.120 24.940 19.470 1.00 0.00

ATOM 150 C96 KIH 1 17.110 23.840 18.640 1.00 0.00

ATOM 151 C97 KIH 1 18.260 23.230 18.200 1.00 0.00

ATOM 152 H43 KIH 1 20.330 24.210 16.240 1.00 0.00

ATOM 153 H44 KIH 1 21.820 23.870 14.190 1.00 0.00

ATOM 154 H45 KIH 1 24.010 23.500 13.380 1.00 0.00

ATOM 155 H46 KIH 1 25.570 22.100 14.560 1.00 0.00

ATOM 156 H47 KIH 1 24.990 21.480 17.000 1.00 0.00

ATOM 157 H48 KIH 1 20.570 25.100 19.620 1.00 0.00

ATOM 158 H49 KIH 1 20.580 26.630 20.760 1.00 0.00

ATOM 159 H50 KIH 1 20.580 28.700 22.170 1.00 0.00

ATOM 160 H51 KIH 1 18.450 29.870 22.560 1.00 0.00

ATOM 161 H52 KIH 1 16.350 28.720 22.000 1.00 0.00

ATOM 162 H53 KIH 1 15.160 27.250 20.790 1.00 0.00

ATOM 163 H54 KIH 1 15.010 25.160 19.490 1.00 0.00

ATOM 164 H55 KIH 1 16.170 23.430 18.300 1.00 0.00

ATOM 165 H56 KIH 1 18.280 22.350 17.580 1.00 0.00

ATOM 166 O7 KIH 1 21.190 22.020 19.970 1.00 0.00

ATOM 167 O8 KIH 1 22.730 21.480 22.890 1.00 0.00

ATOM 168 C98 KIH 1 20.390 21.350 24.060 1.00 0.00

ATOM 169 F4 KIH 1 19.240 20.670 24.140 1.00 0.00

ATOM 170 F5 KIH 1 20.700 21.890 25.250 1.00 0.00

ATOM 171 F6 KIH 1 19.990 22.410 23.340 1.00 0.00

ATOM 172 N2 KIH 1 21.090 19.970 21.760 1.00 0.00

ATOM 173 P2 KIH 1 21.100 20.460 20.190 1.00 0.00

ATOM 174 S2 KIH 1 21.710 20.520 23.190 1.00 0.00

ATOM 175 N3 KIH 1 19.880 19.940 19.480 1.00 0.00

ATOM 176 H57 KIH 1 20.630 19.080 21.960 1.00 0.00

TER

ENDMDL

REMARK GENERATED BY TRJCONV

TITLE KIH t= 7000.00000

REMARK THIS IS A SIMULATION BOX

CRYST1 38.584 38.584 38.584 90.00 90.00 90.00 P 1 1

MODEL 15

ATOM 1 C1 KIH 1 16.560 19.890 16.770 1.00 0.00

ATOM 2 C2 KIH 1 16.440 20.110 18.120 1.00 0.00

ATOM 3 C3 KIH 1 15.200 20.110 18.710 1.00 0.00

ATOM 4 C4 KIH 1 14.020 19.840 18.040 1.00 0.00

ATOM 5 C5 KIH 1 14.210 19.660 16.690 1.00 0.00

ATOM 6 C6 KIH 1 15.430 19.540 16.070 1.00 0.00

ATOM 7 C7 KIH 1 13.060 19.470 15.950 1.00 0.00

ATOM 8 C8 KIH 1 13.050 19.090 14.640 1.00 0.00

ATOM 9 C9 KIH 1 14.300 18.840 14.110 1.00 0.00

ATOM 10 C10 KIH 1 15.480 19.130 14.760 1.00 0.00

ATOM 11 C11 KIH 1 15.040 20.180 20.200 1.00 0.00

ATOM 12 C12 KIH 1 14.730 19.140 21.040 1.00 0.00

ATOM 13 C13 KIH 1 14.740 19.200 22.420 1.00 0.00

ATOM 14 C14 KIH 1 14.390 18.160 23.240 1.00 0.00

ATOM 15 C15 KIH 1 14.150 16.900 22.740 1.00 0.00

ATOM 16 C16 KIH 1 13.970 15.810 23.570 1.00 0.00

ATOM 17 C17 KIH 1 13.930 16.020 24.940 1.00 0.00

ATOM 18 C18 KIH 1 14.080 17.270 25.480 1.00 0.00

ATOM 19 C19 KIH 1 14.340 18.300 24.610 1.00 0.00

ATOM 20 C20 KIH 1 14.630 19.540 25.150 1.00 0.00

ATOM 21 C21 KIH 1 15.140 20.530 24.360 1.00 0.00

ATOM 22 C22 KIH 1 15.080 20.400 22.990 1.00 0.00

ATOM 23 C23 KIH 1 15.360 21.480 22.170 1.00 0.00

ATOM 24 C24 KIH 1 15.440 21.340 20.800 1.00 0.00

ATOM 25 H1 KIH 1 13.110 19.980 18.600 1.00 0.00

ATOM 26 H2 KIH 1 12.130 19.580 16.500 1.00 0.00

ATOM 27 H3 KIH 1 12.190 18.920 14.020 1.00 0.00

ATOM 28 H4 KIH 1 14.430 18.350 13.160 1.00 0.00

ATOM 29 H5 KIH 1 16.410 18.920 14.260 1.00 0.00

ATOM 30 H6 KIH 1 14.490 18.210 20.560 1.00 0.00

ATOM 31 H7 KIH 1 14.250 16.650 21.690 1.00 0.00

ATOM 32 H8 KIH 1 13.910 14.810 23.190 1.00 0.00

ATOM 33 H9 KIH 1 13.640 15.220 25.620 1.00 0.00

ATOM 34 H10 KIH 1 14.020 17.480 26.530 1.00 0.00

ATOM 35 H11 KIH 1 14.780 19.640 26.220 1.00 0.00

ATOM 36 H12 KIH 1 15.210 21.520 24.790 1.00 0.00

ATOM 37 H13 KIH 1 15.380 22.470 22.580 1.00 0.00

ATOM 38 H14 KIH 1 15.630 22.240 20.240 1.00 0.00

ATOM 39 O1 KIH 1 17.540 20.370 18.900 1.00 0.00

ATOM 40 O2 KIH 1 19.260 18.350 22.540 1.00 0.00

ATOM 41 C25 KIH 1 17.890 20.060 16.130 1.00 0.00

ATOM 42 C26 KIH 1 18.930 19.260 16.550 1.00 0.00

ATOM 43 C27 KIH 1 20.090 19.150 15.810 1.00 0.00

ATOM 44 C28 KIH 1 20.330 20.100 14.850 1.00 0.00

ATOM 45 C29 KIH 1 19.390 21.060 14.530 1.00 0.00

ATOM 46 C30 KIH 1 18.200 21.060 15.220 1.00 0.00

ATOM 47 C31 KIH 1 19.660 21.960 13.530 1.00 0.00

ATOM 48 C32 KIH 1 18.770 22.990 13.290 1.00 0.00

ATOM 49 C33 KIH 1 17.570 23.070 13.970 1.00 0.00

ATOM 50 C34 KIH 1 17.330 22.080 14.880 1.00 0.00

ATOM 51 C35 KIH 1 21.080 18.090 16.150 1.00 0.00

ATOM 52 C36 KIH 1 20.710 16.770 16.270 1.00 0.00

ATOM 53 C37 KIH 1 21.640 15.760 16.340 1.00 0.00

ATOM 54 C38 KIH 1 21.310 14.420 16.460 1.00 0.00

ATOM 55 C39 KIH 1 20.040 13.920 16.440 1.00 0.00

ATOM 56 C40 KIH 1 19.730 12.580 16.500 1.00 0.00

ATOM 57 C41 KIH 1 20.730 11.660 16.730 1.00 0.00

ATOM 58 C42 KIH 1 21.990 12.190 16.830 1.00 0.00

ATOM 59 C43 KIH 1 22.320 13.510 16.640 1.00 0.00

ATOM 60 C44 KIH 1 23.630 13.890 16.740 1.00 0.00

ATOM 61 C45 KIH 1 23.930 15.230 16.640 1.00 0.00

ATOM 62 C46 KIH 1 22.950 16.170 16.450 1.00 0.00

ATOM 63 C47 KIH 1 23.340 17.490 16.320 1.00 0.00

ATOM 64 C48 KIH 1 22.390 18.480 16.270 1.00 0.00

ATOM 65 H15 KIH 1 21.220 20.100 14.250 1.00 0.00

ATOM 66 H16 KIH 1 20.610 21.960 13.020 1.00 0.00

ATOM 67 H17 KIH 1 18.940 23.620 12.440 1.00 0.00

ATOM 68 H18 KIH 1 16.780 23.750 13.690 1.00 0.00

ATOM 69 H19 KIH 1 16.370 22.110 15.370 1.00 0.00

ATOM 70 H20 KIH 1 19.640 16.620 16.220 1.00 0.00

ATOM 71 H21 KIH 1 19.280 14.650 16.210 1.00 0.00

ATOM 72 H22 KIH 1 18.710 12.290 16.280 1.00 0.00

ATOM 73 H23 KIH 1 20.510 10.610 16.660 1.00 0.00

ATOM 74 H24 KIH 1 22.830 11.530 17.030 1.00 0.00

ATOM 75 H25 KIH 1 24.420 13.230 17.070 1.00 0.00

ATOM 76 H26 KIH 1 24.980 15.470 16.660 1.00 0.00

ATOM 77 H27 KIH 1 24.360 17.840 16.290 1.00 0.00

ATOM 78 H28 KIH 1 22.890 19.440 16.340 1.00 0.00

ATOM 79 O3 KIH 1 18.800 18.510 17.690 1.00 0.00

ATOM 80 O4 KIH 1 17.320 16.960 22.590 1.00 0.00

ATOM 81 C49 KIH 1 19.510 15.970 21.480 1.00 0.00

ATOM 82 F1 KIH 1 18.800 14.990 20.910 1.00 0.00

ATOM 83 F2 KIH 1 20.440 16.250 20.550 1.00 0.00

ATOM 84 F3 KIH 1 20.150 15.410 22.510 1.00 0.00

ATOM 85 N1 KIH 1 17.990 18.100 20.360 1.00 0.00

ATOM 86 P1 KIH 1 18.600 19.210 19.100 1.00 0.00

ATOM 87 S1 KIH 1 18.470 17.380 21.830 1.00 0.00

ATOM 88 C50 KIH 1 23.820 21.720 19.160 1.00 0.00

ATOM 89 C51 KIH 1 23.550 20.530 19.790 1.00 0.00

ATOM 90 C52 KIH 1 24.550 19.870 20.460 1.00 0.00

ATOM 91 C53 KIH 1 25.710 20.550 20.760 1.00 0.00

ATOM 92 C54 KIH 1 25.870 21.850 20.370 1.00 0.00

ATOM 93 C55 KIH 1 24.980 22.380 19.460 1.00 0.00

ATOM 94 C56 KIH 1 26.870 22.620 20.910 1.00 0.00

ATOM 95 C57 KIH 1 27.050 23.930 20.510 1.00 0.00

ATOM 96 C58 KIH 1 26.190 24.490 19.590 1.00 0.00

ATOM 97 C59 KIH 1 25.160 23.700 19.130 1.00 0.00

ATOM 98 C60 KIH 1 24.340 18.470 20.920 1.00 0.00

ATOM 99 C61 KIH 1 24.300 18.170 22.260 1.00 0.00

ATOM 100 C62 KIH 1 23.990 16.930 22.760 1.00 0.00

ATOM 101 C63 KIH 1 23.940 16.670 24.120 1.00 0.00

ATOM 102 C64 KIH 1 24.140 17.660 25.040 1.00 0.00

ATOM 103 C65 KIH 1 24.110 17.360 26.380 1.00 0.00

ATOM 104 C66 KIH 1 23.870 16.080 26.820 1.00 0.00

ATOM 105 C67 KIH 1 23.650 15.090 25.900 1.00 0.00

ATOM 106 C68 KIH 1 23.660 15.400 24.560 1.00 0.00

ATOM 107 C69 KIH 1 23.540 14.400 23.630 1.00 0.00

ATOM 108 C70 KIH 1 23.520 14.650 22.280 1.00 0.00

ATOM 109 C71 KIH 1 23.810 15.930 21.840 1.00 0.00

ATOM 110 C72 KIH 1 23.870 16.160 20.490 1.00 0.00

ATOM 111 C73 KIH 1 24.230 17.410 20.050 1.00 0.00

ATOM 112 H29 KIH 1 26.480 20.080 21.360 1.00 0.00

ATOM 113 H30 KIH 1 27.510 22.100 21.600 1.00 0.00

ATOM 114 H31 KIH 1 27.910 24.380 20.970 1.00 0.00

ATOM 115 H32 KIH 1 26.260 25.560 19.510 1.00 0.00

ATOM 116 H33 KIH 1 24.410 24.170 18.500 1.00 0.00

ATOM 117 H34 KIH 1 24.360 19.050 22.890 1.00 0.00

ATOM 118 H35 KIH 1 24.380 18.690 24.810 1.00 0.00

ATOM 119 H36 KIH 1 24.360 18.130 27.090 1.00 0.00

ATOM 120 H37 KIH 1 23.680 15.850 27.870 1.00 0.00

ATOM 121 H38 KIH 1 23.380 14.100 26.240 1.00 0.00

ATOM 122 H39 KIH 1 23.380 13.360 23.880 1.00 0.00

ATOM 123 H40 KIH 1 23.360 13.850 21.570 1.00 0.00

ATOM 124 H41 KIH 1 23.550 15.410 19.780 1.00 0.00

ATOM 125 H42 KIH 1 24.120 17.710 19.020 1.00 0.00

ATOM 126 O5 KIH 1 22.370 19.840 19.570 1.00 0.00

ATOM 127 O6 KIH 1 21.920 19.490 24.110 1.00 0.00

ATOM 128 C74 KIH 1 22.760 22.280 18.280 1.00 0.00

ATOM 129 C75 KIH 1 21.530 22.550 18.810 1.00 0.00

ATOM 130 C76 KIH 1 20.610 23.370 18.200 1.00 0.00

ATOM 131 C77 KIH 1 20.870 23.810 16.930 1.00 0.00

ATOM 132 C78 KIH 1 22.040 23.470 16.300 1.00 0.00

ATOM 133 C79 KIH 1 22.990 22.750 17.000 1.00 0.00

ATOM 134 C80 KIH 1 22.210 23.680 14.950 1.00 0.00

ATOM 135 C81 KIH 1 23.350 23.240 14.340 1.00 0.00

ATOM 136 C82 KIH 1 24.310 22.490 14.980 1.00 0.00

ATOM 137 C83 KIH 1 24.110 22.310 16.330 1.00 0.00

ATOM 138 C84 KIH 1 19.390 23.890 18.870 1.00 0.00

ATOM 139 C85 KIH 1 19.410 24.720 19.980 1.00 0.00

ATOM 140 C86 KIH 1 18.280 25.290 20.510 1.00 0.00

ATOM 141 C87 KIH 1 18.260 26.070 21.640 1.00 0.00

ATOM 142 C88 KIH 1 19.420 26.370 22.310 1.00 0.00

ATOM 143 C89 KIH 1 19.450 27.160 23.430 1.00 0.00

ATOM 144 C90 KIH 1 18.280 27.740 23.840 1.00 0.00

ATOM 145 C91 KIH 1 17.090 27.470 23.190 1.00 0.00

ATOM 146 C92 KIH 1 17.090 26.540 22.170 1.00 0.00

ATOM 147 C93 KIH 1 15.890 26.250 21.580 1.00 0.00

ATOM 148 C94 KIH 1 15.910 25.420 20.490 1.00 0.00

ATOM 149 C95 KIH 1 17.080 24.950 19.920 1.00 0.00

ATOM 150 C96 KIH 1 17.020 24.120 18.830 1.00 0.00

ATOM 151 C97 KIH 1 18.170 23.550 18.330 1.00 0.00

ATOM 152 H43 KIH 1 20.120 24.380 16.390 1.00 0.00

ATOM 153 H44 KIH 1 21.450 24.160 14.360 1.00 0.00

ATOM 154 H45 KIH 1 23.500 23.530 13.300 1.00 0.00

ATOM 155 H46 KIH 1 25.260 22.370 14.460 1.00 0.00

ATOM 156 H47 KIH 1 24.870 21.750 16.830 1.00 0.00

ATOM 157 H48 KIH 1 20.410 24.760 20.400 1.00 0.00

ATOM 158 H49 KIH 1 20.420 26.080 22.000 1.00 0.00

ATOM 159 H50 KIH 1 20.320 27.460 23.990 1.00 0.00

ATOM 160 H51 KIH 1 18.240 28.480 24.620 1.00 0.00

ATOM 161 H52 KIH 1 16.210 28.030 23.460 1.00 0.00

ATOM 162 H53 KIH 1 14.900 26.550 21.930 1.00 0.00

ATOM 163 H54 KIH 1 14.960 25.230 20.040 1.00 0.00

ATOM 164 H55 KIH 1 16.040 23.840 18.500 1.00 0.00

ATOM 165 H56 KIH 1 18.100 22.870 17.500 1.00 0.00

ATOM 166 O7 KIH 1 21.190 22.020 20.050 1.00 0.00

ATOM 167 O8 KIH 1 23.010 21.350 22.890 1.00 0.00

ATOM 168 C98 KIH 1 20.630 21.680 24.010 1.00 0.00

ATOM 169 F4 KIH 1 21.070 22.350 25.080 1.00 0.00

ATOM 170 F5 KIH 1 20.210 22.650 23.170 1.00 0.00

ATOM 171 F6 KIH 1 19.510 21.090 24.450 1.00 0.00

ATOM 172 N2 KIH 1 21.170 20.050 21.810 1.00 0.00

ATOM 173 P2 KIH 1 21.070 20.460 20.210 1.00 0.00

ATOM 174 S2 KIH 1 21.820 20.600 23.220 1.00 0.00

ATOM 175 N3 KIH 1 19.870 19.890 19.530 1.00 0.00

ATOM 176 H57 KIH 1 20.720 19.150 21.960 1.00 0.00

TER

ENDMDL

REMARK GENERATED BY TRJCONV

TITLE KIH t= 7500.00000

REMARK THIS IS A SIMULATION BOX

CRYST1 38.785 38.785 38.785 90.00 90.00 90.00 P 1 1

MODEL 16

ATOM 1 C1 KIH 1 16.230 19.740 17.120 1.00 0.00

ATOM 2 C2 KIH 1 16.400 20.140 18.430 1.00 0.00

ATOM 3 C3 KIH 1 15.300 20.250 19.250 1.00 0.00

ATOM 4 C4 KIH 1 14.060 19.830 18.840 1.00 0.00

ATOM 5 C5 KIH 1 13.860 19.550 17.510 1.00 0.00

ATOM 6 C6 KIH 1 14.950 19.450 16.670 1.00 0.00

ATOM 7 C7 KIH 1 12.640 19.140 17.030 1.00 0.00

ATOM 8 C8 KIH 1 12.470 18.610 15.770 1.00 0.00

ATOM 9 C9 KIH 1 13.540 18.670 14.900 1.00 0.00

ATOM 10 C10 KIH 1 14.770 19.060 15.370 1.00 0.00

ATOM 11 C11 KIH 1 15.400 20.610 20.700 1.00 0.00

ATOM 12 C12 KIH 1 15.120 19.680 21.680 1.00 0.00

ATOM 13 C13 KIH 1 15.160 19.890 23.040 1.00 0.00

ATOM 14 C14 KIH 1 14.690 19.020 23.990 1.00 0.00

ATOM 15 C15 KIH 1 14.310 17.720 23.710 1.00 0.00

ATOM 16 C16 KIH 1 13.990 16.820 24.700 1.00 0.00

ATOM 17 C17 KIH 1 14.030 17.270 26.010 1.00 0.00

ATOM 18 C18 KIH 1 14.470 18.530 26.300 1.00 0.00

ATOM 19 C19 KIH 1 14.800 19.420 25.300 1.00 0.00

ATOM 20 C20 KIH 1 15.230 20.670 25.680 1.00 0.00

ATOM 21 C21 KIH 1 15.700 21.480 24.670 1.00 0.00

ATOM 22 C22 KIH 1 15.690 21.120 23.350 1.00 0.00

ATOM 23 C23 KIH 1 16.070 22.060 22.420 1.00 0.00

ATOM 24 C24 KIH 1 15.890 21.830 21.070 1.00 0.00

ATOM 25 H1 KIH 1 13.240 19.810 19.540 1.00 0.00

ATOM 26 H2 KIH 1 11.850 18.980 17.760 1.00 0.00

ATOM 27 H3 KIH 1 11.480 18.310 15.480 1.00 0.00

ATOM 28 H4 KIH 1 13.300 18.270 13.920 1.00 0.00

ATOM 29 H5 KIH 1 15.600 18.960 14.700 1.00 0.00

ATOM 30 H6 KIH 1 14.530 18.840 21.330 1.00 0.00

ATOM 31 H7 KIH 1 14.270 17.350 22.690 1.00 0.00

ATOM 32 H8 KIH 1 13.840 15.810 24.370 1.00 0.00

ATOM 33 H9 KIH 1 13.810 16.610 26.840 1.00 0.00

ATOM 34 H10 KIH 1 14.440 18.810 27.340 1.00 0.00

ATOM 35 H11 KIH 1 15.270 21.000 26.700 1.00 0.00

ATOM 36 H12 KIH 1 16.050 22.460 24.980 1.00 0.00

ATOM 37 H13 KIH 1 16.600 22.950 22.700 1.00 0.00

ATOM 38 H14 KIH 1 16.270 22.510 20.320 1.00 0.00

ATOM 39 O1 KIH 1 17.640 20.470 18.940 1.00 0.00

ATOM 40 O2 KIH 1 19.450 18.020 22.680 1.00 0.00

ATOM 41 C25 KIH 1 17.450 19.910 16.300 1.00 0.00

ATOM 42 C26 KIH 1 18.570 19.170 16.620 1.00 0.00

ATOM 43 C27 KIH 1 19.710 19.270 15.830 1.00 0.00

ATOM 44 C28 KIH 1 19.780 20.380 15.030 1.00 0.00

ATOM 45 C29 KIH 1 18.670 21.150 14.750 1.00 0.00

ATOM 46 C30 KIH 1 17.460 20.900 15.360 1.00 0.00

ATOM 47 C31 KIH 1 18.800 22.220 13.890 1.00 0.00

ATOM 48 C32 KIH 1 17.740 23.040 13.620 1.00 0.00

ATOM 49 C33 KIH 1 16.540 22.870 14.290 1.00 0.00

ATOM 50 C34 KIH 1 16.430 21.790 15.130 1.00 0.00

ATOM 51 C35 KIH 1 20.970 18.550 16.140 1.00 0.00

ATOM 52 C36 KIH 1 21.000 17.170 16.270 1.00 0.00

ATOM 53 C37 KIH 1 22.110 16.430 16.560 1.00 0.00

ATOM 54 C38 KIH 1 22.120 15.070 16.790 1.00 0.00

ATOM 55 C39 KIH 1 20.950 14.340 16.790 1.00 0.00

ATOM 56 C40 KIH 1 20.910 13.010 17.110 1.00 0.00

ATOM 57 C41 KIH 1 22.100 12.380 17.430 1.00 0.00

ATOM 58 C42 KIH 1 23.290 13.040 17.250 1.00 0.00

ATOM 59 C43 KIH 1 23.300 14.390 16.950 1.00 0.00

ATOM 60 C44 KIH 1 24.510 15.050 16.880 1.00 0.00

ATOM 61 C45 KIH 1 24.490 16.420 16.780 1.00 0.00

ATOM 62 C46 KIH 1 23.300 17.110 16.660 1.00 0.00

ATOM 63 C47 KIH 1 23.340 18.490 16.590 1.00 0.00

ATOM 64 C48 KIH 1 22.160 19.180 16.400 1.00 0.00

ATOM 65 H15 KIH 1 20.690 20.680 14.530 1.00 0.00

ATOM 66 H16 KIH 1 19.740 22.340 13.360 1.00 0.00

ATOM 67 H17 KIH 1 17.730 23.910 12.990 1.00 0.00

ATOM 68 H18 KIH 1 15.770 23.590 14.070 1.00 0.00

ATOM 69 H19 KIH 1 15.500 21.710 15.680 1.00 0.00

ATOM 70 H20 KIH 1 19.970 16.830 16.270 1.00 0.00

ATOM 71 H21 KIH 1 20.010 14.820 16.550 1.00 0.00

ATOM 72 H22 KIH 1 19.980 12.460 17.200 1.00 0.00

ATOM 73 H23 KIH 1 22.180 11.330 17.690 1.00 0.00

ATOM 74 H24 KIH 1 24.170 12.490 17.540 1.00 0.00

ATOM 75 H25 KIH 1 25.500 14.660 16.940 1.00 0.00

ATOM 76 H26 KIH 1 25.450 16.920 16.700 1.00 0.00

ATOM 77 H27 KIH 1 24.270 19.010 16.680 1.00 0.00

ATOM 78 H28 KIH 1 22.230 20.260 16.340 1.00 0.00

ATOM 79 O3 KIH 1 18.740 18.510 17.820 1.00 0.00

ATOM 80 O4 KIH 1 17.400 16.670 22.620 1.00 0.00

ATOM 81 C49 KIH 1 19.530 15.910 21.420 1.00 0.00

ATOM 82 F1 KIH 1 18.880 15.170 20.510 1.00 0.00

ATOM 83 F2 KIH 1 20.670 16.270 20.830 1.00 0.00

ATOM 84 F3 KIH 1 19.950 15.060 22.360 1.00 0.00

ATOM 85 N1 KIH 1 18.090 18.250 20.550 1.00 0.00

ATOM 86 P1 KIH 1 18.650 19.280 19.200 1.00 0.00

ATOM 87 S1 KIH 1 18.490 17.290 21.890 1.00 0.00

ATOM 88 C50 KIH 1 23.990 21.720 19.160 1.00 0.00

ATOM 89 C51 KIH 1 23.770 20.500 19.740 1.00 0.00

ATOM 90 C52 KIH 1 24.750 19.840 20.430 1.00 0.00

ATOM 91 C53 KIH 1 25.950 20.500 20.600 1.00 0.00

ATOM 92 C54 KIH 1 26.130 21.790 20.170 1.00 0.00

ATOM 93 C55 KIH 1 25.120 22.420 19.480 1.00 0.00

ATOM 94 C56 KIH 1 27.200 22.490 20.660 1.00 0.00

ATOM 95 C57 KIH 1 27.310 23.840 20.370 1.00 0.00

ATOM 96 C58 KIH 1 26.360 24.450 19.590 1.00 0.00

ATOM 97 C59 KIH 1 25.270 23.750 19.130 1.00 0.00

ATOM 98 C60 KIH 1 24.480 18.520 21.080 1.00 0.00

ATOM 99 C61 KIH 1 24.430 18.430 22.450 1.00 0.00

ATOM 100 C62 KIH 1 24.230 17.270 23.140 1.00 0.00

ATOM 101 C63 KIH 1 24.020 17.170 24.490 1.00 0.00

ATOM 102 C64 KIH 1 24.140 18.280 25.300 1.00 0.00

ATOM 103 C65 KIH 1 23.970 18.220 26.660 1.00 0.00

ATOM 104 C66 KIH 1 23.860 16.960 27.210 1.00 0.00

ATOM 105 C67 KIH 1 23.940 15.820 26.450 1.00 0.00

ATOM 106 C68 KIH 1 23.940 15.930 25.080 1.00 0.00

ATOM 107 C69 KIH 1 24.110 14.790 24.340 1.00 0.00

ATOM 108 C70 KIH 1 24.290 14.900 22.980 1.00 0.00

ATOM 109 C71 KIH 1 24.280 16.130 22.370 1.00 0.00

ATOM 110 C72 KIH 1 24.390 16.180 21.000 1.00 0.00

ATOM 111 C73 KIH 1 24.450 17.380 20.330 1.00 0.00

ATOM 112 H29 KIH 1 26.730 19.990 21.140 1.00 0.00

ATOM 113 H30 KIH 1 27.920 22.050 21.320 1.00 0.00

ATOM 114 H31 KIH 1 28.140 24.460 20.650 1.00 0.00

ATOM 115 H32 KIH 1 26.510 25.500 19.390 1.00 0.00

ATOM 116 H33 KIH 1 24.470 24.240 18.600 1.00 0.00

ATOM 117 H34 KIH 1 24.690 19.430 22.810 1.00 0.00

ATOM 118 H35 KIH 1 24.280 19.250 24.850 1.00 0.00

ATOM 119 H36 KIH 1 23.810 19.090 27.270 1.00 0.00

ATOM 120 H37 KIH 1 23.770 16.710 28.250 1.00 0.00

ATOM 121 H38 KIH 1 23.920 14.840 26.920 1.00 0.00

ATOM 122 H39 KIH 1 24.080 13.790 24.760 1.00 0.00

ATOM 123 H40 KIH 1 24.260 14.050 22.310 1.00 0.00

ATOM 124 H41 KIH 1 24.390 15.340 20.320 1.00 0.00

ATOM 125 H42 KIH 1 24.620 17.360 19.260 1.00 0.00

ATOM 126 O5 KIH 1 22.530 19.890 19.590 1.00 0.00

ATOM 127 O6 KIH 1 21.500 19.490 24.100 1.00 0.00

ATOM 128 C74 KIH 1 22.920 22.260 18.260 1.00 0.00

ATOM 129 C75 KIH 1 21.740 22.670 18.840 1.00 0.00

ATOM 130 C76 KIH 1 20.850 23.320 18.000 1.00 0.00

ATOM 131 C77 KIH 1 21.220 23.650 16.730 1.00 0.00

ATOM 132 C78 KIH 1 22.340 23.100 16.140 1.00 0.00

ATOM 133 C79 KIH 1 23.230 22.420 16.930 1.00 0.00

ATOM 134 C80 KIH 1 22.520 23.150 14.770 1.00 0.00

ATOM 135 C81 KIH 1 23.710 22.690 14.230 1.00 0.00

ATOM 136 C82 KIH 1 24.640 22.100 15.060 1.00 0.00

ATOM 137 C83 KIH 1 24.410 21.960 16.410 1.00 0.00

ATOM 138 C84 KIH 1 19.550 23.840 18.530 1.00 0.00

ATOM 139 C85 KIH 1 19.340 24.470 19.720 1.00 0.00

ATOM 140 C86 KIH 1 18.110 24.950 20.120 1.00 0.00

ATOM 141 C87 KIH 1 17.860 25.510 21.350 1.00 0.00

ATOM 142 C88 KIH 1 18.810 25.580 22.330 1.00 0.00

ATOM 143 C89 KIH 1 18.540 26.090 23.570 1.00 0.00

ATOM 144 C90 KIH 1 17.290 26.590 23.880 1.00 0.00

ATOM 145 C91 KIH 1 16.340 26.640 22.900 1.00 0.00

ATOM 146 C92 KIH 1 16.630 26.020 21.710 1.00 0.00

ATOM 147 C93 KIH 1 15.620 25.930 20.780 1.00 0.00

ATOM 148 C94 KIH 1 15.790 25.270 19.600 1.00 0.00

ATOM 149 C95 KIH 1 17.040 24.770 19.280 1.00 0.00

ATOM 150 C96 KIH 1 17.210 24.090 18.100 1.00 0.00

ATOM 151 C97 KIH 1 18.450 23.640 17.730 1.00 0.00

ATOM 152 H43 KIH 1 20.600 24.330 16.170 1.00 0.00

ATOM 153 H44 KIH 1 22.030 23.870 14.130 1.00 0.00

ATOM 154 H45 KIH 1 23.930 22.820 13.190 1.00 0.00

ATOM 155 H46 KIH 1 25.520 21.660 14.610 1.00 0.00

ATOM 156 H47 KIH 1 25.170 21.390 16.920 1.00 0.00

ATOM 157 H48 KIH 1 20.260 24.650 20.280 1.00 0.00

ATOM 158 H49 KIH 1 19.860 25.310 22.260 1.00 0.00

ATOM 159 H50 KIH 1 19.390 26.080 24.240 1.00 0.00

ATOM 160 H51 KIH 1 17.230 27.190 24.780 1.00 0.00

ATOM 161 H52 KIH 1 15.320 26.980 23.030 1.00 0.00

ATOM 162 H53 KIH 1 14.670 26.370 21.090 1.00 0.00

ATOM 163 H54 KIH 1 14.930 25.260 18.960 1.00 0.00

ATOM 164 H55 KIH 1 16.380 23.950 17.430 1.00 0.00

ATOM 165 H56 KIH 1 18.470 23.110 16.790 1.00 0.00

ATOM 166 O7 KIH 1 21.280 22.090 20.010 1.00 0.00

ATOM 167 O8 KIH 1 22.870 21.220 22.990 1.00 0.00

ATOM 168 C98 KIH 1 20.330 21.710 23.500 1.00 0.00

ATOM 169 F4 KIH 1 20.520 22.240 24.710 1.00 0.00

ATOM 170 F5 KIH 1 20.100 22.690 22.620 1.00 0.00

ATOM 171 F6 KIH 1 19.110 21.160 23.590 1.00 0.00

ATOM 172 N2 KIH 1 21.030 19.940 21.680 1.00 0.00

ATOM 173 P2 KIH 1 21.190 20.520 20.140 1.00 0.00

ATOM 174 S2 KIH 1 21.610 20.530 23.100 1.00 0.00

ATOM 175 N3 KIH 1 19.980 19.920 19.510 1.00 0.00

ATOM 176 H57 KIH 1 20.640 19.020 21.850 1.00 0.00

TER

ENDMDL

REMARK GENERATED BY TRJCONV

TITLE KIH t= 8000.00000

REMARK THIS IS A SIMULATION BOX

CRYST1 38.340 38.340 38.340 90.00 90.00 90.00 P 1 1

MODEL 17

ATOM 1 C1 KIH 1 16.120 19.690 17.340 1.00 0.00

ATOM 2 C2 KIH 1 16.290 20.130 18.630 1.00 0.00

ATOM 3 C3 KIH 1 15.230 20.350 19.470 1.00 0.00

ATOM 4 C4 KIH 1 13.970 20.040 19.000 1.00 0.00

ATOM 5 C5 KIH 1 13.770 19.400 17.810 1.00 0.00

ATOM 6 C6 KIH 1 14.870 19.260 16.980 1.00 0.00

ATOM 7 C7 KIH 1 12.560 18.850 17.490 1.00 0.00

ATOM 8 C8 KIH 1 12.370 18.220 16.270 1.00 0.00

ATOM 9 C9 KIH 1 13.440 18.150 15.410 1.00 0.00

ATOM 10 C10 KIH 1 14.690 18.580 15.800 1.00 0.00

ATOM 11 C11 KIH 1 15.490 20.700 20.890 1.00 0.00

ATOM 12 C12 KIH 1 15.310 19.860 21.970 1.00 0.00

ATOM 13 C13 KIH 1 15.430 20.250 23.290 1.00 0.00

ATOM 14 C14 KIH 1 15.380 19.370 24.350 1.00 0.00

ATOM 15 C15 KIH 1 15.140 18.020 24.300 1.00 0.00

ATOM 16 C16 KIH 1 15.050 17.200 25.400 1.00 0.00

ATOM 17 C17 KIH 1 15.320 17.750 26.630 1.00 0.00

ATOM 18 C18 KIH 1 15.640 19.080 26.720 1.00 0.00

ATOM 19 C19 KIH 1 15.700 19.860 25.590 1.00 0.00

ATOM 20 C20 KIH 1 16.070 21.170 25.780 1.00 0.00

ATOM 21 C21 KIH 1 16.190 22.030 24.710 1.00 0.00

ATOM 22 C22 KIH 1 15.900 21.530 23.460 1.00 0.00

ATOM 23 C23 KIH 1 16.070 22.430 22.430 1.00 0.00

ATOM 24 C24 KIH 1 16.010 21.960 21.140 1.00 0.00

ATOM 25 H1 KIH 1 13.150 20.180 19.690 1.00 0.00

ATOM 26 H2 KIH 1 11.740 19.070 18.160 1.00 0.00

ATOM 27 H3 KIH 1 11.400 17.920 15.870 1.00 0.00

ATOM 28 H4 KIH 1 13.280 17.670 14.460 1.00 0.00

ATOM 29 H5 KIH 1 15.480 18.360 15.080 1.00 0.00

ATOM 30 H6 KIH 1 15.090 18.840 21.690 1.00 0.00

ATOM 31 H7 KIH 1 15.160 17.530 23.330 1.00 0.00

ATOM 32 H8 KIH 1 14.820 16.160 25.270 1.00 0.00

ATOM 33 H9 KIH 1 15.240 17.170 27.540 1.00 0.00

ATOM 34 H10 KIH 1 15.770 19.510 27.700 1.00 0.00

ATOM 35 H11 KIH 1 16.430 21.480 26.760 1.00 0.00

ATOM 36 H12 KIH 1 16.640 23.010 24.800 1.00 0.00

ATOM 37 H13 KIH 1 16.450 23.410 22.650 1.00 0.00

ATOM 38 H14 KIH 1 16.220 22.600 20.290 1.00 0.00

ATOM 39 O1 KIH 1 17.590 20.410 19.040 1.00 0.00

ATOM 40 O2 KIH 1 19.540 17.890 22.530 1.00 0.00

ATOM 41 C25 KIH 1 17.260 19.870 16.400 1.00 0.00

ATOM 42 C26 KIH 1 18.480 19.320 16.670 1.00 0.00

ATOM 43 C27 KIH 1 19.540 19.500 15.810 1.00 0.00

ATOM 44 C28 KIH 1 19.370 20.320 14.720 1.00 0.00

ATOM 45 C29 KIH 1 18.140 20.820 14.370 1.00 0.00

ATOM 46 C30 KIH 1 17.090 20.580 15.240 1.00 0.00

ATOM 47 C31 KIH 1 17.990 21.490 13.170 1.00 0.00

ATOM 48 C32 KIH 1 16.800 22.060 12.830 1.00 0.00

ATOM 49 C33 KIH 1 15.720 21.900 13.670 1.00 0.00

ATOM 50 C34 KIH 1 15.950 21.240 14.860 1.00 0.00

ATOM 51 C35 KIH 1 20.880 18.880 16.000 1.00 0.00

ATOM 52 C36 KIH 1 21.030 17.520 16.200 1.00 0.00

ATOM 53 C37 KIH 1 22.250 16.910 16.080 1.00 0.00

ATOM 54 C38 KIH 1 22.430 15.560 16.220 1.00 0.00

ATOM 55 C39 KIH 1 21.400 14.740 16.640 1.00 0.00

ATOM 56 C40 KIH 1 21.550 13.390 16.790 1.00 0.00

ATOM 57 C41 KIH 1 22.790 12.810 16.610 1.00 0.00

ATOM 58 C42 KIH 1 23.820 13.580 16.130 1.00 0.00

ATOM 59 C43 KIH 1 23.620 14.930 15.960 1.00 0.00

ATOM 60 C44 KIH 1 24.710 15.740 15.690 1.00 0.00

ATOM 61 C45 KIH 1 24.630 17.120 15.730 1.00 0.00

ATOM 62 C46 KIH 1 23.400 17.670 15.950 1.00 0.00

ATOM 63 C47 KIH 1 23.280 19.040 16.010 1.00 0.00

ATOM 64 C48 KIH 1 22.030 19.620 15.990 1.00 0.00

ATOM 65 H15 KIH 1 20.240 20.400 14.080 1.00 0.00

ATOM 66 H16 KIH 1 18.840 21.560 12.490 1.00 0.00

ATOM 67 H17 KIH 1 16.750 22.700 11.960 1.00 0.00

ATOM 68 H18 KIH 1 14.750 22.360 13.500 1.00 0.00

ATOM 69 H19 KIH 1 15.040 21.170 15.420 1.00 0.00

ATOM 70 H20 KIH 1 20.160 16.890 16.330 1.00 0.00

ATOM 71 H21 KIH 1 20.400 15.080 16.910 1.00 0.00

ATOM 72 H22 KIH 1 20.750 12.760 17.130 1.00 0.00

ATOM 73 H23 KIH 1 22.930 11.740 16.760 1.00 0.00

ATOM 74 H24 KIH 1 24.720 13.020 15.920 1.00 0.00

ATOM 75 H25 KIH 1 25.640 15.240 15.520 1.00 0.00

ATOM 76 H26 KIH 1 25.500 17.750 15.760 1.00 0.00

ATOM 77 H27 KIH 1 24.160 19.660 15.970 1.00 0.00

ATOM 78 H28 KIH 1 22.090 20.690 15.830 1.00 0.00

ATOM 79 O3 KIH 1 18.670 18.500 17.780 1.00 0.00

ATOM 80 O4 KIH 1 17.430 16.740 22.690 1.00 0.00

ATOM 81 C49 KIH 1 19.180 15.800 21.070 1.00 0.00

ATOM 82 F1 KIH 1 18.330 14.990 20.420 1.00 0.00

ATOM 83 F2 KIH 1 20.130 16.040 20.170 1.00 0.00

ATOM 84 F3 KIH 1 19.810 15.020 21.960 1.00 0.00

ATOM 85 N1 KIH 1 17.990 18.260 20.530 1.00 0.00

ATOM 86 P1 KIH 1 18.640 19.230 19.180 1.00 0.00

ATOM 87 S1 KIH 1 18.460 17.260 21.820 1.00 0.00

ATOM 88 C50 KIH 1 23.950 21.590 19.330 1.00 0.00

ATOM 89 C51 KIH 1 23.660 20.270 19.600 1.00 0.00

ATOM 90 C52 KIH 1 24.500 19.500 20.360 1.00 0.00

ATOM 91 C53 KIH 1 25.620 20.070 20.900 1.00 0.00

ATOM 92 C54 KIH 1 25.860 21.420 20.750 1.00 0.00

ATOM 93 C55 KIH 1 25.040 22.170 19.950 1.00 0.00

ATOM 94 C56 KIH 1 26.960 21.960 21.390 1.00 0.00

ATOM 95 C57 KIH 1 27.160 23.320 21.430 1.00 0.00

ATOM 96 C58 KIH 1 26.280 24.090 20.710 1.00 0.00

ATOM 97 C59 KIH 1 25.270 23.530 19.960 1.00 0.00

ATOM 98 C60 KIH 1 24.280 18.030 20.510 1.00 0.00

ATOM 99 C61 KIH 1 24.050 17.540 21.770 1.00 0.00

ATOM 100 C62 KIH 1 23.660 16.230 21.940 1.00 0.00

ATOM 101 C63 KIH 1 23.320 15.740 23.170 1.00 0.00

ATOM 102 C64 KIH 1 23.420 16.510 24.300 1.00 0.00

ATOM 103 C65 KIH 1 23.060 15.970 25.520 1.00 0.00

ATOM 104 C66 KIH 1 22.700 14.660 25.650 1.00 0.00

ATOM 105 C67 KIH 1 22.660 13.870 24.520 1.00 0.00

ATOM 106 C68 KIH 1 22.880 14.440 23.290 1.00 0.00

ATOM 107 C69 KIH 1 22.770 13.640 22.190 1.00 0.00

ATOM 108 C70 KIH 1 23.000 14.150 20.930 1.00 0.00

ATOM 109 C71 KIH 1 23.540 15.410 20.840 1.00 0.00

ATOM 110 C72 KIH 1 23.770 15.900 19.570 1.00 0.00

ATOM 111 C73 KIH 1 24.220 17.200 19.410 1.00 0.00

ATOM 112 H29 KIH 1 26.350 19.470 21.420 1.00 0.00

ATOM 113 H30 KIH 1 27.580 21.310 21.990 1.00 0.00

ATOM 114 H31 KIH 1 27.960 23.760 22.020 1.00 0.00

ATOM 115 H32 KIH 1 26.300 25.160 20.620 1.00 0.00

ATOM 116 H33 KIH 1 24.620 24.180 19.400 1.00 0.00

ATOM 117 H34 KIH 1 24.160 18.230 22.600 1.00 0.00

ATOM 118 H35 KIH 1 23.690 17.560 24.300 1.00 0.00

ATOM 119 H36 KIH 1 23.010 16.570 26.410 1.00 0.00

ATOM 120 H37 KIH 1 22.460 14.300 26.650 1.00 0.00

ATOM 121 H38 KIH 1 22.420 12.820 24.670 1.00 0.00

ATOM 122 H39 KIH 1 22.660 12.560 22.250 1.00 0.00

ATOM 123 H40 KIH 1 22.930 13.560 20.030 1.00 0.00

ATOM 124 H41 KIH 1 23.640 15.420 18.620 1.00 0.00

ATOM 125 H42 KIH 1 24.390 17.600 18.430 1.00 0.00

ATOM 126 O5 KIH 1 22.370 19.800 19.360 1.00 0.00

ATOM 127 O6 KIH 1 22.270 19.420 23.760 1.00 0.00

ATOM 128 C74 KIH 1 23.110 22.380 18.400 1.00 0.00

ATOM 129 C75 KIH 1 21.810 22.610 18.810 1.00 0.00

ATOM 130 C76 KIH 1 20.990 23.370 18.000 1.00 0.00

ATOM 131 C77 KIH 1 21.550 23.980 16.890 1.00 0.00

ATOM 132 C78 KIH 1 22.830 23.710 16.470 1.00 0.00

ATOM 133 C79 KIH 1 23.620 22.860 17.220 1.00 0.00

ATOM 134 C80 KIH 1 23.390 24.420 15.440 1.00 0.00

ATOM 135 C81 KIH 1 24.740 24.280 15.160 1.00 0.00

ATOM 136 C82 KIH 1 25.470 23.340 15.850 1.00 0.00

ATOM 137 C83 KIH 1 24.920 22.620 16.890 1.00 0.00

ATOM 138 C84 KIH 1 19.550 23.640 18.240 1.00 0.00

ATOM 139 C85 KIH 1 19.130 24.090 19.470 1.00 0.00

ATOM 140 C86 KIH 1 17.870 24.590 19.720 1.00 0.00

ATOM 141 C87 KIH 1 17.490 25.230 20.880 1.00 0.00

ATOM 142 C88 KIH 1 18.230 25.300 22.040 1.00 0.00

ATOM 143 C89 KIH 1 17.770 25.990 23.130 1.00 0.00

ATOM 144 C90 KIH 1 16.500 26.520 23.120 1.00 0.00

ATOM 145 C91 KIH 1 15.730 26.470 21.990 1.00 0.00

ATOM 146 C92 KIH 1 16.240 25.800 20.900 1.00 0.00

ATOM 147 C93 KIH 1 15.360 25.650 19.860 1.00 0.00

ATOM 148 C94 KIH 1 15.640 24.810 18.820 1.00 0.00

ATOM 149 C95 KIH 1 16.950 24.400 18.710 1.00 0.00

ATOM 150 C96 KIH 1 17.280 23.730 17.560 1.00 0.00

ATOM 151 C97 KIH 1 18.580 23.360 17.300 1.00 0.00

ATOM 152 H43 KIH 1 20.920 24.690 16.360 1.00 0.00

ATOM 153 H44 KIH 1 22.820 24.980 14.720 1.00 0.00

ATOM 154 H45 KIH 1 25.200 24.940 14.450 1.00 0.00

ATOM 155 H46 KIH 1 26.520 23.220 15.640 1.00 0.00

ATOM 156 H47 KIH 1 25.540 21.830 17.300 1.00 0.00

ATOM 157 H48 KIH 1 19.950 24.280 20.150 1.00 0.00

ATOM 158 H49 KIH 1 19.190 24.820 22.140 1.00 0.00

ATOM 159 H50 KIH 1 18.460 26.210 23.930 1.00 0.00

ATOM 160 H51 KIH 1 16.180 27.140 23.950 1.00 0.00

ATOM 161 H52 KIH 1 14.740 26.910 21.990 1.00 0.00

ATOM 162 H53 KIH 1 14.350 26.030 19.920 1.00 0.00

ATOM 163 H54 KIH 1 14.960 24.660 17.990 1.00 0.00

ATOM 164 H55 KIH 1 16.540 23.490 16.810 1.00 0.00

ATOM 165 H56 KIH 1 18.910 22.920 16.370 1.00 0.00

ATOM 166 O7 KIH 1 21.210 22.050 19.920 1.00 0.00

ATOM 167 O8 KIH 1 22.540 21.660 22.730 1.00 0.00

ATOM 168 C98 KIH 1 20.370 21.210 24.180 1.00 0.00

ATOM 169 F4 KIH 1 20.730 21.410 25.460 1.00 0.00

ATOM 170 F5 KIH 1 19.880 22.390 23.770 1.00 0.00

ATOM 171 F6 KIH 1 19.320 20.370 24.250 1.00 0.00

ATOM 172 N2 KIH 1 21.120 20.010 21.720 1.00 0.00

ATOM 173 P2 KIH 1 21.180 20.490 20.140 1.00 0.00

ATOM 174 S2 KIH 1 21.690 20.590 23.150 1.00 0.00

ATOM 175 N3 KIH 1 19.910 19.920 19.570 1.00 0.00

ATOM 176 H57 KIH 1 20.580 19.180 21.870 1.00 0.00

TER

ENDMDL

REMARK GENERATED BY TRJCONV

TITLE KIH t= 8500.00000

REMARK THIS IS A SIMULATION BOX

CRYST1 38.428 38.428 38.428 90.00 90.00 90.00 P 1 1

MODEL 18

ATOM 1 C1 KIH 1 16.230 19.710 17.180 1.00 0.00

ATOM 2 C2 KIH 1 16.340 20.120 18.490 1.00 0.00

ATOM 3 C3 KIH 1 15.230 20.100 19.310 1.00 0.00

ATOM 4 C4 KIH 1 14.020 19.580 18.900 1.00 0.00

ATOM 5 C5 KIH 1 13.940 19.080 17.620 1.00 0.00

ATOM 6 C6 KIH 1 15.050 19.100 16.800 1.00 0.00

ATOM 7 C7 KIH 1 12.790 18.470 17.170 1.00 0.00

ATOM 8 C8 KIH 1 12.740 17.860 15.940 1.00 0.00

ATOM 9 C9 KIH 1 13.800 17.970 15.090 1.00 0.00

ATOM 10 C10 KIH 1 14.970 18.540 15.560 1.00 0.00

ATOM 11 C11 KIH 1 15.330 20.640 20.690 1.00 0.00

ATOM 12 C12 KIH 1 15.360 19.770 21.750 1.00 0.00

ATOM 13 C13 KIH 1 15.630 20.200 23.020 1.00 0.00

ATOM 14 C14 KIH 1 15.690 19.400 24.140 1.00 0.00

ATOM 15 C15 KIH 1 15.680 18.030 24.090 1.00 0.00

ATOM 16 C16 KIH 1 15.920 17.290 25.230 1.00 0.00

ATOM 17 C17 KIH 1 16.080 17.930 26.440 1.00 0.00

ATOM 18 C18 KIH 1 16.140 19.290 26.520 1.00 0.00

ATOM 19 C19 KIH 1 15.930 20.000 25.360 1.00 0.00

ATOM 20 C20 KIH 1 16.240 21.330 25.540 1.00 0.00

ATOM 21 C21 KIH 1 16.270 22.090 24.400 1.00 0.00

ATOM 22 C22 KIH 1 15.860 21.540 23.210 1.00 0.00

ATOM 23 C23 KIH 1 15.890 22.420 22.140 1.00 0.00

ATOM 24 C24 KIH 1 15.580 21.980 20.880 1.00 0.00

ATOM 25 H1 KIH 1 13.170 19.420 19.540 1.00 0.00

ATOM 26 H2 KIH 1 11.900 18.320 17.770 1.00 0.00

ATOM 27 H3 KIH 1 11.830 17.320 15.710 1.00 0.00

ATOM 28 H4 KIH 1 13.770 17.640 14.060 1.00 0.00

ATOM 29 H5 KIH 1 15.800 18.610 14.860 1.00 0.00

ATOM 30 H6 KIH 1 15.210 18.730 21.500 1.00 0.00

ATOM 31 H7 KIH 1 15.680 17.550 23.120 1.00 0.00

ATOM 32 H8 KIH 1 16.040 16.210 25.250 1.00 0.00

ATOM 33 H9 KIH 1 16.130 17.300 27.320 1.00 0.00

ATOM 34 H10 KIH 1 16.480 19.760 27.430 1.00 0.00

ATOM 35 H11 KIH 1 16.670 21.800 26.410 1.00 0.00

ATOM 36 H12 KIH 1 16.520 23.140 24.460 1.00 0.00

ATOM 37 H13 KIH 1 16.160 23.450 22.270 1.00 0.00

ATOM 38 H14 KIH 1 15.540 22.740 20.110 1.00 0.00

ATOM 39 O1 KIH 1 17.550 20.430 19.080 1.00 0.00

ATOM 40 O2 KIH 1 19.140 18.340 22.650 1.00 0.00

ATOM 41 C25 KIH 1 17.380 20.040 16.310 1.00 0.00

ATOM 42 C26 KIH 1 18.530 19.320 16.580 1.00 0.00

ATOM 43 C27 KIH 1 19.580 19.360 15.700 1.00 0.00

ATOM 44 C28 KIH 1 19.560 20.180 14.600 1.00 0.00

ATOM 45 C29 KIH 1 18.430 20.910 14.350 1.00 0.00

ATOM 46 C30 KIH 1 17.340 20.840 15.190 1.00 0.00

ATOM 47 C31 KIH 1 18.390 21.790 13.290 1.00 0.00

ATOM 48 C32 KIH 1 17.240 22.440 12.950 1.00 0.00

ATOM 49 C33 KIH 1 16.080 22.260 13.680 1.00 0.00

ATOM 50 C34 KIH 1 16.180 21.460 14.800 1.00 0.00

ATOM 51 C35 KIH 1 20.710 18.400 15.860 1.00 0.00

ATOM 52 C36 KIH 1 20.800 17.200 15.180 1.00 0.00

ATOM 53 C37 KIH 1 21.960 16.470 15.170 1.00 0.00

ATOM 54 C38 KIH 1 22.060 15.220 14.580 1.00 0.00

ATOM 55 C39 KIH 1 21.000 14.530 14.080 1.00 0.00

ATOM 56 C40 KIH 1 21.140 13.260 13.550 1.00 0.00

ATOM 57 C41 KIH 1 22.390 12.700 13.540 1.00 0.00

ATOM 58 C42 KIH 1 23.470 13.360 14.080 1.00 0.00

ATOM 59 C43 KIH 1 23.320 14.660 14.520 1.00 0.00

ATOM 60 C44 KIH 1 24.460 15.290 14.960 1.00 0.00

ATOM 61 C45 KIH 1 24.320 16.450 15.670 1.00 0.00

ATOM 62 C46 KIH 1 23.070 17.000 15.790 1.00 0.00

ATOM 63 C47 KIH 1 22.980 18.240 16.380 1.00 0.00

ATOM 64 C48 KIH 1 21.810 18.960 16.480 1.00 0.00

ATOM 65 H15 KIH 1 20.420 20.220 13.930 1.00 0.00

ATOM 66 H16 KIH 1 19.290 21.920 12.720 1.00 0.00

ATOM 67 H17 KIH 1 17.220 22.990 12.030 1.00 0.00

ATOM 68 H18 KIH 1 15.080 22.610 13.450 1.00 0.00

ATOM 69 H19 KIH 1 15.390 21.340 15.530 1.00 0.00

ATOM 70 H20 KIH 1 19.930 16.840 14.650 1.00 0.00

ATOM 71 H21 KIH 1 20.050 15.040 14.130 1.00 0.00

ATOM 72 H22 KIH 1 20.260 12.770 13.140 1.00 0.00

ATOM 73 H23 KIH 1 22.440 11.760 13.020 1.00 0.00

ATOM 74 H24 KIH 1 24.460 12.910 14.110 1.00 0.00

ATOM 75 H25 KIH 1 25.440 14.840 14.840 1.00 0.00

ATOM 76 H26 KIH 1 25.230 16.920 16.020 1.00 0.00

ATOM 77 H27 KIH 1 23.880 18.750 16.670 1.00 0.00

ATOM 78 H28 KIH 1 21.720 19.930 16.930 1.00 0.00

ATOM 79 O3 KIH 1 18.700 18.590 17.740 1.00 0.00

ATOM 80 O4 KIH 1 17.460 16.560 22.480 1.00 0.00

ATOM 81 C49 KIH 1 19.740 16.110 21.410 1.00 0.00

ATOM 82 F1 KIH 1 19.250 15.090 20.720 1.00 0.00

ATOM 83 F2 KIH 1 20.740 16.710 20.740 1.00 0.00

ATOM 84 F3 KIH 1 20.240 15.550 22.520 1.00 0.00

ATOM 85 N1 KIH 1 18.020 18.160 20.430 1.00 0.00

ATOM 86 P1 KIH 1 18.620 19.270 19.170 1.00 0.00

ATOM 87 S1 KIH 1 18.500 17.320 21.840 1.00 0.00

ATOM 88 C50 KIH 1 24.100 21.100 19.210 1.00 0.00

ATOM 89 C51 KIH 1 23.660 19.930 19.810 1.00 0.00

ATOM 90 C52 KIH 1 24.580 19.080 20.380 1.00 0.00

ATOM 91 C53 KIH 1 25.820 19.510 20.780 1.00 0.00

ATOM 92 C54 KIH 1 26.220 20.750 20.340 1.00 0.00

ATOM 93 C55 KIH 1 25.340 21.520 19.620 1.00 0.00

ATOM 94 C56 KIH 1 27.510 21.140 20.620 1.00 0.00

ATOM 95 C57 KIH 1 27.940 22.380 20.210 1.00 0.00

ATOM 96 C58 KIH 1 27.060 23.270 19.650 1.00 0.00

ATOM 97 C59 KIH 1 25.820 22.780 19.300 1.00 0.00

ATOM 98 C60 KIH 1 24.100 17.740 20.840 1.00 0.00

ATOM 99 C61 KIH 1 23.980 17.340 22.150 1.00 0.00

ATOM 100 C62 KIH 1 23.640 16.080 22.590 1.00 0.00

ATOM 101 C63 KIH 1 23.590 15.720 23.920 1.00 0.00

ATOM 102 C64 KIH 1 23.670 16.640 24.940 1.00 0.00

ATOM 103 C65 KIH 1 23.810 16.310 26.270 1.00 0.00

ATOM 104 C66 KIH 1 23.640 14.980 26.550 1.00 0.00

ATOM 105 C67 KIH 1 23.310 14.050 25.600 1.00 0.00

ATOM 106 C68 KIH 1 23.370 14.410 24.270 1.00 0.00

ATOM 107 C69 KIH 1 23.160 13.450 23.300 1.00 0.00

ATOM 108 C70 KIH 1 23.060 13.890 22.000 1.00 0.00

ATOM 109 C71 KIH 1 23.390 15.170 21.600 1.00 0.00

ATOM 110 C72 KIH 1 23.460 15.530 20.270 1.00 0.00

ATOM 111 C73 KIH 1 23.880 16.780 19.880 1.00 0.00

ATOM 112 H29 KIH 1 26.330 18.880 21.500 1.00 0.00

ATOM 113 H30 KIH 1 28.130 20.580 21.290 1.00 0.00

ATOM 114 H31 KIH 1 28.930 22.770 20.450 1.00 0.00

ATOM 115 H32 KIH 1 27.410 24.280 19.520 1.00 0.00

ATOM 116 H33 KIH 1 25.140 23.500 18.860 1.00 0.00

ATOM 117 H34 KIH 1 24.220 18.170 22.790 1.00 0.00

ATOM 118 H35 KIH 1 23.870 17.680 24.710 1.00 0.00

ATOM 119 H36 KIH 1 23.790 17.020 27.100 1.00 0.00

ATOM 120 H37 KIH 1 23.700 14.610 27.570 1.00 0.00

ATOM 121 H38 KIH 1 23.040 13.040 25.890 1.00 0.00

ATOM 122 H39 KIH 1 22.910 12.440 23.570 1.00 0.00

ATOM 123 H40 KIH 1 22.940 13.220 21.160 1.00 0.00

ATOM 124 H41 KIH 1 23.350 14.710 19.580 1.00 0.00

ATOM 125 H42 KIH 1 24.020 16.930 18.820 1.00 0.00

ATOM 126 O5 KIH 1 22.350 19.510 19.670 1.00 0.00

ATOM 127 O6 KIH 1 22.060 19.510 24.020 1.00 0.00

ATOM 128 C74 KIH 1 23.210 21.770 18.210 1.00 0.00

ATOM 129 C75 KIH 1 22.000 22.260 18.650 1.00 0.00

ATOM 130 C76 KIH 1 21.130 22.950 17.840 1.00 0.00

ATOM 131 C77 KIH 1 21.460 22.940 16.510 1.00 0.00

ATOM 132 C78 KIH 1 22.600 22.320 16.040 1.00 0.00

ATOM 133 C79 KIH 1 23.490 21.690 16.870 1.00 0.00

ATOM 134 C80 KIH 1 22.870 22.400 14.690 1.00 0.00

ATOM 135 C81 KIH 1 24.040 21.890 14.180 1.00 0.00

ATOM 136 C82 KIH 1 24.920 21.240 15.020 1.00 0.00

ATOM 137 C83 KIH 1 24.660 21.180 16.370 1.00 0.00

ATOM 138 C84 KIH 1 19.860 23.550 18.340 1.00 0.00

ATOM 139 C85 KIH 1 19.820 24.320 19.480 1.00 0.00

ATOM 140 C86 KIH 1 18.680 24.940 19.940 1.00 0.00

ATOM 141 C87 KIH 1 18.640 25.540 21.180 1.00 0.00

ATOM 142 C88 KIH 1 19.770 25.730 21.930 1.00 0.00

ATOM 143 C89 KIH 1 19.740 26.360 23.160 1.00 0.00

ATOM 144 C90 KIH 1 18.550 26.880 23.600 1.00 0.00

ATOM 145 C91 KIH 1 17.400 26.680 22.870 1.00 0.00

ATOM 146 C92 KIH 1 17.460 26.060 21.640 1.00 0.00

ATOM 147 C93 KIH 1 16.320 25.980 20.880 1.00 0.00

ATOM 148 C94 KIH 1 16.380 25.460 19.610 1.00 0.00

ATOM 149 C95 KIH 1 17.510 24.790 19.220 1.00 0.00

ATOM 150 C96 KIH 1 17.510 24.010 18.100 1.00 0.00

ATOM 151 C97 KIH 1 18.670 23.380 17.680 1.00 0.00

ATOM 152 H43 KIH 1 20.770 23.460 15.870 1.00 0.00

ATOM 153 H44 KIH 1 22.080 22.850 14.110 1.00 0.00

ATOM 154 H45 KIH 1 24.360 21.920 13.150 1.00 0.00

ATOM 155 H46 KIH 1 25.800 20.770 14.600 1.00 0.00

ATOM 156 H47 KIH 1 25.410 20.650 16.950 1.00 0.00

ATOM 157 H48 KIH 1 20.800 24.370 19.930 1.00 0.00

ATOM 158 H49 KIH 1 20.760 25.350 21.730 1.00 0.00

ATOM 159 H50 KIH 1 20.700 26.360 23.650 1.00 0.00

ATOM 160 H51 KIH 1 18.540 27.370 24.570 1.00 0.00

ATOM 161 H52 KIH 1 16.620 27.290 23.300 1.00 0.00

ATOM 162 H53 KIH 1 15.400 26.380 21.280 1.00 0.00

ATOM 163 H54 KIH 1 15.490 25.410 19.000 1.00 0.00

ATOM 164 H55 KIH 1 16.670 23.930 17.420 1.00 0.00

ATOM 165 H56 KIH 1 18.610 22.690 16.850 1.00 0.00

ATOM 166 O7 KIH 1 21.550 21.940 19.910 1.00 0.00

ATOM 167 O8 KIH 1 22.770 21.480 22.690 1.00 0.00

ATOM 168 C98 KIH 1 20.450 21.550 23.980 1.00 0.00

ATOM 169 F4 KIH 1 19.460 20.790 24.460 1.00 0.00

ATOM 170 F5 KIH 1 20.970 22.200 25.030 1.00 0.00

ATOM 171 F6 KIH 1 19.900 22.510 23.220 1.00 0.00

ATOM 172 N2 KIH 1 21.060 19.970 21.770 1.00 0.00

ATOM 173 P2 KIH 1 21.160 20.420 20.180 1.00 0.00

ATOM 174 S2 KIH 1 21.720 20.600 23.150 1.00 0.00

ATOM 175 N3 KIH 1 19.890 19.970 19.540 1.00 0.00

ATOM 176 H57 KIH 1 20.540 19.120 21.940 1.00 0.00

TER

ENDMDL

REMARK GENERATED BY TRJCONV

TITLE KIH t= 9000.00000

REMARK THIS IS A SIMULATION BOX

CRYST1 38.608 38.608 38.608 90.00 90.00 90.00 P 1 1

MODEL 19

ATOM 1 C1 KIH 1 16.070 19.530 17.360 1.00 0.00

ATOM 2 C2 KIH 1 16.240 20.060 18.630 1.00 0.00

ATOM 3 C3 KIH 1 15.220 19.860 19.520 1.00 0.00

ATOM 4 C4 KIH 1 14.080 19.150 19.210 1.00 0.00

ATOM 5 C5 KIH 1 13.960 18.600 17.950 1.00 0.00

ATOM 6 C6 KIH 1 14.970 18.760 17.030 1.00 0.00

ATOM 7 C7 KIH 1 12.890 17.750 17.760 1.00 0.00

ATOM 8 C8 KIH 1 12.780 17.280 16.470 1.00 0.00

ATOM 9 C9 KIH 1 13.690 17.470 15.460 1.00 0.00

ATOM 10 C10 KIH 1 14.810 18.180 15.790 1.00 0.00

ATOM 11 C11 KIH 1 15.390 20.340 20.920 1.00 0.00

ATOM 12 C12 KIH 1 15.160 19.580 22.040 1.00 0.00

ATOM 13 C13 KIH 1 15.550 20.020 23.280 1.00 0.00

ATOM 14 C14 KIH 1 15.540 19.220 24.400 1.00 0.00

ATOM 15 C15 KIH 1 15.310 17.870 24.330 1.00 0.00

ATOM 16 C16 KIH 1 15.280 17.090 25.460 1.00 0.00

ATOM 17 C17 KIH 1 15.480 17.650 26.690 1.00 0.00

ATOM 18 C18 KIH 1 15.730 19.000 26.780 1.00 0.00

ATOM 19 C19 KIH 1 15.850 19.750 25.630 1.00 0.00

ATOM 20 C20 KIH 1 16.200 21.070 25.750 1.00 0.00

ATOM 21 C21 KIH 1 16.190 21.890 24.650 1.00 0.00

ATOM 22 C22 KIH 1 15.890 21.350 23.420 1.00 0.00

ATOM 23 C23 KIH 1 16.040 22.170 22.320 1.00 0.00

ATOM 24 C24 KIH 1 15.850 21.630 21.070 1.00 0.00

ATOM 25 H1 KIH 1 13.480 18.910 20.070 1.00 0.00

ATOM 26 H2 KIH 1 12.140 17.610 18.520 1.00 0.00

ATOM 27 H3 KIH 1 11.850 16.810 16.180 1.00 0.00

ATOM 28 H4 KIH 1 13.590 16.990 14.500 1.00 0.00

ATOM 29 H5 KIH 1 15.570 18.400 15.040 1.00 0.00

ATOM 30 H6 KIH 1 14.870 18.580 21.760 1.00 0.00

ATOM 31 H7 KIH 1 15.150 17.350 23.400 1.00 0.00

ATOM 32 H8 KIH 1 15.240 16.010 25.450 1.00 0.00

ATOM 33 H9 KIH 1 15.410 16.970 27.530 1.00 0.00

ATOM 34 H10 KIH 1 15.860 19.390 27.780 1.00 0.00

ATOM 35 H11 KIH 1 16.500 21.500 26.700 1.00 0.00

ATOM 36 H12 KIH 1 16.370 22.950 24.750 1.00 0.00

ATOM 37 H13 KIH 1 16.420 23.170 22.410 1.00 0.00

ATOM 38 H14 KIH 1 15.970 22.220 20.170 1.00 0.00

ATOM 39 O1 KIH 1 17.520 20.320 19.070 1.00 0.00

ATOM 40 O2 KIH 1 19.310 18.500 22.740 1.00 0.00

ATOM 41 C25 KIH 1 17.100 20.000 16.400 1.00 0.00

ATOM 42 C26 KIH 1 18.300 19.370 16.570 1.00 0.00

ATOM 43 C27 KIH 1 19.310 19.740 15.710 1.00 0.00

ATOM 44 C28 KIH 1 19.130 20.650 14.690 1.00 0.00

ATOM 45 C29 KIH 1 17.910 21.250 14.530 1.00 0.00

ATOM 46 C30 KIH 1 16.900 20.940 15.410 1.00 0.00

ATOM 47 C31 KIH 1 17.670 22.110 13.490 1.00 0.00

ATOM 48 C32 KIH 1 16.450 22.760 13.450 1.00 0.00

ATOM 49 C33 KIH 1 15.480 22.540 14.390 1.00 0.00

ATOM 50 C34 KIH 1 15.690 21.580 15.360 1.00 0.00

ATOM 51 C35 KIH 1 20.630 19.080 15.910 1.00 0.00

ATOM 52 C36 KIH 1 20.770 17.760 15.540 1.00 0.00

ATOM 53 C37 KIH 1 21.960 17.080 15.450 1.00 0.00

ATOM 54 C38 KIH 1 22.170 15.800 15.010 1.00 0.00

ATOM 55 C39 KIH 1 21.140 15.050 14.500 1.00 0.00

ATOM 56 C40 KIH 1 21.350 13.800 13.990 1.00 0.00

ATOM 57 C41 KIH 1 22.590 13.210 14.070 1.00 0.00

ATOM 58 C42 KIH 1 23.630 13.930 14.610 1.00 0.00

ATOM 59 C43 KIH 1 23.400 15.200 15.110 1.00 0.00

ATOM 60 C44 KIH 1 24.490 15.960 15.450 1.00 0.00

ATOM 61 C45 KIH 1 24.320 17.260 15.870 1.00 0.00

ATOM 62 C46 KIH 1 23.060 17.800 15.880 1.00 0.00

ATOM 63 C47 KIH 1 22.910 19.090 16.370 1.00 0.00

ATOM 64 C48 KIH 1 21.730 19.780 16.340 1.00 0.00

ATOM 65 H15 KIH 1 19.960 20.960 14.070 1.00 0.00

ATOM 66 H16 KIH 1 18.430 22.220 12.740 1.00 0.00

ATOM 67 H17 KIH 1 16.230 23.410 12.610 1.00 0.00

ATOM 68 H18 KIH 1 14.550 23.070 14.320 1.00 0.00

ATOM 69 H19 KIH 1 14.850 21.470 16.030 1.00 0.00

ATOM 70 H20 KIH 1 19.930 17.280 15.080 1.00 0.00

ATOM 71 H21 KIH 1 20.110 15.360 14.370 1.00 0.00

ATOM 72 H22 KIH 1 20.560 13.190 13.560 1.00 0.00

ATOM 73 H23 KIH 1 22.750 12.200 13.710 1.00 0.00

ATOM 74 H24 KIH 1 24.660 13.610 14.560 1.00 0.00

ATOM 75 H25 KIH 1 25.500 15.610 15.630 1.00 0.00

ATOM 76 H26 KIH 1 25.170 17.900 16.070 1.00 0.00

ATOM 77 H27 KIH 1 23.770 19.660 16.690 1.00 0.00

ATOM 78 H28 KIH 1 21.570 20.780 16.720 1.00 0.00

ATOM 79 O3 KIH 1 18.610 18.580 17.670 1.00 0.00

ATOM 80 O4 KIH 1 17.620 16.680 22.600 1.00 0.00

ATOM 81 C49 KIH 1 19.860 16.320 21.470 1.00 0.00

ATOM 82 F1 KIH 1 19.420 15.390 20.580 1.00 0.00

ATOM 83 F2 KIH 1 20.960 16.830 20.910 1.00 0.00

ATOM 84 F3 KIH 1 20.240 15.610 22.540 1.00 0.00

ATOM 85 N1 KIH 1 18.140 18.150 20.470 1.00 0.00

ATOM 86 P1 KIH 1 18.640 19.210 19.110 1.00 0.00

ATOM 87 S1 KIH 1 18.610 17.500 21.970 1.00 0.00

ATOM 88 C50 KIH 1 24.040 21.840 19.720 1.00 0.00

ATOM 89 C51 KIH 1 23.730 20.530 19.980 1.00 0.00

ATOM 90 C52 KIH 1 24.670 19.730 20.590 1.00 0.00

ATOM 91 C53 KIH 1 25.790 20.290 21.150 1.00 0.00

ATOM 92 C54 KIH 1 26.080 21.630 20.990 1.00 0.00

ATOM 93 C55 KIH 1 25.220 22.360 20.200 1.00 0.00

ATOM 94 C56 KIH 1 27.240 22.160 21.480 1.00 0.00

ATOM 95 C57 KIH 1 27.580 23.450 21.140 1.00 0.00

ATOM 96 C58 KIH 1 26.740 24.200 20.350 1.00 0.00

ATOM 97 C59 KIH 1 25.530 23.680 19.970 1.00 0.00

ATOM 98 C60 KIH 1 24.380 18.310 20.900 1.00 0.00

ATOM 99 C61 KIH 1 24.250 17.840 22.190 1.00 0.00

ATOM 100 C62 KIH 1 23.800 16.560 22.440 1.00 0.00

ATOM 101 C63 KIH 1 23.520 16.080 23.700 1.00 0.00

ATOM 102 C64 KIH 1 23.440 16.890 24.790 1.00 0.00

ATOM 103 C65 KIH 1 23.140 16.390 26.040 1.00 0.00

ATOM 104 C66 KIH 1 22.970 15.030 26.210 1.00 0.00

ATOM 105 C67 KIH 1 23.180 14.180 25.150 1.00 0.00

ATOM 106 C68 KIH 1 23.340 14.730 23.900 1.00 0.00

ATOM 107 C69 KIH 1 23.520 13.840 22.850 1.00 0.00

ATOM 108 C70 KIH 1 23.740 14.340 21.590 1.00 0.00

ATOM 109 C71 KIH 1 23.840 15.690 21.380 1.00 0.00

ATOM 110 C72 KIH 1 24.240 16.140 20.130 1.00 0.00

ATOM 111 C73 KIH 1 24.320 17.470 19.810 1.00 0.00

ATOM 112 H29 KIH 1 26.500 19.680 21.700 1.00 0.00

ATOM 113 H30 KIH 1 27.830 21.570 22.180 1.00 0.00

ATOM 114 H31 KIH 1 28.570 23.780 21.420 1.00 0.00

ATOM 115 H32 KIH 1 26.990 25.210 20.060 1.00 0.00

ATOM 116 H33 KIH 1 24.820 24.260 19.390 1.00 0.00

ATOM 117 H34 KIH 1 24.310 18.530 23.020 1.00 0.00

ATOM 118 H35 KIH 1 23.590 17.960 24.790 1.00 0.00

ATOM 119 H36 KIH 1 23.020 17.040 26.890 1.00 0.00

ATOM 120 H37 KIH 1 22.720 14.690 27.200 1.00 0.00

ATOM 121 H38 KIH 1 23.080 13.120 25.330 1.00 0.00

ATOM 122 H39 KIH 1 23.440 12.770 22.860 1.00 0.00

ATOM 123 H40 KIH 1 23.850 13.660 20.760 1.00 0.00

ATOM 124 H41 KIH 1 24.240 15.440 19.310 1.00 0.00

ATOM 125 H42 KIH 1 24.640 17.740 18.820 1.00 0.00

ATOM 126 O5 KIH 1 22.500 19.990 19.640 1.00 0.00

ATOM 127 O6 KIH 1 21.760 19.200 23.960 1.00 0.00

ATOM 128 C74 KIH 1 23.110 22.470 18.740 1.00 0.00

ATOM 129 C75 KIH 1 21.770 22.630 19.030 1.00 0.00

ATOM 130 C76 KIH 1 20.930 23.370 18.220 1.00 0.00

ATOM 131 C77 KIH 1 21.360 23.690 16.960 1.00 0.00

ATOM 132 C78 KIH 1 22.650 23.440 16.590 1.00 0.00

ATOM 133 C79 KIH 1 23.500 22.810 17.470 1.00 0.00

ATOM 134 C80 KIH 1 22.970 23.750 15.290 1.00 0.00

ATOM 135 C81 KIH 1 24.270 23.520 14.890 1.00 0.00

ATOM 136 C82 KIH 1 25.170 22.910 15.740 1.00 0.00

ATOM 137 C83 KIH 1 24.750 22.470 16.960 1.00 0.00

ATOM 138 C84 KIH 1 19.510 23.640 18.570 1.00 0.00

ATOM 139 C85 KIH 1 19.240 24.370 19.710 1.00 0.00

ATOM 140 C86 KIH 1 18.010 24.750 20.180 1.00 0.00

ATOM 141 C87 KIH 1 17.850 25.390 21.390 1.00 0.00

ATOM 142 C88 KIH 1 18.860 25.350 22.320 1.00 0.00

ATOM 143 C89 KIH 1 18.720 25.900 23.580 1.00 0.00

ATOM 144 C90 KIH 1 17.520 26.540 23.840 1.00 0.00

ATOM 145 C91 KIH 1 16.460 26.480 22.970 1.00 0.00

ATOM 146 C92 KIH 1 16.630 25.930 21.720 1.00 0.00

ATOM 147 C93 KIH 1 15.580 25.700 20.870 1.00 0.00

ATOM 148 C94 KIH 1 15.680 24.970 19.710 1.00 0.00

ATOM 149 C95 KIH 1 16.930 24.530 19.350 1.00 0.00

ATOM 150 C96 KIH 1 17.110 23.790 18.210 1.00 0.00

ATOM 151 C97 KIH 1 18.370 23.410 17.820 1.00 0.00

ATOM 152 H43 KIH 1 20.590 24.150 16.350 1.00 0.00

ATOM 153 H44 KIH 1 22.340 24.360 14.660 1.00 0.00

ATOM 154 H45 KIH 1 24.390 23.830 13.860 1.00 0.00

ATOM 155 H46 KIH 1 26.140 22.730 15.320 1.00 0.00

ATOM 156 H47 KIH 1 25.470 22.000 17.630 1.00 0.00

ATOM 157 H48 KIH 1 20.160 24.740 20.140 1.00 0.00

ATOM 158 H49 KIH 1 19.800 24.870 22.090 1.00 0.00

ATOM 159 H50 KIH 1 19.540 25.890 24.270 1.00 0.00

ATOM 160 H51 KIH 1 17.300 26.850 24.850 1.00 0.00

ATOM 161 H52 KIH 1 15.480 26.870 23.250 1.00 0.00

ATOM 162 H53 KIH 1 14.600 26.100 21.110 1.00 0.00

ATOM 163 H54 KIH 1 14.760 24.820 19.170 1.00 0.00

ATOM 164 H55 KIH 1 16.290 23.650 17.510 1.00 0.00

ATOM 165 H56 KIH 1 18.500 23.000 16.830 1.00 0.00

ATOM 166 O7 KIH 1 21.210 22.120 20.180 1.00 0.00

ATOM 167 O8 KIH 1 22.690 21.270 22.870 1.00 0.00

ATOM 168 C98 KIH 1 20.310 21.370 23.930 1.00 0.00

ATOM 169 F4 KIH 1 20.710 21.960 25.070 1.00 0.00

ATOM 170 F5 KIH 1 19.910 22.420 23.180 1.00 0.00

ATOM 171 F6 KIH 1 19.180 20.760 24.280 1.00 0.00

ATOM 172 N2 KIH 1 21.030 19.940 21.680 1.00 0.00

ATOM 173 P2 KIH 1 21.100 20.540 20.140 1.00 0.00

ATOM 174 S2 KIH 1 21.590 20.380 23.170 1.00 0.00

ATOM 175 N3 KIH 1 19.880 19.970 19.490 1.00 0.00

ATOM 176 H57 KIH 1 20.670 19.000 21.740 1.00 0.00

TER

ENDMDL

REMARK GENERATED BY TRJCONV

TITLE KIH t= 9500.00000

REMARK THIS IS A SIMULATION BOX

CRYST1 38.494 38.494 38.494 90.00 90.00 90.00 P 1 1

MODEL 20

ATOM 1 C1 KIH 1 16.030 19.510 17.290 1.00 0.00

ATOM 2 C2 KIH 1 16.200 20.030 18.550 1.00 0.00

ATOM 3 C3 KIH 1 15.180 19.970 19.470 1.00 0.00

ATOM 4 C4 KIH 1 13.960 19.450 19.120 1.00 0.00

ATOM 5 C5 KIH 1 13.780 18.870 17.880 1.00 0.00

ATOM 6 C6 KIH 1 14.850 18.860 17.020 1.00 0.00

ATOM 7 C7 KIH 1 12.550 18.420 17.490 1.00 0.00

ATOM 8 C8 KIH 1 12.390 17.800 16.270 1.00 0.00

ATOM 9 C9 KIH 1 13.460 17.740 15.410 1.00 0.00

ATOM 10 C10 KIH 1 14.650 18.360 15.760 1.00 0.00

ATOM 11 C11 KIH 1 15.440 20.640 20.780 1.00 0.00

ATOM 12 C12 KIH 1 15.210 19.940 21.930 1.00 0.00

ATOM 13 C13 KIH 1 15.650 20.420 23.150 1.00 0.00

ATOM 14 C14 KIH 1 15.820 19.530 24.180 1.00 0.00

ATOM 15 C15 KIH 1 15.620 18.170 24.200 1.00 0.00

ATOM 16 C16 KIH 1 15.720 17.390 25.330 1.00 0.00

ATOM 17 C17 KIH 1 16.170 18.000 26.480 1.00 0.00

ATOM 18 C18 KIH 1 16.500 19.320 26.490 1.00 0.00

ATOM 19 C19 KIH 1 16.370 20.040 25.330 1.00 0.00

ATOM 20 C20 KIH 1 16.730 21.370 25.430 1.00 0.00

ATOM 21 C21 KIH 1 16.580 22.240 24.370 1.00 0.00

ATOM 22 C22 KIH 1 16.030 21.740 23.210 1.00 0.00

ATOM 23 C23 KIH 1 16.030 22.560 22.110 1.00 0.00

ATOM 24 C24 KIH 1 15.770 21.970 20.890 1.00 0.00

ATOM 25 H1 KIH 1 13.080 19.550 19.750 1.00 0.00

ATOM 26 H2 KIH 1 11.700 18.500 18.150 1.00 0.00

ATOM 27 H3 KIH 1 11.460 17.360 15.940 1.00 0.00

ATOM 28 H4 KIH 1 13.390 17.220 14.470 1.00 0.00

ATOM 29 H5 KIH 1 15.430 18.350 14.990 1.00 0.00

ATOM 30 H6 KIH 1 14.910 18.920 21.810 1.00 0.00

ATOM 31 H7 KIH 1 15.180 17.690 23.330 1.00 0.00

ATOM 32 H8 KIH 1 15.470 16.340 25.370 1.00 0.00

ATOM 33 H9 KIH 1 16.280 17.360 27.350 1.00 0.00

ATOM 34 H10 KIH 1 16.910 19.680 27.440 1.00 0.00

ATOM 35 H11 KIH 1 17.180 21.790 26.300 1.00 0.00

ATOM 36 H12 KIH 1 16.950 23.260 24.330 1.00 0.00

ATOM 37 H13 KIH 1 16.230 23.620 22.150 1.00 0.00

ATOM 38 H14 KIH 1 15.980 22.490 19.970 1.00 0.00

ATOM 39 O1 KIH 1 17.490 20.370 18.910 1.00 0.00

ATOM 40 O2 KIH 1 19.260 18.550 22.660 1.00 0.00

ATOM 41 C25 KIH 1 17.090 19.750 16.260 1.00 0.00

ATOM 42 C26 KIH 1 18.270 19.090 16.460 1.00 0.00

ATOM 43 C27 KIH 1 19.280 19.160 15.510 1.00 0.00

ATOM 44 C28 KIH 1 19.170 20.150 14.560 1.00 0.00

ATOM 45 C29 KIH 1 17.960 20.750 14.320 1.00 0.00

ATOM 46 C30 KIH 1 16.900 20.610 15.200 1.00 0.00

ATOM 47 C31 KIH 1 17.860 21.520 13.190 1.00 0.00

ATOM 48 C32 KIH 1 16.680 22.140 12.860 1.00 0.00

ATOM 49 C33 KIH 1 15.640 22.060 13.760 1.00 0.00

ATOM 50 C34 KIH 1 15.790 21.380 14.940 1.00 0.00

ATOM 51 C35 KIH 1 20.560 18.410 15.630 1.00 0.00

ATOM 52 C36 KIH 1 20.620 17.110 15.170 1.00 0.00

ATOM 53 C37 KIH 1 21.800 16.410 15.200 1.00 0.00

ATOM 54 C38 KIH 1 21.940 15.150 14.640 1.00 0.00

ATOM 55 C39 KIH 1 20.920 14.490 14.010 1.00 0.00

ATOM 56 C40 KIH 1 21.040 13.170 13.590 1.00 0.00

ATOM 57 C41 KIH 1 22.300 12.630 13.520 1.00 0.00

ATOM 58 C42 KIH 1 23.360 13.240 14.150 1.00 0.00

ATOM 59 C43 KIH 1 23.160 14.510 14.630 1.00 0.00

ATOM 60 C44 KIH 1 24.210 15.110 15.290 1.00 0.00

ATOM 61 C45 KIH 1 24.110 16.320 15.940 1.00 0.00

ATOM 62 C46 KIH 1 22.890 16.960 15.810 1.00 0.00

ATOM 63 C47 KIH 1 22.790 18.220 16.330 1.00 0.00

ATOM 64 C48 KIH 1 21.690 19.020 16.090 1.00 0.00

ATOM 65 H15 KIH 1 20.030 20.330 13.940 1.00 0.00

ATOM 66 H16 KIH 1 18.710 21.700 12.560 1.00 0.00

ATOM 67 H17 KIH 1 16.410 22.760 12.010 1.00 0.00

ATOM 68 H18 KIH 1 14.710 22.580 13.550 1.00 0.00

ATOM 69 H19 KIH 1 15.010 21.380 15.670 1.00 0.00

ATOM 70 H20 KIH 1 19.680 16.690 14.810 1.00 0.00

ATOM 71 H21 KIH 1 19.940 14.900 13.870 1.00 0.00

ATOM 72 H22 KIH 1 20.190 12.540 13.390 1.00 0.00

ATOM 73 H23 KIH 1 22.390 11.690 12.990 1.00 0.00

ATOM 74 H24 KIH 1 24.270 12.660 14.150 1.00 0.00

ATOM 75 H25 KIH 1 25.170 14.600 15.250 1.00 0.00

ATOM 76 H26 KIH 1 24.940 16.790 16.450 1.00 0.00

ATOM 77 H27 KIH 1 23.660 18.720 16.740 1.00 0.00

ATOM 78 H28 KIH 1 21.640 20.080 16.330 1.00 0.00

ATOM 79 O3 KIH 1 18.560 18.490 17.670 1.00 0.00

ATOM 80 O4 KIH 1 17.640 16.710 22.580 1.00 0.00

ATOM 81 C49 KIH 1 20.010 16.390 21.620 1.00 0.00

ATOM 82 F1 KIH 1 19.520 15.200 21.250 1.00 0.00

ATOM 83 F2 KIH 1 20.880 16.770 20.690 1.00 0.00

ATOM 84 F3 KIH 1 20.730 16.200 22.740 1.00 0.00

ATOM 85 N1 KIH 1 18.090 18.160 20.430 1.00 0.00

ATOM 86 P1 KIH 1 18.570 19.220 19.060 1.00 0.00

ATOM 87 S1 KIH 1 18.630 17.500 21.900 1.00 0.00

ATOM 88 C50 KIH 1 23.890 21.570 19.240 1.00 0.00

ATOM 89 C51 KIH 1 23.570 20.300 19.690 1.00 0.00

ATOM 90 C52 KIH 1 24.490 19.580 20.420 1.00 0.00

ATOM 91 C53 KIH 1 25.660 20.210 20.790 1.00 0.00

ATOM 92 C54 KIH 1 25.950 21.480 20.340 1.00 0.00

ATOM 93 C55 KIH 1 25.050 22.190 19.600 1.00 0.00

ATOM 94 C56 KIH 1 27.160 22.050 20.670 1.00 0.00

ATOM 95 C57 KIH 1 27.550 23.280 20.220 1.00 0.00

ATOM 96 C58 KIH 1 26.570 24.060 19.640 1.00 0.00

ATOM 97 C59 KIH 1 25.390 23.480 19.240 1.00 0.00

ATOM 98 C60 KIH 1 24.220 18.230 20.980 1.00 0.00

ATOM 99 C61 KIH 1 24.180 17.990 22.330 1.00 0.00

ATOM 100 C62 KIH 1 24.110 16.710 22.800 1.00 0.00

ATOM 101 C63 KIH 1 24.210 16.460 24.150 1.00 0.00

ATOM 102 C64 KIH 1 24.260 17.440 25.110 1.00 0.00

ATOM 103 C65 KIH 1 24.310 17.160 26.460 1.00 0.00

ATOM 104 C66 KIH 1 24.400 15.830 26.810 1.00 0.00

ATOM 105 C67 KIH 1 24.260 14.810 25.900 1.00 0.00

ATOM 106 C68 KIH 1 24.250 15.150 24.560 1.00 0.00

ATOM 107 C69 KIH 1 24.130 14.130 23.650 1.00 0.00

ATOM 108 C70 KIH 1 23.800 14.380 22.350 1.00 0.00

ATOM 109 C71 KIH 1 23.900 15.690 21.900 1.00 0.00

ATOM 110 C72 KIH 1 23.790 15.930 20.550 1.00 0.00

ATOM 111 C73 KIH 1 24.060 17.200 20.100 1.00 0.00

ATOM 112 H29 KIH 1 26.350 19.640 21.400 1.00 0.00

ATOM 113 H30 KIH 1 27.900 21.570 21.300 1.00 0.00

ATOM 114 H31 KIH 1 28.570 23.600 20.350 1.00 0.00

ATOM 115 H32 KIH 1 26.770 25.090 19.410 1.00 0.00

ATOM 116 H33 KIH 1 24.680 24.100 18.730 1.00 0.00

ATOM 117 H34 KIH 1 24.390 18.910 22.870 1.00 0.00

ATOM 118 H35 KIH 1 24.280 18.470 24.800 1.00 0.00

ATOM 119 H36 KIH 1 24.320 17.990 27.150 1.00 0.00

ATOM 120 H37 KIH 1 24.460 15.570 27.860 1.00 0.00

ATOM 121 H38 KIH 1 24.320 13.790 26.230 1.00 0.00

ATOM 122 H39 KIH 1 24.110 13.090 23.920 1.00 0.00

ATOM 123 H40 KIH 1 23.640 13.590 21.630 1.00 0.00

ATOM 124 H41 KIH 1 23.630 15.160 19.820 1.00 0.00

ATOM 125 H42 KIH 1 24.120 17.440 19.050 1.00 0.00

ATOM 126 O5 KIH 1 22.320 19.760 19.530 1.00 0.00

ATOM 127 O6 KIH 1 22.060 19.300 24.000 1.00 0.00

ATOM 128 C74 KIH 1 22.930 22.170 18.270 1.00 0.00

ATOM 129 C75 KIH 1 21.680 22.510 18.750 1.00 0.00

ATOM 130 C76 KIH 1 20.800 23.150 17.890 1.00 0.00

ATOM 131 C77 KIH 1 21.180 23.410 16.590 1.00 0.00

ATOM 132 C78 KIH 1 22.430 23.020 16.160 1.00 0.00

ATOM 133 C79 KIH 1 23.320 22.340 16.960 1.00 0.00

ATOM 134 C80 KIH 1 22.710 23.400 14.870 1.00 0.00

ATOM 135 C81 KIH 1 23.860 22.930 14.300 1.00 0.00

ATOM 136 C82 KIH 1 24.810 22.280 15.050 1.00 0.00

ATOM 137 C83 KIH 1 24.530 22.030 16.380 1.00 0.00

ATOM 138 C84 KIH 1 19.440 23.530 18.370 1.00 0.00

ATOM 139 C85 KIH 1 19.370 24.330 19.480 1.00 0.00

ATOM 140 C86 KIH 1 18.160 24.810 19.950 1.00 0.00

ATOM 141 C87 KIH 1 18.090 25.660 21.020 1.00 0.00

ATOM 142 C88 KIH 1 19.060 25.680 21.990 1.00 0.00

ATOM 143 C89 KIH 1 18.990 26.520 23.080 1.00 0.00

ATOM 144 C90 KIH 1 17.910 27.380 23.200 1.00 0.00

ATOM 145 C91 KIH 1 16.960 27.390 22.200 1.00 0.00

ATOM 146 C92 KIH 1 16.990 26.480 21.170 1.00 0.00

ATOM 147 C93 KIH 1 15.960 26.460 20.250 1.00 0.00

ATOM 148 C94 KIH 1 15.960 25.420 19.360 1.00 0.00

ATOM 149 C95 KIH 1 17.070 24.620 19.140 1.00 0.00

ATOM 150 C96 KIH 1 17.110 23.860 17.990 1.00 0.00

ATOM 151 C97 KIH 1 18.290 23.250 17.670 1.00 0.00

ATOM 152 H43 KIH 1 20.490 23.970 15.990 1.00 0.00

ATOM 153 H44 KIH 1 21.980 23.960 14.300 1.00 0.00

ATOM 154 H45 KIH 1 24.020 23.320 13.290 1.00 0.00

ATOM 155 H46 KIH 1 25.730 22.040 14.560 1.00 0.00

ATOM 156 H47 KIH 1 25.260 21.550 17.000 1.00 0.00

ATOM 157 H48 KIH 1 20.300 24.590 19.960 1.00 0.00

ATOM 158 H49 KIH 1 19.950 25.070 22.000 1.00 0.00

ATOM 159 H50 KIH 1 19.810 26.680 23.760 1.00 0.00

ATOM 160 H51 KIH 1 17.780 28.060 24.020 1.00 0.00

ATOM 161 H52 KIH 1 16.140 28.060 22.300 1.00 0.00

ATOM 162 H53 KIH 1 15.080 27.030 20.470 1.00 0.00

ATOM 163 H54 KIH 1 15.080 25.440 18.750 1.00 0.00

ATOM 164 H55 KIH 1 16.280 23.790 17.310 1.00 0.00

ATOM 165 H56 KIH 1 18.490 22.700 16.760 1.00 0.00

ATOM 166 O7 KIH 1 21.170 22.010 19.930 1.00 0.00

ATOM 167 O8 KIH 1 22.730 21.380 22.880 1.00 0.00

ATOM 168 C98 KIH 1 20.320 21.220 24.130 1.00 0.00

ATOM 169 F4 KIH 1 20.530 21.530 25.420 1.00 0.00

ATOM 170 F5 KIH 1 20.010 22.410 23.590 1.00 0.00

ATOM 171 F6 KIH 1 19.170 20.550 24.160 1.00 0.00

ATOM 172 N2 KIH 1 21.100 20.010 21.760 1.00 0.00

ATOM 173 P2 KIH 1 21.060 20.450 20.180 1.00 0.00

ATOM 174 S2 KIH 1 21.680 20.470 23.240 1.00 0.00

ATOM 175 N3 KIH 1 19.880 19.870 19.450 1.00 0.00

ATOM 176 H57 KIH 1 20.740 19.070 21.880 1.00 0.00

TER

ENDMDL

REMARK GENERATED BY TRJCONV

TITLE KIH t= 10000.00000

REMARK THIS IS A SIMULATION BOX

CRYST1 38.494 38.494 38.494 90.00 90.00 90.00 P 1 1

MODEL 21

ATOM 1 C1 KIH 1 16.350 19.750 17.030 1.00 0.00

ATOM 2 C2 KIH 1 16.360 20.050 18.370 1.00 0.00

ATOM 3 C3 KIH 1 15.270 19.850 19.170 1.00 0.00

ATOM 4 C4 KIH 1 14.110 19.370 18.600 1.00 0.00

ATOM 5 C5 KIH 1 14.080 18.970 17.280 1.00 0.00

ATOM 6 C6 KIH 1 15.200 19.190 16.510 1.00 0.00

ATOM 7 C7 KIH 1 12.930 18.430 16.750 1.00 0.00

ATOM 8 C8 KIH 1 12.960 17.930 15.460 1.00 0.00

ATOM 9 C9 KIH 1 14.110 17.980 14.720 1.00 0.00

ATOM 10 C10 KIH 1 15.180 18.680 15.240 1.00 0.00

ATOM 11 C11 KIH 1 15.330 19.950 20.660 1.00 0.00

ATOM 12 C12 KIH 1 14.960 18.910 21.490 1.00 0.00

ATOM 13 C13 KIH 1 14.880 18.980 22.860 1.00 0.00

ATOM 14 C14 KIH 1 14.530 17.920 23.670 1.00 0.00

ATOM 15 C15 KIH 1 13.940 16.790 23.170 1.00 0.00

ATOM 16 C16 KIH 1 13.780 15.730 24.030 1.00 0.00

ATOM 17 C17 KIH 1 14.010 15.790 25.380 1.00 0.00

ATOM 18 C18 KIH 1 14.540 16.970 25.890 1.00 0.00

ATOM 19 C19 KIH 1 14.810 17.990 25.010 1.00 0.00

ATOM 20 C20 KIH 1 15.460 19.080 25.530 1.00 0.00

ATOM 21 C21 KIH 1 15.860 20.110 24.710 1.00 0.00

ATOM 22 C22 KIH 1 15.540 20.070 23.370 1.00 0.00

ATOM 23 C23 KIH 1 15.870 21.150 22.590 1.00 0.00

ATOM 24 C24 KIH 1 15.770 21.120 21.220 1.00 0.00

ATOM 25 H1 KIH 1 13.210 19.090 19.120 1.00 0.00

ATOM 26 H2 KIH 1 12.070 18.340 17.390 1.00 0.00

ATOM 27 H3 KIH 1 12.110 17.340 15.150 1.00 0.00

ATOM 28 H4 KIH 1 14.140 17.470 13.770 1.00 0.00

ATOM 29 H5 KIH 1 16.030 18.730 14.590 1.00 0.00

ATOM 30 H6 KIH 1 14.640 18.010 20.990 1.00 0.00

ATOM 31 H7 KIH 1 13.590 16.790 22.150 1.00 0.00

ATOM 32 H8 KIH 1 13.470 14.830 23.510 1.00 0.00

ATOM 33 H9 KIH 1 13.870 15.000 26.100 1.00 0.00

ATOM 34 H10 KIH 1 14.760 16.990 26.950 1.00 0.00

ATOM 35 H11 KIH 1 15.710 19.170 26.580 1.00 0.00

ATOM 36 H12 KIH 1 16.420 20.950 25.100 1.00 0.00

ATOM 37 H13 KIH 1 16.050 22.120 23.020 1.00 0.00

ATOM 38 H14 KIH 1 16.080 21.950 20.600 1.00 0.00

ATOM 39 O1 KIH 1 17.560 20.390 18.980 1.00 0.00

ATOM 40 O2 KIH 1 19.430 18.070 22.690 1.00 0.00

ATOM 41 C25 KIH 1 17.570 20.050 16.230 1.00 0.00

ATOM 42 C26 KIH 1 18.710 19.390 16.630 1.00 0.00

ATOM 43 C27 KIH 1 19.820 19.550 15.810 1.00 0.00

ATOM 44 C28 KIH 1 19.800 20.410 14.740 1.00 0.00

ATOM 45 C29 KIH 1 18.630 21.010 14.350 1.00 0.00

ATOM 46 C30 KIH 1 17.540 20.950 15.200 1.00 0.00

ATOM 47 C31 KIH 1 18.530 21.760 13.210 1.00 0.00

ATOM 48 C32 KIH 1 17.370 22.440 12.900 1.00 0.00

ATOM 49 C33 KIH 1 16.370 22.490 13.840 1.00 0.00

ATOM 50 C34 KIH 1 16.420 21.740 15.000 1.00 0.00

ATOM 51 C35 KIH 1 21.070 18.750 15.860 1.00 0.00

ATOM 52 C36 KIH 1 21.090 17.430 15.450 1.00 0.00

ATOM 53 C37 KIH 1 22.230 16.720 15.190 1.00 0.00

ATOM 54 C38 KIH 1 22.230 15.490 14.570 1.00 0.00

ATOM 55 C39 KIH 1 21.100 14.830 14.150 1.00 0.00

ATOM 56 C40 KIH 1 21.080 13.560 13.600 1.00 0.00

ATOM 57 C41 KIH 1 22.290 12.980 13.300 1.00 0.00

ATOM 58 C42 KIH 1 23.430 13.560 13.810 1.00 0.00

ATOM 59 C43 KIH 1 23.420 14.810 14.390 1.00 0.00

ATOM 60 C44 KIH 1 24.610 15.410 14.700 1.00 0.00

ATOM 61 C45 KIH 1 24.590 16.650 15.310 1.00 0.00

ATOM 62 C46 KIH 1 23.400 17.290 15.580 1.00 0.00

ATOM 63 C47 KIH 1 23.430 18.590 16.050 1.00 0.00

ATOM 64 C48 KIH 1 22.280 19.320 16.200 1.00 0.00

ATOM 65 H15 KIH 1 20.650 20.500 14.080 1.00 0.00

ATOM 66 H16 KIH 1 19.390 21.800 12.570 1.00 0.00

ATOM 67 H17 KIH 1 17.240 22.940 11.940 1.00 0.00

ATOM 68 H18 KIH 1 15.530 23.110 13.600 1.00 0.00

ATOM 69 H19 KIH 1 15.510 21.700 15.570 1.00 0.00

ATOM 70 H20 KIH 1 20.110 17.040 15.180 1.00 0.00

ATOM 71 H21 KIH 1 20.140 15.320 14.070 1.00 0.00

ATOM 72 H22 KIH 1 20.120 13.180 13.290 1.00 0.00

ATOM 73 H23 KIH 1 22.300 12.020 12.820 1.00 0.00

ATOM 74 H24 KIH 1 24.370 13.010 13.810 1.00 0.00

ATOM 75 H25 KIH 1 25.460 14.820 14.380 1.00 0.00

ATOM 76 H26 KIH 1 25.560 17.090 15.460 1.00 0.00

ATOM 77 H27 KIH 1 24.390 19.010 16.320 1.00 0.00

ATOM 78 H28 KIH 1 22.340 20.350 16.530 1.00 0.00

ATOM 79 O3 KIH 1 18.790 18.550 17.720 1.00 0.00

ATOM 80 O4 KIH 1 17.360 16.790 22.650 1.00 0.00

ATOM 81 C49 KIH 1 19.440 15.920 21.390 1.00 0.00

ATOM 82 F1 KIH 1 18.950 15.330 20.300 1.00 0.00

ATOM 83 F2 KIH 1 20.690 16.310 21.110 1.00 0.00

ATOM 84 F3 KIH 1 19.540 15.000 22.360 1.00 0.00

ATOM 85 N1 KIH 1 18.100 18.200 20.480 1.00 0.00

ATOM 86 P1 KIH 1 18.640 19.240 19.130 1.00 0.00

ATOM 87 S1 KIH 1 18.470 17.330 21.910 1.00 0.00

ATOM 88 C50 KIH 1 23.890 21.870 19.400 1.00 0.00

ATOM 89 C51 KIH 1 23.670 20.530 19.650 1.00 0.00

ATOM 90 C52 KIH 1 24.660 19.810 20.270 1.00 0.00

ATOM 91 C53 KIH 1 25.810 20.460 20.710 1.00 0.00

ATOM 92 C54 KIH 1 25.980 21.800 20.540 1.00 0.00

ATOM 93 C55 KIH 1 24.960 22.520 19.970 1.00 0.00

ATOM 94 C56 KIH 1 27.090 22.470 21.020 1.00 0.00

ATOM 95 C57 KIH 1 27.340 23.790 20.740 1.00 0.00

ATOM 96 C58 KIH 1 26.300 24.510 20.170 1.00 0.00

ATOM 97 C59 KIH 1 25.130 23.880 19.840 1.00 0.00

ATOM 98 C60 KIH 1 24.380 18.350 20.420 1.00 0.00

ATOM 99 C61 KIH 1 24.110 17.900 21.690 1.00 0.00

ATOM 100 C62 KIH 1 23.950 16.570 22.020 1.00 0.00

ATOM 101 C63 KIH 1 23.860 15.990 23.250 1.00 0.00

ATOM 102 C64 KIH 1 23.690 16.740 24.390 1.00 0.00

ATOM 103 C65 KIH 1 23.380 16.200 25.620 1.00 0.00

ATOM 104 C66 KIH 1 23.470 14.850 25.830 1.00 0.00

ATOM 105 C67 KIH 1 23.730 14.060 24.740 1.00 0.00

ATOM 106 C68 KIH 1 23.800 14.640 23.500 1.00 0.00

ATOM 107 C69 KIH 1 23.910 13.790 22.410 1.00 0.00

ATOM 108 C70 KIH 1 24.090 14.300 21.150 1.00 0.00

ATOM 109 C71 KIH 1 24.100 15.670 20.990 1.00 0.00

ATOM 110 C72 KIH 1 24.240 16.100 19.700 1.00 0.00

ATOM 111 C73 KIH 1 24.350 17.450 19.400 1.00 0.00

ATOM 112 H29 KIH 1 26.660 19.880 21.040 1.00 0.00

ATOM 113 H30 KIH 1 27.820 21.890 21.560 1.00 0.00

ATOM 114 H31 KIH 1 28.220 24.350 21.010 1.00 0.00

ATOM 115 H32 KIH 1 26.510 25.550 19.970 1.00 0.00

ATOM 116 H33 KIH 1 24.360 24.520 19.440 1.00 0.00

ATOM 117 H34 KIH 1 24.110 18.630 22.490 1.00 0.00

ATOM 118 H35 KIH 1 23.640 17.810 24.370 1.00 0.00

ATOM 119 H36 KIH 1 23.160 16.740 26.530 1.00 0.00

ATOM 120 H37 KIH 1 23.410 14.420 26.820 1.00 0.00

ATOM 121 H38 KIH 1 23.720 12.990 24.860 1.00 0.00

ATOM 122 H39 KIH 1 23.980 12.720 22.490 1.00 0.00

ATOM 123 H40 KIH 1 24.290 13.700 20.280 1.00 0.00

ATOM 124 H41 KIH 1 24.310 15.430 18.850 1.00 0.00

ATOM 125 H42 KIH 1 24.570 17.770 18.390 1.00 0.00

ATOM 126 O5 KIH 1 22.450 19.900 19.560 1.00 0.00

ATOM 127 O6 KIH 1 22.030 19.390 23.960 1.00 0.00

ATOM 128 C74 KIH 1 22.880 22.500 18.480 1.00 0.00

ATOM 129 C75 KIH 1 21.590 22.700 18.910 1.00 0.00

ATOM 130 C76 KIH 1 20.650 23.270 18.080 1.00 0.00

ATOM 131 C77 KIH 1 21.030 23.430 16.760 1.00 0.00

ATOM 132 C78 KIH 1 22.300 23.210 16.280 1.00 0.00

ATOM 133 C79 KIH 1 23.250 22.820 17.210 1.00 0.00

ATOM 134 C80 KIH 1 22.510 23.250 14.920 1.00 0.00

ATOM 135 C81 KIH 1 23.770 22.970 14.460 1.00 0.00

ATOM 136 C82 KIH 1 24.760 22.640 15.350 1.00 0.00

ATOM 137 C83 KIH 1 24.510 22.640 16.710 1.00 0.00

ATOM 138 C84 KIH 1 19.380 23.710 18.700 1.00 0.00

ATOM 139 C85 KIH 1 19.310 24.610 19.730 1.00 0.00

ATOM 140 C86 KIH 1 18.120 24.950 20.340 1.00 0.00

ATOM 141 C87 KIH 1 18.100 25.710 21.480 1.00 0.00

ATOM 142 C88 KIH 1 19.190 26.160 22.190 1.00 0.00

ATOM 143 C89 KIH 1 19.040 26.900 23.350 1.00 0.00

ATOM 144 C90 KIH 1 17.810 27.350 23.750 1.00 0.00

ATOM 145 C91 KIH 1 16.740 27.030 22.950 1.00 0.00

ATOM 146 C92 KIH 1 16.860 26.130 21.930 1.00 0.00

ATOM 147 C93 KIH 1 15.700 25.620 21.410 1.00 0.00

ATOM 148 C94 KIH 1 15.770 24.690 20.400 1.00 0.00

ATOM 149 C95 KIH 1 16.980 24.360 19.830 1.00 0.00

ATOM 150 C96 KIH 1 17.070 23.550 18.730 1.00 0.00

ATOM 151 C97 KIH 1 18.260 23.080 18.210 1.00 0.00

ATOM 152 H43 KIH 1 20.290 23.810 16.060 1.00 0.00

ATOM 153 H44 KIH 1 21.680 23.490 14.260 1.00 0.00

ATOM 154 H45 KIH 1 24.080 23.080 13.420 1.00 0.00

ATOM 155 H46 KIH 1 25.730 22.360 14.960 1.00 0.00

ATOM 156 H47 KIH 1 25.390 22.340 17.260 1.00 0.00

ATOM 157 H48 KIH 1 20.250 24.960 20.110 1.00 0.00

ATOM 158 H49 KIH 1 20.190 25.850 21.930 1.00 0.00

ATOM 159 H50 KIH 1 19.920 27.070 23.940 1.00 0.00

ATOM 160 H51 KIH 1 17.820 27.990 24.620 1.00 0.00

ATOM 161 H52 KIH 1 15.800 27.480 23.230 1.00 0.00

ATOM 162 H53 KIH 1 14.700 25.850 21.760 1.00 0.00

ATOM 163 H54 KIH 1 14.810 24.330 20.040 1.00 0.00

ATOM 164 H55 KIH 1 16.150 23.120 18.370 1.00 0.00

ATOM 165 H56 KIH 1 18.310 22.300 17.460 1.00 0.00

ATOM 166 O7 KIH 1 21.150 22.110 20.090 1.00 0.00

ATOM 167 O8 KIH 1 22.750 21.450 22.890 1.00 0.00

ATOM 168 C98 KIH 1 20.310 21.410 23.860 1.00 0.00

ATOM 169 F4 KIH 1 20.600 22.090 24.980 1.00 0.00

ATOM 170 F5 KIH 1 19.840 22.320 23.000 1.00 0.00

ATOM 171 F6 KIH 1 19.300 20.580 24.160 1.00 0.00

ATOM 172 N2 KIH 1 21.070 19.970 21.720 1.00 0.00

ATOM 173 P2 KIH 1 21.130 20.530 20.170 1.00 0.00

ATOM 174 S2 KIH 1 21.690 20.520 23.150 1.00 0.00

ATOM 175 N3 KIH 1 19.930 19.920 19.500 1.00 0.00

ATOM 176 H57 KIH 1 20.530 19.120 21.810 1.00 0.00

TER

ENDMDL
